# Link between short tandem repeats and translation initiation site selection

**DOI:** 10.1186/s40246-018-0181-3

**Published:** 2018-10-29

**Authors:** Masoud Arabfard, Kaveh Kavousi, Ahmad Delbari, Mina Ohadi

**Affiliations:** 1 Department of Bioinformatics, Kish International Campus University of Tehran, Kish, Iran; 2 Laboratory of Complex Biological Systems and Bioinformatics (CBB), Department of Bioinformatics, Institute of Biochemistry and Biophysics (IBB), University of Tehran, Tehran, Iran; 3 Iranian Research Center on Aging, University of Social Welfare and Rehabilitation Sciences, Tehran, Iran

**Keywords:** Translation initiation site, Short tandem repeat, Genome-scale, Human-specific, Selection

## Abstract

**Background:**

Despite their vast biological implication, the relevance of short tandem repeats (STRs)/microsatellites to the protein-coding gene translation initiation sites (TISs) remains largely unknown.

**Methods:**

We performed an Ensembl-based comparative genomics study of all annotated orthologous TIS-flanking sequences in human and 46 other species across vertebrates, on the genomic DNA and cDNA platforms (755,956 TISs), aimed at identifying human-specific STRs in this interval. The collected data were used to examine the hypothesis of a link between STRs and TISs. BLAST was used to compare the initial five amino acids (excluding the initial methionine), codons of which were flanked by STRs in human, with the initial five amino acids of all annotated proteins for the orthologous genes in other vertebrates (total of 5,314,979 pair-wise TIS comparisons on the genomic DNA and cDNA platforms) in order to compare the number of events in which human-specific and non-specific STRs occurred with homologous and non-homologous TISs (i.e., ≥ 50% and < 50% similarity of the five amino acids).

**Results:**

We detected differential distribution of the human-specific STRs in comparison to the overall distribution of STRs on the genomic DNA and cDNA platforms (Mann Whitney *U* test *p* = 1.4 × 10^−11^ and *p* < 7.9 × 10^−11^, respectively). We also found excess occurrence of non-homologous TISs with human-specific STRs and excess occurrence of homologous TISs with non-specific STRs on both platforms (*p* < 0.00001).

**Conclusion:**

We propose a link between STRs and TIS selection, based on the differential co-occurrence rate of human-specific STRs with non-homologous TISs and non-specific STRs with homologous TISs.

**Electronic supplementary material:**

The online version of this article (10.1186/s40246-018-0181-3) contains supplementary material, which is available to authorized users.

## Introduction

An increasing number of human protein-coding genes are unraveled to consist of alternative translation initiation sites (TISs), which are selected based on complex and yet not fully known scanning mechanisms [1, 2]. The alternative TISs result in various protein structures and functions [3, 4]. Selection of TISs and the level of translation and protein synthesis depend partially on the *cis-*regulatory elements in the mRNA sequence and its secondary structure such as the formation of hair-pins and thermal stability [5–7]. Genomic DNA *cis-*elements can also affect translation and TISs through various mechanisms (for a review see [8]).

One of the important and understudied *cis*-regulatory elements affecting translation is short tandem repeats (STRs)/microsatellites. In physiological terms, STRs can dramatically influence TIS and the amount of protein synthesis. Poly(A) tracts in the 5′-untranslated region (UTR) are important sites for translation regulation in yeast. These poly(A) tracts can interact with translation initiation factors or poly(A) binding proteins (PABP) to either increase or decrease translation efficiency. Pre-AUG A_N_ can enhance internal ribosomal entry both in the presence of PABP and eIF-4G in *Saccharomyces cerevisiae* [9], and in the complete absence of PABP and eIF-4G [10]. Biased distribution of dinucleotide repeats is a known phenomenon in the region immediately upstream of the TISs in *Escherichia coli* [11]. In pathological instances, expansion of STRs in the RNA structure results in toxic RNAs and non-AUG translation and the development of several human-specific neurological [12–14].

Genomic DNA STRs can affect TISs through their effect on alternative splicing and shuffling of novel ATG translation sites in novel exons [15]. Genome-scale findings of the evolutionary trend of a number of STRs have begun to unfold their implication in respect to speciation and species-specific characteristics/phenotypes [16–27]. The hypermutable nature of STRs and their large unascertained reservoir of functionality make them an ideal source of evolutionary adaptation, speciation, and disease [28–35]. In line with the above, recent reports indicate a role of repetitive sequences in the creation of new transcription start sites (TSSs) in human [36–39].

This research aimed to examine a possible link between STRs and TIS selection through studying the occurrence rate of TIS-flanking human-specific and non-specific STRs with homologous and non-homologous proteins.

## Methods

### Data collection

Forty-seven species encompassing major classes of vertebrates were selected, and in each species, the 120 bp upstream genomic DNA and cDNA sequences flanking all annotated protein-coding TISs (*n* = 755,956) were downloaded based on the Ensembl database version 90 (https://asia.ensembl.org). The species studied are alphabetically listed as follows: anole lizard, armadillo, bush baby, cat, chicken, chimpanzee, cow, dog, dolphin, duck, ferret, fugu, gibbon, golden hamster, gorilla, guinea pig, hedgehog, human, horse, kangaroo rat, lamprey, lesser hedgehog tenrec, macaque, marmoset, megabat, microbat, mouse, mouse lemur, olive baboon, opossum, orangutan, pig, platypus, prairie vole, rabbit, rat, sheep, shrew, shrew mouse, squirrel, tarsier, Tasmanian devil, tree shrew, turkey, vervet-AGM, Xenopus, and zebrafish.

For each gene in each species, its Ensembl ID, all the annotated transcript IDs, the genomic DNA sequence, the cDNA, and the coding DNA sequence (CDS) were retrieved (the list of genes is available upon request). The genomic DNA, CDS, and the annotated cDNAs were downloaded using REST API from the Ensembl database. The first start codon for each transcript was determined using BLAST between the CDS and cDNA. The 120 bp genomic DNA and cDNA interval upstream of the start codon (ATG) were investigated for the presence of STRs of ≥ 3-repeats (Additional file 1).

### Retrieval of gene IDs across species

Using the Enhanced REST API tools, a set of functions were developed to analyze genes and their transcripts information, including *func_get_ensemblID* and *func_get_TranscriptsID*. The genomic DNA, cDNA, and CDS sequences of genes and their respective transcripts were obtained using *func_get_GenomicSequence*, *func_get_cDNASequence*, and *func_get_CDSSequence* functions.

### Identification of STRs in the human TIS-flanking genomic and cDNA intervals

A general method of finding human-specific and non-specific STRs (≥ 3-repeats in all classes of STRs, except the mononucleotide repeats, in which STRs of ≥ 6-repeats were studied) for each individual gene was developed and applied as follows: the 120 bp genomic DNA and cDNA sequence upstream of the TISs of all annotated protein-coding gene transcripts was screened in human and 46 other species across vertebrates for the presence of STRs. A list of all STRs and their abundance was prepared for each gene in every species. The data obtained on the human STRs was compared to those of other species, and the term “human-specific” was applied to STRs that were not detected at ≥ 3-repeats in any other species. Exceptionally, in the mononucleotide category, the threshold of repeats for “human-specificity” was set at > 6-repeats. The relevant pseudo-code for the identification of repeated substrings was used for STR identification (Additional file 2).

Mann-Whitney *U* test was used to compare the distribution of human-specific vs. the overall (specific and non-specific) STR distribution in human.

### TIS homology threshold estimation

Weighted homology scoring was performed, in which the initial five amino acids (excluding the initial methionine) of the human protein-coding TISs, codons of which were flanked by STRs, were BLASTed (compared using BLAST) against all initial protein-coding five amino acids annotated for the orthologous genes in 46 species across vertebrates [(3,872,779 pair-wise TIS comparisons on the genomic DNA platform (Ensembl 91) and 1,442,200 pair-wise TIS comparisons on the cDNA platform (Ensembl 92)]. The above was aimed at comparing the number of events in which human-specific and non-specific STRs occurred with homologous and non-homologous TISs.

The following equation was developed for the weighted scoring of homology (Eq. 1), where *A* refers to the five amino acid sequence (excluding the initial methionine M), codons of which were flanked by a STR at the genomic DNA or cDNA sequence, *j* refers to the gene, and *B* refers to all the transcripts of the same gene that contain the STR in other species.

If M is the first methionine amino acid of two sequences, for all five successive positions represented by *i* in the equation, we defined five weight coefficients *W*_1_ to *W*_5_ based on the importance of the amino acid position, i.e., proximity to the methionine starting codon, observed in the *W* vector. The degree of homology between the two sequences *A* and *B* was calculated using function φ for all five positions with the operations $\sum \limits_{i=1}^{L=5}{W}_i\varphi \left({A}_{ji k},{B}_{ji{k}^{\prime }}\right)$. We repeated this operation for *k* transcripts, where *k* stands for the number of transcripts in human. *k*^′^ refers to all transcripts of the gene *j* in other species.

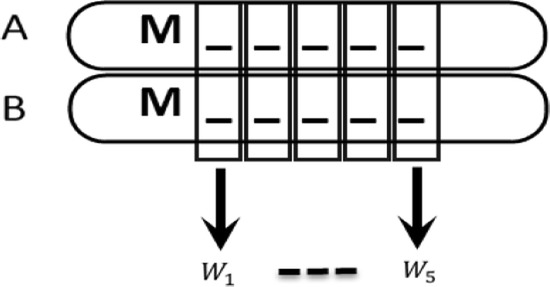

1 $$ {H}_k^j={\sum}_{i=1}^{L=5}{W}_i\varphi \left({A}_{ji k},{B}_{ji{k}^{\prime }}\right);\mathrm{for}\kern0.5em \mathrm{all}\kern0.5em k\kern0.5em \mathrm{and}\kern0.5em {k}^{\prime } $$

$$ \varphi \left(x,y\right)=\left\{\ \begin{array}{c}1;\mathrm{if}\ x\ne y\\ {}0;\mathrm{otherwise}\end{array}\right. $$

$$ W=\left\{25,25,25,12.5,12.5\ \Big\}\right. $$

Homology of the five amino acids, and therefore the TISs, was inferred based on the %similarity scoring. We validated the homology threshold by measuring the %similarity of 3000 random pairs of human proteins (the first five amino acids excluding the initial methionine), where similarity of ≥ 50% was virtually non-existent in that sample (6 in 3000, false positive rate = 0.001) (Additional file 3).

Finally, the two by two table and Fisher exact statistics were used to examine the link between STRs and TISs.

## Results

### Genome-scale distribution of human STRs in the 120 bp upstream sequence of TISs

#### Genomic DNA platform

**Fig. 1:**
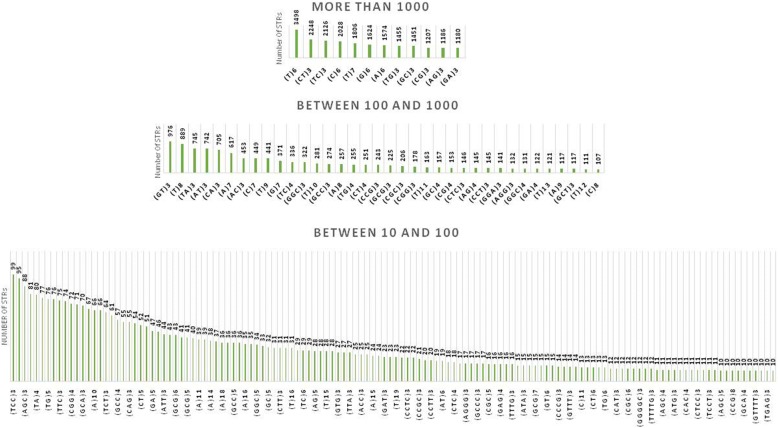
Genome-scale landscape of STRs in the 120 bp genomic DNA sequence upstream of human TISs. The abundance of STRs is sorted in the ascending order

Mono- and dinucleotide STRs dominated STRs of > 1000 counts, and the (T)6 mononucleotide repeat was the most abundant STR in this interval, succeeded by the (CT)3 and (TC)3 dinucleotide STRs (Fig. 1). Trinucleotide STRs were less abundant, observed at counts between 100 and 1000, and predominated by GC-rich composition such as (GGC)3, (GCC)3, (CCG)3, and (GCG)3. In the non-GC composition, (CTC)3 and (CCT)3 were the most common trinucleotide STRs. Tetra-, penta-, and hexanucleotide STRs were at lesser abundance than the above categories and observed at < 100 counts, where (CCTC)3 and (CCGC)3 were the most abundant tetranucleotide STRs. Only three pentanucleotide STR classes, (GGGGC)3, (TTTTG)3, and (GTTTT)3, were observed at counts > 10 in the screened interval.

#### cDNA platform

**Fig. 2:**
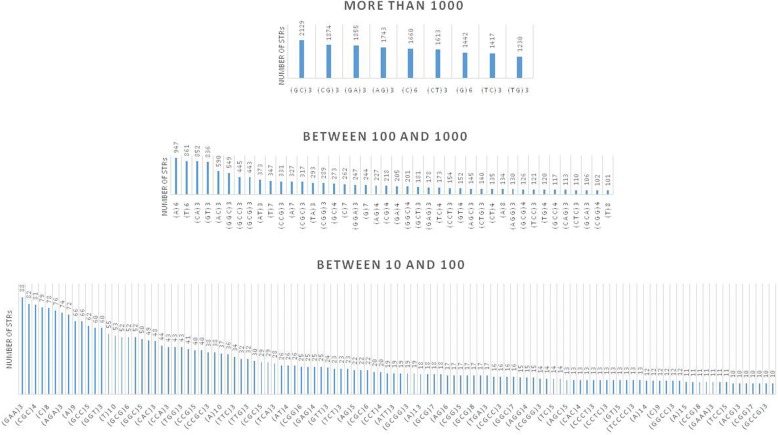
Genome-scale landscape of STRs in the 120 bp cDNA sequence upstream of human TISs. The abundance of STRs is sorted in the ascending order

The overall distribution of STRs in the 120 bp TIS-flanking cDNA sequences was significantly different in comparison to the genomic DNA STRs (Fig. 2). In comparison to the genomic DNA platform on which T(6) was the most abundant STR, GC-rich dinucleotide repeats were the most abundant on the cDNA platform. Numerous other instances at high, medium, and low abundance differentiated the genomic DNA vs. cDNA platforms (e.g., differential abundance of (T)8, (GT)3, (TA)3, and (CA)3 between the two platforms).

### Human-specific STR fingerprints on the TIS-flanking genomic DNA and cDNA platforms and differential distribution of these compartments in comparison to the overall STR distribution

#### Genomic DNA platform

The flanking sequence of 755,956 TISs was screened in human and 46 other species in order to identify human-specific STRs. One thousand eight hundred eighty-seven genes contained human-specific TIS-flanking STRs on the genomic DNA platform, which were of a wide range of nucleotide compositions of mono-, di-, tri-, tetra-, penta-, and hexanucleotide repeats, of which poly(A) and poly(T) STRs were the longest (the 1st percentile, based on STR length, is listed in Table 1, and the list of all genes is provided as Additional file 4).

**Table 1:** The 1st percentile of human protein-coding genes which contain human-specific STRs (length-wise) in their TIS-flanking genomic DNA sequence

Gene symbol	Gene Ensembl ID	Transcript ID	STR	GO term
*NVL*	ENSG00000143748	ENST00000436927	(T)22	ATP binding
*OR4K2*	ENSG00000165762	ENST00000641885	(T)20	Olfactory receptor activity
*MGRN1*	ENSG00000102858	ENST00000591895	(A)18	–
*SULT1A3*	ENSG00000261052	ENST00000338971		Sulfotransferase activity
		ENST00000395138		
*GDI2*	ENSG00000057608	ENST00000380127	(T)17	GTPase activator activity
		ENST00000609712		
*SULT1A4*	ENSG00000213648	ENST00000360423	(A)17	Sulfotransferase activity
*ZNF283*	ENSG00000167637	ENST00000618787	(T)17	Regulation of transcription
		ENST00000593268		
*ADAP2*	ENSG00000184060	ENST00000581548	(A)16	GTPase activator activity
*DDX20*	ENSG00000064703	ENST00000475700		Nucleic acid binding
*SGIP1*	ENSG00000118473	ENST00000435165	(A)16	Clathrin-dependent endocytosis
*LCA5L*	ENSG00000157578	ENST00000288350		Intraciliary transport
		ENST00000485895		
		ENST00000418018		
		ENST00000448288		
		ENST00000434281		
		ENST00000438404		
		ENST00000411566		
		ENST00000415863		
		ENST00000426783		
		ENST00000456017		
		ENST00000451131		
*LRRC36*	ENSG00000159708	ENST00000569499	(T)14	–
		ENST00000568804		
*OR7A10*	ENSG00000127515	ENST00000641129	(CT)14	G protein-coupled receptor activity
*POLR2F*	ENSG00000100142	ENST00000492213	(T)14	Transcription, DNA templated
*SNX19*	ENSG00000120451	ENST00000528555	(T)14	Integral component of membrane
		ENST00000530356		
*TEX11*	ENSG00000120498	ENST00000395889	(TTCC)14	Meiotic cell cycle
*ACAT1*	ENSG00000075239	ENST00000527942	(T)13	Transferring acyl groups
*CHRFAM7A*	ENSG00000166664	ENST00000299847	(T)13	Ion transmembrane transport
		ENST00000562729		
*GALK2*	ENSG00000156958	ENST00000560654	(TG)13	Phosphotransferase activity
		ENST00000396509		
		ENST00000558145		
		ENST00000544523		
		ENST00000560138		

As an extreme example, the TIS of the *NVL* gene was flanked by a human-specific (T)22 STR, which was the longest STR detected in a human protein-coding gene TIS-flanking sequence. The TIS of the gene, *SULT1A3*, was flanked by the longest poly(A) at (A)18. Short- and medium-length STRs were also detected in the human-specific compartment (Additional file 4).

**Fig. 3:**
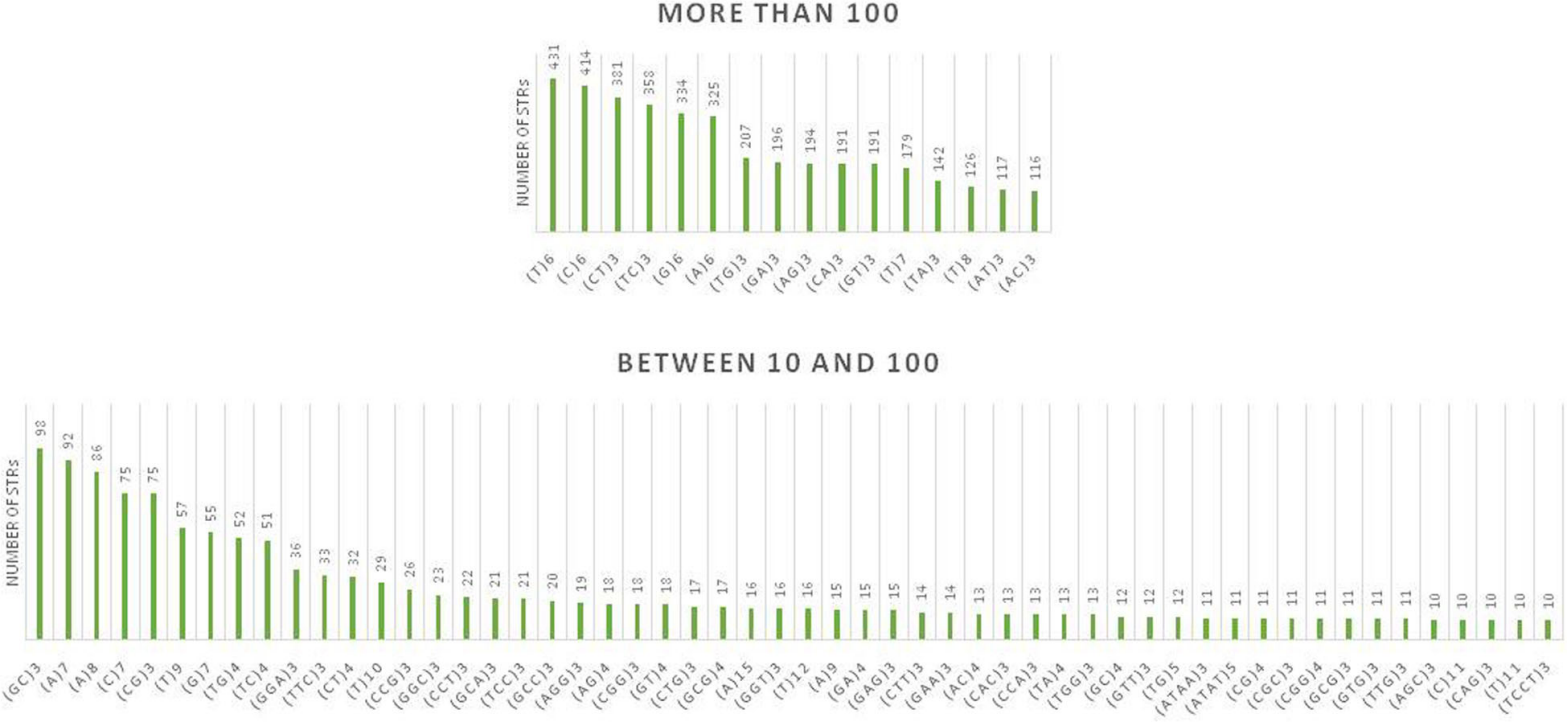
Distribution of the human-specific STRs in the TIS-flanking genomic DNA sequence. A significant skewing was observed between this compartment and the compartment containing the overall (human-specific and non-specific) STRs. The abundance of STRs is sorted in the ascending order

Significant skewing was observed in the distribution of human-specific STRs (Fig. 3) vs. the overall (i.e., human-specific and non-specific) STRs (Fig. 1) (Mann Whitney *U* test, *p* = 1 × 10^−5^). While the (GC)3 and (CG)3 dinucleotide STRs were enriched in the overall STR compartment, their abundance was significantly lower in the human-specific compartment. Instead, (CA)3 and (AC)3 were significantly more abundant in the human-specific compartment. Differences in the distribution of tri- and tetranucleotide STRs were also observed between the two compartments. While trinucleotide and tetranucleotide STRs of GC composition were more abundant in the overall compartment, non-GC STR compositions (e.g., GGA, TTC, GCA, and ATAA) were more abundant in the human-specific compartment.

#### cDNA platform

Two thousand six hundred genes contained human-specific STRs in their TIS cDNA flanking sequence (the 1st percentile based on length is represented in Table 2 and the complete list as Additional file 5). Similar to the genomic DNA platform, poly(A) and Poly(T) STRs were the longest STRs identified in the interval. The longest STR in this interval was (A)20 and belonged to *KCTD19*.

**Table 2:** The 1st percentile of human protein-coding genes which contain human-specific STRs (length-wise) in their TIS-flanking cDNA sequence

Gene symbol	Gene Ensembl ID	Transcript ID	STR	GO term
*KCTD19*	ENSG00000168676	ENST00000566295	(A)20	Protein homooligomerization
*ATP8B1*	ENSG00000081923	ENST00000585322	(A)17	Magnesium ion binding
*C1QTNF1*	ENSG00000173918	ENST00000583904		Collagen trimer
*SEC11A*	ENSG00000140612	ENST00000558196		Peptidase activity
*SHQ1*	ENSG00000144736	ENST00000463369		–
*SPRY1*	ENSG00000164056	ENST00000505319		Multicellular organism development
		ENST00000610581		
*DDX20*	ENSG00000064703	ENST00000475700	(A)16	ATP binding
*NAB1*	ENSG00000138386	ENST00000409641	(T)16	Negative regulation of transcription
*SGIP1*	ENSG00000118473	ENST00000435165	(A)16	–
*SOX6*	ENSG00000110693	ENST00000528252	(A)14	Multicellular organism development
*DPP6*	ENSG00000130226	ENST00000406326	(T)13	Proteolysis
		ENST00000377770		
*MLF1*	ENSG00000178053	ENST00000482628	(G)13	–
*SHC4*	ENSG00000185634	ENST00000558220	(T)13	Stem cell differentiation
*ITGB1BP2*	ENSG00000147166	ENST00000538820	(T)12	Calcium ion binding
*NELL2*	ENSG00000184613	ENST00000548531		Calcium ion binding
*GIPC1*	ENSG00000123159	ENST00000393028	(GCG)11	–
		ENST00000345425		
		ENST00000587210		
*HBS1L*	ENSG00000112339	ENST00000527578	(T)11	GTPase activity
*HOPX*	ENSG00000171476	ENST00000556376	(A)11	Cell differentiation
*OR7D2*	ENSG00000188000	ENST00000642043	(T)11	G protein-coupled receptor activity
*RNF145*	ENSG00000145860	ENST00000520638		Integral component of membrane
*TXNL4A*	ENSG00000141759	ENST00000592837		mRNA splicing, via spliceosome
*ABCF1*	ENSG00000204574	ENST00000468958	(A)10	ATP binding
*ARHGEF18*	ENSG00000104880	ENST00000359920	(T)10	Rho guanyl-nucleotide exchange factor activity
*ARL14*	ENSG00000179674	ENST00000320767	(A)10	GTP binding
*ASNS*	ENSG00000070669	ENST00000448127	(T)10	Asparagine biosynthetic process
*EIF2S1*	ENSG00000134001	ENST00000466499		Translation initiation factor activity

**Fig. 4:**
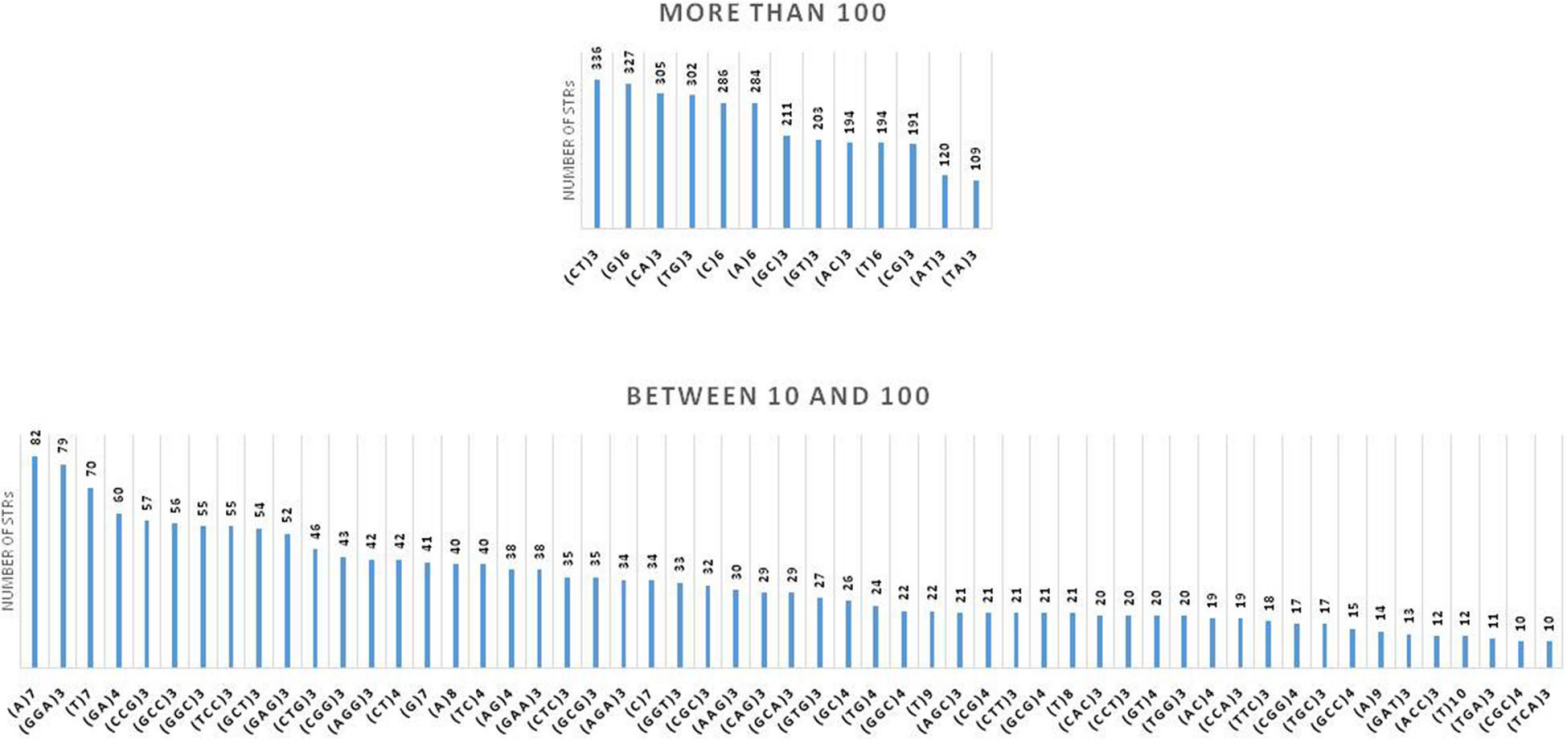
Distribution of the human-specific STR compartment in the TIS-flanking cDNA sequence. A significant skewing was observed between this compartment and the compartment containing the overall (human-specific and non-specific) STRs. The abundance of STRs is sorted in the ascending order

The distribution of human-specific STRs on the cDNA platform (Fig. 4) was unique to this platform and different from the overall STR distribution on the cDNA platform (Fig. 2) (Mann-Whitney *U* test *p* = 1 × 10^−5^). While the CG and CG dinucleotide STRs were more abundant in the overall distribution, CT, CA, and TG repeats were more abundant in the human-specific compartment. Various other differences were detected in other classes of STRs (e.g., more abundance of non-GC compositions in the trinucleotide STRs such as GGA, GCT, and CTG).

### STRs link to TIS selection on both genomic DNA and cDNA platforms

**Fig. 5:**
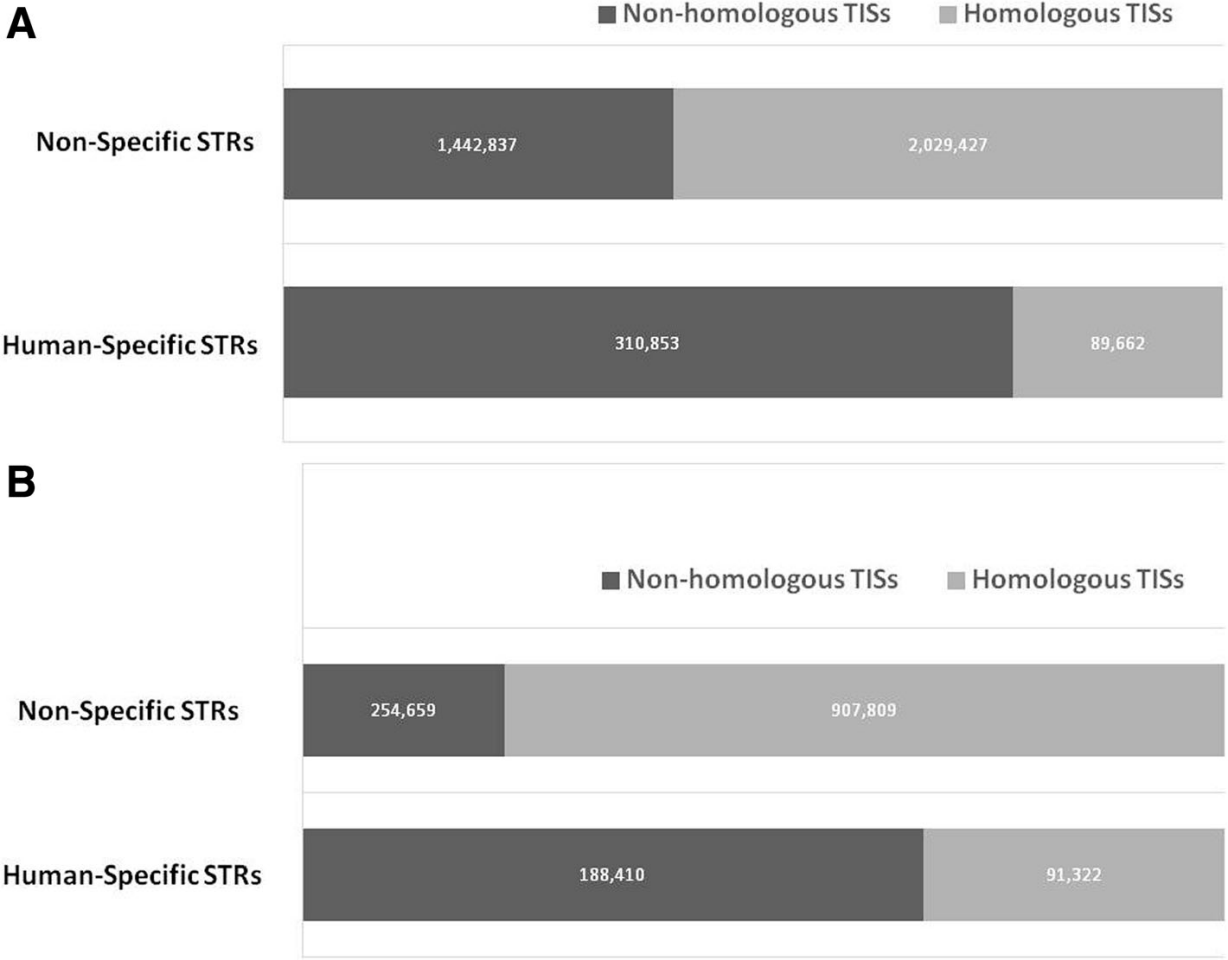
Evaluation of a link between STRs and TIS selection on the genomic DNA (**a**) and cDNA (**b**) platforms. The Fisher exact test statistic value < 0.00001. The number of times in which human-specific and non-specific STRs occurred with homologous and non-homologous TISs in other species is counted. %Similarity was checked for the first five amino acids (excluding the initiating methionine) of all annotated proteins for the orthologous genes in 46 species. TIS = translation initiation site. STR = short tandem repeat

We examined the hypothesis that there may be a link between STRs and TIS selection. The initial five amino acids (excluding the initial methionine) of the human protein sequences, codons of which were flanked by STRs at the genomic DNA and cDNA, were BLASTed (compared using BLAST) against the initial five amino acids of all the proteins annotated for the orthologous genes in 46 species across vertebrates in order to compare the number of events in which human-specific and non-specific STRs occurred with homologous and non-homologous TISs (≥ 50% and < 50% similarity of the five amino acids). Total of 5,314,979 pair-wise TIS comparisons were performed, and significant correlation was observed between STRs and TIS selection both on the genomic DNA (Fig. 5a) and cDNA platforms (Fig. 5b) (*p* < 0.00001), where there was excess occurrence of non-homologous TISs with human-specific STRs, and vice versa (i.e., excess occurrence of homologous TISs with non-specific STRs).

## Discussion

In this study, we characterized the genome-scale STR landscape of the immediate 120 bp upstream sequence of human TISs on the genomic DNA and cDNA platforms, cataloged the human-specific compartment of these STRs, and investigated a possible link between STRs and TIS selection. Our findings provide the first evidence of a link between STRs and TIS selection on both platforms. This link is primarily deduced based on the differential co-occurrence rate of human-specific STRs with non-homologous TISs and non-specific STRs with homologous TISs.

Sequence similarity searches can reliably identify “homologous” proteins or genes by detecting excess similarity [40]. The TIS homology threshold of ≥ 50% was validated based on 3000 random similarity scorings of the initial protein-coding five amino acids (excluding the initiating methionine) of human proteins, in which that threshold was non-existent in effect (false-positive rate = 0.001). This scoring methodology was consistently applied to the TISs linked to human-specific and non-specific STRs.

We also observed differential distribution of the human-specific STRs vs. the overall distribution of STRs on both genomic and cDNA platforms. Importantly, each platform had a unique pattern of STR distribution, indicating differential selection of STRs based on their location and evolutionary course. Genome-scale skewing of STRs, albeit at a lesser scale of STR classes, was reported by our group in a preliminary study of the gene core promoter interval [36].

It is imperative to envision that human-specific *cis* elements at the mRNA and DNA may result in the production of proteins that are specific to humans. The RNA structure influences recruitment of various RNA binding proteins and determines alternative TISs [41]. Indeed, the ribosomal machinery has the potential to scan and use several open reading frames (ORFs) at a particular mRNA species [42]. When located at the 5′ or 3′ UTR, STRs can modulate translation, the effect of which has biological and pathological implications [13, 43, 44]. The disorders linked to the 5′ UTR STRs encompass a number of human-specific neurological disorders.

On the genomic DNA platform, proximity to the splice sites may increase the biological/pathological implication of repeats [45]. Similar to the cDNA STRs, we observed significant enrichment of non-homologous TISs co-occurring with human-specific genomic STRs, which were substantially near the exons (within 120 bp upstream of the TISs).

Gene Ontology (GO) search yielded a variety of terms across the identified genes, including neuron cell fate specification, multicellular organism development, translation initiation factor activity, and cell differentiation (https://www.ebi.ac.uk/QuickGO/), examples of which are presented in Tables 1 and 2.

**Fig. 6:**
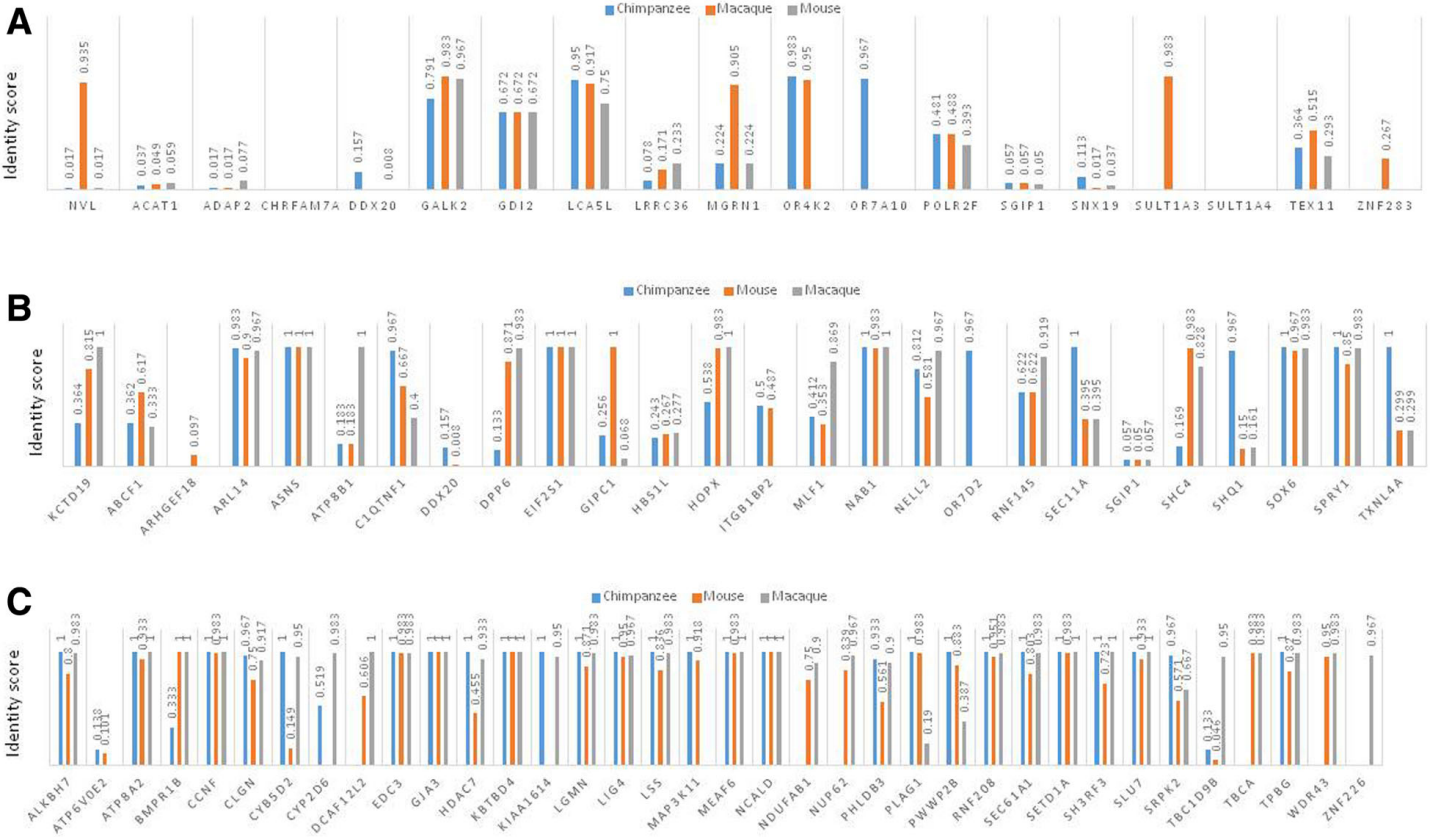
Sample protein conservation analysis encoded by the genes listed in Table 1 (**a**), Table 2 (**b**), and several randomly selected proteins, whose codons were flanked by non-specific STRs (**c**), between human and three other species. Maximum identity scores were annotated for each gene in every species. Identity scores were considerably higher for the proteins in the non-specific STR compartment

EMBOSS Needle (https://www.ebi.ac.uk/Tools/psa/emboss_needle) pair-wise comparison was performed between human and three other species (chimpanzee, macaque, and mouse), of the proteins encoded by the transcripts in Table 1 (Fig. 6a), Table 2 (Fig. 6b), and several randomly selected proteins, codons of which were flanked by non-specific STRs (Fig. 6c). Identity scores were considerably lower across the three species for the genes in Tables 1 and 2, compared to the identity scores for the genes in the non-specific STR compartment.

A number of the identified genes such as *ACAT1* (Table 1) and *SGIP1* (Table 2) confer risk for diseases or endophenotypes that are predominantly specific to the human species, such as complex psychiatric disorders [46, 47]. *SULT1A4* (Table 1) plays a critical role in neurotransmitter metabolism in the human brain and is also linked to neurodegeneration [48]. *APOA2* (Additional file 4) along with several other lipoproteins is linked to cognitive health [49]. In another remarkable example, *TBR1* (Additional file 5) is involved in *FOXP2* gene expression, which has pivotal role in speech and language in human [50]. *SRGAP2* (Additional file 5) family proteins may have increased the density of dendritic spines and promoted neoteny of the human brain during crucial periods of human evolution [51].

GO terms, protein conservation comparisons, and phenotypes stated above are only a few examples of the identified genes, in which human-specific STRs and the linked TISs may contribute to human evolution and disease. Future studies are warranted to examine the implication of the identified STRs and genes at the inter- and intra-species levels.

## Conclusion

We characterized the landscape of STRs at the immediate upstream genomic DNA and cDNA sequences flanking the human protein-coding gene TISs and found differential distribution of the human-specific STRs in comparison to the overall distribution of STRs on both platforms. Further, we propose a link between STRs and TIS selection, based on the differential co-occurrence rate of human-specific STRs with non-homologous TISs and non-specific STRs with homologous TISs. The data presented here have implications at the inter- and intraspecies levels, which warrant further functional and evolutionary studies.

## Additional files

Additional file 1: Workflow of STR identification in the TIS-flanking genomic DNA and cDNA upstream sequences. (JPG 37 kb)

Additional file 2: Pseudo-codes used for STR identification. (JPG 43 kb)

Additional file 3: Homology threshold validation. Three thousand random pair-wise similarity checks were performed on the five initial amino acids (excluding methionine) of human protein sequences. A similarity threshold of ≥ 50% was considered “homology.” (JPG 24 kb)

Additional file 4: List of all human protein-coding genes which contain human-specific STRs in their TIS-flanking genomic DNA sequence. (DOCX 282 kb)

Additional file 5: List of all human protein-coding genes which contain human-specific STRs in their TIS-flanking cDNA sequence. (DOCX 393 kb)

## Abbreviations

cDNA: Complementary DNA
CDS: Coding DNA sequence
GO: Gene Ontology
mRNA: Messenger RNA
STR: Short tandem repeat
TIS: Translation initiation site
TSS: Transcription start site
