# Supplementary figures and images for: Link between short tandem repeats and translation initiation site selection
Source: Hum Genomics. 2018 Oct 29;12:47. doi: 10.1186/s40246-018-0181-3 (PMC6206671; doi:10.1186/s40246-018-0181-3)

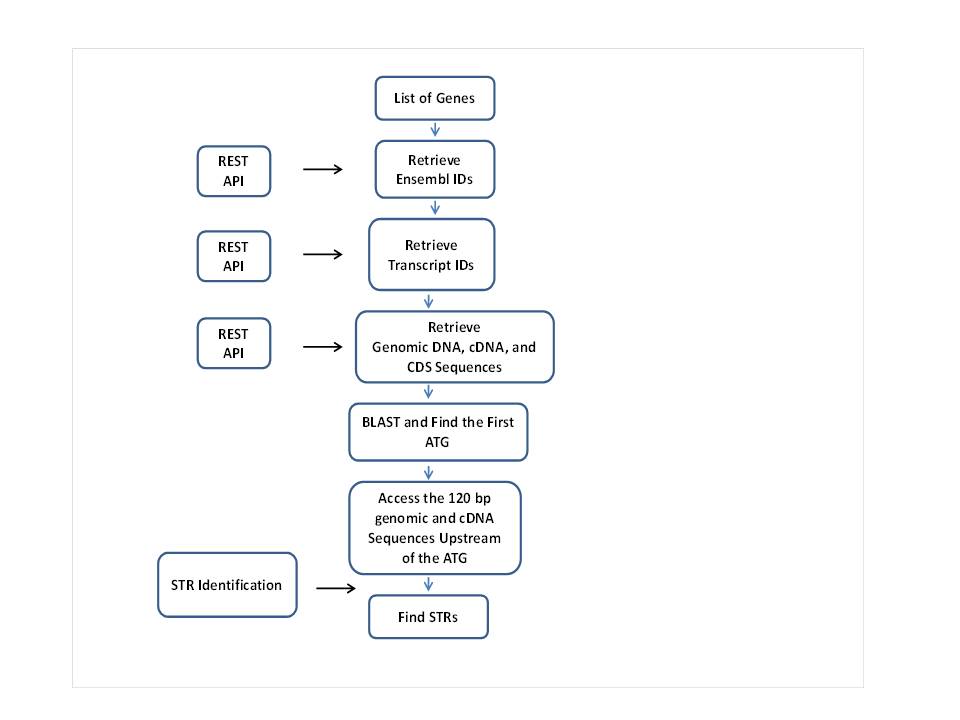

Supplement: Supplementary file 1 — Workflow of STR identification in the TIS-flanking genomic DNA and cDNA upstream sequences. (JPG 37 kb) [file 40246_2018_181_MOESM1_ESM.jpg]

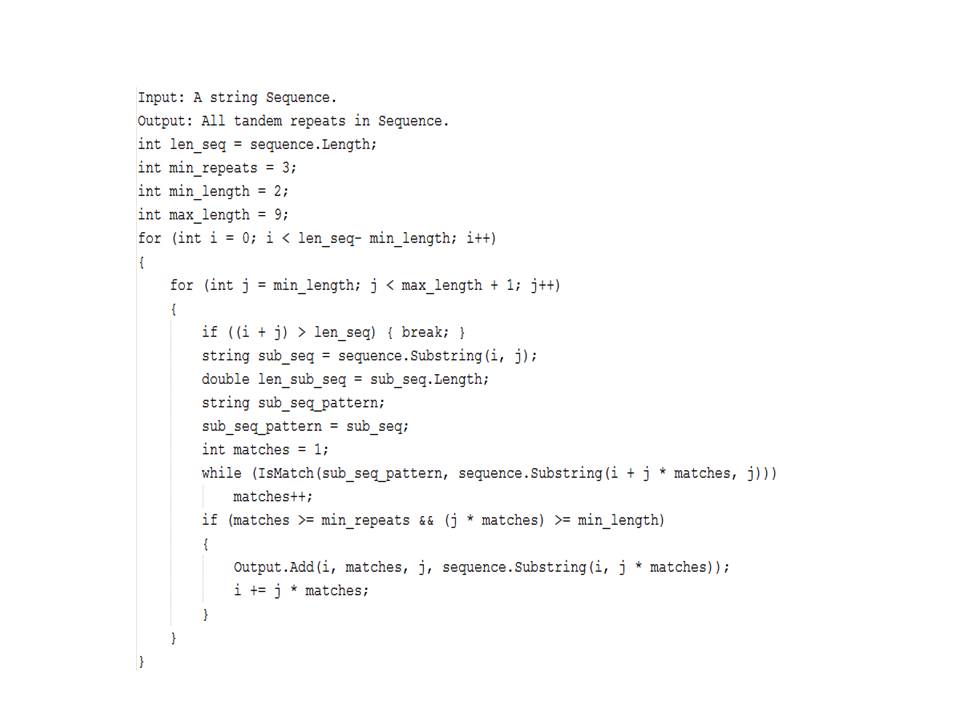

Supplement: Supplementary file 2 — Pseudo-codes used for STR identification. (JPG 43 kb) [file 40246_2018_181_MOESM2_ESM.jpg]

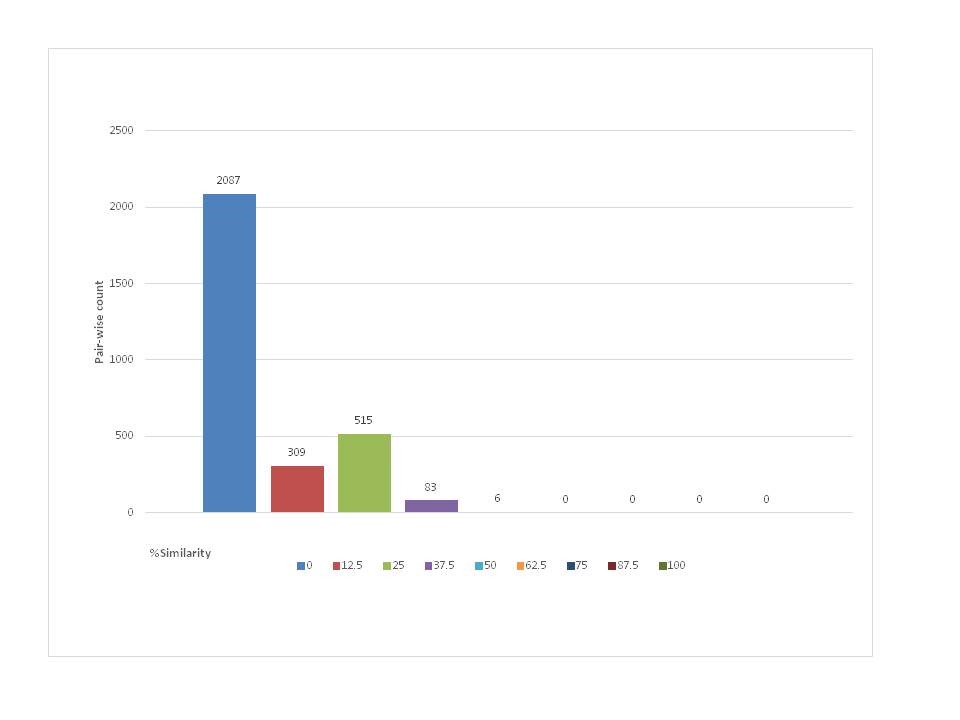

Supplement: Supplementary file 3 — Homology threshold validation. Three thousand random pair-wise similarity checks were performed on the five initial amino acids (excluding methionine) of human protein sequences. A similarity threshold of ≥ 50% was considered “homology.” (JPG 24 kb) [file 40246_2018_181_MOESM3_ESM.jpg]
