# Supplementary material for: Link between short tandem repeats and translation initiation site selection
Source: Hum Genomics. 2018 Oct 29;12:47. doi: 10.1186/s40246-018-0181-3 (PMC6206671; doi:10.1186/s40246-018-0181-3)
Supplement: Supplementary file 4 — List of all human protein-coding genes which contain human-specific STRs in their TIS-flanking genomic DNA sequence. (DOCX 282 kb) [file 40246_2018_181_MOESM4_ESM.docx]

| Gene Symbol | Gene Ensembl ID | Transcript ID | STR |
| --- | --- | --- | --- |
| NVL | ENSG00000143748 | ENST00000436927 | (T)22 |
| OR4K2 | ENSG00000165762 | ENST00000641885 | (T)20 |
| MGRN1 | ENSG00000102858 | ENST00000591895 | (A)18 |
| SULT1A3 | ENSG00000261052 | ENST00000338971 |  |
|  |  | ENST00000395138 |  |
| GDI2 | ENSG00000057608 | ENST00000380127 | (T)17 |
|  |  | ENST00000609712 |  |
| SULT1A4 | ENSG00000213648 | ENST00000360423 | (A)17 |
| ZNF283 | ENSG00000167637 | ENST00000618787 | (T)17 |
|  |  | ENST00000593268 |  |
| ADAP2 | ENSG00000184060 | ENST00000581548 | (A)16 |
| DDX20 | ENSG00000064703 | ENST00000475700 |  |
| SGIP1 | ENSG00000118473 | ENST00000435165 | (A)16 |
| LCA5L | ENSG00000157578 | ENST00000288350 |  |
|  |  | ENST00000358268 |  |
|  |  | ENST00000485895 |  |
|  |  | ENST00000418018 |  |
|  |  | ENST00000448288 |  |
|  |  | ENST00000434281 |  |
|  |  | ENST00000438404 |  |
|  |  | ENST00000411566 |  |
|  |  | ENST00000415863 |  |
|  |  | ENST00000426783 |  |
|  |  | ENST00000456017 |  |
|  |  | ENST00000451131 |  |
| LRRC36 | ENSG00000159708 | ENST00000569499 | (T)14 |
|  |  | ENST00000568804 |  |
| OR7A10 | ENSG00000127515 | ENST00000641129 | (CT)14 |
| POLR2F | ENSG00000100142 | ENST00000492213 | (T)14 |
| SNX19 | ENSG00000120451 | ENST00000528555 |  |
|  |  | ENST00000530356 |  |
| TEX11 | ENSG00000120498 | ENST00000395889 | (TTCC)14 |
| ACAT1 | ENSG00000075239 | ENST00000527942 | (T)13 |
| CHRFAM7A | ENSG00000166664 | ENST00000299847 |  |
|  |  | ENST00000562729 |  |
| GALK2 | ENSG00000156958 | ENST00000560654 | (TG)13 |
|  |  | ENST00000396509 |  |
|  |  | ENST00000558145 |  |
|  |  | ENST00000544523 |  |
|  |  | ENST00000560138 |  |
|  |  | ENST00000559454 |  |
| GLOD4 | ENSG00000167699 | ENST00000536578 | (A)13 |
| MYO5C | ENSG00000128833 | ENST00000558479 |  |
| PYCR1 | ENSG00000183010 | ENST00000582198 |  |
|  |  | ENST00000579366 |  |
| RFX2 | ENSG00000087903 | ENST00000586806 | (T)13 |
| SLC6A9 | ENSG00000196517 | ENST00000475075 | (A)13 |
| CHMP2B | ENSG00000083937 | ENST00000472024 | (T)12 |
| CNGA1 | ENSG00000198515 | ENST00000402813 |  |
| EFHB | ENSG00000163576 | ENST00000344838 |  |
| KCNN4 | ENSG00000104783 | ENST00000615047 | (A)12 |
| MRPS36 | ENSG00000134056 | ENST00000512880 | (T)12 |
|  |  | ENST00000602380 |  |
| NPTN | ENSG00000156642 | ENST00000565282 |  |
| RFC5 | ENSG00000111445 | ENST00000484086 |  |
| SNX1 | ENSG00000028528 | ENST00000560829 | (A)12 |
| TTC14 | ENSG00000163728 | ENST00000492617 | (T)12 |
|  |  | ENST00000495660 |  |
| DAGLB | ENSG00000164535 | ENST00000436575 |  |
| PFN2 | ENSG00000070087 | ENST00000481767 |  |
|  |  | ENST00000494827 |  |
|  |  | ENST00000497148 |  |
|  |  | ENST00000475518 |  |
|  |  | ENST00000481275 |  |
|  |  | ENST00000498307 |  |
|  |  | ENST00000489155 |  |
| TNFAIP8 | ENSG00000145779 | ENST00000388882 |  |
| APOA2 | ENSG00000158874 | ENST00000468465 | (GT)11 |
|  |  | ENST00000463812 |  |
| CHN1 | ENSG00000128656 | ENST00000409089 | (T)11 |
|  |  | ENST00000444394 |  |
| EXTL3 | ENSG00000012232 | ENST00000523149 |  |
| HS3ST4 | ENSG00000182601 | ENST00000331351 | (GCG)11 |
| KITLG | ENSG00000049130 | ENST00000552044 | (T)11 |
| OR52N4 | ENSG00000181074 | ENST00000641350 |  |
| PDXDC1 | ENSG00000179889 | ENST00000563522 |  |
|  |  | ENST00000566426 |  |
|  |  | ENST00000567306 |  |
| TSNAXIP1 | ENSG00000102904 | ENST00000388833 | (A)11 |
| ZNF480 | ENSG00000198464 | ENST00000335090 | (T)11 |
| ZNF91 | ENSG00000167232 | ENST00000595533 |  |
| ABCF1 | ENSG00000204574 | ENST00000468958 | (A)10 |
| ACAP2 | ENSG00000114331 | ENST00000439666 | (T)10 |
| ARHGEF18 | ENSG00000104880 | ENST00000359920 |  |
| ARL14 | ENSG00000179674 | ENST00000320767 | (A)10 |
| COBLL1 | ENSG00000082438 | ENST00000439313 | (T)10 |
| DCTN4 | ENSG00000132912 | ENST00000424236 |  |
|  |  | ENST00000518015 |  |
|  |  | ENST00000521533 |  |
| LRRC37A3 | ENSG00000176809 | ENST00000400877 |  |
|  |  |  |  |
| NPM1 | ENSG00000181163 | ENST00000521672 |  |
| OR7A17 | ENSG00000185385 | ENST00000642123 | (AATA)10 |
|  |  | ENST00000641113 |  |
| PDE4DIP | ENSG00000178104 | ENST00000479408 | (A)10 |
| POLR3E | ENSG00000058600 | ENST00000565358 | (T)10 |
| PSMA1 | ENSG00000129084 | ENST00000530457 |  |
|  |  | ENST00000533068 |  |
| RAPGEF5 | ENSG00000136237 | ENST00000458533 |  |
| RAPGEF6 | ENSG00000158987 | ENST00000513227 |  |
| SRPK1 | ENSG00000096063 | ENST00000512445 |  |
| TLR2 | ENSG00000137462 | ENST00000646900 | (GT)10 |
|  |  | ENST00000646219 |  |
|  |  | ENST00000642580 |  |
|  |  | ENST00000644308 |  |
|  |  | ENST00000643501 |  |
|  |  | ENST00000642700 |  |
|  |  | ENST00000260010 |  |
| TMBIM4 | ENSG00000155957 | ENST00000544599 | (T)10 |
| UGT2B7 | ENSG00000171234 | ENST00000502942 |  |
| FZD8 | ENSG00000177283 | ENST00000374694 | (C)10 |
| RAPGEF2 | ENSG00000109756 | ENST00000264431 | (T)10 |
| UHRF1BP1L | ENSG00000111647 | ENST00000545232 | (C)10 |
|  |  | ENST00000548045 |  |
|  |  | ENST00000551973 |  |
|  |  | ENST00000550544 |  |
|  |  | ENST00000551980 |  |
| AKR1E2 | ENSG00000165568 | ENST00000533295 | (T)9 |
| AUTS2 | ENSG00000158321 | ENST00000644939 |  |
|  |  | ENST00000406775 |  |
|  |  | ENST00000342771 |  |
| BBS1 | ENSG00000174483 | ENST00000526815 |  |
| CBWD5 | ENSG00000147996 | ENST00000377392 |  |
| CMAS | ENSG00000111726 | ENST00000538498 |  |
| EFHC1 | ENSG00000096093 | ENST00000636702 |  |
| EIF4A1 | ENSG00000161960 | ENST00000578754 |  |
| ENTPD1 | ENSG00000138185 | ENST00000543964 |  |
| FOXE3 | ENSG00000186790 | ENST00000335071 | (G)9 |
| GOSR2 | ENSG00000108433 | ENST00000638892 | (T)9 |
| IL31RA | ENSG00000164509 | ENST00000396836 | (A)9 |
| IPO8 | ENSG00000133704 | ENST00000543446 |  |
| KIAA0391 | ENSG00000100890 | ENST00000604948 | (T)9 |
| LCE4A | ENSG00000187170 | ENST00000368777 | (A)9 |
| MRPL10 | ENSG00000159111 | ENST00000290208 | (CT)9 |
|  |  |  | (T)9 |
|  |  | ENST00000414011 | (CT)9 |
|  |  |  | (T)9 |
| MRPS28 | ENSG00000147586 | ENST00000521434 |  |
|  |  | ENST00000520946 |  |
| OR7A17 | ENSG00000185385 | ENST00000642123 | (A)9 |
|  |  | ENST00000641113 |  |
| POMT1 | ENSG00000130714 | ENST00000341012 | (T)9 |
|  |  | ENST00000448212 |  |
| RGPD5 | ENSG00000015568 | ENST00000432606 |  |
| RGPD8 | ENSG00000169629 | ENST00000409750 |  |
| SCAPER | ENSG00000140386 | ENST00000568382 |  |
|  |  | ENST00000564177 |  |
| SLC22A18AS | ENSG00000254827 | ENST00000625099 |  |
| SRRM1 | ENSG00000133226 | ENST00000593639 |  |
|  |  | ENST00000596378 |  |
| SSH1 | ENSG00000084112 | ENST00000546697 |  |
| TTC7A | ENSG00000068724 | ENST00000409245 |  |
| ZNF140 | ENSG00000196387 | ENST00000544426 |  |
| ZNF182 | ENSG00000147118 | ENST00000396965 | (A)9 |
| ZNF254 | ENSG00000213096 | ENST00000616028 | (T)9 |
| ZNF766 | ENSG00000196214 | ENST00000601711 | (A)9 |
|  |  | ENST00000593612 |  |
| ZXDA | ENSG00000198205 | ENST00000358697 | (T)9 |
| TM9SF3 | ENSG00000077147 | ENST00000443638 |  |
| AASDHPPT | ENSG00000149313 | ENST00000533423 | (A)8 |
|  |  | ENST00000524411 |  |
| AFF1 | ENSG00000172493 | ENST00000511442 | (T)8 |
|  |  |  |  |
| ATOH1 | ENSG00000172238 | ENST00000306011 |  |
| B4GALT6 | ENSG00000118276 | ENST00000579372 | (A)8 |
| C11orf65 | ENSG00000166323 | ENST00000525729 |  |
|  |  | ENST00000615746 |  |
|  |  | ENST00000393084 |  |
| CDH4 | ENSG00000179242 | ENST00000614565 | (CGG)8 |
| CTNNA2 | ENSG00000066032 | ENST00000361291 | (A)8 |
|  |  | ENST00000540488 |  |
| DGKI | ENSG00000157680 | ENST00000453654 | (GT)8 |
| FRG1 | ENSG00000109536 | ENST00000531991 | (A)8 |
| FRMD6 | ENSG00000139926 | ENST00000555936 | (T)8 |
|  |  |  |  |
|  |  | ENST00000554167 |  |
|  |  |  |  |
| GSTA3 | ENSG00000174156 | ENST00000370968 |  |
| HEXB | ENSG00000049860 | ENST00000511181 | (A)8 |
| HNRNPD | ENSG00000138668 | ENST00000515432 | (T)8 |
| HSD11B1L | ENSG00000167733 | ENST00000342970 | (C)8 |
| LRRTM4 | ENSG00000176204 | ENST00000456154 | (T)8 |
| MLXIP | ENSG00000175727 | ENST00000366272 |  |
| MYL10 | ENSG00000106436 | ENST00000223167 | (A)8 |
| OR2J3 | ENSG00000204701 | ENST00000641151 | (T)8 |
|  |  | ENST00000641960 |  |
| OR4D1 | ENSG00000141194 | ENST00000641449 |  |
| OR51G2 | ENSG00000176893 | ENST00000641926 |  |
| OR52N1 | ENSG00000181001 | ENST00000641645 |  |
| OR5K1 | ENSG00000232382 | ENST00000642057 |  |
| OR6C75 | ENSG00000187857 | ENST00000641576 | (A)8 |
| PEG10 | ENSG00000242265 | ENST00000615790 |  |
|  |  | ENST00000612941 |  |
| PITPNA | ENSG00000174238 | ENST00000576761 | (T)8 |
| PREPL | ENSG00000138078 | ENST00000541738 |  |
|  |  | ENST00000409411 |  |
|  |  | ENST00000409957 |  |
| PRKCZ | ENSG00000067606 | ENST00000486681 | (A)8 |
|  |  | ENST00000497183 |  |
| PROS1 | ENSG00000184500 | ENST00000407433 |  |
|  |  | ENST00000472684 |  |
| RFC5 | ENSG00000111445 | ENST00000484086 |  |
| RFPL4A | ENSG00000223638 | ENST00000434937 | (T)8 |
| RIC8B | ENSG00000111785 | ENST00000549643 |  |
| RNF128 | ENSG00000133135 | ENST00000418562 |  |
| RPL36A | ENSG00000241343 | ENST00000471855 |  |
| SCAPER | ENSG00000140386 | ENST00000568382 |  |
|  |  | ENST00000564177 |  |
| SCFD1 | ENSG00000092108 | ENST00000544052 |  |
| SLC7A2 | ENSG00000003989 | ENST00000470360 |  |
| SNRPC | ENSG00000124562 | ENST00000374017 | (A)8 |
| SNX31 | ENSG00000174226 | ENST00000520661 | (GTT)8 |
| STXBP1 | ENSG00000136854 | ENST00000637521 | (CT)8 |
|  |  | ENST00000625363 |  |
|  |  | ENST00000626539 |  |
|  |  | ENST00000637173 |  |
|  |  | ENST00000630492 |  |
|  |  | ENST00000626333 |  |
| SWAP70 | ENSG00000133789 | ENST00000534662 | (T)8 |
| TLR10 | ENSG00000174123 | ENST00000308973 |  |
|  |  | ENST00000613579 |  |
|  |  | ENST00000361424 |  |
|  |  | ENST00000622002 |  |
|  |  | ENST00000506111 |  |
|  |  | ENST00000508334 |  |
|  |  | ENST00000502321 |  |
| TMEM105 | ENSG00000185332 | ENST00000332900 | (G)8 |
|  |  | ENST00000574093 |  |
| TRAPPC4 | ENSG00000196655 | ENST00000533632 | (GGA)8 |
| TTC27 | ENSG00000018699 | ENST00000448773 | (T)8 |
| UNC13C | ENSG00000137766 | ENST00000539562 |  |
| USP9Y | ENSG00000114374 | ENST00000338981 | (A)8 |
| WDR33 | ENSG00000136709 | ENST00000436787 | (TATT)8 |
| XPO6 | ENSG00000169180 | ENST00000565698 | (T)8 |
|  |  | ENST00000570033 |  |
|  |  | ENST00000566073 |  |
| YEATS4 | ENSG00000127337 | ENST00000549685 |  |
| YIPF2 | ENSG00000130733 | ENST00000586575 | (C)8 |
|  |  | ENST00000587943 |  |
| ZNF781 | ENSG00000196381 | ENST00000358582 | (A)8 |
|  |  | ENST00000590008 |  |
| ZNF804A | ENSG00000170396 | ENST00000613975 | (T)8 |
| ATOH1 | ENSG00000172238 | ENST00000306011 | (A)4 |
| ATP6V1D | ENSG00000100554 | ENST00000553687 | (T)8 |
| CHMP2B | ENSG00000083937 | ENST00000472024 |  |
| CREB5 | ENSG00000146592 | ENST00000396299 |  |
|  |  | ENST00000409603 |  |
| CXCR4 | ENSG00000121966 | ENST00000409817 |  |
| PIGX | ENSG00000163964 | ENST00000426755 | (A)8 |
|  |  | ENST00000421265 |  |
|  |  | ENST00000451319 |  |
| RNF208 | ENSG00000212864 | ENST00000392827 | (C)8 |
| SAMD1 | ENSG00000141858 | ENST00000533683 | (G)8 |
| ADAM32 | ENSG00000197140 | ENST00000521741 | (T)7 |
| AK3 | ENSG00000147853 | ENST00000611749 |  |
|  |  | ENST00000359883 |  |
| APOC2 | ENSG00000234906 | ENST00000252490 | (C)7 |
|  |  | ENST00000592257 |  |
|  |  | ENST00000591597 |  |
|  |  | ENST00000590360 |  |
| ARID1A | ENSG00000117713 | ENST00000430799 | (A)7 |
|  |  | ENST00000637465 |  |
|  |  | ENST00000524572 |  |
|  |  | ENST00000374152 |  |
|  |  | ENST00000615191 |  |
| BTBD9 | ENSG00000183826 | ENST00000419706 |  |
| C3orf18 | ENSG00000088543 | ENST00000422619 |  |
| C9orf153 | ENSG00000187753 | ENST00000339137 | (T)7 |
|  |  | ENST00000376001 |  |
| CACNA1D | ENSG00000157388 | ENST00000637424 | (A)7 |
| CACNA2D4 | ENSG00000151062 | ENST00000538450 | (T)7 |
| CASP4 | ENSG00000196954 | ENST00000393150 | (A)7 |
| CEP120 | ENSG00000168944 | ENST00000306481 |  |
|  |  |  |  |
|  |  | ENST00000508442 |  |
|  |  |  |  |
| CEP164 | ENSG00000110274 | ENST00000533153 | (T)7 |
|  |  | ENST00000525416 |  |
| CTDP1 | ENSG00000060069 | ENST00000590635 |  |
|  |  | ENST00000299543 |  |
| CYTH2 | ENSG00000105443 | ENST00000620214 | (A)7 |
| DCTN5 | ENSG00000166847 | ENST00000566298 | (T)7 |
| DEPDC1 | ENSG00000024526 | ENST00000525124 | (A)7 |
| DNAH10 | ENSG00000197653 | ENST00000614082 | (T)7 |
| DPY19L2 | ENSG00000177990 | ENST00000538147 |  |
| EPHB6 | ENSG00000106123 | ENST00000411471 | (C)7 |
| ERBB3 | ENSG00000065361 | ENST00000553131 | (G)7 |
| ETFA | ENSG00000140374 | ENST00000560726 | (A)7 |
| FAM19A2 | ENSG00000198673 | ENST00000550003 |  |
| FBXL7 | ENSG00000183580 | ENST00000504595 | (G)7 |
| FLG | ENSG00000143631 | ENST00000368799 | (A)7 |
| FOXR2 | ENSG00000189299 | ENST00000339140 |  |
| FXR1 | ENSG00000114416 | ENST00000484042 | (T)7 |
| GET4 | ENSG00000239857 | ENST00000441491 |  |
| GIMAP7 | ENSG00000179144 | ENST00000313543 |  |
| GYPB | ENSG00000250361 | ENST00000429670 |  |
|  |  | ENST00000502664 |  |
| GYPE | ENSG00000197465 | ENST00000358615 |  |
| HIST1H2BN | ENSG00000233822 | ENST00000612898 |  |
|  |  | ENST00000606613 |  |
| HNRNPU | ENSG00000153187 | ENST00000440865 |  |
| HS3ST4 | ENSG00000182601 | ENST00000331351 | (GCC)7 |
| IFI27 | ENSG00000165949 | ENST00000618863 | (G)7 |
| IFT122 | ENSG00000163913 | ENST00000507221 | (T)7 |
| ITM2C | ENSG00000135916 | ENST00000541852 | (G)7 |
|  |  | ENST00000543957 |  |
|  |  | ENST00000409704 |  |
|  |  | ENST00000418408 |  |
| KCNIP4 | ENSG00000185774 | ENST00000382148 | (ATT)7 |
| LAMP3 | ENSG00000078081 | ENST00000466939 | (T)7 |
|  |  | ENST00000470251 |  |
| LCMT2 | ENSG00000168806 | ENST00000305641 |  |
| LRCH1 | ENSG00000136141 | ENST00000463929 |  |
|  |  | ENST00000478412 |  |
| LRRC36 | ENSG00000159708 | ENST00000563189 | (A)7 |
|  |  | ENST00000435835 |  |
| MAFB | ENSG00000204103 | ENST00000373313 | (C)7 |
| MED20 | ENSG00000124641 | ENST00000434077 | (T)7 |
| MED26 | ENSG00000105085 | ENST00000598608 | (G)7 |
| MFSD1 | ENSG00000118855 | ENST00000465739 | (A)7 |
| MKLN1 | ENSG00000128585 | ENST00000421797 | (T)7 |
|  |  | ENST00000416992 |  |
|  |  | ENST00000429546 |  |
|  |  | ENST00000446815 |  |
| MLF1 | ENSG00000178053 | ENST00000491767 | (A)7 |
| MRPS27 | ENSG00000113048 | ENST00000457646 |  |
|  |  | ENST00000508863 |  |
|  |  | ENST00000515404 |  |
| MSL1 | ENSG00000188895 | ENST00000582920 |  |
| NCBP2L | ENSG00000170935 | ENST00000639756 | (T)7 |
| NLGN4Y | ENSG00000165246 | ENST00000382872 |  |
| NLRP4 | ENSG00000160505 | ENST00000587891 | (A)7 |
| NPM1 | ENSG00000181163 | ENST00000521672 | (G)7 |
| NVL | ENSG00000143748 | ENST00000469968 | (T)7 |
|  |  | ENST00000391875 |  |
| OR2AP1 | ENSG00000179615 | ENST00000641114 |  |
| OR2B3 | ENSG00000204703 | ENST00000377173 |  |
| OR2F2 | ENSG00000221910 | ENST00000408955 |  |
| OR2T8 | ENSG00000177462 | ENST00000641945 |  |
| OR4C11 | ENSG00000172188 | ENST00000641580 |  |
| OR4K14 | ENSG00000169484 | ENST00000641793 |  |
| OR4S2 | ENSG00000174982 | ENST00000641692 |  |
| OR51A4 | ENSG00000205497 | ENST00000641898 |  |
| OR5B12 | ENSG00000172362 | ENST00000641921 | (A)7 |
| OR5B2 | ENSG00000172365 | ENST00000641342 | (T)7 |
| OR5K2 | ENSG00000231861 | ENST00000427338 |  |
| OR6C75 | ENSG00000187857 | ENST00000641576 | (A)7 |
| OR6K3 | ENSG00000203757 | ENST00000368145 | (T)7 |
| OR7A5 | ENSG00000188269 | ENST00000322301 | (CT)7 |
| PANK2 | ENSG00000125779 | ENST00000495692 | (T)7 |
| PARVB | ENSG00000188677 | ENST00000444029 |  |
| PDE1C | ENSG00000154678 | ENST00000396193 | (G)7 |
| PDE6G | ENSG00000185527 | ENST00000573076 | (C)7 |
| PDHX | ENSG00000110435 | ENST00000533550 | (T)7 |
| PELP1 | ENSG00000141456 | ENST00000436683 |  |
|  |  | ENST00000570387 |  |
| PHF3 | ENSG00000118482 | ENST00000506783 | (A)7 |
|  |  | ENST00000515594 |  |
| PLAC1 | ENSG00000170965 | ENST00000359237 | (T)7 |
| PLBD1 | ENSG00000121316 | ENST00000540572 | (A)7 |
| PRODH | ENSG00000100033 | ENST00000610940 | (GCGGG)7 |
|  |  | ENST00000357068 |  |
| PSMA4 | ENSG00000041357 | ENST00000413382 | (T)7 |
| PSME1 | ENSG00000092010 | ENST00000559123 |  |
| RANBP3 | ENSG00000031823 | ENST00000592621 | (C)7 |
|  |  | ENST00000590623 |  |
| RIC8B | ENSG00000111785 | ENST00000549643 | (G)7 |
| ROBO1 | ENSG00000169855 | ENST00000464233 | (T)7 |
| RPGRIP1 | ENSG00000092200 | ENST00000382933 | (A)7 |
|  |  | ENST00000557351 |  |
| SERINC2 | ENSG00000168528 | ENST00000536859 |  |
| SGK3 | ENSG00000104205 | ENST00000521960 |  |
| SLC2A14 | ENSG00000173262 | ENST00000543909 | (T)7 |
|  |  | ENST00000396589 |  |
|  |  | ENST00000537557 |  |
|  |  | ENST00000535266 |  |
| SLC4A1 | ENSG00000004939 | ENST00000631130 |  |
| SLC4A4 | ENSG00000080493 | ENST00000638464 | (TG)7 |
| SMPD4 | ENSG00000136699 | ENST00000441135 | (A)7 |
| sox5 | ENSG00000134532 | ENST00000441133 | (G)7 |
| STON1-GTF2A1L | ENSG00000068781 | ENST00000405008 | (T)7 |
|  |  | ENST00000402114 |  |
|  |  | ENST00000394754 |  |
| SUMO1 | ENSG00000116030 | ENST00000409498 |  |
|  |  | ENST00000409205 |  |
| SYCP1 | ENSG00000198765 | ENST00000369522 | (C)7 |
|  |  | ENST00000455987 |  |
|  |  | ENST00000618516 |  |
|  |  | ENST00000613524 |  |
|  |  | ENST00000369518 |  |
| TAF6 | ENSG00000106290 | ENST00000437822 | (A)7 |
| TSNAXIP1 | ENSG00000102904 | ENST00000415766 | (G)7 |
| TTC7A | ENSG00000068724 | ENST00000409245 | (T)7 |
| UAP1L1 | ENSG00000197355 | ENST00000360271 | (C)7 |
| UBR5 | ENSG00000104517 | ENST00000521566 |  |
| WFDC2 | ENSG00000101443 | ENST00000342873 | (G)7 |
| XPNPEP1 | ENSG00000108039 | ENST00000369683 | (T)7 |
| ZNF283 | ENSG00000167637 | ENST00000588797 | (A)7 |
| ZNF491 | ENSG00000177599 | ENST00000323169 |  |
|  |  | ENST00000450087 |  |
| ZNF71 | ENSG00000197951 | ENST00000599599 | (T)7 |
|  |  | ENST00000328070 |  |
| ZNF766 | ENSG00000196214 | ENST00000601711 | (A)7 |
|  |  | ENST00000593612 |  |
| ZNF85 | ENSG00000105750 | ENST00000596534 | (T)7 |
|  |  | ENST00000595742 |  |
| ZNHIT3 | ENSG00000273611 | ENST00000619730 |  |
|  |  | ENST00000612494 |  |
|  |  | ENST00000622013 |  |
| ABCA4 | ENSG00000198691 | ENST00000536513 | (T)6 |
| ACTN1 | ENSG00000072110 | ENST00000556433 | (G)6 |
| ADAM32 | ENSG00000197140 | ENST00000521741 | (T)6 |
| ADRA2C | ENSG00000184160 | ENST00000509482 | (G)6 |
| ADRB1 | ENSG00000043591 | ENST00000369295 | (C)6 |
| AKAP13 | ENSG00000170776 | ENST00000560340 |  |
| AKR1C3 | ENSG00000196139 | ENST00000380554 | (G)6 |
| ANKRD20A1 | ENSG00000260691 | ENST00000562196 |  |
| ANKRD20A3 | ENSG00000276203 | ENST00000611388 |  |
| ANKRD20A4 | ENSG00000172014 | ENST00000357336 |  |
|  |  | ENST00000615302 |  |
| APLP1 | ENSG00000105290 | ENST00000589743 | (C)6 |
|  |  | ENST00000592316 |  |
| ARHGAP5 | ENSG00000100852 | ENST00000396582 | (G)6 |
| ARHGEF2 | ENSG00000116584 | ENST00000313695 | (T)6 |
|  |  | ENST00000471589 |  |
|  |  | ENST00000497907 |  |
| ARHGEF35 | ENSG00000213214 | ENST00000378115 |  |
| ARID1B | ENSG00000049618 | ENST00000346085 | (G)6 |
| ARL16 | ENSG00000214087 | ENST00000570561 |  |
|  |  | ENST00000576135 |  |
| ATP1B3 | ENSG00000069849 | ENST00000475483 | (AC)6 |
|  |  | ENST00000462082 |  |
| ATP9A | ENSG00000054793 | ENST00000338821 | (G)6 |
| AUTS2 | ENSG00000158321 | ENST00000644506 | (C)6 |
|  |  | ENST00000644359 |  |
| B9D1 | ENSG00000108641 | ENST00000575403 | (T)6 |
|  |  | ENST00000477478 |  |
|  |  | ENST00000642870 |  |
|  |  | ENST00000575478 |  |
|  |  | ENST00000574508 |  |
|  |  | ENST00000487415 |  |
| BCAR1 | ENSG00000050820 | ENST00000418647 | (A)6 |
| BMP4 | ENSG00000125378 | ENST00000559501 |  |
| BTBD9 | ENSG00000183826 | ENST00000314100 |  |
|  |  | ENST00000497373 |  |
| C11orf54 | ENSG00000182919 | ENST00000540113 | (T)6 |
|  |  | ENST00000530620 |  |
| C16orf74 | ENSG00000154102 | ENST00000602675 | (CAG)6 |
|  |  |  | (C)6 |
|  |  | ENST00000602766 | (CAG)6 |
|  |  |  | (C)6 |
| C1orf159 | ENSG00000131591 | ENST00000379339 |  |
|  |  | ENST00000421241 |  |
|  |  | ENST00000379320 |  |
|  |  | ENST00000379319 |  |
|  |  | ENST00000434641 |  |
|  |  | ENST00000462097 |  |
|  |  | ENST00000475119 |  |
|  |  | ENST00000442117 |  |
|  |  | ENST00000427787 |  |
| C1orf226 | ENSG00000239887 | ENST00000426197 |  |
| C21orf62 | ENSG00000205929 | ENST00000490358 | (G)6 |
|  |  | ENST00000487113 |  |
| C22orf42 | ENSG00000205856 | ENST00000382097 | (T)6 |
| C2orf70 | ENSG00000173557 | ENST00000409392 | (GA)6 |
|  |  |  | (T)6 |
| C6orf106 | ENSG00000196821 | ENST00000374023 | (C)6 |
|  |  | ENST00000374026 |  |
| C9orf40 | ENSG00000135045 | ENST00000376854 |  |
| C9orf50 | ENSG00000179058 | ENST00000372478 |  |
| C9orf64 | ENSG00000165118 | ENST00000314700 | (T)6 |
| C9orf66 | ENSG00000183784 | ENST00000382387 | (C)6 |
| CACNA1C | ENSG00000151067 | ENST00000335762 | (G)6 |
|  |  | ENST00000399655 |  |
| CBWD1 | ENSG00000172785 | ENST00000314367 | (T)6 |
|  |  | ENST00000382389 |  |
| CBWD6 | ENSG00000215126 | ENST00000613716 |  |
| CCDC66 | ENSG00000180376 | ENST00000326595 | (A)6 |
| CCHCR1 | ENSG00000204536 | ENST00000513222 | (C)6 |
| CCT5 | ENSG00000150753 | ENST00000280326 | (G)6 |
|  |  | ENST00000503026 | (A)6 |
|  |  | ENST00000515390 | (G)6 |
| CD200 | ENSG00000091972 | ENST00000607597 |  |
| CD74 | ENSG00000019582 | ENST00000377795 |  |
|  |  | ENST00000353334 |  |
| CD81 | ENSG00000110651 | ENST00000492252 |  |
| CDC42BPA | ENSG00000143776 | ENST00000366766 | (A)6 |
| CDIPT | ENSG00000103502 | ENST00000561555 | (T)6 |
| CELF5 | ENSG00000161082 | ENST00000292672 | (CCG)6 |
| CENPB | ENSG00000125817 | ENST00000379751 | (G)6 |
| CFL1 | ENSG00000172757 | ENST00000527344 | (A)6 |
|  |  | ENST00000531407 |  |
|  |  | ENST00000524553 |  |
|  |  | ENST00000531413 |  |
|  |  | ENST00000530413 |  |
|  |  | ENST00000534784 |  |
| CHD2 | ENSG00000173575 | ENST00000625990 |  |
| CHD9 | ENSG00000177200 | ENST00000565442 |  |
| CHRNA4 | ENSG00000101204 | ENST00000615287 | (C)6 |
| CHRNB2 | ENSG00000160716 | ENST00000368476 |  |
|  |  | ENST00000637900 |  |
| CINP | ENSG00000100865 | ENST00000536961 | (T)6 |
| CIZ1 | ENSG00000148337 | ENST00000629610 | (G)6 |
| CLN3 | ENSG00000188603 | ENST00000568497 |  |
|  |  |  | (C)6 |
| CLPB | ENSG00000162129 | ENST00000437826 | (G)6 |
|  |  |  |  |
| CNPY1 | ENSG00000146910 | ENST00000636446 | (A)6 |
| CNTROB | ENSG00000170037 | ENST00000575408 | (T)6 |
| COQ5 | ENSG00000110871 | ENST00000552443 |  |
|  |  | ENST00000551769 |  |
| CORO7 | ENSG00000262246 | ENST00000571052 | (C)6 |
|  |  | ENST00000570645 |  |
|  |  | ENST00000576051 |  |
| COX8A | ENSG00000176340 | ENST00000314133 | (T)6 |
| CPAMD8 | ENSG00000160111 | ENST00000388925 | (C)6 |
| CSMD2 | ENSG00000121904 | ENST00000373381 | (T)6 |
| CTXN1 | ENSG00000178531 | ENST00000318978 | (GGC)6 |
| CUEDC1 | ENSG00000180891 | ENST00000577589 | (C)6 |
| CXCR4 | ENSG00000121966 | ENST00000409817 | (T)6 |
| CXorf40B | ENSG00000197021 | ENST00000462691 | (G)6 |
|  |  | ENST00000370406 |  |
|  |  | ENST00000355203 |  |
|  |  | ENST00000370404 |  |
|  |  | ENST00000370409 |  |
|  |  | ENST00000483447 |  |
| CYP46A1 | ENSG00000036530 | ENST00000380228 | (T)6 |
| CYTH2 | ENSG00000105443 | ENST00000620214 | (A)6 |
| DBN1 | ENSG00000113758 | ENST00000512501 |  |
|  |  |  | (G)6 |
| DCDC1 | ENSG00000170959 | ENST00000406071 | (T)6 |
| DCTN4 | ENSG00000132912 | ENST00000521728 |  |
| DCTN5 | ENSG00000166847 | ENST00000566298 |  |
| DDX58 | ENSG00000107201 | ENST00000379868 |  |
| DDX59 | ENSG00000118197 | ENST00000433235 |  |
|  |  | ENST00000453944 |  |
| DEK | ENSG00000124795 | ENST00000503715 | (A)6 |
| DHFR | ENSG00000228716 | ENST00000439211 | (G)6 |
|  |  | ENST00000505337 |  |
| DNAJB13 | ENSG00000187726 | ENST00000537753 |  |
| DPYSL4 | ENSG00000151640 | ENST00000338492 | (GCCC)6 |
| DTX2 | ENSG00000091073 | ENST00000446600 | (A)6 |
| DZIP1L | ENSG00000158163 | ENST00000327532 | (C)6 |
|  |  | ENST00000469243 |  |
|  |  | ENST00000467030 |  |
| EDC3 | ENSG00000179151 | ENST00000566219 | (T)6 |
| EGFLAM | ENSG00000164318 | ENST00000397202 |  |
| EIF4A1 | ENSG00000161960 | ENST00000578754 |  |
| ELP2 | ENSG00000134759 | ENST00000543127 |  |
| ENOPH1 | ENSG00000145293 | ENST00000509635 | (A)6 |
| ENTPD1 | ENSG00000138185 | ENST00000453258 | (T)6 |
|  |  | ENST00000543964 |  |
| EPB41 | ENSG00000159023 | ENST00000343067 | (C)6 |
|  |  | ENST00000347529 |  |
|  |  | ENST00000646260 |  |
|  |  | ENST00000373798 |  |
|  |  | ENST00000644600 |  |
|  |  | ENST00000643173 |  |
|  |  | ENST00000644780 |  |
|  |  | ENST00000643604 |  |
|  |  | ENST00000373797 |  |
|  |  | ENST00000349460 |  |
|  |  | ENST00000645184 |  |
| EPCAM | ENSG00000119888 | ENST00000405271 |  |
| EPHA5 | ENSG00000145242 | ENST00000273854 | (G)6 |
|  |  | ENST00000613740 |  |
|  |  | ENST00000622150 |  |
|  |  | ENST00000432638 |  |
|  |  | ENST00000354839 |  |
|  |  | ENST00000511294 |  |
| EPHB1 | ENSG00000154928 | ENST00000460895 | (T)6 |
|  |  | ENST00000497173 |  |
|  |  | ENST00000473867 |  |
|  |  | ENST00000474732 |  |
| ETFA | ENSG00000140374 | ENST00000560726 |  |
| EXTL3 | ENSG00000012232 | ENST00000523149 |  |
| FAM193A | ENSG00000125386 | ENST00000637812 | (C)6 |
| FAM57B | ENSG00000149926 | ENST00000561666 | (G)6 |
| FAM69A | ENSG00000154511 | ENST00000613047 | (T)6 |
| FBXW10 | ENSG00000171931 | ENST00000395665 | (C)6 |
|  |  | ENST00000308799 |  |
|  |  | ENST00000301938 |  |
| FCF1 | ENSG00000119616 | ENST00000534938 | (T)6 |
| FCHSD2 | ENSG00000137478 | ENST00000311172 | (A)6 |
|  |  | ENST00000409853 |  |
| FDFT1 | ENSG00000079459 | ENST00000528643 |  |
|  |  | ENST00000525777 |  |
| FEM1B | ENSG00000169018 | ENST00000566739 | (C)6 |
| FERMT3 | ENSG00000149781 | ENST00000541252 | (G)6 |
| FGFR1 | ENSG00000077782 | ENST00000335922 | (C)6 |
| FGG | ENSG00000171557 | ENST00000443553 | (A)6 |
|  |  | ENST00000393846 |  |
| FLG | ENSG00000143631 | ENST00000368799 |  |
| FLNB | ENSG00000136068 | ENST00000493452 |  |
| FMN2 | ENSG00000155816 | ENST00000319653 | (C)6 |
| FOXE1 | ENSG00000178919 | ENST00000375123 |  |
| FRG1 | ENSG00000109536 | ENST00000524583 | (T)6 |
|  |  | ENST00000531991 |  |
| GAB2 | ENSG00000033327 | ENST00000361507 | (G)6 |
| GBA | ENSG00000177628 | ENST00000428024 | (C)6 |
| GDA | ENSG00000119125 | ENST00000414671 | (A)6 |
| GDF1 | ENSG00000130283 | ENST00000247005 | (C)6 |
| GLE1 | ENSG00000119392 | ENST00000309971 | (G)6 |
|  |  | ENST00000372770 |  |
| GLIPR1 | ENSG00000139278 | ENST00000550491 | (T)6 |
| GNB3 | ENSG00000111664 | ENST00000229264 | (G)6 |
|  |  | ENST00000541257 |  |
|  |  | ENST00000541978 |  |
|  |  | ENST00000435982 |  |
| GNL1 | ENSG00000204590 | ENST00000433809 | (C)6 |
| GOSR2 | ENSG00000108433 | ENST00000640608 | (G)6 |
|  |  | ENST00000638634 |  |
|  |  | ENST00000623037 |  |
|  |  | ENST00000225567 |  |
|  |  | ENST00000415811 |  |
|  |  | ENST00000576910 |  |
|  |  | ENST00000640051 |  |
|  |  | ENST00000573224 |  |
| GP1BA | ENSG00000185245 | ENST00000329125 |  |
| GRASP | ENSG00000161835 | ENST00000546756 |  |
| GRHL2 | ENSG00000083307 | ENST00000395927 | (A)6 |
| GTF2H2C | ENSG00000183474 | ENST00000512736 |  |
|  |  | ENST00000510979 |  |
|  |  | ENST00000514162 |  |
|  |  | ENST00000380729 |  |
|  |  | ENST00000507595 |  |
| HAUS4 | ENSG00000092036 | ENST00000490506 | (T)6 |
|  |  |  | (TC)6 |
|  |  | ENST00000554406 | (T)6 |
|  |  |  | (TC)6 |
| HCN2 | ENSG00000099822 | ENST00000251287 | (CGG)6 |
| HEXB | ENSG00000049860 | ENST00000509579 | (G)6 |
| HEXDC | ENSG00000169660 | ENST00000578632 | (C)6 |
| HIBCH | ENSG00000198130 | ENST00000410045 | (A)6 |
| HIST3H2A | ENSG00000181218 | ENST00000366695 | (T)6 |
| HLA-DMA | ENSG00000204257 | ENST00000422832 |  |
| HLA-DRB3 | ENSG00000230463 | ENST00000426847 | (G)6 |
| HMG20A | ENSG00000140382 | ENST00000558651 | (C)6 |
|  |  | ENST00000558176 |  |
| HMGB4 | ENSG00000176256 | ENST00000519684 | (A)6 |
|  |  | ENST00000522796 |  |
| HNF1A | ENSG00000135100 | ENST00000615446 | (C)6 |
| HNRNPH1 | ENSG00000169045 | ENST00000521790 | (A)6 |
| HNRNPL | ENSG00000104824 | ENST00000600873 |  |
| HPD | ENSG00000158104 | ENST00000543163 | (G)6 |
| HSFX1 | ENSG00000171116 | ENST00000370416 | (C)6 |
| IL31RA | ENSG00000164509 | ENST00000490985 | (T)6 |
| INF2 | ENSG00000203485 | ENST00000617571 | (G)6 |
| ING1 | ENSG00000153487 | ENST00000375774 | (C)6 |
| INSM1 | ENSG00000173404 | ENST00000310227 | (G)6 |
| INTS6 | ENSG00000102786 | ENST00000497989 | (C)6 |
| IPO8 | ENSG00000133704 | ENST00000535989 | (T)6 |
|  |  | ENST00000543446 | (A)6 |
|  |  | ENST00000358724 | (T)6 |
| ITGA8 | ENSG00000077943 | ENST00000378076 | (C)6 |
| ITGAV | ENSG00000138448 | ENST00000433736 |  |
| IVL | ENSG00000163207 | ENST00000368764 | (A)6 |
| JUN | ENSG00000177606 | ENST00000371222 | (G)6 |
| KCNMA1 | ENSG00000156113 | ENST00000639601 | (GGC)6 |
| KCNN4 | ENSG00000104783 | ENST00000615047 | (C)6 |
| KCTD14 | ENSG00000151364 | ENST00000533144 | (T)6 |
| KIF12 | ENSG00000136883 | ENST00000374118 | (C)6 |
| KIF22 | ENSG00000079616 | ENST00000561482 |  |
|  |  | ENST00000569636 |  |
|  |  | ENST00000400751 |  |
|  |  | ENST00000569382 |  |
| KLHL17 | ENSG00000187961 | ENST00000622660 | (G)6 |
| KLK2 | ENSG00000167751 | ENST00000325321 | (C)6 |
|  |  | ENST00000600690 |  |
| KRT85 | ENSG00000135443 | ENST00000544265 | (A)6 |
| KSR1 | ENSG00000141068 | ENST00000268763 | (T)6 |
| LCAT | ENSG00000213398 | ENST00000570980 | (G)6 |
| LFNG | ENSG00000106003 | ENST00000402045 |  |
| LONP1 | ENSG00000196365 | ENST00000585374 |  |
|  |  |  |  |
|  |  | ENST00000590729 |  |
| LRCH1 | ENSG00000136141 | ENST00000463929 | (T)6 |
|  |  |  |  |
|  |  | ENST00000478412 |  |
|  |  |  |  |
| LRP1B | ENSG00000168702 | ENST00000389484 | (GCC)6 |
|  |  | ENST00000434794 |  |
| LRRC37A3 | ENSG00000176809 | ENST00000339474 | (T)6 |
|  |  |  |  |
| LRRC49 | ENSG00000137821 | ENST00000560158 | (A)6 |
| LSM5 | ENSG00000106355 | ENST00000409909 | (T)6 |
|  |  | ENST00000409292 |  |
|  |  | ENST00000410044 |  |
|  |  | ENST00000409782 |  |
|  |  | ENST00000409952 |  |
| MAD1L1 | ENSG00000002822 | ENST00000450235 | (A)6 |
| MAGEB16 | ENSG00000189023 | ENST00000399992 | (T)6 |
| MAGI2 | ENSG00000187391 | ENST00000354212 | (C)6 |
|  |  | ENST00000419488 |  |
|  |  | ENST00000637441 |  |
|  |  | ENST00000522391 |  |
| MAP2 | ENSG00000078018 | ENST00000199940 | (T)6 |
|  |  | ENST00000392193 |  |
|  |  | ENST00000360351 |  |
|  |  | ENST00000361559 |  |
|  |  | ENST00000445941 |  |
|  |  | ENST00000392194 |  |
| MED15 | ENSG00000099917 | ENST00000438962 | (C)6 |
| MED19 | ENSG00000156603 | ENST00000431606 | (A)6 |
|  |  | ENST00000337672 |  |
| MED26 | ENSG00000105085 | ENST00000598608 | (G)6 |
| MED27 | ENSG00000160563 | ENST00000292035 | (T)6 |
| MEP1B | ENSG00000141434 | ENST00000581184 |  |
| MFNG | ENSG00000100060 | ENST00000436341 | (G)6 |
|  |  | ENST00000442496 |  |
| MORN2 | ENSG00000188010 | ENST00000340556 | (T)6 |
|  |  | ENST00000410014 |  |
|  |  | ENST00000409665 |  |
| MRPL19 | ENSG00000115364 | ENST00000476622 |  |
| MRPL34 | ENSG00000130312 | ENST00000600434 |  |
|  |  | ENST00000594999 |  |
|  |  | ENST00000252602 |  |
| MRPS28 | ENSG00000147586 | ENST00000521434 |  |
|  |  | ENST00000519120 | (A)6 |
|  |  | ENST00000520946 | (T)6 |
| MSL1 | ENSG00000188895 | ENST00000582920 | (G)6 |
| MTA2 | ENSG00000149480 | ENST00000524902 | (C)6 |
|  |  | ENST00000527204 |  |
| MTHFD1 | ENSG00000100714 | ENST00000554739 | (G)6 |
|  |  | ENST00000554768 |  |
|  |  | ENST00000557539 |  |
| MTX3 | ENSG00000177034 | ENST00000512560 | (T)6 |
| MUC5B | ENSG00000117983 | ENST00000529681 | (C)6 |
| MVK | ENSG00000110921 | ENST00000539696 |  |
| MYL10 | ENSG00000106436 | ENST00000223167 | (T)6 |
| NBPF4 | ENSG00000196427 | ENST00000415641 |  |
| NDUFA5 | ENSG00000128609 | ENST00000470123 | (A)6 |
| NEDD4L | ENSG00000049759 | ENST00000592846 | (T)6 |
|  |  | ENST00000456986 |  |
|  |  | ENST00000435432 |  |
|  |  | ENST00000456173 |  |
|  |  | ENST00000588494 |  |
|  |  | ENST00000431212 |  |
|  |  | ENST00000586268 |  |
|  |  | ENST00000587190 |  |
|  |  | ENST00000635997 | (C)6 |
| NFATC4 | ENSG00000100968 | ENST00000413692 | (TG)6 |
|  |  | ENST00000554591 |  |
| NPTN | ENSG00000156642 | ENST00000565282 | (T)6 |
| OCEL1 | ENSG00000099330 | ENST00000597836 | (C)6 |
| OGDHL | ENSG00000197444 | ENST00000432695 | (T)6 |
| OGFOD1 | ENSG00000087263 | ENST00000561646 | (A)6 |
| OR11H12 | ENSG00000257115 | ENST00000550708 |  |
| OR2A1 | ENSG00000221970 | ENST00000641044 | (T)6 |
| OR2A12 | ENSG00000221858 | ENST00000641592 |  |
| OR2A42 | ENSG00000212807 | ENST00000641810 |  |
| OR2AJ1 | ENSG00000177275 | ENST00000318244 |  |
|  |  |  | (A)6 |
| OR2J3 | ENSG00000204701 | ENST00000641151 | (T)6 |
|  |  |  |  |
|  |  | ENST00000641960 |  |
|  |  |  |  |
| OR4E2 | ENSG00000221977 | ENST00000641524 |  |
| OR4N2 | ENSG00000176294 | ENST00000557414 |  |
|  |  | ENST00000557677 |  |
|  |  | ENST00000641240 |  |
| OR52I2 | ENSG00000226288 | ENST00000641896 | (A)6 |
|  |  | ENST00000641486 |  |
| OR5B3 | ENSG00000172769 | ENST00000641865 | (T)6 |
| OR5V1 | ENSG00000243729 | ENST00000641768 |  |
|  |  | ENST00000377154 |  |
| OR6N2 | ENSG00000188340 | ENST00000641131 | (GA)6 |
| OR7C1 | ENSG00000127530 | ENST00000641666 | (T)6 |
|  |  | ENST00000642030 |  |
|  |  | ENST00000642000 |  |
|  |  | ENST00000248073 |  |
| OR8G1 | ENSG00000197849 | ENST00000641972 |  |
| OR8G5 | ENSG00000255298 | ENST00000641992 |  |
|  |  | ENST00000641707 |  |
| OR9A4 | ENSG00000258083 | ENST00000641559 |  |
| OR9G4 | ENSG00000172457 | ENST00000641581 | (A)6 |
|  |  | ENST00000641668 |  |
| OSGIN1 | ENSG00000140961 | ENST00000343939 | (G)6 |
| PAAF1 | ENSG00000175575 | ENST00000536582 | (T)6 |
| PABPC4L | ENSG00000254535 | ENST00000421491 | (C)6 |
| PALB2 | ENSG00000083093 | ENST00000568219 | (A)6 |
|  |  |  |  |
| PBX1 | ENSG00000185630 | ENST00000560641 | (T)6 |
|  |  | ENST00000482110 |  |
|  |  | ENST00000558837 |  |
|  |  | ENST00000559560 |  |
| PCDHA10 | ENSG00000250120 | ENST00000562220 | (A)6 |
| PCDHA5 | ENSG00000204965 | ENST00000614258 |  |
|  |  | ENST00000529619 |  |
| PCDHB14 | ENSG00000120327 | ENST00000624896 | (T)6 |
| PCDHB5 | ENSG00000113209 | ENST00000623915 | (A)6 |
| PDE2A | ENSG00000186642 | ENST00000542223 |  |
| PDE4B | ENSG00000184588 | ENST00000371045 | (G)6 |
|  |  |  |  |
| PLCZ1 | ENSG00000139151 | ENST00000538330 | (T)6 |
|  |  | ENST00000543242 |  |
| PNMA6A | ENSG00000235961 | ENST00000421798 | (C)6 |
| PNMT | ENSG00000141744 | ENST00000269582 | (A)6 |
| POLD4 | ENSG00000175482 | ENST00000529704 | (C)6 |
|  |  |  |  |
|  |  | ENST00000530584 |  |
|  |  |  |  |
|  |  | ENST00000532830 |  |
|  |  |  |  |
|  |  | ENST00000531239 |  |
|  |  |  |  |
| PPM1D | ENSG00000170836 | ENST00000590418 | (A)6 |
| PPM1M | ENSG00000164088 | ENST00000296487 | (C)6 |
| PPP1R12C | ENSG00000125503 | ENST00000435544 |  |
| PROCA1 | ENSG00000167525 | ENST00000415329 |  |
| PRRT4 | ENSG00000224940 | ENST00000489835 | (G)6 |
|  |  | ENST00000446477 |  |
|  |  | ENST00000535159 |  |
|  |  | ENST00000489517 |  |
|  |  | ENST00000464607 |  |
| PSMA4 | ENSG00000041357 | ENST00000413382 | (T)6 |
| PSMB3 | ENSG00000277791 | ENST00000610434 | (C)6 |
| PTGER3 | ENSG00000050628 | ENST00000370931 | (G)6 |
|  |  | ENST00000628037 |  |
|  |  | ENST00000370932 |  |
|  |  | ENST00000356595 |  |
|  |  | ENST00000306666 |  |
|  |  | ENST00000351052 |  |
|  |  | ENST00000354608 |  |
| PTPN7 | ENSG00000143851 | ENST00000476061 |  |
| PVALB | ENSG00000100362 | ENST00000404171 | (C)6 |
|  |  |  |  |
| QARS | ENSG00000172053 | ENST00000635541 | (G)6 |
|  |  |  | (T)6 |
|  |  | ENST00000635622 | (G)6 |
|  |  |  | (T)6 |
| RAB24 | ENSG00000169228 | ENST00000303270 | (G)6 |
| RAB40AL | ENSG00000102128 | ENST00000218249 |  |
| RAD50 | ENSG00000113522 | ENST00000416135 | (T)6 |
| RBMY1A1 | ENSG00000234414 | ENST00000439108 |  |
|  |  | ENST00000303902 |  |
|  |  | ENST00000382707 |  |
| RBMY1B | ENSG00000242875 | ENST00000383020 |  |
|  |  | ENST00000619219 |  |
| RBMY1D | ENSG00000244395 | ENST00000382680 |  |
| RBMY1E | ENSG00000242389 | ENST00000382659 |  |
| RBMY1F | ENSG00000169800 | ENST00000303766 |  |
| RETN | ENSG00000104918 | ENST00000221515 | (G)6 |
|  |  | ENST00000629642 |  |
| RFPL1 | ENSG00000128250 | ENST00000354373 |  |
|  |  |  |  |
| RFPL2 | ENSG00000128253 | ENST00000248980 |  |
|  |  |  |  |
| RFPL3 | ENSG00000128276 | ENST00000249007 |  |
|  |  |  |  |
| RGPD5 | ENSG00000015568 | ENST00000432606 | (A)6 |
| RGPD8 | ENSG00000169629 | ENST00000409750 |  |
| RIMS1 | ENSG00000079841 | ENST00000517827 | (T)6 |
| RNF167 | ENSG00000108523 | ENST00000570328 |  |
|  |  | ENST00000576229 |  |
| ROR1 | ENSG00000185483 | ENST00000371080 | (GCC)6 |
|  |  | ENST00000371079 |  |
| RPL23 | ENSG00000125691 | ENST00000245857 | (T)6 |
|  |  | ENST00000470646 |  |
| RPS3 | ENSG00000149273 | ENST00000422465 |  |
|  |  | ENST00000528847 |  |
| RRAS2 | ENSG00000133818 | ENST00000414023 | (A)6 |
|  |  | ENST00000529237 |  |
|  |  | ENST00000534746 |  |
|  |  | ENST00000526063 |  |
|  |  | ENST00000532814 |  |
|  |  | ENST00000531421 |  |
| RRBP1 | ENSG00000125844 | ENST00000470422 | (C)6 |
| RTN2 | ENSG00000125744 | ENST00000590526 | (A)6 |
| SACM1L | ENSG00000211456 | ENST00000438671 | (T)6 |
| SAMD1 | ENSG00000141858 | ENST00000533683 | (C)6 |
|  |  |  | (G)6 |
| SCAF1 | ENSG00000126461 | ENST00000360565 | (C)6 |
|  |  | ENST00000598359 |  |
|  |  | ENST00000601038 |  |
| SCML1 | ENSG00000047634 | ENST00000398080 | (T)6 |
|  |  | ENST00000380045 |  |
| SCRIB | ENSG00000180900 | ENST00000377533 |  |
| SDK1 | ENSG00000146555 | ENST00000404826 | (G)6 |
| SEC14L2 | ENSG00000100003 | ENST00000429917 | (C)6 |
| SEC61B | ENSG00000106803 | ENST00000498603 | (G)6 |
| SEL1L2 | ENSG00000101251 | ENST00000646153 | (T)6 |
|  |  | ENST00000473203 |  |
| SERHL2 | ENSG00000183569 | ENST00000447870 | (G)6 |
| SERPINB13 | ENSG00000197641 | ENST00000431153 | (T)6 |
| SFRP1 | ENSG00000104332 | ENST00000379845 | (G)6 |
| SFRP4 | ENSG00000106483 | ENST00000447200 | (A)6 |
| SGK1 | ENSG00000118515 | ENST00000237305 | (T)6 |
|  |  | ENST00000461976 |  |
| SH3GL3 | ENSG00000140600 | ENST00000427482 | (G)6 |
| SIRPG | ENSG00000089012 | ENST00000381580 | (A)6 |
| SLC16A2 | ENSG00000147100 | ENST00000587091 | (C)6 |
| SLC39A6 | ENSG00000141424 | ENST00000440549 | (A)6 |
| SMTN | ENSG00000183963 | ENST00000404574 | (C)6 |
| SNRPA | ENSG00000077312 | ENST00000601545 | (A)6 |
| SNX31 | ENSG00000174226 | ENST00000428383 |  |
| SOD3 | ENSG00000109610 | ENST00000598411 | (G)6 |
| SPATA4 | ENSG00000150628 | ENST00000515234 | (T)6 |
| SPDYE2 | ENSG00000205238 | ENST00000341656 | (C)6 |
| SPIN4 | ENSG00000186767 | ENST00000374884 | (A)6 |
| SPINK13 | ENSG00000214510 | ENST00000511106 | (T)6 |
| SPOCD1 | ENSG00000134668 | ENST00000257100 | (G)6 |
|  |  | ENST00000528791 |  |
| SRPK1 | ENSG00000096063 | ENST00000423325 | (T)6 |
| SRSF11 | ENSG00000116754 | ENST00000370949 | (A)6 |
| SRXN1 | ENSG00000271303 | ENST00000381962 | (G)6 |
| SS18 | ENSG00000141380 | ENST00000542420 | (T)6 |
|  |  | ENST00000579061 |  |
|  |  | ENST00000584083 |  |
|  |  | ENST00000581021 |  |
| SSBP4 | ENSG00000130511 | ENST00000599699 | (C)6 |
| STON1 | ENSG00000243244 | ENST00000404752 |  |
|  |  | ENST00000406226 |  |
| STON1-GTF2A1L | ENSG00000068781 | ENST00000405008 |  |
|  |  | ENST00000402114 |  |
|  |  | ENST00000394754 |  |
| SUMO1 | ENSG00000116030 | ENST00000409498 | (T)6 |
|  |  | ENST00000409205 |  |
| SYCE1L | ENSG00000205078 | ENST00000378644 |  |
| SYDE1 | ENSG00000105137 | ENST00000600252 | (C)6 |
| SYNE1 | ENSG00000131018 | ENST00000448038 | (A)6 |
| SYNGAP1 | ENSG00000197283 | ENST00000636905 | (GGC)6 |
| TAAR2 | ENSG00000146378 | ENST00000275191 | (T)6 |
| TARM1 | ENSG00000248385 | ENST00000616041 | (GAAA)6 |
| TAS2R1 | ENSG00000169777 | ENST00000382492 | (T)6 |
|  |  |  |  |
| TBCA | ENSG00000171530 | ENST00000522370 |  |
| TBL1Y | ENSG00000092377 | ENST00000383032 | (A)6 |
|  |  |  | (T)6 |
|  |  | ENST00000355162 | (A)6 |
|  |  |  | (T)6 |
|  |  | ENST00000346432 | (A)6 |
|  |  |  | (T)6 |
| TCIRG1 | ENSG00000110719 | ENST00000532635 | (G)6 |
| TET2 | ENSG00000168769 | ENST00000305737 | (ATAG)6 |
|  |  | ENST00000540549 |  |
|  |  | ENST00000380013 |  |
|  |  | ENST00000413648 |  |
|  |  | ENST00000514870 |  |
| TICAM1 | ENSG00000127666 | ENST00000248244 | (G)6 |
| TIGIT | ENSG00000181847 | ENST00000481065 | (A)6 |
| TLE2 | ENSG00000065717 | ENST00000591529 | (T)6 |
| TM4SF19 | ENSG00000145107 | ENST00000446879 | (C)6 |
|  |  | ENST00000454715 |  |
| TMEM132A | ENSG00000006118 | ENST00000544065 | (G)6 |
|  |  |  | (C)6 |
| TMEM229B | ENSG00000198133 | ENST00000557006 |  |
|  |  | ENST00000357461 |  |
|  |  | ENST00000554278 |  |
|  |  | ENST00000554480 |  |
|  |  | ENST00000555994 |  |
|  |  | ENST00000557779 |  |
| TMEM63A | ENSG00000196187 | ENST00000537914 |  |
| TMEM72 | ENSG00000187783 | ENST00000544540 | (T)6 |
| TNFRSF18 | ENSG00000186891 | ENST00000486728 | (G)6 |
| TNKS | ENSG00000173273 | ENST00000518281 | (T)6 |
| TNXB | ENSG00000168477 | ENST00000644971 | (G)6 |
|  |  | ENST00000375244 |  |
|  |  | ENST00000479795 |  |
|  |  | ENST00000442721 |  |
| TPSAB1 | ENSG00000172236 | ENST00000338844 | (C)6 |
|  |  | ENST00000461509 |  |
| TPSD1 | ENSG00000095917 | ENST00000397534 |  |
|  |  | ENST00000211076 |  |
| TRIM16L | ENSG00000108448 | ENST00000395671 |  |
| TRIM45 | ENSG00000134253 | ENST00000369461 |  |
| TRPM8 | ENSG00000144481 | ENST00000433712 | (T)6 |
| TTC27 | ENSG00000018699 | ENST00000448773 |  |
| TUBA8 | ENSG00000183785 | ENST00000416740 | (G)6 |
| TXNDC2 | ENSG00000168454 | ENST00000536353 | (T)6 |
|  |  | ENST00000584255 |  |
|  |  | ENST00000357775 |  |
| UBR5 | ENSG00000104517 | ENST00000521566 |  |
| UNC5C | ENSG00000182168 | ENST00000453304 | (C)6 |
|  |  | ENST00000513796 |  |
|  |  | ENST00000506749 |  |
|  |  | ENST00000504962 |  |
| USP15 | ENSG00000135655 | ENST00000548836 | (T)6 |
|  |  |  | (A)6 |
| USP30 | ENSG00000135093 | ENST00000392784 | (T)6 |
|  |  | ENST00000536723 |  |
| USP9Y | ENSG00000114374 | ENST00000338981 | (A)6 |
| VAMP2 | ENSG00000220205 | ENST00000404970 | (C)6 |
| VGLL4 | ENSG00000144560 | ENST00000458499 | (A)6 |
| VMA21 | ENSG00000160131 | ENST00000370361 | (T)6 |
| VPS26A | ENSG00000122958 | ENST00000489794 |  |
| WDR11 | ENSG00000120008 | ENST00000604585 |  |
| WDR78 | ENSG00000152763 | ENST00000488333 |  |
|  |  |  |  |
| WDR92 | ENSG00000243667 | ENST00000406245 | (A)6 |
| WSCD2 | ENSG00000075035 | ENST00000551638 | (T)6 |
| WWOX | ENSG00000186153 | ENST00000566780 | (G)6 |
|  |  | ENST00000627394 |  |
|  |  | ENST00000402655 |  |
|  |  | ENST00000406884 |  |
|  |  | ENST00000539474 |  |
|  |  | ENST00000569818 |  |
|  |  | ENST00000355860 |  |
|  |  | ENST00000408984 |  |
| XKR7 | ENSG00000260903 | ENST00000562532 | (C)6 |
| YEATS4 | ENSG00000127337 | ENST00000549685 | (T)6 |
| YTHDC1 | ENSG00000083896 | ENST00000505251 | (C)6 |
| ZAN | ENSG00000146839 | ENST00000613979 | (G)6 |
|  |  | ENST00000620596 |  |
|  |  | ENST00000618565 |  |
| ZDHHC8 | ENSG00000099904 | ENST00000334554 | (CGC)6 |
| ZFHX3 | ENSG00000140836 | ENST00000397992 | (G)6 |
| ZFYVE1 | ENSG00000165861 | ENST00000394207 |  |
|  |  | ENST00000555072 |  |
| ZNF136 | ENSG00000196646 | ENST00000439995 | (T)6 |
| ZNF138 | ENSG00000197008 | ENST00000437743 | (TG)6 |
| ZNF140 | ENSG00000196387 | ENST00000544426 | (A)6 |
| ZNF180 | ENSG00000167384 | ENST00000592529 | (G)6 |
|  |  | ENST00000586637 |  |
|  |  | ENST00000591064 |  |
| ZNF2 | ENSG00000275111 | ENST00000614034 | (C)6 |
|  |  | ENST00000622059 |  |
| ZNF257 | ENSG00000197134 | ENST00000597927 | (A)6 |
| ZNF28 | ENSG00000198538 | ENST00000438150 | (T)6 |
|  |  | ENST00000391783 |  |
| ZNF345 | ENSG00000251247 | ENST00000589046 | (A)6 |
|  |  | ENST00000586933 |  |
|  |  | ENST00000614069 |  |
|  |  | ENST00000532141 |  |
|  |  | ENST00000420450 |  |
|  |  | ENST00000526123 |  |
|  |  | ENST00000529555 |  |
|  |  | ENST00000331800 |  |
|  |  | ENST00000612719 |  |
|  |  | ENST00000586646 |  |
|  |  | ENST00000585396 |  |
| ZNF438 | ENSG00000183621 | ENST00000375311 |  |
|  |  |  |  |
| ZNF442 | ENSG00000198342 | ENST00000438182 | (T)6 |
|  |  | ENST00000424168 |  |
| ZNF529 | ENSG00000186020 | ENST00000585960 | (A)6 |
| ZNF546 | ENSG00000187187 | ENST00000599504 | (C)6 |
|  |  | ENST00000601138 |  |
| ZNF548 | ENSG00000188785 | ENST00000594668 | (T)6 |
| ZNF600 | ENSG00000189190 | ENST00000338230 |  |
| ZNF638 | ENSG00000075292 | ENST00000466330 |  |
| ZNF699 | ENSG00000196110 | ENST00000591998 |  |
| ZNF714 | ENSG00000160352 | ENST00000616183 | (A)6 |
| ZNF765 | ENSG00000196417 | ENST00000505866 | (T)6 |
| ZNF813 | ENSG00000198346 | ENST00000468450 |  |
| ZNF823 | ENSG00000197933 | ENST00000586121 | (C)6 |
| ZNF85 | ENSG00000105750 | ENST00000596534 | (A)6 |
|  |  | ENST00000595742 |  |
| ZSWIM1 | ENSG00000168612 | ENST00000372523 |  |
| ZYG11A | ENSG00000203995 | ENST00000371532 |  |
|  |  | ENST00000612017 |  |
| ACSBG1 | ENSG00000103740 | ENST00000559241 | (G)6 |
| ADAM32 | ENSG00000197140 | ENST00000523400 | (A)6 |
| ASH2L | ENSG00000129691 | ENST00000545394 |  |
| BIRC2 | ENSG00000110330 | ENST00000533742 |  |
| CACNA1A | ENSG00000141837 | ENST00000574974 | (C)6 |
|  |  | ENST00000592864 |  |
| CACNA1C | ENSG00000151067 | ENST00000543114 | (A)6 |
| CBWD5 | ENSG00000147996 | ENST00000377392 | (T)6 |
| EFHB | ENSG00000163576 | ENST00000344838 |  |
| EML2 | ENSG00000125746 | ENST00000586770 | (A)6 |
|  |  | ENST00000591721 |  |
|  |  | ENST00000593255 |  |
|  |  | ENST00000590043 |  |
|  |  | ENST00000590018 |  |
| GDA | ENSG00000119125 | ENST00000545168 |  |
| GDI2 | ENSG00000057608 | ENST00000380127 | (T)6 |
|  |  | ENST00000609712 |  |
| GLIPR1L2 | ENSG00000180481 | ENST00000378692 | (A)6 |
| GTF2H1 | ENSG00000110768 | ENST00000524753 | (T)6 |
| IMPACT | ENSG00000154059 | ENST00000585067 | (A)6 |
| INTS6 | ENSG00000102786 | ENST00000491189 |  |
|  |  | ENST00000488009 |  |
| IPO8 | ENSG00000133704 | ENST00000544829 |  |
| IQCJ-SCHIP1 | ENSG00000283154 | ENST00000638749 | (T)6 |
| KNTC1 | ENSG00000184445 | ENST00000423927 |  |
| LIMA1 | ENSG00000050405 | ENST00000547825 | (A)6 |
| MACF1 | ENSG00000127603 | ENST00000530275 | (G)6 |
| MAVS | ENSG00000088888 | ENST00000416600 | (C)6 |
| MGLL | ENSG00000074416 | ENST00000398104 | (A)6 |
|  |  | ENST00000434178 |  |
| MRPL13 | ENSG00000172172 | ENST00000518918 | (C)6 |
| MYL10 | ENSG00000106436 | ENST00000223167 | (T)6 |
| NFE2L2 | ENSG00000116044 | ENST00000397063 | (A)6 |
|  |  | ENST00000446151 |  |
|  |  | ENST00000464747 |  |
|  |  | ENST00000449627 |  |
|  |  | ENST00000448782 |  |
|  |  | ENST00000421929 |  |
|  |  | ENST00000423513 |  |
|  |  | ENST00000588123 |  |
| NFIA | ENSG00000162599 | ENST00000371189 |  |
| OGT | ENSG00000147162 | ENST00000444774 | (C)6 |
| OR2AP1 | ENSG00000179615 | ENST00000641114 | (T)6 |
| OR4A47 | ENSG00000237388 | ENST00000446524 | (C)6 |
| OR4E2 | ENSG00000221977 | ENST00000641524 |  |
| OR6C1 | ENSG00000205330 | ENST00000642104 | (A)6 |
| OR8G1 | ENSG00000197849 | ENST00000641972 | (T)6 |
| PDCL3 | ENSG00000115539 | ENST00000416255 | (A)6 |
| PIP5K1A | ENSG00000143398 | ENST00000447555 | (T)6 |
| PITPNA | ENSG00000174238 | ENST00000576722 |  |
|  |  | ENST00000575895 |  |
|  |  | ENST00000576010 |  |
|  |  | ENST00000573231 |  |
| RAB30 | ENSG00000137502 | ENST00000524635 | (C)6 |
| RAD21 | ENSG00000164754 | ENST00000518055 | (A)6 |
| RNF165 | ENSG00000141622 | ENST00000593230 | (C)6 |
| SACM1L | ENSG00000211456 | ENST00000433336 | (T)6 |
| TIGD7 | ENSG00000140993 | ENST00000396862 | (A)6 |
|  |  | ENST00000573608 |  |
| TMEM30A | ENSG00000112697 | ENST00000370050 | (T)6 |
| TOM1L2 | ENSG00000175662 | ENST00000478943 |  |
| TRAPPC2 | ENSG00000196459 | ENST00000359680 | (A)6 |
|  |  | ENST00000380579 |  |
|  |  | ENST00000519885 |  |
| TREML4 | ENSG00000188056 | ENST00000341495 | (T)6 |
|  |  | ENST00000448827 |  |
| TUBGCP2 | ENSG00000130640 | ENST00000543663 | (A)6 |
|  |  | ENST00000368563 |  |
| UBR5 | ENSG00000104517 | ENST00000521566 |  |
| VCAN | ENSG00000038427 | ENST00000512590 | (T)6 |
| ZNF678 | ENSG00000181450 | ENST00000343776 | (A)6 |
|  |  | ENST00000608949 |  |
| ABCG1 | ENSG00000160179 | ENST00000347800 | (CT)5 |
| ANK3 | ENSG00000151150 | ENST00000373827 | (AGC)5 |
| ARNT2 | ENSG00000172379 | ENST00000525103 | (TC)5 |
| ASAH2B | ENSG00000204147 | ENST00000374007 | (TG)5 |
|  |  | ENST00000647317 |  |
| ASH2L | ENSG00000129691 | ENST00000545394 | (GT)5 |
| BARX1 | ENSG00000131668 | ENST00000253968 | (CGC)5 |
| C12orf4 | ENSG00000047621 | ENST00000542080 | (AG)5 |
| C17orf49 | ENSG00000258315 | ENST00000546495 | (GCG)5 |
|  |  | ENST00000546760 |  |
|  |  | ENST00000552402 |  |
|  |  | ENST00000439424 |  |
| CCDC91 | ENSG00000123106 | ENST00000540794 | (CT)5 |
| CELF5 | ENSG00000161082 | ENST00000541430 | (CGC)5 |
| CLDN3 | ENSG00000165215 | ENST00000395145 | (GTCC)5 |
| DOCK2 | ENSG00000134516 | ENST00000540750 | (TC)5 |
| FAM43A | ENSG00000185112 | ENST00000329759 | (CCG)5 |
| FAM86B2 | ENSG00000145002 | ENST00000309608 | (TG)5 |
| FEZ2 | ENSG00000171055 | ENST00000305852 | (GT)5 |
| FOSB | ENSG00000125740 | ENST00000592811 | (TG)5 |
|  |  | ENST00000586615 |  |
|  |  | ENST00000589593 |  |
| HDGFL1 | ENSG00000112273 | ENST00000510882 | (GC)5 |
| IMPDH1 | ENSG00000106348 | ENST00000496200 | (AGC)5 |
|  |  | ENST00000626419 |  |
|  |  | ENST00000470772 |  |
|  |  | ENST00000480861 |  |
| KRTAP15-1 | ENSG00000186970 | ENST00000334067 | (CA)5 |
| MASP1 | ENSG00000127241 | ENST00000439271 | (GA)5 |
| MKNK1 | ENSG00000079277 | ENST00000532110 | (AG)5 |
| MRPL10 | ENSG00000159111 | ENST00000290208 | (TTCC)5 |
|  |  | ENST00000414011 |  |
| NF1 | ENSG00000196712 | ENST00000490416 | (TG)5 |
| NFKBIE | ENSG00000146232 | ENST00000275015 | (AATG)5 |
| NLE1 | ENSG00000073536 | ENST00000586869 | (GT)5 |
| NUP50 | ENSG00000093000 | ENST00000407019 | (TTG)5 |
|  |  | ENST00000417702 |  |
|  |  | ENST00000396096 |  |
| OR14A16 | ENSG00000196772 | ENST00000641093 | (GT)5 |
| OR1C1 | ENSG00000221888 | ENST00000641256 | (TC)5 |
| OR6C3 | ENSG00000205329 | ENST00000641364 | (GA)5 |
|  |  | ENST00000641740 |  |
| PCSK6 | ENSG00000140479 | ENST00000611716 | (GGC)5 |
|  |  | ENST00000331826 |  |
| PFN2 | ENSG00000070087 | ENST00000481767 | (TG)5 |
|  |  | ENST00000494827 |  |
|  |  | ENST00000497148 |  |
|  |  | ENST00000475518 |  |
|  |  | ENST00000481275 |  |
|  |  | ENST00000498307 |  |
|  |  | ENST00000489155 |  |
| PLOD2 | ENSG00000152952 | ENST00000461497 | (AT)5 |
| RALYL | ENSG00000184672 | ENST00000522613 | (GTTT)5 |
|  |  | ENST00000522455 |  |
|  |  | ENST00000521695 |  |
|  |  | ENST00000521268 |  |
|  |  | ENST00000518566 |  |
|  |  | ENST00000517988 |  |
|  |  | ENST00000522647 |  |
| RIC8B | ENSG00000111785 | ENST00000549643 | (TG)5 |
| RPL23 | ENSG00000125691 | ENST00000378096 | (GA)5 |
| RYR2 | ENSG00000198626 | ENST00000366574 | (CCG)5 |
| SERPINA9 | ENSG00000170054 | ENST00000424550 | (CA)5 |
| SLC35B2 | ENSG00000157593 | ENST00000537814 | (AG)5 |
| STK33 | ENSG00000130413 | ENST00000358872 | (TA)5 |
| STRA6 | ENSG00000137868 | ENST00000574278 | (TG)5 |
| TBC1D16 | ENSG00000167291 | ENST00000573782 | (AG)5 |
|  |  | ENST00000574427 |  |
| ZNF626 | ENSG00000188171 | ENST00000595405 | (AT)5 |
| ACADSB | ENSG00000196177 | ENST00000368869 | (TAA)4 |
| ACTN2 | ENSG00000077522 | ENST00000546208 | (TG)4 |
| ADAP2 | ENSG00000184060 | ENST00000581548 | (CT)4 |
| ADARB2 | ENSG00000185736 | ENST00000381312 | (GCG)4 |
| AIG1 | ENSG00000146416 | ENST00000458219 | (TC)4 |
| AKAP8L | ENSG00000011243 | ENST00000600247 | (CTATGG)4 |
| ANKRD30B | ENSG00000180777 | ENST00000358984 | (CT)4 |
| ARHGAP5 | ENSG00000100852 | ENST00000396582 | (GCG)4 |
| ARHGAP8 | ENSG00000241484 | ENST00000389774 | (GGC)4 |
|  |  | ENST00000396119 |  |
|  |  | ENST00000336963 |  |
|  |  | ENST00000356099 |  |
|  |  | ENST00000412433 |  |
| ARL14 | ENSG00000179674 | ENST00000320767 | (AAGA)4 |
|  |  |  | (AG)4 |
| ASGR1 | ENSG00000141505 | ENST00000380920 | (CTGGGG)4 |
|  |  | ENST00000570576 |  |
| ATAD3B | ENSG00000160072 | ENST00000308647 | (GCG)4 |
| AUTS2 | ENSG00000158321 | ENST00000443672 | (CAGCAC)4 |
| BAIAP2 | ENSG00000175866 | ENST00000575989 | (TC)4 |
|  |  | ENST00000575958 |  |
|  |  | ENST00000573659 |  |
|  |  | ENST00000572073 |  |
|  |  | ENST00000573677 |  |
| BATF2 | ENSG00000168062 | ENST00000527716 | (GA)4 |
| BCAP29 | ENSG00000075790 | ENST00000465919 | (AC)4 |
| BEGAIN | ENSG00000183092 | ENST00000554140 | (GCG)4 |
| BRD2 | ENSG00000204256 | ENST00000449085 | (CT)4 |
| BUB1 | ENSG00000169679 | ENST00000447014 | (TA)4 |
|  |  | ENST00000420328 |  |
| C19orf66 | ENSG00000130813 | ENST00000397881 | (TC)4 |
| C3orf20 | ENSG00000131379 | ENST00000435614 | (CCA)4 |
|  |  | ENST00000412910 |  |
| CAPNS1 | ENSG00000126247 | ENST00000592354 | (CA)4 |
| CCDC149 | ENSG00000181982 | ENST00000389609 | (TCA)4 |
| CCS | ENSG00000173992 | ENST00000534763 | (AC)4 |
| CD81 | ENSG00000110651 | ENST00000475945 | (TC)4 |
|  |  | ENST00000530648 |  |
|  |  | ENST00000492627 |  |
|  |  | ENST00000526072 |  |
| CDH2 | ENSG00000170558 | ENST00000269141 | (CCGCCG)4 |
| CENPB | ENSG00000125817 | ENST00000379751 | (CGG)4 |
| CENPM | ENSG00000100162 | ENST00000404067 | (CT)4 |
|  |  | ENST00000402338 |  |
|  |  | ENST00000402420 |  |
| CFHR1 | ENSG00000244414 | ENST00000320493 | (CAC)4 |
|  |  | ENST00000367424 |  |
| CFHR2 | ENSG00000080910 | ENST00000476712 |  |
|  |  | ENST00000367415 |  |
| CHIA | ENSG00000134216 | ENST00000483391 | (AG)4 |
|  |  | ENST00000451398 |  |
|  |  | ENST00000353665 |  |
|  |  | ENST00000489524 |  |
| CHTF18 | ENSG00000127586 | ENST00000262315 | (CGG)4 |
|  |  | ENST00000455171 |  |
|  |  | ENST00000317063 |  |
| CNGA1 | ENSG00000198515 | ENST00000402813 | (AT)4 |
| COL6A1 | ENSG00000142156 | ENST00000361866 | (CGG)4 |
|  |  | ENST00000612273 |  |
| COQ5 | ENSG00000110871 | ENST00000552443 | (TG)4 |
|  |  | ENST00000551769 |  |
| CRTC1 | ENSG00000105662 | ENST00000321949 | (GAG)4 |
| CSMD1 | ENSG00000183117 | ENST00000400186 | (CT)4 |
|  |  | ENST00000602723 |  |
|  |  | ENST00000635120 |  |
|  |  | ENST00000520002 |  |
|  |  | ENST00000602557 |  |
| CYFIP1 | ENSG00000273749 | ENST00000617556 | (TC)4 |
| DDHD2 | ENSG00000085788 | ENST00000527834 | (TGT)4 |
|  |  | ENST00000397166 |  |
|  |  | ENST00000533100 |  |
|  |  | ENST00000528358 |  |
|  |  | ENST00000532222 |  |
|  |  | ENST00000520272 |  |
| DHRSX | ENSG00000169084 | ENST00000334651 | (GCG)4 |
|  |  | ENST00000412516 |  |
| DLG2 | ENSG00000150672 | ENST00000376104 | (ATA)4 |
| DOK2 | ENSG00000147443 | ENST00000518197 | (TC)4 |
| DPY19L2 | ENSG00000177990 | ENST00000541083 | (ATA)4 |
| EFHC1 | ENSG00000096093 | ENST00000637315 | (AC)4 |
| EPHA10 | ENSG00000183317 | ENST00000540011 | (TC)4 |
| EPS8L1 | ENSG00000131037 | ENST00000586329 | (TCC)4 |
| ESCO1 | ENSG00000141446 | ENST00000622333 | (TATA)4 |
| ESRP2 | ENSG00000103067 | ENST00000564382 | (TC)4 |
|  |  | ENST00000562724 |  |
| FAM118A | ENSG00000100376 | ENST00000452238 | (TG)4 |
|  |  | ENST00000424557 |  |
| FAM43B | ENSG00000183114 | ENST00000332947 | (CGC)4 |
| FAM50A | ENSG00000071859 | ENST00000393600 | (CCGCCG)4 |
| FBXW11 | ENSG00000072803 | ENST00000518752 | (TC)4 |
| FGG | ENSG00000171557 | ENST00000443553 | (TAA)4 |
|  |  | ENST00000393846 |  |
| FMNL3 | ENSG00000161791 | ENST00000550424 | (TA)4 |
| FOSB | ENSG00000125740 | ENST00000592811 | (GT)4 |
|  |  |  | (TG)4 |
|  |  | ENST00000586615 | (GT)4 |
|  |  |  | (TG)4 |
|  |  | ENST00000589593 | (GT)4 |
|  |  |  | (TG)4 |
| FOXR2 | ENSG00000189299 | ENST00000339140 | (TC)4 |
| FRG2 | ENSG00000205097 | ENST00000504750 | (GA)4 |
| FRG2B | ENSG00000225899 | ENST00000443774 |  |
| FUT4 | ENSG00000196371 | ENST00000358752 |  |
| GABRG2 | ENSG00000113327 | ENST00000640985 | (AC)4 |
| GALK2 | ENSG00000156958 | ENST00000560654 | (TG)4 |
|  |  | ENST00000396509 |  |
|  |  | ENST00000558145 |  |
|  |  | ENST00000544523 |  |
|  |  | ENST00000560138 |  |
|  |  | ENST00000559454 |  |
| GIPC3 | ENSG00000179855 | ENST00000322315 | (GCG)4 |
| GMFB | ENSG00000197045 | ENST00000553333 | (TG)4 |
| GNAS | ENSG00000087460 | ENST00000371100 | (TAA)4 |
| GPR32 | ENSG00000142511 | ENST00000270590 | (AAT)4 |
| GPR42 | ENSG00000126251 | ENST00000454971 | (CT)4 |
| GRAMD4 | ENSG00000075240 | ENST00000447351 | (TG)4 |
|  |  | ENST00000431155 |  |
|  |  | ENST00000406902 |  |
| GRHL2 | ENSG00000083307 | ENST00000395927 | (CT)4 |
| GRIP1 | ENSG00000155974 | ENST00000398016 | (CTG)4 |
| HIST1H3G | ENSG00000273983 | ENST00000614378 | (TA)4 |
| HK1 | ENSG00000156515 | ENST00000359426 | (GAG)4 |
| HRASLS | ENSG00000127252 | ENST00000264735 | (CGGG)4 |
| HSD17B4 | ENSG00000133835 | ENST00000509514 | (TTAT)4 |
| HYI | ENSG00000178922 | ENST00000372425 | (GCCCGCC)4 |
|  |  | ENST00000583037 |  |
|  |  | ENST00000372432 |  |
| IL31RA | ENSG00000164509 | ENST00000396836 | (TG)4 |
|  |  | ENST00000447346 |  |
|  |  | ENST00000359040 |  |
| INPP5K | ENSG00000132376 | ENST00000449479 | (TC)4 |
|  |  | ENST00000498390 |  |
| ISG15 | ENSG00000187608 | ENST00000624697 | (TG)4 |
|  |  | ENST00000624652 |  |
| KCNE1 | ENSG00000180509 | ENST00000432085 | (GT)4 |
|  |  | ENST00000621601 |  |
|  |  | ENST00000337385 |  |
|  |  | ENST00000611936 |  |
|  |  | ENST00000399289 |  |
|  |  | ENST00000399286 |  |
|  |  | ENST00000416357 |  |
|  |  | ENST00000399284 |  |
| KCNF1 | ENSG00000162975 | ENST00000295082 | (GCG)4 |
| KNTC1 | ENSG00000184445 | ENST00000436959 | (TC)4 |
| LAMA5 | ENSG00000130702 | ENST00000252999 | (CG)4 |
| LANCL1 | ENSG00000115365 | ENST00000443314 | (CGC)4 |
|  |  | ENST00000441020 |  |
|  |  | ENST00000450366 |  |
|  |  | ENST00000233714 |  |
|  |  | ENST00000431941 |  |
|  |  | ENST00000448951 |  |
| LCE1B | ENSG00000196734 | ENST00000360090 | (CT)4 |
| MAD1L1 | ENSG00000002822 | ENST00000437877 | (TG)4 |
|  |  |  |  |
| MAGEB16 | ENSG00000189023 | ENST00000399988 | (TC)4 |
|  |  | ENST00000399987 |  |
|  |  | ENST00000399989 |  |
| MAGEB3 | ENSG00000198798 | ENST00000361644 | (CT)4 |
| MATK | ENSG00000007264 | ENST00000395045 | (AG)4 |
|  |  | ENST00000590980 |  |
| MCM3 | ENSG00000112118 | ENST00000616552 | (GAA)4 |
|  |  | ENST00000419835 |  |
| MEP1B | ENSG00000141434 | ENST00000581184 | (GTT)4 |
| MERTK | ENSG00000153208 | ENST00000616902 | (TG)4 |
| MTPAP | ENSG00000107951 | ENST00000417581 | (GT)4 |
| MYH2 | ENSG00000125414 | ENST00000578017 | (GTT)4 |
| NAP1L4 | ENSG00000205531 | ENST00000399614 | (TG)4 |
| NCKAP1L | ENSG00000123338 | ENST00000545638 | (GAG)4 |
| NEDD9 | ENSG00000111859 | ENST00000508546 | (AT)4 |
| NOVA2 | ENSG00000104967 | ENST00000596784 | (CT)4 |
| NPAS1 | ENSG00000130751 | ENST00000602189 | (GC)4 |
|  |  | ENST00000439365 |  |
|  |  | ENST00000594670 |  |
| NQO1 | ENSG00000181019 | ENST00000564043 | (GT)4 |
| NUBPL | ENSG00000151413 | ENST00000551314 | (ATT)4 |
| OCEL1 | ENSG00000099330 | ENST00000597836 | (CG)4 |
| ONECUT3 | ENSG00000205922 | ENST00000382349 | (GC)4 |
| OR10C1 | ENSG00000206474 | ENST00000444197 | (AG)4 |
| OR14A16 | ENSG00000196772 | ENST00000641093 | (TA)4 |
| OR4D1 | ENSG00000141194 | ENST00000641449 | (GTTT)4 |
| OR52K1 | ENSG00000196778 | ENST00000641528 | (TA)4 |
| OR6C6 | ENSG00000188324 | ENST00000358433 |  |
| OR9A4 | ENSG00000258083 | ENST00000641559 | (TC)4 |
| OSGIN1 | ENSG00000140961 | ENST00000343939 |  |
| OVOL3 | ENSG00000105261 | ENST00000633214 | (TG)4 |
| PABPC1L | ENSG00000101104 | ENST00000372824 | (CT)4 |
|  |  | ENST00000372819 |  |
|  |  | ENST00000217075 |  |
| PAGE2B | ENSG00000238269 | ENST00000374974 | (AT)4 |
| PAMR1 | ENSG00000149090 | ENST00000621476 | (AACA)4 |
|  |  | ENST00000527605 |  |
| PARG | ENSG00000227345 | ENST00000402038 | (TC)4 |
|  |  | ENST00000616448 |  |
| PBXIP1 | ENSG00000163346 | ENST00000368465 | (TG)4 |
| PCDHA9 | ENSG00000204961 | ENST00000532602 | (CT)4 |
|  |  | ENST00000378122 |  |
| PCDHB5 | ENSG00000113209 | ENST00000623915 | (AG)4 |
| PCMT1 | ENSG00000120265 | ENST00000495487 | (TC)4 |
| PDE4D | ENSG00000113448 | ENST00000340635 | (GCG)4 |
| PDIA6 | ENSG00000143870 | ENST00000381611 | (TC)4 |
| PEBP4 | ENSG00000134020 | ENST00000522278 | (CG)4 |
| PEG10 | ENSG00000242265 | ENST00000482108 | (CCT)4 |
|  |  | ENST00000613043 |  |
|  |  | ENST00000617526 |  |
| PGAP3 | ENSG00000161395 | ENST00000584856 | (CT)4 |
| PIGZ | ENSG00000119227 | ENST00000412723 | (TAT)4 |
|  |  | ENST00000443835 |  |
| PKD1 | ENSG00000008710 | ENST00000262304 | (CG)4 |
|  |  | ENST00000423118 |  |
| PKD2 | ENSG00000118762 | ENST00000508588 | (TC)4 |
| PLCZ1 | ENSG00000139151 | ENST00000534932 | (AT)4 |
| POLD4 | ENSG00000175482 | ENST00000529704 | (AC)4 |
|  |  | ENST00000530584 |  |
|  |  | ENST00000532830 |  |
|  |  | ENST00000531239 |  |
| POLR2J | ENSG00000005075 | ENST00000292614 | (GCG)4 |
| PRAMEF4 | ENSG00000243073 | ENST00000235349 | (GA)4 |
| PROCA1 | ENSG00000167525 | ENST00000415329 | (GAG)4 |
| PRR5 | ENSG00000186654 | ENST00000403581 | (TAA)4 |
| PTPN2 | ENSG00000175354 | ENST00000646492 | (CT)4 |
|  |  |  |  |
|  |  | ENST00000645191 |  |
|  |  |  |  |
|  |  | ENST00000592059 |  |
|  |  |  |  |
| PTPRN2 | ENSG00000155093 | ENST00000389413 | (GC)4 |
|  |  | ENST00000409483 |  |
| RAB34 | ENSG00000109113 | ENST00000430132 | (TC)4 |
| RAP1GAP | ENSG00000076864 | ENST00000374761 | (GC)4 |
| RBM6 | ENSG00000004534 | ENST00000421682 | (TAA)4 |
| REXO2 | ENSG00000076043 | ENST00000544827 | (GT)4 |
| RGS11 | ENSG00000076344 | ENST00000316163 | (CCG)4 |
| RGS7 | ENSG00000182901 | ENST00000366563 | (ATG)4 |
|  |  | ENST00000366564 |  |
|  |  | ENST00000366565 |  |
| RIC8B | ENSG00000111785 | ENST00000549643 | (CT)4 |
| RNASE2 | ENSG00000169385 | ENST00000304625 | (GT)4 |
| ROCK2 | ENSG00000134318 | ENST00000401753 |  |
| RRAGD | ENSG00000025039 | ENST00000359203 |  |
| SECISBP2L | ENSG00000138593 | ENST00000380927 | (TC)4 |
| SGK1 | ENSG00000118515 | ENST00000367857 | (GT)4 |
| SGMS1 | ENSG00000198964 | ENST00000429490 | (AG)4 |
|  |  | ENST00000361781 |  |
|  |  | ENST00000361543 |  |
|  |  | ENST00000619438 |  |
| SIRT6 | ENSG00000077463 | ENST00000594279 | (TC)4 |
| SLC30A3 | ENSG00000115194 | ENST00000233535 | (CT)4 |
| SLC4A8 | ENSG00000050438 | ENST00000535225 | (GTT)4 |
|  |  | ENST00000358657 |  |
|  |  | ENST00000514353 |  |
| SLC9A6 | ENSG00000198689 | ENST00000636347 | (CGG)4 |
|  |  | ENST00000636092 |  |
|  |  | ENST00000637195 |  |
|  |  | ENST00000637234 |  |
|  |  | ENST00000637581 |  |
|  |  | ENST00000370701 |  |
|  |  | ENST00000627534 |  |
|  |  | ENST00000630721 |  |
| SNRPA1 | ENSG00000131876 | ENST00000560496 | (TG)4 |
| SPAG5 | ENSG00000076382 | ENST00000321765 | (CG)4 |
| SPEG | ENSG00000072195 | ENST00000435853 | (GT)4 |
| SPIN4 | ENSG00000186767 | ENST00000374884 | (TA)4 |
| SPRR2A | ENSG00000241794 | ENST00000392653 | (TC)4 |
| SPRR2D | ENSG00000163216 | ENST00000360379 |  |
|  |  | ENST00000368758 |  |
|  |  | ENST00000368757 |  |
| SPRR2E | ENSG00000203785 | ENST00000368750 |  |
| SPRR2F | ENSG00000244094 | ENST00000468739 |  |
| ST20 | ENSG00000180953 | ENST00000562759 | (TTAT)4 |
|  |  | ENST00000478497 |  |
|  |  | ENST00000485386 |  |
| STIM2 | ENSG00000109689 | ENST00000467087 | (GCG)4 |
|  |  | ENST00000467011 |  |
|  |  | ENST00000465503 |  |
| SULT1A1 | ENSG00000196502 | ENST00000350842 | (GC)4 |
| SYBU | ENSG00000147642 | ENST00000533394 | (TC)4 |
| TAB2 | ENSG00000055208 | ENST00000606202 |  |
| TAOK3 | ENSG00000135090 | ENST00000536979 | (GT)4 |
| TBL1Y | ENSG00000092377 | ENST00000383032 | (CA)4 |
|  |  | ENST00000355162 |  |
|  |  | ENST00000346432 |  |
| TCFL5 | ENSG00000101190 | ENST00000335351 | (CGCCTCC)4 |
| TINAGL1 | ENSG00000142910 | ENST00000537531 | (AG)4 |
| TMEM176A | ENSG00000002933 | ENST00000461345 | (TC)4 |
|  |  | ENST00000468689 |  |
| TMEM9B | ENSG00000175348 | ENST00000534025 | (GCCTGA)4 |
| TNKS | ENSG00000173273 | ENST00000518281 | (TA)4 |
| TOR3A | ENSG00000186283 | ENST00000447595 | (GA)4 |
| TP53I13 | ENSG00000167543 | ENST00000583940 | (AG)4 |
|  |  | ENST00000580183 |  |
|  |  | ENST00000578749 |  |
|  |  | ENST00000582829 |  |
|  |  | ENST00000581411 |  |
| TSHZ3 | ENSG00000121297 | ENST00000558569 | (GGC)4 |
| VARS2 | ENSG00000137411 | ENST00000541562 | (GC)4 |
| VN1R2 | ENSG00000196131 | ENST00000341702 | (TC)4 |
| WDR81 | ENSG00000167716 | ENST00000309182 | (GAG)4 |
|  |  | ENST00000418841 | (TG)4 |
| WDR82 | ENSG00000164091 | ENST00000469000 | (TA)4 |
|  |  | ENST00000463624 |  |
| WDR89 | ENSG00000140006 | ENST00000394942 | (ATT)4 |
|  |  | ENST00000267522 |  |
|  |  | ENST00000554717 |  |
| WDR90 | ENSG00000161996 | ENST00000547944 | (AC)4 |
|  |  | ENST00000315764 |  |
| XAGE1A | ENSG00000204379 | ENST00000375602 |  |
|  |  | ENST00000375600 |  |
| XAGE1B | ENSG00000204382 | ENST00000375616 |  |
|  |  | ENST00000375613 |  |
|  |  | ENST00000518075 |  |
| XPNPEP1 | ENSG00000108039 | ENST00000502935 | (GCCC)4 |
|  |  | ENST00000322238 |  |
| ZAP70 | ENSG00000115085 | ENST00000451498 | (TG)4 |
| ZDHHC18 | ENSG00000204160 | ENST00000374142 | (GC)4 |
| ZFP36L1 | ENSG00000185650 | ENST00000557022 | (TC)4 |
| ZNF138 | ENSG00000197008 | ENST00000437743 | (TG)4 |
| ZNF254 | ENSG00000213096 | ENST00000613065 |  |
| ZNF638 | ENSG00000075292 | ENST00000466975 | (CG)4 |
| ZNF676 | ENSG00000196109 | ENST00000397121 | (GT)4 |
| ZNF707 | ENSG00000181135 | ENST00000526315 | (TG)4 |
| ZNF717 | ENSG00000227124 | ENST00000477374 | (TC)4 |
|  |  | ENST00000468296 |  |
| ZNF862 | ENSG00000106479 | ENST00000460379 | (TG)4 |
| ZSCAN10 | ENSG00000130182 | ENST00000538082 | (GGA)4 |
| MARCH6 | ENSG00000145495 | ENST00000510792 | (TA)3 |
|  |  |  | (GA)3 |
| SEPT7 | ENSG00000122545 | ENST00000432293 | (GAA)3 |
| SEPT9 | ENSG00000184640 | ENST00000591934 | (TCT)3 |
| AAMP | ENSG00000127837 | ENST00000447885 | (TC)3 |
| AANAT | ENSG00000129673 | ENST00000250615 | (GA)3 |
| ABCA4 | ENSG00000198691 | ENST00000536513 |  |
| ABCB9 | ENSG00000150967 | ENST00000540971 | (GT)3 |
|  |  | ENST00000536976 | (GA)3 |
|  |  | ENST00000541424 |  |
| ABCC10 | ENSG00000124574 | ENST00000372515 | (CTG)3 |
| ABCF1 | ENSG00000204574 | ENST00000468958 | (GAT)3 |
| ABLIM2 | ENSG00000163995 | ENST00000514025 | (GC)3 |
|  |  | ENST00000510277 |  |
| ACBD5 | ENSG00000107897 | ENST00000375897 | (CCT)3 |
|  |  | ENST00000375888 |  |
| ACE | ENSG00000159640 | ENST00000582627 | (GC)3 |
| ACOT7 | ENSG00000097021 | ENST00000608083 | (CCG)3 |
| ACRBP | ENSG00000111644 | ENST00000546114 | (TC)3 |
| ACSBG1 | ENSG00000103740 | ENST00000560817 | (AT)3 |
| ACSS3 | ENSG00000111058 | ENST00000549175 |  |
| ACTN2 | ENSG00000077522 | ENST00000546208 | (GTGT)3 |
| ACTR3C | ENSG00000106526 | ENST00000252071 | (AG)3 |
|  |  | ENST00000477367 |  |
| ADAMDEC1 | ENSG00000134028 | ENST00000522298 | (AT)3 |
| ADAMTSL1 | ENSG00000178031 | ENST00000636209 | (GCC)3 |
| ADAP2 | ENSG00000184060 | ENST00000581548 | (GA)3 |
| ADH7 | ENSG00000196344 | ENST00000437033 | (TA)3 |
|  |  | ENST00000209665 |  |
| ADPRHL1 | ENSG00000153531 | ENST00000612156 | (GAG)3 |
|  |  | ENST00000375418 |  |
| ADSSL1 | ENSG00000185100 | ENST00000556623 | (TG)3 |
| AES | ENSG00000104964 | ENST00000221561 | (CAC)3 |
|  |  |  | (CA)3 |
| AFF1 | ENSG00000172493 | ENST00000511442 | (TA)3 |
| AGAP5 | ENSG00000172650 | ENST00000581191 | (GT)3 |
|  |  | ENST00000443782 |  |
|  |  | ENST00000374094 |  |
| AGTR1 | ENSG00000144891 | ENST00000497524 | (GTTT)3 |
|  |  | ENST00000349243 |  |
|  |  | ENST00000404754 |  |
|  |  | ENST00000475347 |  |
|  |  | ENST00000474935 |  |
|  |  | ENST00000461609 |  |
| AIG1 | ENSG00000146416 | ENST00000458219 | (TG)3 |
| AK5 | ENSG00000154027 | ENST00000344720 | (TTAA)3 |
|  |  |  | (ATTAA)3 |
|  |  | ENST00000478407 | (TTAA)3 |
|  |  |  | (ATTAA)3 |
| AKAP2 | ENSG00000241978 | ENST00000434623 | (GC)3 |
| AKAP7 | ENSG00000118507 | ENST00000537868 | (TGAT)3 |
|  |  | ENST00000263050 |  |
| AKNA | ENSG00000106948 | ENST00000374075 | (AG)3 |
| AKR1B10 | ENSG00000198074 | ENST00000359579 |  |
| AKR1C3 | ENSG00000196139 | ENST00000380554 | (AGC)3 |
| AKR7L | ENSG00000211454 | ENST00000420396 | (GC)3 |
| ALG3 | ENSG00000214160 | ENST00000445626 | (GA)3 |
| ALG5 | ENSG00000120697 | ENST00000443765 | (CG)3 |
|  |  | ENST00000239891 |  |
| ANGEL1 | ENSG00000013523 | ENST00000555079 | (AG)3 |
| ANGPTL3 | ENSG00000132855 | ENST00000371129 | (AT)3 |
| ANK3 | ENSG00000151150 | ENST00000280772 | (GT)3 |
|  |  | ENST00000373827 | (GA)3 |
| ANKFN1 | ENSG00000153930 | ENST00000635860 | (GT)3 |
| ANKZF1 | ENSG00000163516 | ENST00000453432 | (AG)3 |
| ANLN | ENSG00000011426 | ENST00000424865 | (AT)3 |
|  |  | ENST00000418118 |  |
| ANXA3 | ENSG00000138772 | ENST00000512884 | (CT)3 |
|  |  | ENST00000503570 |  |
| AP1S3 | ENSG00000152056 | ENST00000444408 | (AT)3 |
| APBB2 | ENSG00000163697 | ENST00000509446 | (TA)3 |
|  |  | ENST00000503264 |  |
| APEH | ENSG00000164062 | ENST00000449966 | (TC)3 |
| APLP1 | ENSG00000105290 | ENST00000589743 | (GAAA)3 |
|  |  | ENST00000592316 |  |
| APOA2 | ENSG00000158874 | ENST00000468465 | (TG)3 |
|  |  | ENST00000463812 |  |
| APOC2 | ENSG00000234906 | ENST00000252490 | (CA)3 |
|  |  | ENST00000592257 |  |
|  |  | ENST00000591597 |  |
|  |  | ENST00000590360 |  |
| APOD | ENSG00000189058 | ENST00000421243 | (GTTT)3 |
| APOL4 | ENSG00000100336 | ENST00000352371 | (AGG)3 |
| APOM | ENSG00000204444 | ENST00000375918 | (TC)3 |
|  |  | ENST00000375920 |  |
| AQP7 | ENSG00000165269 | ENST00000439678 | (AC)3 |
|  |  |  |  |
|  |  | ENST00000447660 |  |
|  |  |  |  |
| ARAP1 | ENSG00000186635 | ENST00000393605 | (TG)3 |
|  |  |  |  |
| ARAP3 | ENSG00000120318 | ENST00000239440 | (AG)3 |
|  |  | ENST00000504448 |  |
| ARCN1 | ENSG00000095139 | ENST00000359415 | (TGTTC)3 |
| ARHGAP5 | ENSG00000100852 | ENST00000396582 | (GC)3 |
|  |  |  | (GGA)3 |
| ARHGAP6 | ENSG00000047648 | ENST00000337414 | (AGG)3 |
|  |  | ENST00000380718 |  |
| ARID1A | ENSG00000117713 | ENST00000430799 | (TA)3 |
|  |  | ENST00000637465 |  |
|  |  | ENST00000524572 |  |
|  |  | ENST00000374152 |  |
|  |  | ENST00000615191 |  |
| ARID1B | ENSG00000049618 | ENST00000346085 | (GC)3 |
|  |  |  | (CAC)3 |
| ARID3B | ENSG00000179361 | ENST00000566147 | (TGA)3 |
| ARL1 | ENSG00000120805 | ENST00000551688 | (GA)3 |
| ARL16 | ENSG00000214087 | ENST00000573392 | (TTAA)3 |
|  |  | ENST00000574938 |  |
|  |  | ENST00000573715 |  |
| ARMC9 | ENSG00000135931 | ENST00000446447 | (TC)3 |
| ARPC4 | ENSG00000241553 | ENST00000498623 | (CT)3 |
|  |  | ENST00000485273 |  |
| ARR3 | ENSG00000120500 | ENST00000480877 |  |
|  |  |  |  |
| ASAH2B | ENSG00000204147 | ENST00000374007 |  |
|  |  | ENST00000647317 |  |
| ASB4 | ENSG00000005981 | ENST00000428113 | (TC)3 |
| ASCL4 | ENSG00000187855 | ENST00000342331 | (TCT)3 |
| ASGR2 | ENSG00000161944 | ENST00000355035 | (CAGCTC)3 |
|  |  | ENST00000254850 |  |
| ASH2L | ENSG00000129691 | ENST00000343823 | (CA)3 |
| ASNS | ENSG00000070669 | ENST00000422745 | (TG)3 |
|  |  | ENST00000444334 |  |
|  |  | ENST00000453600 |  |
| ASRGL1 | ENSG00000162174 | ENST00000526096 | (CGG)3 |
| ASTN2 | ENSG00000148219 | ENST00000288520 | (AG)3 |
| ASXL1 | ENSG00000171456 | ENST00000643168 | (AT)3 |
|  |  |  | (GGAT)3 |
|  |  | ENST00000645688 | (AT)3 |
|  |  |  | (GGAT)3 |
| ATAD2 | ENSG00000156802 | ENST00000521903 | (ACG)3 |
| ATF7 | ENSG00000170653 | ENST00000548446 | (CT)3 |
|  |  | ENST00000420353 |  |
|  |  | ENST00000456903 |  |
|  |  | ENST00000588232 | (GCT)3 |
|  |  | ENST00000551480 |  |
|  |  | ENST00000588078 | (CT)3 |
|  |  | ENST00000548118 |  |
|  |  | ENST00000591397 |  |
| ATG3 | ENSG00000144848 | ENST00000492886 |  |
| ATG4A | ENSG00000101844 | ENST00000457035 | (TG)3 |
| ATG9A | ENSG00000198925 | ENST00000431715 | (CT)3 |
| ATL1 | ENSG00000198513 | ENST00000557735 | (TC)3 |
| ATOX1 | ENSG00000177556 | ENST00000522314 | (TG)3 |
|  |  | ENST00000520382 |  |
| ATP13A4 | ENSG00000127249 | ENST00000400270 | (TC)3 |
|  |  |  | (CT)3 |
| ATP6V0A2 | ENSG00000185344 | ENST00000504192 | (GA)3 |
| ATP6V0B | ENSG00000117410 | ENST00000472505 | (TGG)3 |
|  |  | ENST00000236067 |  |
| ATP6V1A | ENSG00000114573 | ENST00000496747 | (GA)3 |
| ATP8B1 | ENSG00000081923 | ENST00000588255 | (CT)3 |
| AUTS2 | ENSG00000158321 | ENST00000615871 |  |
|  |  | ENST00000611706 |  |
| BATF2 | ENSG00000168062 | ENST00000435842 | (GCA)3 |
| BBS1 | ENSG00000174483 | ENST00000524705 | (GCT)3 |
| BBS12 | ENSG00000181004 | ENST00000314218 | (TA)3 |
|  |  | ENST00000542236 |  |
| BBS5 | ENSG00000163093 | ENST00000295240 | (GA)3 |
|  |  | ENST00000392663 |  |
| BCAP29 | ENSG00000075790 | ENST00000465919 |  |
| BCAR1 | ENSG00000050820 | ENST00000393422 | (GCC)3 |
| BCAS3 | ENSG00000141376 | ENST00000586705 | (AT)3 |
|  |  | ENST00000585744 |  |
|  |  | ENST00000588874 |  |
|  |  | ENST00000590128 |  |
|  |  | ENST00000586484 |  |
| BCL2L13 | ENSG00000099968 | ENST00000611738 | (GA)3 |
|  |  | ENST00000618481 |  |
| BCR | ENSG00000186716 | ENST00000305877 | (CCG)3 |
|  |  | ENST00000359540 |  |
| BEST1 | ENSG00000167995 | ENST00000449131 | (CA)3 |
|  |  |  | (CCACCC)3 |
| BHLHE23 | ENSG00000125533 | ENST00000612929 | (GCAGCG)3 |
| BIN3 | ENSG00000147439 | ENST00000519513 | (GA)3 |
| BMP8A | ENSG00000183682 | ENST00000331593 | (CGG)3 |
| BPY2 | ENSG00000183753 | ENST00000602732 | (CT)3 |
|  |  | ENST00000331070 |  |
| BPY2B | ENSG00000183795 | ENST00000602770 |  |
|  |  | ENST00000382392 |  |
| BPY2C | ENSG00000185894 | ENST00000382287 |  |
| BRAP | ENSG00000089234 | ENST00000327551 | (AG)3 |
| BRCA1 | ENSG00000012048 | ENST00000493795 | (TTCT)3 |
|  |  | ENST00000493919 |  |
|  |  | ENST00000644555 |  |
| BRD2 | ENSG00000204256 | ENST00000449085 | (AGC)3 |
| BRD9 | ENSG00000028310 | ENST00000467963 | (CCG)3 |
|  |  | ENST00000489093 | (CAGA)3 |
|  |  |  | (CT)3 |
| BRSK2 | ENSG00000174672 | ENST00000544817 | (TG)3 |
| BSN | ENSG00000164061 | ENST00000296452 | (GCG)3 |
| BUB1 | ENSG00000169679 | ENST00000447014 | (CT)3 |
|  |  | ENST00000420328 |  |
| BZW2 | ENSG00000136261 | ENST00000452975 | (CTTTTT)3 |
| C10orf55 | ENSG00000222047 | ENST00000412307 | (AC)3 |
| C11orf65 | ENSG00000166323 | ENST00000525729 | (TAAA)3 |
|  |  | ENST00000615746 |  |
|  |  | ENST00000393084 |  |
| C11orf71 | ENSG00000180425 | ENST00000623205 | (TG)3 |
| C12orf10 | ENSG00000139637 | ENST00000548632 | (GCT)3 |
|  |  | ENST00000549488 | (GA)3 |
|  |  |  | (GT)3 |
| C12orf4 | ENSG00000047621 | ENST00000542080 | (TA)3 |
| C12orf42 | ENSG00000179088 | ENST00000548048 | (GT)3 |
| C14orf132 | ENSG00000227051 | ENST00000553764 | (ACAT)3 |
| C15orf59 | ENSG00000205363 | ENST00000559817 | (GTG)3 |
| C16orf74 | ENSG00000154102 | ENST00000602675 | (CAT)3 |
|  |  | ENST00000602766 |  |
| C17orf100 | ENSG00000256806 | ENST00000542475 | (CT)3 |
| C17orf49 | ENSG00000258315 | ENST00000546495 | (GC)3 |
|  |  | ENST00000546760 |  |
|  |  | ENST00000552402 |  |
|  |  | ENST00000439424 |  |
|  |  | ENST00000552775 | (GA)3 |
| C17orf64 | ENSG00000141371 | ENST00000461535 | (CCT)3 |
|  |  | ENST00000474834 |  |
| C19orf24 | ENSG00000228300 | ENST00000409293 | (CG)3 |
| C19orf54 | ENSG00000188493 | ENST00000470681 | (CT)3 |
|  |  | ENST00000598485 |  |
| C1orf116 | ENSG00000182795 | ENST00000359470 | (TG)3 |
| C1orf185 | ENSG00000204006 | ENST00000467127 | (GA)3 |
| C1orf198 | ENSG00000119280 | ENST00000470540 | (GGC)3 |
|  |  | ENST00000427697 | (AGG)3 |
|  |  | ENST00000522201 | (GGC)3 |
| C1orf226 | ENSG00000239887 | ENST00000420220 | (CT)3 |
| C1QB | ENSG00000173369 | ENST00000510260 | (GA)3 |
|  |  | ENST00000509305 |  |
|  |  | ENST00000432749 |  |
| C20orf173 | ENSG00000125975 | ENST00000246199 | (AG)3 |
| C22orf42 | ENSG00000205856 | ENST00000382097 | (AGG)3 |
|  |  |  | (GT)3 |
| C2orf81 | ENSG00000284308 | ENST00000640868 | (CT)3 |
|  |  | ENST00000640331 |  |
| C3orf20 | ENSG00000131379 | ENST00000435614 | (CAC)3 |
|  |  |  | (TC)3 |
|  |  | ENST00000412910 | (CAC)3 |
|  |  |  | (TC)3 |
| C3orf58 | ENSG00000181744 | ENST00000315691 | (GC)3 |
| C6orf223 | ENSG00000181577 | ENST00000442114 | (TC)3 |
| C8orf34 | ENSG00000165084 | ENST00000518698 | (CTG)3 |
|  |  | ENST00000337103 | (TA)3 |
|  |  |  | (TG)3 |
| C8orf58 | ENSG00000241852 | ENST00000409586 | (GGCCGG)3 |
|  |  | ENST00000614574 |  |
|  |  | ENST00000289989 |  |
|  |  | ENST00000615223 | (AG)3 |
| C9orf40 | ENSG00000135045 | ENST00000376854 | (TC)3 |
| C9orf50 | ENSG00000179058 | ENST00000372478 | (CG)3 |
| C9orf92 | ENSG00000205549 | ENST00000380685 | (TTGT)3 |
| CAB39L | ENSG00000102547 | ENST00000409130 | (CT)3 |
| CABP1 | ENSG00000157782 | ENST00000453000 | (TG)3 |
| CABYR | ENSG00000154040 | ENST00000627314 | (GTT)3 |
|  |  | ENST00000327201 |  |
|  |  | ENST00000585037 |  |
|  |  | ENST00000577705 |  |
| CACNA1C | ENSG00000151067 | ENST00000496818 | (CA)3 |
| CACNA1D | ENSG00000157388 | ENST00000637424 | (AG)3 |
|  |  | ENST00000638120 | (GA)3 |
|  |  |  | (CAT)3 |
| CACNA2D4 | ENSG00000151062 | ENST00000538450 | (TC)3 |
|  |  | ENST00000538027 |  |
| CACNB2 | ENSG00000165995 | ENST00000324631 | (GAG)3 |
| CACNB4 | ENSG00000182389 | ENST00000637284 | (TTC)3 |
|  |  | ENST00000637514 |  |
| CAPN12 | ENSG00000182472 | ENST00000328867 | (AG)3 |
| CARD10 | ENSG00000100065 | ENST00000437756 | (TC)3 |
| CARD6 | ENSG00000132357 | ENST00000254691 | (AT)3 |
| CASP8 | ENSG00000064012 | ENST00000450491 |  |
| CCBE1 | ENSG00000183287 | ENST00000589419 | (TC)3 |
| CCDC116 | ENSG00000161180 | ENST00000425975 | (CTGC)3 |
| CCDC149 | ENSG00000181982 | ENST00000502801 | (CGG)3 |
| CCDC63 | ENSG00000173093 | ENST00000545036 | (GAA)3 |
| CCDC8 | ENSG00000169515 | ENST00000307522 | (AGG)3 |
| CCDC88B | ENSG00000168071 | ENST00000359902 | (TC)3 |
|  |  |  | (CT)3 |
| CCL14 | ENSG00000276409 | ENST00000614009 | (TGA)3 |
| CCNI | ENSG00000118816 | ENST00000507788 | (GCGCCC)3 |
|  |  |  | (GC)3 |
|  |  |  | (CG)3 |
| CCS | ENSG00000173992 | ENST00000534763 | (CA)3 |
| CD226 | ENSG00000150637 | ENST00000581982 | (CTT)3 |
|  |  | ENST00000577287 |  |
| CD37 | ENSG00000104894 | ENST00000598095 | (TCA)3 |
|  |  | ENST00000426897 |  |
|  |  | ENST00000597602 |  |
| CD70 | ENSG00000125726 | ENST00000423145 | (CCTT)3 |
|  |  | ENST00000245903 |  |
|  |  | ENST00000597430 |  |
| CD81 | ENSG00000110651 | ENST00000475945 | (GT)3 |
|  |  | ENST00000530648 |  |
|  |  | ENST00000492627 |  |
|  |  | ENST00000526072 |  |
| CDC42BPA | ENSG00000143776 | ENST00000366766 | (GAA)3 |
| CDCA2 | ENSG00000184661 | ENST00000380665 | (AG)3 |
| CDH11 | ENSG00000140937 | ENST00000566827 | (TC)3 |
| CDH2 | ENSG00000170558 | ENST00000418492 | (GA)3 |
| CDH4 | ENSG00000179242 | ENST00000614565 | (GCG)3 |
|  |  | ENST00000611855 | (GT)3 |
|  |  | ENST00000543233 | (CT)3 |
| CDH6 | ENSG00000113361 | ENST00000514738 |  |
|  |  |  |  |
| CDHR3 | ENSG00000128536 | ENST00000478080 | (TGCA)3 |
| CDK5RAP3 | ENSG00000108465 | ENST00000579632 | (CT)3 |
|  |  | ENST00000536708 |  |
| CDKN2A | ENSG00000147889 | ENST00000578845 | (TG)3 |
|  |  |  | (TC)3 |
|  |  | ENST00000498628 | (TG)3 |
|  |  |  | (TC)3 |
|  |  | ENST00000494262 | (TG)3 |
|  |  |  | (TC)3 |
|  |  | ENST00000479692 | (TG)3 |
|  |  |  | (TC)3 |
|  |  | ENST00000497750 | (TG)3 |
|  |  |  | (TC)3 |
| CDON | ENSG00000064309 | ENST00000531738 | (GT)3 |
| CDR1 | ENSG00000184258 | ENST00000370532 | (AT)3 |
| CDYL | ENSG00000153046 | ENST00000440139 | (GAG)3 |
| CEACAM1 | ENSG00000079385 | ENST00000161559 | (AG)3 |
|  |  | ENST00000403444 |  |
|  |  | ENST00000599389 |  |
|  |  | ENST00000600172 | (CT)3 |
|  |  |  |  |
|  |  | ENST00000471298 |  |
|  |  |  |  |
| CEACAM21 | ENSG00000007129 | ENST00000407170 | (TC)3 |
| CELF2 | ENSG00000048740 | ENST00000637215 | (CCT)3 |
|  |  | ENST00000636488 |  |
| CELF6 | ENSG00000140488 | ENST00000395258 | (GA)3 |
| CEND1 | ENSG00000184524 | ENST00000330106 | (GC)3 |
| CEP120 | ENSG00000168944 | ENST00000306481 | (TA)3 |
|  |  | ENST00000508442 |  |
| CEP164 | ENSG00000110274 | ENST00000533153 | (TC)3 |
|  |  | ENST00000525416 |  |
| CERCAM | ENSG00000167123 | ENST00000613052 | (TGTC)3 |
| CES4A | ENSG00000172824 | ENST00000540947 | (CA)3 |
|  |  | ENST00000540579 | (GCC)3 |
|  |  | ENST00000535696 |  |
| CES5A | ENSG00000159398 | ENST00000541580 | (AG)3 |
| CFB | ENSG00000243649 | ENST00000475617 | (AC)3 |
|  |  |  | (TC)3 |
|  |  | ENST00000425368 | (AC)3 |
|  |  |  | (TC)3 |
| CFHR1 | ENSG00000244414 | ENST00000320493 | (TA)3 |
|  |  | ENST00000367424 |  |
| CFHR2 | ENSG00000080910 | ENST00000476712 |  |
|  |  | ENST00000367415 |  |
| CFL1 | ENSG00000172757 | ENST00000534769 | (TC)3 |
| CGB2 | ENSG00000104818 | ENST00000474913 | (CTG)3 |
|  |  |  | (TGC)3 |
| CGNL1 | ENSG00000128849 | ENST00000281282 | (TC)3 |
| CHD2 | ENSG00000173575 | ENST00000625990 | (ATA)3 |
| CHGB | ENSG00000089199 | ENST00000455042 | (CA)3 |
| CHMP1B | ENSG00000255112 | ENST00000526991 | (GC)3 |
| CHN1 | ENSG00000128656 | ENST00000409089 | (GT)3 |
|  |  | ENST00000444394 |  |
| CHRFAM7A | ENSG00000166664 | ENST00000397827 | (CA)3 |
|  |  | ENST00000401522 |  |
| CHRM5 | ENSG00000184984 | ENST00000560035 | (CT)3 |
|  |  | ENST00000383263 |  |
| CHRNA7 | ENSG00000175344 | ENST00000638106 | (CA)3 |
|  |  | ENST00000306901 | (GC)3 |
|  |  |  |  |
|  |  | ENST00000454250 |  |
|  |  |  |  |
|  |  | ENST00000637552 |  |
| CHST10 | ENSG00000115526 | ENST00000448989 | (GA)3 |
| CKAP4 | ENSG00000136026 | ENST00000553039 | (AG)3 |
| CLCN7 | ENSG00000103249 | ENST00000569851 | (GCG)3 |
| CLDN9 | ENSG00000213937 | ENST00000445369 | (AC)3 |
| CLINT1 | ENSG00000113282 | ENST00000523094 | (TG)3 |
|  |  | ENST00000530742 |  |
| CLIP4 | ENSG00000115295 | ENST00000401617 | (AG)3 |
|  |  | ENST00000438819 |  |
| CLMN | ENSG00000165959 | ENST00000556441 | (TG)3 |
| CLUL1 | ENSG00000079101 | ENST00000579912 | (TTCTT)3 |
|  |  | ENST00000540035 |  |
|  |  | ENST00000581619 | (TC)3 |
|  |  |  | (CT)3 |
| CNN1 | ENSG00000130176 | ENST00000585869 | (AG)3 |
|  |  |  |  |
| CNN2 | ENSG00000064666 | ENST00000348419 | (TCCCG)3 |
| CNOT8 | ENSG00000155508 | ENST00000523698 | (GGT)3 |
|  |  |  | (AG)3 |
|  |  | ENST00000519903 | (GGT)3 |
|  |  |  | (AG)3 |
|  |  | ENST00000521450 | (GGT)3 |
|  |  |  | (AG)3 |
|  |  | ENST00000520671 | (GGT)3 |
|  |  |  | (AG)3 |
|  |  | ENST00000521583 | (GGT)3 |
|  |  |  | (AG)3 |
| CNPY1 | ENSG00000146910 | ENST00000636372 | (CCT)3 |
| CNPY2 | ENSG00000257727 | ENST00000551286 | (TC)3 |
| CNPY4 | ENSG00000166997 | ENST00000483756 |  |
| COBL | ENSG00000106078 | ENST00000449281 | (TGT)3 |
| COG5 | ENSG00000164597 | ENST00000605888 | (CTG)3 |
| COL23A1 | ENSG00000050767 | ENST00000390654 | (CG)3 |
|  |  | ENST00000646779 | (CA)3 |
| COL6A3 | ENSG00000163359 | ENST00000295550 | (GA)3 |
|  |  | ENST00000353578 |  |
|  |  | ENST00000472056 |  |
|  |  | ENST00000347401 |  |
|  |  | ENST00000392004 |  |
|  |  | ENST00000392003 |  |
|  |  | ENST00000433762 |  |
| COL7A1 | ENSG00000114270 | ENST00000422991 | (GT)3 |
| COMP | ENSG00000105664 | ENST00000542601 | (TGG)3 |
| COPS6 | ENSG00000168090 | ENST00000419210 | (TC)3 |
| COQ7 | ENSG00000167186 | ENST00000566110 | (GAA)3 |
|  |  | ENST00000544894 |  |
|  |  | ENST00000561858 |  |
| CORO2A | ENSG00000106789 | ENST00000343933 | (TG)3 |
| CORO2B | ENSG00000103647 | ENST00000261861 | (TC)3 |
|  |  | ENST00000540068 |  |
| CORO7 | ENSG00000262246 | ENST00000572549 | (CA)3 |
|  |  | ENST00000575850 |  |
| CPAMD8 | ENSG00000160111 | ENST00000388925 | (GGC)3 |
| CPE | ENSG00000109472 | ENST00000513982 | (AT)3 |
|  |  | ENST00000431967 |  |
| CPEB2 | ENSG00000137449 | ENST00000507071 | (AGCCGC)3 |
|  |  | ENST00000345451 |  |
| CPNE4 | ENSG00000196353 | ENST00000429747 | (TG)3 |
|  |  | ENST00000512055 |  |
|  |  | ENST00000511604 |  |
|  |  | ENST00000505881 |  |
|  |  | ENST00000514999 |  |
|  |  | ENST00000505957 |  |
| CPSF4 | ENSG00000160917 | ENST00000292476 | (CGC)3 |
|  |  | ENST00000441580 | (CT)3 |
|  |  | ENST00000412686 |  |
| CREB3L1 | ENSG00000157613 | ENST00000534787 | (CAC)3 |
| CREB5 | ENSG00000146592 | ENST00000426500 | (TGTT)3 |
|  |  |  | (CT)3 |
| CREM | ENSG00000095794 | ENST00000494479 | (AG)3 |
|  |  | ENST00000495301 |  |
| CRLF1 | ENSG00000006016 | ENST00000392386 | (CCG)3 |
| CRTAM | ENSG00000109943 | ENST00000533709 | (AC)3 |
| CRYAA | ENSG00000160202 | ENST00000398133 | (AG)3 |
| CSNK1A1 | ENSG00000113712 | ENST00000504676 | (CT)3 |
|  |  | ENST00000515435 |  |
|  |  | ENST00000606719 | (ATA)3 |
| CSTL1 | ENSG00000125823 | ENST00000619238 | (TGC)3 |
| CT45A1 | ENSG00000268940 | ENST00000594565 | (AT)3 |
| CTAG1A | ENSG00000268651 | ENST00000593606 | (TC)3 |
| CTAG1B | ENSG00000184033 | ENST00000328435 |  |
| CTCF | ENSG00000102974 | ENST00000401394 | (CA)3 |
| CTNND1 | ENSG00000198561 | ENST00000529526 | (GGA)3 |
|  |  | ENST00000426142 | (TA)3 |
|  |  | ENST00000532463 |  |
|  |  | ENST00000529986 |  |
|  |  | ENST00000532787 |  |
|  |  | ENST00000532649 | (GGA)3 |
|  |  | ENST00000528621 |  |
|  |  | ENST00000530748 |  |
|  |  | ENST00000528232 | (TA)3 |
|  |  | ENST00000529873 | (GGA)3 |
|  |  | ENST00000532844 |  |
|  |  | ENST00000526357 |  |
|  |  | ENST00000530094 | (TA)3 |
|  |  | ENST00000415361 |  |
|  |  | ENST00000532245 |  |
|  |  | ENST00000534579 | (GGA)3 |
|  |  | ENST00000530068 |  |
|  |  | ENST00000534647 |  |
| CTNND2 | ENSG00000169862 | ENST00000502551 | (TG)3 |
|  |  | ENST00000508761 |  |
| CXCR3 | ENSG00000186810 | ENST00000373691 | (GA)3 |
| CXorf40B | ENSG00000197021 | ENST00000462691 |  |
|  |  | ENST00000370406 |  |
|  |  | ENST00000355203 |  |
|  |  | ENST00000370404 |  |
|  |  | ENST00000370409 |  |
|  |  | ENST00000483447 |  |
| CYB5A | ENSG00000166347 | ENST00000494131 | (GC)3 |
| CYB5R2 | ENSG00000166394 | ENST00000524608 | (CT)3 |
| CYP26B1 | ENSG00000003137 | ENST00000412253 | (AC)3 |
|  |  |  | (GC)3 |
| CYP2B6 | ENSG00000197408 | ENST00000593831 | (GA)3 |
| CYP2F1 | ENSG00000197446 | ENST00000331105 | (TC)3 |
| CYP39A1 | ENSG00000146233 | ENST00000619708 | (GT)3 |
| CYTH1 | ENSG00000108669 | ENST00000586043 | (AGA)3 |
| CYTIP | ENSG00000115165 | ENST00000418920 | (TA)3 |
| DACT2 | ENSG00000164488 | ENST00000610183 | (TC)3 |
| DAOA | ENSG00000182346 | ENST00000329625 | (TA)3 |
|  |  |  | (AG)3 |
| DAP | ENSG00000112977 | ENST00000230895 | (CGC)3 |
|  |  | ENST00000432074 |  |
| DBN1 | ENSG00000113758 | ENST00000506117 | (AG)3 |
| DBNL | ENSG00000136279 | ENST00000448521 | (CGGCC)3 |
|  |  | ENST00000456905 |  |
| DCAF11 | ENSG00000100897 | ENST00000396936 | (TC)3 |
| DCAF13 | ENSG00000164934 | ENST00000521999 | (CAT)3 |
| DCAF8L1 | ENSG00000226372 | ENST00000441525 | (CT)3 |
| DDI2 | ENSG00000197312 | ENST00000480945 | (CCGAG)3 |
| DDIT4 | ENSG00000168209 | ENST00000307365 | (GGTCT)3 |
| DDX28 | ENSG00000182810 | ENST00000332395 | (CA)3 |
| DEFB128 | ENSG00000185982 | ENST00000334391 |  |
| DEK | ENSG00000124795 | ENST00000503715 | (AG)3 |
| DEPDC1 | ENSG00000024526 | ENST00000525124 | (AT)3 |
| DGCR6 | ENSG00000183628 | ENST00000413981 | (CAG)3 |
| DGKI | ENSG00000157680 | ENST00000453654 | (GT)3 |
| DHFR | ENSG00000228716 | ENST00000504396 | (TA)3 |
| DHRS2 | ENSG00000100867 | ENST00000432832 | (AGG)3 |
|  |  | ENST00000250383 |  |
|  |  | ENST00000344777 |  |
| DHX30 | ENSG00000132153 | ENST00000348968 | (TC)3 |
| DHX58 | ENSG00000108771 | ENST00000591220 | (CT)3 |
| DHX58 |  |  |  |
| DIRAS1 | ENSG00000176490 | ENST00000323469 |  |
|  |  | ENST00000585334 |  |
|  |  | ENST00000588128 |  |
| DLC1 | ENSG00000164741 | ENST00000503161 | (GT)3 |
| DLG2 | ENSG00000150672 | ENST00000530800 | (AGG)3 |
|  |  | ENST00000527088 | (TG)3 |
| DMBT1 | ENSG00000187908 | ENST00000338354 | (AC)3 |
|  |  | ENST00000344338 |  |
|  |  | ENST00000330163 |  |
|  |  | ENST00000368955 |  |
|  |  | ENST00000368909 |  |
|  |  | ENST00000368956 |  |
| DMPK | ENSG00000104936 | ENST00000596067 | (GA)3 |
| DNAH14 | ENSG00000185842 | ENST00000430092 | (TA)3 |
|  |  | ENST00000366850 |  |
|  |  | ENST00000400952 |  |
|  |  | ENST00000366849 |  |
| DNAH8 | ENSG00000124721 | ENST00000359357 | (TG)3 |
| DNM1L | ENSG00000087470 | ENST00000414834 | (AT)3 |
| DNMT1 | ENSG00000130816 | ENST00000340748 | (GCC)3 |
|  |  | ENST00000359526 |  |
| DOC2A | ENSG00000149927 | ENST00000567332 | (GA)3 |
|  |  | ENST00000563125 |  |
|  |  | ENST00000574405 |  |
| DOCK10 | ENSG00000135905 | ENST00000258390 | (GC)3 |
| DOCK2 | ENSG00000134516 | ENST00000540750 | (CA)3 |
| DOCK5 | ENSG00000147459 | ENST00000410074 | (CGG)3 |
|  |  | ENST00000481100 |  |
| DOCK9 | ENSG00000088387 | ENST00000427887 | (AGGCGG)3 |
| DOK1 | ENSG00000115325 | ENST00000409429 | (CT)3 |
| DOK5 | ENSG00000101134 | ENST00000395939 | (CA)3 |
| DPP10 | ENSG00000175497 | ENST00000410059 | (GCA)3 |
|  |  |  | (CAG)3 |
| DPP3 | ENSG00000254986 | ENST00000532019 | (AGG)3 |
|  |  | ENST00000533725 |  |
| DPY19L2 | ENSG00000177990 | ENST00000324472 | (TC)3 |
| DSCAM | ENSG00000171587 | ENST00000400454 | (GGCG)3 |
| DST | ENSG00000151914 | ENST00000523817 | (GA)3 |
| DUSP21 | ENSG00000189037 | ENST00000339042 | (AG)3 |
| DUSP22 | ENSG00000112679 | ENST00000603453 | (CA)3 |
|  |  | ENST00000605315 |  |
|  |  | ENST00000603881 |  |
|  |  | ENST00000605035 |  |
|  |  | ENST00000605863 |  |
|  |  | ENST00000604971 |  |
| DUSP6 | ENSG00000139318 | ENST00000547291 | (GT)3 |
| EAPP | ENSG00000129518 | ENST00000554792 | (TTG)3 |
| ECE1 | ENSG00000117298 | ENST00000473505 | (TG)3 |
| ECHDC3 | ENSG00000134463 | ENST00000422887 | (GT)3 |
| ECI2 | ENSG00000198721 | ENST00000495548 | (CG)3 |
| EEF1D | ENSG00000104529 | ENST00000528382 | (AC)3 |
| EFCAB2 | ENSG00000203666 | ENST00000366522 | (TG)3 |
| EFR3B | ENSG00000084710 | ENST00000405108 | (CT)3 |
|  |  |  | (GT)3 |
|  |  |  | (TC)3 |
| EGFLAM | ENSG00000164318 | ENST00000397202 | (AG)3 |
| EHD1 | ENSG00000110047 | ENST00000421510 |  |
|  |  |  | (CT)3 |
|  |  | ENST00000455148 | (AG)3 |
|  |  |  | (CT)3 |
| EID1 | ENSG00000255302 | ENST00000530028 | (GC)3 |
|  |  |  |  |
| EID2 | ENSG00000176396 | ENST00000390658 |  |
| EIF3E | ENSG00000104408 | ENST00000518345 | (AT)3 |
| EIF4A1 | ENSG00000161960 | ENST00000578754 | (CT)3 |
|  |  |  | (AT)3 |
|  |  |  | (TA)3 |
|  |  | ENST00000581770 | (CGGG)3 |
| EIF4E | ENSG00000151247 | ENST00000504432 | (CT)3 |
| EIF5A | ENSG00000132507 | ENST00000336458 | (GA)3 |
|  |  | ENST00000576930 |  |
|  |  | ENST00000572815 |  |
|  |  | ENST00000573542 |  |
|  |  | ENST00000419711 |  |
|  |  | ENST00000571955 |  |
|  |  | ENST00000573714 |  |
|  |  | ENST00000416016 |  |
| ELP2 | ENSG00000134759 | ENST00000543127 | (TC)3 |
| EMB | ENSG00000170571 | ENST00000303221 | (GCG)3 |
|  |  | ENST00000508934 |  |
| EML2 | ENSG00000125746 | ENST00000586770 | (ATAA)3 |
|  |  | ENST00000591721 |  |
|  |  | ENST00000593255 |  |
|  |  | ENST00000590043 |  |
|  |  | ENST00000590018 |  |
| EML5 | ENSG00000165521 | ENST00000554922 | (CGC)3 |
| ENC1 | ENSG00000171617 | ENST00000302351 | (CAA)3 |
|  |  | ENST00000618628 |  |
|  |  | ENST00000537006 |  |
|  |  | ENST00000508331 |  |
| ENO1 | ENSG00000074800 | ENST00000646539 | (TC)3 |
|  |  | ENST00000234590 |  |
|  |  | ENST00000647408 |  |
|  |  | ENST00000497492 |  |
|  |  | ENST00000646660 |  |
|  |  | ENST00000646906 |  |
|  |  | ENST00000643438 |  |
|  |  | ENST00000489867 |  |
| ENOPH1 | ENSG00000145293 | ENST00000505846 | (GA)3 |
| ENOSF1 | ENSG00000132199 | ENST00000251101 | (CTCCCGCC)3 |
| EPB42 | ENSG00000166947 | ENST00000568508 | (GA)3 |
| EPDR1 | ENSG00000086289 | ENST00000476620 | (AT)3 |
| EPHB1 | ENSG00000154928 | ENST00000493838 | (CT)3 |
| EPS15L1 | ENSG00000127527 | ENST00000602009 |  |
| EPS8L1 | ENSG00000131037 | ENST00000586329 | (TCC)3 |
| ERBB3 | ENSG00000065361 | ENST00000643266 | (GT)3 |
|  |  | ENST00000549061 |  |
|  |  | ENST00000549672 |  |
|  |  | ENST00000415288 |  |
| ERBB4 | ENSG00000178568 | ENST00000342788 | (CG)3 |
|  |  | ENST00000436443 |  |
| ESCO1 | ENSG00000141446 | ENST00000622333 | (TG)3 |
|  |  |  | (AAGA)3 |
| ESD | ENSG00000139684 | ENST00000378697 | (TA)3 |
|  |  |  | (GTT)3 |
| ESPNL | ENSG00000144488 | ENST00000409506 | (TTC)3 |
|  |  | ENST00000423032 |  |
| ESRP2 | ENSG00000103067 | ENST00000564382 | (TG)3 |
|  |  | ENST00000562724 |  |
| ETFDH | ENSG00000171503 | ENST00000507475 | (AT)3 |
| ETV4 | ENSG00000175832 | ENST00000586826 | (TCC)3 |
| EVPLL | ENSG00000214860 | ENST00000399134 | (GA)3 |
| EXOSC4 | ENSG00000178896 | ENST00000527954 | (GT)3 |
| F13A1 | ENSG00000124491 | ENST00000264870 | (AT)3 |
|  |  | ENST00000414279 |  |
| F8A3 | ENSG00000277150 | ENST00000622749 | (GCG)3 |
| FADS1 | ENSG00000149485 | ENST00000536991 | (TC)3 |
| FADS2 | ENSG00000134824 | ENST00000521571 |  |
|  |  | ENST00000355484 |  |
| FAIM2 | ENSG00000135472 | ENST00000550890 | (CAG)3 |
| FAM133B | ENSG00000234545 | ENST00000438306 | (AT)3 |
|  |  | ENST00000445716 | (TC)3 |
|  |  | ENST00000427372 | (AT)3 |
| FAM153B | ENSG00000182230 | ENST00000503724 | (TC)3 |
|  |  |  | (CCA)3 |
| FAM156A | ENSG00000268350 | ENST00000622447 | (CA)3 |
|  |  | ENST00000617970 |  |
|  |  | ENST00000611661 |  |
|  |  | ENST00000596733 |  |
|  |  | ENST00000623782 |  |
|  |  | ENST00000615092 |  |
|  |  | ENST00000612915 |  |
|  |  | ENST00000612846 |  |
|  |  | ENST00000622197 |  |
|  |  | ENST00000619373 |  |
|  |  | ENST00000622732 |  |
|  |  | ENST00000619518 |  |
|  |  | ENST00000618601 |  |
|  |  | ENST00000622323 |  |
|  |  | ENST00000612083 |  |
| FAM172A | ENSG00000113391 | ENST00000505869 | (AAATT)3 |
|  |  | ENST00000509739 | (TAAAT)3 |
|  |  | ENST00000509163 | (AAATT)3 |
| FAM184A | ENSG00000111879 | ENST00000621231 | (GCA)3 |
| FAM184B | ENSG00000047662 | ENST00000265018 | (GA)3 |
| FAM193A | ENSG00000125386 | ENST00000637812 | (CCG)3 |
| FAM19A2 | ENSG00000198673 | ENST00000549958 | (TC)3 |
| FAM43B | ENSG00000183114 | ENST00000332947 | (GC)3 |
| FAM47E | ENSG00000189157 | ENST00000510197 | (CCT)3 |
|  |  |  | (CA)3 |
| FAM50A | ENSG00000071859 | ENST00000393600 | (GCCGCT)3 |
| FAM53C | ENSG00000120709 | ENST00000511276 | (TG)3 |
| FAM81A | ENSG00000157470 | ENST00000560474 | (TAA)3 |
|  |  | ENST00000560087 |  |
| FAM83F | ENSG00000133477 | ENST00000333407 | (CCGGGG)3 |
| FAN1 | ENSG00000198690 | ENST00000562892 | (GAA)3 |
| FANCF | ENSG00000183161 | ENST00000327470 | (AG)3 |
|  |  |  |  |
| FBXL12 | ENSG00000127452 | ENST00000585379 | (TG)3 |
|  |  | ENST00000591009 |  |
|  |  | ENST00000590277 |  |
| FBXL16 | ENSG00000127585 | ENST00000562563 | (CGCC)3 |
| FBXL18 | ENSG00000155034 | ENST00000620087 | (CG)3 |
|  |  |  |  |
| FBXL7 | ENSG00000183580 | ENST00000510662 | (GAA)3 |
|  |  |  | (AGAAA)3 |
| FCF1 | ENSG00000119616 | ENST00000534938 | (GT)3 |
| FCHSD1 | ENSG00000197948 | ENST00000518499 | (CT)3 |
| FDPS | ENSG00000160752 | ENST00000611010 | (TA)3 |
| FGF11 | ENSG00000161958 | ENST00000575235 | (TC)3 |
|  |  | ENST00000572907 |  |
|  |  | ENST00000575398 |  |
|  |  | ENST00000575082 |  |
| FGF2 | ENSG00000138685 | ENST00000608478 | (GGCCGG)3 |
|  |  | ENST00000644866 |  |
| FGF7 | ENSG00000140285 | ENST00000560979 | (AC)3 |
| FGFR1 | ENSG00000077782 | ENST00000335922 | (CA)3 |
|  |  |  | (GA)3 |
| FIBCD1 | ENSG00000130720 | ENST00000448616 | (CCCGCG)3 |
|  |  | ENST00000372338 |  |
|  |  | ENST00000451466 |  |
| FKBP1A | ENSG00000088832 | ENST00000400137 | (CCGC)3 |
|  |  | ENST00000618612 |  |
|  |  | ENST00000439640 |  |
|  |  | ENST00000381719 |  |
|  |  | ENST00000614856 |  |
| FKBP1B | ENSG00000119782 | ENST00000452109 | (GT)3 |
| FLCN | ENSG00000154803 | ENST00000417064 | (CT)3 |
| FLNB | ENSG00000136068 | ENST00000493452 | (AT)3 |
| FMN2 | ENSG00000155816 | ENST00000319653 | (GCC)3 |
| FNBP4 | ENSG00000109920 | ENST00000540172 | (CT)3 |
| FNIP1 | ENSG00000217128 | ENST00000615660 | (AT)3 |
| FOLR3 | ENSG00000110203 | ENST00000622388 | (AG)3 |
|  |  | ENST00000611028 |  |
|  |  | ENST00000546166 |  |
| FOSB | ENSG00000125740 | ENST00000592811 | (TG)3 |
|  |  |  | (GT)3 |
|  |  | ENST00000586615 | (TG)3 |
|  |  |  | (GT)3 |
|  |  | ENST00000589593 | (TG)3 |
|  |  |  | (GT)3 |
| FOSL2 | ENSG00000075426 | ENST00000436647 | (ATT)3 |
| FOXD2 | ENSG00000186564 | ENST00000334793 | (GC)3 |
| FOXE3 | ENSG00000186790 | ENST00000335071 | (CG)3 |
| FOXJ3 | ENSG00000198815 | ENST00000422278 | (AT)3 |
| FOXP2 | ENSG00000128573 | ENST00000393491 | (CTTTCT)3 |
|  |  | ENST00000393489 |  |
| FOXP4 | ENSG00000137166 | ENST00000451305 | (TG)3 |
| FOXR2 | ENSG00000189299 | ENST00000339140 | (TC)3 |
| FRG1 | ENSG00000109536 | ENST00000524583 | (TA)3 |
| FRMD5 | ENSG00000171877 | ENST00000417257 | (GGGC)3 |
|  |  | ENST00000402883 |  |
|  |  | ENST00000484674 | (AT)3 |
| FRRS1 | ENSG00000156869 | ENST00000646001 | (TA)3 |
|  |  | ENST00000287474 |  |
| FRS2 | ENSG00000166225 | ENST00000547414 | (GAT)3 |
| FSCN1 | ENSG00000075618 | ENST00000444748 | (GT)3 |
|  |  |  | (CT)3 |
|  |  | ENST00000447103 | (GT)3 |
|  |  |  | (CT)3 |
|  |  | ENST00000405801 | (GT)3 |
|  |  |  | (CT)3 |
| FSTL3 | ENSG00000070404 | ENST00000592947 | (GT)3 |
|  |  |  | (CAA)3 |
|  |  | ENST00000588773 | (GT)3 |
|  |  |  | (CAA)3 |
|  |  | ENST00000591573 | (GT)3 |
|  |  |  | (CAA)3 |
| FSTL4 | ENSG00000053108 | ENST00000510685 | (CA)3 |
| FTCD | ENSG00000160282 | ENST00000291670 | (AG)3 |
|  |  | ENST00000397748 |  |
|  |  | ENST00000397746 |  |
|  |  | ENST00000397743 |  |
| FUT3 | ENSG00000171124 | ENST00000303225 | (CT)3 |
|  |  |  |  |
|  |  | ENST00000458379 |  |
|  |  |  |  |
|  |  | ENST00000589918 |  |
|  |  |  |  |
|  |  | ENST00000589620 |  |
|  |  |  |  |
|  |  | ENST00000589714 |  |
|  |  |  |  |
|  |  | ENST00000587048 |  |
|  |  |  |  |
|  |  | ENST00000585715 |  |
|  |  |  |  |
| FUT5 | ENSG00000130383 | ENST00000588525 | (TC)3 |
|  |  |  | (CT)3 |
|  |  |  |  |
| FUT6 | ENSG00000156413 | ENST00000524754 |  |
|  |  | ENST00000527106 |  |
|  |  | ENST00000318336 |  |
|  |  | ENST00000286955 |  |
|  |  | ENST00000529165 |  |
|  |  | ENST00000531085 |  |
|  |  | ENST00000531199 |  |
|  |  | ENST00000532464 |  |
|  |  | ENST00000528505 |  |
| FYCO1 | ENSG00000163820 | ENST00000438446 | (TG)3 |
| FZD1 | ENSG00000157240 | ENST00000287934 | (AG)3 |
| FZD5 | ENSG00000163251 | ENST00000295417 | (TG)3 |
| FZD8 | ENSG00000177283 | ENST00000374694 | (CT)3 |
| GABARAPL2 | ENSG00000034713 | ENST00000568455 |  |
| GABRA1 | ENSG00000022355 | ENST00000638159 | (AT)3 |
|  |  | ENST00000522651 |  |
| GABRG2 | ENSG00000113327 | ENST00000640757 | (GC)3 |
|  |  | ENST00000638660 | (CT)3 |
|  |  | ENST00000638552 |  |
|  |  | ENST00000640574 |  |
| GABRG3 | ENSG00000182256 | ENST00000333743 | (TG)3 |
| GADD45B | ENSG00000099860 | ENST00000587887 | (GCC)3 |
| GALNT14 | ENSG00000158089 | ENST00000406653 | (TC)3 |
| GALNT8 | ENSG00000130035 | ENST00000252318 | (CT)3 |
| GAPVD1 | ENSG00000165219 | ENST00000265956 | (GA)3 |
|  |  |  | (AC)3 |
| GBA2 | ENSG00000070610 | ENST00000378088 | (TG)3 |
| GBP3 | ENSG00000117226 | ENST00000564665 | (TC)3 |
|  |  |  |  |
| GCA | ENSG00000115271 | ENST00000446271 | (GA)3 |
| GCHFR | ENSG00000137880 | ENST00000559932 | (CCT)3 |
| GCNT7 | ENSG00000124091 | ENST00000243913 | (AT)3 |
| GDI2 | ENSG00000057608 | ENST00000380127 | (TC)3 |
|  |  | ENST00000609712 |  |
| GDPD5 | ENSG00000158555 | ENST00000526177 | (CT)3 |
|  |  |  | (AC)3 |
| GEMIN4 | ENSG00000179409 | ENST00000576778 | (TG)3 |
|  |  | ENST00000576383 |  |
| GFPT2 | ENSG00000131459 | ENST00000518906 | (AG)3 |
|  |  |  | (TC)3 |
| GIPC1 | ENSG00000123159 | ENST00000393028 | (CT)3 |
|  |  | ENST00000591349 |  |
|  |  | ENST00000587210 |  |
|  |  | ENST00000587969 |  |
| GIT1 | ENSG00000108262 | ENST00000577466 |  |
| GJA3 | ENSG00000121743 | ENST00000241125 | (GCC)3 |
| GJA4 | ENSG00000187513 | ENST00000342280 | (AC)3 |
| GJA5 | ENSG00000265107 | ENST00000579774 | (CTT)3 |
|  |  | ENST00000621517 |  |
| GJC2 | ENSG00000198835 | ENST00000366714 | (AG)3 |
| GLCE | ENSG00000138604 | ENST00000559420 | (AGC)3 |
| GLDC | ENSG00000178445 | ENST00000639318 | (TC)3 |
|  |  | ENST00000640208 |  |
| GMEB2 | ENSG00000101216 | ENST00000370069 |  |
| GMFB | ENSG00000197045 | ENST00000553333 | (AT)3 |
| GNAS | ENSG00000087460 | ENST00000371081 | (CCG)3 |
| GOLGA6A | ENSG00000159289 | ENST00000290438 | (CA)3 |
| GOLGA6D | ENSG00000140478 | ENST00000434739 |  |
| GOLGA6L1 | ENSG00000273976 | ENST00000614055 |  |
|  |  |  | (TA)3 |
| GOLGA6L6 | ENSG00000277322 | ENST00000619213 | (CA)3 |
|  |  |  | (TA)3 |
| GOLGA8A | ENSG00000175265 | ENST00000432566 | (GA)3 |
| GOLGA8F | ENSG00000153684 | ENST00000526619 | (TG)3 |
| GOLT1B | ENSG00000111711 | ENST00000542038 | (TTC)3 |
| GOSR2 | ENSG00000108433 | ENST00000638892 | (AGA)3 |
| GPR143 | ENSG00000101850 | ENST00000447366 | (CT)3 |
|  |  |  | (TC)3 |
|  |  | ENST00000431126 | (CT)3 |
|  |  |  | (TC)3 |
| GPR17 | ENSG00000144230 | ENST00000544369 | (TGG)3 |
|  |  | ENST00000272644 |  |
|  |  | ENST00000423019 |  |
| GPR176 | ENSG00000166073 | ENST00000299092 | (GGA)3 |
|  |  |  | (GC)3 |
|  |  | ENST00000561100 | (GGA)3 |
|  |  |  | (GC)3 |
| GPR35 | ENSG00000178623 | ENST00000319838 | (CT)3 |
|  |  | ENST00000403859 |  |
|  |  | ENST00000407714 |  |
| GPX2 | ENSG00000176153 | ENST00000612794 |  |
|  |  | ENST00000389614 |  |
| GRB14 | ENSG00000115290 | ENST00000424693 | (TA)3 |
| GREM1 | ENSG00000166923 | ENST00000622074 | (TAT)3 |
|  |  | ENST00000560677 |  |
|  |  | ENST00000560830 |  |
| GRIA2 | ENSG00000120251 | ENST00000507898 | (CG)3 |
|  |  | ENST00000393815 |  |
|  |  | ENST00000506284 |  |
|  |  | ENST00000505888 |  |
|  |  | ENST00000503437 | (CA)3 |
| GRIK1 | ENSG00000171189 | ENST00000389125 | (TC)3 |
|  |  | ENST00000399913 |  |
|  |  | ENST00000399914 |  |
|  |  | ENST00000399907 |  |
|  |  | ENST00000399909 |  |
| GRM4 | ENSG00000124493 | ENST00000374177 | (CT)3 |
|  |  | ENST00000609443 | (GGCTG)3 |
| GRWD1 | ENSG00000105447 | ENST00000598711 | (CT)3 |
| GSK3A | ENSG00000105723 | ENST00000398249 | (GAG)3 |
| GSTT1 | ENSG00000277656 | ENST00000612885 | (GGTC)3 |
|  |  | ENST00000627958 |  |
|  |  | ENST00000628417 |  |
| GTF2H1 | ENSG00000110768 | ENST00000524753 | (TA)3 |
| GTF2H2C | ENSG00000183474 | ENST00000512736 |  |
|  |  | ENST00000510979 |  |
|  |  | ENST00000514162 |  |
|  |  | ENST00000380729 |  |
| GTF3C5 | ENSG00000148308 | ENST00000439697 | (GT)3 |
|  |  |  | (CT)3 |
| GUK1 | ENSG00000143774 | ENST00000435153 | (TC)3 |
|  |  | ENST00000412265 |  |
| GYPC | ENSG00000136732 | ENST00000356887 | (CT)3 |
| H2BFM | ENSG00000101812 | ENST00000355016 |  |
| HCFC1R1 | ENSG00000103145 | ENST00000248089 |  |
|  |  | ENST00000572355 |  |
|  |  | ENST00000574980 |  |
|  |  | ENST00000354679 |  |
|  |  | ENST00000573842 |  |
| HECTD2 | ENSG00000165338 | ENST00000631422 | (ACC)3 |
| HECTD3 | ENSG00000126107 | ENST00000372168 | (TG)3 |
| HEG1 | ENSG00000173706 | ENST00000311127 | (CCGCTGC)3 |
| HEPH | ENSG00000089472 | ENST00000519389 | (TCCT)3 |
| HEXB | ENSG00000049860 | ENST00000509579 | (TG)3 |
|  |  |  | (GA)3 |
| HGS | ENSG00000185359 | ENST00000571518 | (AGG)3 |
|  |  | ENST00000577012 |  |
| HHLA2 | ENSG00000114455 | ENST00000467562 | (CA)3 |
| HHLA3 | ENSG00000197568 | ENST00000361764 | (GT)3 |
|  |  | ENST00000359875 |  |
|  |  | ENST00000370940 |  |
| HIST1H3E | ENSG00000274750 | ENST00000634733 | (TA)3 |
| HLA-C | ENSG00000204525 | ENST00000640219 | (CGGGG)3 |
| HLA-DOA | ENSG00000204252 | ENST00000229829 | (CTT)3 |
| HMGB4 | ENSG00000176256 | ENST00000519684 | (TC)3 |
|  |  | ENST00000522796 |  |
| HNF4A | ENSG00000101076 | ENST00000316673 | (TGC)3 |
| HNRNPUL1 | ENSG00000105323 | ENST00000597725 | (TC)3 |
| HOOK2 | ENSG00000095066 | ENST00000589400 | (GA)3 |
|  |  | ENST00000592079 |  |
|  |  | ENST00000590839 |  |
|  |  | ENST00000588052 |  |
| HPD | ENSG00000158104 | ENST00000543163 | (CT)3 |
| HPS5 | ENSG00000110756 | ENST00000544218 | (CA)3 |
| HRCT1 | ENSG00000196196 | ENST00000354323 | (AG)3 |
| HRH1 | ENSG00000196639 | ENST00000438284 | (CT)3 |
|  |  | ENST00000431010 |  |
|  |  | ENST00000413416 |  |
| HSD17B3 | ENSG00000130948 | ENST00000375263 | (AG)3 |
|  |  | ENST00000375262 |  |
| HSD17B4 | ENSG00000133835 | ENST00000645099 | (GA)3 |
| HSP90B1 | ENSG00000166598 | ENST00000549334 | (AG)3 |
|  |  |  |  |
| HSPG2 | ENSG00000142798 | ENST00000374695 | (GAGC)3 |
|  |  |  | (AG)3 |
| HTATIP2 | ENSG00000109854 | ENST00000530266 | (TCC)3 |
|  |  | ENST00000421577 |  |
|  |  | ENST00000443524 |  |
|  |  | ENST00000451739 |  |
|  |  | ENST00000532505 |  |
| ICAM3 | ENSG00000076662 | ENST00000589261 | (CT)3 |
|  |  | ENST00000590569 | (CCT)3 |
|  |  | ENST00000589249 | (CT)3 |
| ICAM4 | ENSG00000105371 | ENST00000380770 |  |
|  |  | ENST00000340992 |  |
| IDI1 | ENSG00000067064 | ENST00000427898 | (AC)3 |
| IDUA | ENSG00000127415 | ENST00000514224 | (GT)3 |
| IER2 | ENSG00000160888 | ENST00000587885 | (AG)3 |
|  |  | ENST00000292433 |  |
|  |  | ENST00000588173 |  |
| IER5L | ENSG00000188483 | ENST00000372491 | (CAG)3 |
|  |  |  | (GGC)3 |
|  |  |  | (CCG)3 |
| IFITM2 | ENSG00000185201 | ENST00000533141 | (TC)3 |
| IFT27 | ENSG00000100360 | ENST00000440696 | (TG)3 |
| IGDCC3 | ENSG00000174498 | ENST00000558354 | (TC)3 |
| IGFBP2 | ENSG00000115457 | ENST00000434997 |  |
| IKBKG | ENSG00000269335 | ENST00000615186 | (GC)3 |
| IKZF4 | ENSG00000123411 | ENST00000547791 | (AC)3 |
| IL10 | ENSG00000136634 | ENST00000471071 | (TC)3 |
| IL19 | ENSG00000142224 | ENST00000340758 | (CA)3 |
| IL2RG | ENSG00000147168 | ENST00000464642 | (CCT)3 |
| ILF3 | ENSG00000129351 | ENST00000587941 | (AC)3 |
|  |  |  | (CT)3 |
| IMMP2L | ENSG00000184903 | ENST00000405709 | (AT)3 |
|  |  | ENST00000331762 |  |
|  |  | ENST00000452895 |  |
|  |  | ENST00000447215 |  |
| IMP3 | ENSG00000177971 | ENST00000314852 | (CG)3 |
|  |  | ENST00000403490 |  |
| IMP4 | ENSG00000136718 | ENST00000428740 | (TC)3 |
| IMPDH1 | ENSG00000106348 | ENST00000496200 | (CG)3 |
|  |  | ENST00000626419 |  |
|  |  | ENST00000470772 |  |
|  |  | ENST00000480861 |  |
| INPP5K | ENSG00000132376 | ENST00000449479 | (CT)3 |
|  |  |  | (TG)3 |
|  |  | ENST00000498390 | (CT)3 |
|  |  |  | (TG)3 |
| IPO5 | ENSG00000065150 | ENST00000471898 | (CGC)3 |
|  |  | ENST00000357602 |  |
|  |  | ENST00000475420 |  |
|  |  | ENST00000480641 | (TA)3 |
| IPO8 | ENSG00000133704 | ENST00000544829 | (AT)3 |
| IQCE | ENSG00000106012 | ENST00000623361 | (TC)3 |
|  |  | ENST00000325979 |  |
|  |  | ENST00000423395 |  |
|  |  | ENST00000422276 |  |
|  |  | ENST00000423196 | (TG)3 |
| IRAK1BP1 | ENSG00000146243 | ENST00000607739 | (CT)3 |
| IREB2 | ENSG00000136381 | ENST00000560840 | (AT)3 |
| IRF2BP1 | ENSG00000170604 | ENST00000302165 | (GC)3 |
| IRF2BP2 | ENSG00000168264 | ENST00000366610 | (CTC)3 |
|  |  | ENST00000366609 |  |
| IRF7 | ENSG00000185507 | ENST00000525445 | (GC)3 |
| IRF8 | ENSG00000140968 | ENST00000562492 | (AC)3 |
|  |  | ENST00000569607 |  |
| IRS1 | ENSG00000169047 | ENST00000305123 | (TGG)3 |
| ISG15 | ENSG00000187608 | ENST00000624697 | (TG)3 |
|  |  | ENST00000624652 |  |
| ITCH | ENSG00000078747 | ENST00000374864 | (AT)3 |
|  |  | ENST00000486883 |  |
|  |  | ENST00000262650 |  |
| JAG2 | ENSG00000184916 | ENST00000331782 | (CGGG)3 |
|  |  | ENST00000347004 |  |
| KANK1 | ENSG00000107104 | ENST00000382303 | (TC)3 |
|  |  | ENST00000619269 |  |
|  |  | ENST00000382297 |  |
| KBTBD11 | ENSG00000176595 | ENST00000320248 | (CG)3 |
| KCNA1 | ENSG00000111262 | ENST00000382545 |  |
| KCNA2 | ENSG00000177301 | ENST00000639233 | (AG)3 |
| KCNA3 | ENSG00000177272 | ENST00000369769 | (GAG)3 |
| KCNA5 | ENSG00000130037 | ENST00000252321 | (AG)3 |
| KCNH7 | ENSG00000184611 | ENST00000332142 | (CG)3 |
|  |  |  | (GA)3 |
|  |  | ENST00000328032 | (CG)3 |
|  |  |  | (GA)3 |
| KCNJ18 | ENSG00000260458 | ENST00000567955 | (CT)3 |
| KCNK10 | ENSG00000100433 | ENST00000312350 | (GA)3 |
|  |  |  | (AC)3 |
| KCNQ3 | ENSG00000184156 | ENST00000638588 | (TG)3 |
| KCNT1 | ENSG00000107147 | ENST00000263604 | (GCCCGCC)3 |
| KCTD12 | ENSG00000178695 | ENST00000377474 | (GCG)3 |
| KDM1B | ENSG00000165097 | ENST00000546309 | (AAAAC)3 |
| KHSRP | ENSG00000088247 | ENST00000595548 | (TCC)3 |
|  |  | ENST00000595258 |  |
| KIAA0319 | ENSG00000137261 | ENST00000430948 | (CT)3 |
|  |  | ENST00000537886 |  |
|  |  | ENST00000378214 |  |
|  |  | ENST00000543707 |  |
| KIAA0319L | ENSG00000142687 | ENST00000373266 | (TG)3 |
| KIAA0355 | ENSG00000166398 | ENST00000588470 | (CA)3 |
| KIAA0391 | ENSG00000100890 | ENST00000604948 | (GA)3 |
|  |  | ENST00000321130 | (TAT)3 |
|  |  | ENST00000605870 |  |
|  |  | ENST00000557404 |  |
| KIAA0895 | ENSG00000164542 | ENST00000453212 | (TC)3 |
|  |  | ENST00000431396 |  |
| KIAA1671 | ENSG00000197077 | ENST00000406486 | (TTGT)3 |
|  |  | ENST00000637069 | (CT)3 |
| KIF6 | ENSG00000164627 | ENST00000394362 | (TC)3 |
|  |  | ENST00000229913 |  |
|  |  | ENST00000538893 |  |
| KITLG | ENSG00000049130 | ENST00000357116 | (GT)3 |
| KLC2 | ENSG00000174996 | ENST00000394065 | (GAG)3 |
| KLF8 | ENSG00000102349 | ENST00000640927 | (CT)3 |
| KLHDC4 | ENSG00000104731 | ENST00000446344 | (AG)3 |
| KLHL12 | ENSG00000117153 | ENST00000367258 | (TA)3 |
| KLHL17 | ENSG00000187961 | ENST00000622660 | (GAA)3 |
|  |  |  | (CA)3 |
| KLHL35 | ENSG00000149243 | ENST00000376292 | (GC)3 |
|  |  |  |  |
| KLRG1 | ENSG00000139187 | ENST00000539240 | (TC)3 |
| KPNA3 | ENSG00000102753 | ENST00000261667 |  |
| KRT23 | ENSG00000108244 | ENST00000436344 | (TG)3 |
| KRT35 | ENSG00000197079 | ENST00000246639 | (GT)3 |
| KRT86 | ENSG00000170442 | ENST00000553310 | (TA)3 |
|  |  |  | (CT)3 |
|  |  | ENST00000423955 | (TA)3 |
|  |  |  | (CT)3 |
|  |  | ENST00000293525 |  |
| KRTAP10-2 | ENSG00000205445 | ENST00000391621 | (TCAC)3 |
| KRTAP10-9 | ENSG00000221837 | ENST00000397911 | (CA)3 |
| KRTAP21-1 | ENSG00000187005 | ENST00000335093 | (TC)3 |
| KRTAP4-8 | ENSG00000204880 | ENST00000333822 | (CT)3 |
| KRTAP4-9 | ENSG00000212722 | ENST00000391415 |  |
| KRTAP5-9 | ENSG00000254997 | ENST00000528743 | (GT)3 |
| KSR1 | ENSG00000141068 | ENST00000583370 | (CT)3 |
|  |  | ENST00000398988 |  |
|  |  | ENST00000509603 |  |
| KTN1 | ENSG00000126777 | ENST00000554507 | (GA)3 |
|  |  | ENST00000554890 |  |
| L3MBTL1 | ENSG00000185513 | ENST00000434666 | (AG)3 |
| LAX1 | ENSG00000122188 | ENST00000367217 | (CT)3 |
| LBX2 | ENSG00000179528 | ENST00000460508 | (TC)3 |
|  |  | ENST00000341396 |  |
| LCE2B | ENSG00000159455 | ENST00000368780 | (TTC)3 |
| LCE2C | ENSG00000187180 | ENST00000368783 |  |
| LCE2D | ENSG00000187223 | ENST00000368784 |  |
| LCE5A | ENSG00000186207 | ENST00000334269 | (ATA)3 |
| LCN10 | ENSG00000187922 | ENST00000497771 | (AG)3 |
|  |  | ENST00000527229 |  |
| LEKR1 | ENSG00000197980 | ENST00000470811 | (TA)3 |
| LHX2 | ENSG00000106689 | ENST00000560961 | (CGG)3 |
| LILRA1 | ENSG00000104974 | ENST00000251372 | (CT)3 |
| LILRA3 | ENSG00000278046 | ENST00000610381 | (TG)3 |
|  |  | ENST00000612127 |  |
|  |  | ENST00000610305 |  |
| LILRB1 | ENSG00000104972 | ENST00000396331 | (CT)3 |
|  |  | ENST00000396327 |  |
|  |  | ENST00000396332 |  |
|  |  | ENST00000324602 |  |
| LILRB2 | ENSG00000131042 | ENST00000391748 | (CA)3 |
|  |  |  | (CT)3 |
|  |  | ENST00000314446 | (CA)3 |
|  |  |  | (CT)3 |
|  |  | ENST00000391749 | (CA)3 |
|  |  |  | (CT)3 |
| LIN37 | ENSG00000267796 | ENST00000587751 |  |
| LIPA | ENSG00000107798 | ENST00000371837 | (GT)3 |
| LLGL2 | ENSG00000073350 | ENST00000583658 | (GGT)3 |
| LMAN1L | ENSG00000140506 | ENST00000562810 | (AC)3 |
|  |  |  | (CA)3 |
|  |  |  |  |
| LMTK3 | ENSG00000142235 | ENST00000600059 | (CG)3 |
| LOXL2 | ENSG00000134013 | ENST00000524144 | (GT)3 |
|  |  |  | (AG)3 |
|  |  |  | (GA)3 |
|  |  | ENST00000520871 | (GT)3 |
|  |  |  | (AG)3 |
|  |  |  | (GA)3 |
| LPA | ENSG00000198670 | ENST00000316300 | (AC)3 |
| LPAR1 | ENSG00000198121 | ENST00000541779 | (TA)3 |
| LPIN2 | ENSG00000101577 | ENST00000584915 |  |
| LPIN3 | ENSG00000132793 | ENST00000373257 | (TG)3 |
|  |  | ENST00000632009 |  |
| LRP12 | ENSG00000147650 | ENST00000523007 | (AGC)3 |
| LRP2BP | ENSG00000109771 | ENST00000510776 | (AG)3 |
|  |  |  | (TG)3 |
| LRP5L | ENSG00000100068 | ENST00000610821 | (CTG)3 |
|  |  |  | (AC)3 |
|  |  | ENST00000402859 | (CTG)3 |
|  |  |  | (AC)3 |
|  |  | ENST00000444995 | (CTG)3 |
|  |  |  | (AC)3 |
|  |  | ENST00000402785 |  |
| LRRC28 | ENSG00000168904 | ENST00000561276 | (TG)3 |
| LRRC36 | ENSG00000159708 | ENST00000563189 | (AC)3 |
| LRRC37A3 | ENSG00000176809 | ENST00000334962 | (CT)3 |
| LRRC49 | ENSG00000137821 | ENST00000443425 | (AC)3 |
|  |  | ENST00000560158 | (GAA)3 |
| LSP1 | ENSG00000130592 | ENST00000381775 | (AG)3 |
|  |  |  |  |
| LSR | ENSG00000105699 | ENST00000601623 | (TC)3 |
| LTBR | ENSG00000111321 | ENST00000228918 | (GCC)3 |
|  |  | ENST00000536876 |  |
| LUZP4 | ENSG00000102021 | ENST00000371921 | (GT)3 |
| LYRM1 | ENSG00000102897 | ENST00000412082 | (ATG)3 |
|  |  | ENST00000562740 |  |
| MAD1L1 | ENSG00000002822 | ENST00000437877 | (GT)3 |
|  |  | ENST00000445959 | (CT)3 |
| MAGEA1 | ENSG00000198681 | ENST00000356661 | (CA)3 |
|  |  |  | (TC)3 |
| MAGEA10 | ENSG00000124260 | ENST00000370323 |  |
|  |  | ENST00000244096 |  |
|  |  | ENST00000444834 |  |
|  |  | ENST00000427322 |  |
|  |  | ENST00000583480 |  |
| MAGEA2 | ENSG00000268606 | ENST00000620710 | (CA)3 |
|  |  |  | (TC)3 |
|  |  | ENST00000611557 | (CA)3 |
|  |  |  | (TC)3 |
|  |  | ENST00000623806 | (CA)3 |
|  |  |  | (TC)3 |
|  |  | ENST00000623438 | (CA)3 |
|  |  |  | (TC)3 |
|  |  | ENST00000598543 | (CA)3 |
|  |  |  | (TC)3 |
|  |  | ENST00000611674 | (CA)3 |
|  |  |  | (TC)3 |
|  |  | ENST00000595583 | (CA)3 |
|  |  |  | (TC)3 |
|  |  | ENST00000617846 | (CA)3 |
|  |  |  | (TC)3 |
| MAGEA2B | ENSG00000183305 | ENST00000331220 | (CA)3 |
|  |  |  | (TC)3 |
|  |  | ENST00000370293 | (CA)3 |
|  |  |  | (TC)3 |
|  |  | ENST00000423993 | (CA)3 |
|  |  |  | (TC)3 |
|  |  | ENST00000447530 | (CA)3 |
|  |  |  | (TC)3 |
|  |  | ENST00000458057 | (CA)3 |
|  |  |  | (TC)3 |
|  |  | ENST00000422085 | (CA)3 |
|  |  |  | (TC)3 |
|  |  | ENST00000453150 | (CA)3 |
|  |  |  | (TC)3 |
|  |  | ENST00000409560 | (CA)3 |
|  |  |  | (TC)3 |
| MAGEA3 | ENSG00000221867 | ENST00000370278 |  |
|  |  |  |  |
|  |  | ENST00000417212 |  |
|  |  |  |  |
| MAGEA4 | ENSG00000147381 | ENST00000431963 |  |
|  |  |  |  |
|  |  | ENST00000276344 |  |
|  |  |  |  |
|  |  | ENST00000448295 |  |
|  |  |  |  |
|  |  | ENST00000393921 |  |
|  |  |  |  |
|  |  | ENST00000430273 |  |
|  |  |  |  |
|  |  | ENST00000441865 |  |
|  |  |  |  |
|  |  | ENST00000370340 |  |
|  |  |  |  |
|  |  | ENST00000425182 |  |
|  |  |  |  |
|  |  | ENST00000416020 |  |
|  |  |  |  |
|  |  | ENST00000457310 |  |
|  |  |  |  |
|  |  | ENST00000393920 |  |
|  |  |  |  |
|  |  | ENST00000370335 |  |
|  |  |  |  |
|  |  | ENST00000360243 |  |
|  |  |  |  |
| MAGEA6 | ENSG00000197172 | ENST00000329342 |  |
|  |  |  |  |
|  |  | ENST00000616035 |  |
|  |  |  |  |
|  |  | ENST00000457643 |  |
|  |  |  |  |
| MAGEA8 | ENSG00000156009 | ENST00000535454 | (CT)3 |
|  |  | ENST00000542674 |  |
|  |  | ENST00000286482 |  |
| MAGEA9 | ENSG00000123584 | ENST00000243314 | (TC)3 |
|  |  |  |  |
| MAGEA9B | ENSG00000267978 | ENST00000595065 |  |
|  |  |  |  |
|  |  | ENST00000593349 |  |
|  |  |  |  |
|  |  | ENST00000602102 |  |
|  |  |  |  |
| MAGEB16 | ENSG00000189023 | ENST00000399992 | (GA)3 |
| MAGEB17 | ENSG00000182798 | ENST00000400004 | (CA)3 |
| MAGEB3 | ENSG00000198798 | ENST00000361644 | (CT)3 |
| MAGEB5 | ENSG00000188408 | ENST00000602297 | (CTG)3 |
| MAGEB6 | ENSG00000176746 | ENST00000379034 | (CT)3 |
|  |  |  |  |
| MAGEE1 | ENSG00000198934 | ENST00000361470 |  |
| MAGI2 | ENSG00000187391 | ENST00000354212 | (TC)3 |
|  |  | ENST00000419488 |  |
|  |  | ENST00000637441 |  |
|  |  | ENST00000522391 |  |
| MAN1C1 | ENSG00000117643 | ENST00000263979 |  |
| MANBA | ENSG00000109323 | ENST00000644159 |  |
|  |  | ENST00000226578 |  |
|  |  | ENST00000647097 |  |
|  |  | ENST00000642252 |  |
|  |  | ENST00000505239 |  |
| MAP4 | ENSG00000047849 | ENST00000420772 | (TG)3 |
|  |  |  | (GA)3 |
| MAP7D1 | ENSG00000116871 | ENST00000429533 | (CT)3 |
|  |  |  |  |
|  |  |  | (ACC)3 |
|  |  | ENST00000530729 | (CT)3 |
|  |  |  |  |
|  |  |  | (ACC)3 |
| MARVELD3 | ENSG00000140832 | ENST00000567501 | (GA)3 |
| MASP1 | ENSG00000127241 | ENST00000439271 |  |
|  |  |  | (AG)3 |
| MAVS | ENSG00000088888 | ENST00000416600 |  |
| MAZ | ENSG00000103495 | ENST00000562557 | (CA)3 |
|  |  |  | (GT)3 |
|  |  | ENST00000568544 | (CA)3 |
|  |  |  | (GT)3 |
|  |  | ENST00000569978 | (CA)3 |
|  |  |  | (GT)3 |
|  |  | ENST00000568282 | (CA)3 |
|  |  |  | (GT)3 |
| MCFD2 | ENSG00000180398 | ENST00000444761 | (CG)3 |
|  |  |  | (CCG)3 |
| MCM10 | ENSG00000065328 | ENST00000479669 | (GA)3 |
|  |  |  | (CT)3 |
| MCM2 | ENSG00000073111 | ENST00000480910 | (GA)3 |
|  |  | ENST00000472731 |  |
| MDGA1 | ENSG00000112139 | ENST00000515437 | (TG)3 |
| MDGA2 | ENSG00000139915 | ENST00000486952 |  |
| MDH2 | ENSG00000146701 | ENST00000461263 | (AG)3 |
|  |  | ENST00000315758 |  |
| MECOM | ENSG00000085276 | ENST00000494292 | (CA)3 |
| MED20 | ENSG00000124641 | ENST00000394251 | (GT)3 |
|  |  |  |  |
| MED25 | ENSG00000104973 | ENST00000617849 | (GTG)3 |
|  |  | ENST00000620467 |  |
|  |  | ENST00000622402 |  |
|  |  | ENST00000612854 |  |
|  |  | ENST00000312865 |  |
| MEIS1 | ENSG00000143995 | ENST00000398506 | (TA)3 |
| MEP1B | ENSG00000141434 | ENST00000581184 |  |
| METRNL | ENSG00000176845 | ENST00000570778 | (CA)3 |
|  |  | ENST00000571814 |  |
| MGAT2 | ENSG00000168282 | ENST00000305386 | (GCC)3 |
|  |  |  | (CGCC)3 |
| MGAT5B | ENSG00000167889 | ENST00000428789 | (CT)3 |
| MIA | ENSG00000261857 | ENST00000597600 | (AG)3 |
|  |  |  | (CT)3 |
|  |  | ENST00000594436 | (AG)3 |
|  |  |  | (CT)3 |
|  |  | ENST00000597784 | (AG)3 |
|  |  |  | (CT)3 |
|  |  | ENST00000263369 | (AG)3 |
|  |  |  | (CT)3 |
| MIA3 | ENSG00000154305 | ENST00000340535 | (GGA)3 |
| MICA | ENSG00000204520 | ENST00000616296 | (AG)3 |
| MICALL1 | ENSG00000100139 | ENST00000445494 | (CCT)3 |
| MICB | ENSG00000204516 | ENST00000538442 | (TG)3 |
| MLXIP | ENSG00000175727 | ENST00000366272 | (CT)3 |
|  |  |  | (CA)3 |
| MORN2 | ENSG00000188010 | ENST00000409077 | (GA)3 |
| MORN5 | ENSG00000185681 | ENST00000418632 | (CA)3 |
| MPHOSPH6 | ENSG00000135698 | ENST00000563504 | (AGA)3 |
| MPZ | ENSG00000158887 | ENST00000491222 | (TC)3 |
| MRPL34 | ENSG00000130312 | ENST00000595444 | (CCT)3 |
| MRPS22 | ENSG00000175110 | ENST00000495225 | (GA)3 |
| MRPS28 | ENSG00000147586 | ENST00000521434 | (CAC)3 |
|  |  | ENST00000519120 | (TA)3 |
|  |  | ENST00000520946 | (CAC)3 |
| MS4A3 | ENSG00000149516 | ENST00000395032 | (GT)3 |
|  |  |  | (AT)3 |
| MS4A7 | ENSG00000166927 | ENST00000528215 | (GA)3 |
| MSI2 | ENSG00000153944 | ENST00000579180 | (AT)3 |
| MTA2 | ENSG00000149480 | ENST00000524902 | (AGG)3 |
|  |  | ENST00000527204 |  |
| MTHFD1 | ENSG00000100714 | ENST00000554739 | (TA)3 |
|  |  | ENST00000554768 |  |
|  |  | ENST00000557539 |  |
| MTMR9 | ENSG00000104643 | ENST00000526292 | (AT)3 |
| MTUS2 | ENSG00000132938 | ENST00000542829 | (GCA)3 |
| MUC20 | ENSG00000176945 | ENST00000447234 | (GA)3 |
|  |  | ENST00000436408 |  |
|  |  | ENST00000445522 | (TG)3 |
| MUC6 | ENSG00000184956 | ENST00000421673 | (GCA)3 |
| MXRA7 | ENSG00000182534 | ENST00000592148 | (TG)3 |
|  |  |  | (GA)3 |
| MYL5 | ENSG00000215375 | ENST00000507804 | (TC)3 |
| MYLK | ENSG00000065534 | ENST00000508240 | (GA)3 |
| MYO15A | ENSG00000091536 | ENST00000205890 | (AG)3 |
|  |  | ENST00000615845 |  |
|  |  | ENST00000647165 |  |
| MYO9A | ENSG00000066933 | ENST00000566885 | (TA)3 |
| N4BP2L2 | ENSG00000244754 | ENST00000399396 | (AT)3 |
|  |  |  | (ATTT)3 |
|  |  | ENST00000505213 | (AT)3 |
|  |  |  | (ATTT)3 |
|  |  | ENST00000267068 | (AT)3 |
|  |  |  | (ATTT)3 |
| NAA15 | ENSG00000164134 | ENST00000515576 | (AT)3 |
| NAA38 | ENSG00000183011 | ENST00000575208 | (GC)3 |
|  |  | ENST00000576384 |  |
| NAIP | ENSG00000249437 | ENST00000523981 | (GT)3 |
| NANP | ENSG00000170191 | ENST00000304788 | (TC)3 |
| NAP1L3 | ENSG00000186310 | ENST00000373079 | (CG)3 |
| NBPF3 | ENSG00000142794 | ENST00000454000 | (TG)3 |
|  |  | ENST00000342104 |  |
|  |  | ENST00000318249 |  |
| NBPF4 | ENSG00000196427 | ENST00000415641 | (TC)3 |
| NBPF6 | ENSG00000186086 | ENST00000370040 |  |
|  |  | ENST00000495380 |  |
| NCAPD3 | ENSG00000151503 | ENST00000530396 | (GT)3 |
| NCAPH | ENSG00000121152 | ENST00000455200 |  |
| NCBP2 | ENSG00000114503 | ENST00000321256 | (CG)3 |
| NCBP2L | ENSG00000170935 | ENST00000639756 | (GTG)3 |
| NCDN | ENSG00000020129 | ENST00000373253 | (TCCC)3 |
|  |  | ENST00000437806 |  |
| NCKAP1L | ENSG00000123338 | ENST00000545638 | (TA)3 |
| NDRG4 | ENSG00000103034 | ENST00000569539 | (GT)3 |
|  |  | ENST00000567063 |  |
|  |  | ENST00000563978 | (TC)3 |
|  |  | ENST00000569923 | (GT)3 |
|  |  | ENST00000562731 |  |
|  |  | ENST00000568424 | (TC)3 |
|  |  | ENST00000566656 | (GT)3 |
|  |  | ENST00000566618 |  |
| NDUFA10 | ENSG00000130414 | ENST00000252711 | (GA)3 |
| NDUFA3 | ENSG00000170906 | ENST00000303553 | (TC)3 |
| NDUFA5 | ENSG00000128609 | ENST00000470123 | (GA)3 |
| NDUFAF3 | ENSG00000178057 | ENST00000326912 | (TC)3 |
|  |  | ENST00000395458 |  |
|  |  | ENST00000451378 |  |
| NDUFB2 | ENSG00000090266 | ENST00000482954 | (GGT)3 |
|  |  | ENST00000460088 |  |
|  |  | ENST00000472695 |  |
|  |  | ENST00000476470 |  |
| NDUFB9 | ENSG00000147684 | ENST00000276689 | (CG)3 |
|  |  | ENST00000518008 |  |
|  |  | ENST00000522532 |  |
|  |  | ENST00000517367 |  |
| NECAB3 | ENSG00000125967 | ENST00000480994 | (AC)3 |
| NEDD9 | ENSG00000111859 | ENST00000513989 | (TC)3 |
| NEK5 | ENSG00000197168 | ENST00000355568 | (GT)3 |
| NFATC1 | ENSG00000131196 | ENST00000253506 | (GC)3 |
|  |  | ENST00000591814 |  |
| NFATC4 | ENSG00000100968 | ENST00000413692 | (AG)3 |
|  |  | ENST00000554591 |  |
|  |  | ENST00000557674 | (CT)3 |
|  |  | ENST00000556169 |  |
|  |  | ENST00000422617 |  |
|  |  | ENST00000555453 |  |
|  |  | ENST00000554473 |  |
|  |  | ENST00000556759 |  |
|  |  | ENST00000555167 |  |
| NFIC | ENSG00000141905 | ENST00000641145 | (TG)3 |
| NFIL3 | ENSG00000165030 | ENST00000297689 | (AC)3 |
| NFKBIA | ENSG00000100906 | ENST00000557389 | (CT)3 |
| NHLRC3 | ENSG00000188811 | ENST00000470258 | (TA)3 |
| NHSL1 | ENSG00000135540 | ENST00000533765 | (GAA)3 |
| NLRP4 | ENSG00000160505 | ENST00000587891 | (GT)3 |
| NOMO2 | ENSG00000185164 | ENST00000330537 | (AGG)3 |
| NOMO3 | ENSG00000103226 | ENST00000399336 |  |
| NOS1AP | ENSG00000198929 | ENST00000493151 | (CTT)3 |
| NOXA1 | ENSG00000188747 | ENST00000341349 | (CGC)3 |
|  |  |  | (GGCCCC)3 |
|  |  | ENST00000392815 | (CGC)3 |
|  |  |  | (GGCCCC)3 |
| NQO1 | ENSG00000181019 | ENST00000564043 | (GT)3 |
| NRG1 | ENSG00000157168 | ENST00000520407 | (AGG)3 |
| NRG3 | ENSG00000185737 | ENST00000372142 | (TG)3 |
| NSF | ENSG00000073969 | ENST00000576040 | (AG)3 |
|  |  | ENST00000571172 |  |
|  |  | ENST00000575068 |  |
| NUDC | ENSG00000090273 | ENST00000435827 | (CT)3 |
| NUDT9 | ENSG00000170502 | ENST00000512216 | (AT)3 |
|  |  | ENST00000473942 |  |
| NUP155 | ENSG00000113569 | ENST00000381843 | (AC)3 |
| NVL | ENSG00000143748 | ENST00000469968 | (TGA)3 |
|  |  | ENST00000391875 |  |
| NXN | ENSG00000167693 | ENST00000537628 | (GT)3 |
| OAZ1 | ENSG00000104904 | ENST00000602676 | (GA)3 |
|  |  | ENST00000582888 |  |
| OBSCN | ENSG00000154358 | ENST00000284548 | (TG)3 |
|  |  | ENST00000422127 |  |
|  |  | ENST00000570156 |  |
| OCM | ENSG00000122543 | ENST00000416608 | (AC)3 |
| OLFM1 | ENSG00000130558 | ENST00000371796 | (TC)3 |
|  |  | ENST00000539877 | (TG)3 |
| ONECUT3 | ENSG00000205922 | ENST00000382349 | (CG)3 |
| OPA1 | ENSG00000198836 | ENST00000643329 | (AC)3 |
|  |  | ENST00000419435 |  |
| OR10G4 | ENSG00000254737 | ENST00000641722 | (AG)3 |
|  |  | ENST00000641521 |  |
| OR10G8 | ENSG00000234560 | ENST00000641224 | (CA)3 |
|  |  |  | (AG)3 |
| OR10H5 | ENSG00000172519 | ENST00000642092 | (CT)3 |
| OR10K2 | ENSG00000180708 | ENST00000641042 | (TC)3 |
| OR11A1 | ENSG00000204694 | ENST00000377148 | (GTTTT)3 |
|  |  | ENST00000641152 |  |
|  |  | ENST00000377149 |  |
| OR13A1 | ENSG00000256574 | ENST00000374401 | (TC)3 |
|  |  | ENST00000553795 |  |
| OR14A16 | ENSG00000196772 | ENST00000641093 | (AT)3 |
| OR14J1 | ENSG00000204695 | ENST00000641895 | (AC)3 |
|  |  |  | (AT)3 |
| OR1A1 | ENSG00000172146 | ENST00000641322 | (AC)3 |
|  |  | ENST00000641732 |  |
| OR1I1 | ENSG00000094661 | ENST00000641398 | (TG)3 |
| OR1L6 | ENSG00000171459 | ENST00000304720 | (TC)3 |
| OR1N2 | ENSG00000171501 | ENST00000373688 | (CA)3 |
|  |  |  | (AATA)3 |
| OR2A5 | ENSG00000221836 | ENST00000641693 | (TG)3 |
| OR2AP1 | ENSG00000179615 | ENST00000641114 | (CT)3 |
| OR2B2 | ENSG00000168131 | ENST00000303324 | (GT)3 |
| OR2J2 | ENSG00000204700 | ENST00000641417 | (CT)3 |
| OR2J3 | ENSG00000204701 | ENST00000641151 | (TG)3 |
|  |  | ENST00000641960 |  |
| OR2T10 | ENSG00000184022 | ENST00000642090 | (AC)3 |
| OR2T8 | ENSG00000177462 | ENST00000641945 | (CA)3 |
| OR2V1 | ENSG00000185372 | ENST00000642036 | (TC)3 |
|  |  | ENST00000641318 |  |
|  |  | ENST00000641551 |  |
| OR2V2 | ENSG00000182613 | ENST00000641492 | (CA)3 |
|  |  | ENST00000641791 |  |
| OR4D6 | ENSG00000166884 | ENST00000300127 | (AG)3 |
| OR4E2 | ENSG00000221977 | ENST00000641524 | (ATA)3 |
| OR4K17 | ENSG00000176230 | ENST00000641386 | (CT)3 |
|  |  | ENST00000641633 |  |
| OR51F2 | ENSG00000176925 | ENST00000641672 | (AG)3 |
| OR51G2 | ENSG00000176893 | ENST00000641926 | (CA)3 |
| OR51M1 | ENSG00000184698 | ENST00000642046 | (TGA)3 |
| OR52E4 | ENSG00000180974 | ENST00000641726 | (AG)3 |
| OR52K1 | ENSG00000196778 | ENST00000641528 | (AT)3 |
| OR5AS1 | ENSG00000181785 | ENST00000641320 | (AG)3 |
| OR5B2 | ENSG00000172365 | ENST00000641342 | (AT)3 |
| OR5K2 | ENSG00000231861 | ENST00000427338 | (AG)3 |
| OR5M10 | ENSG00000254834 | ENST00000526812 |  |
| OR5M3 | ENSG00000174937 | ENST00000641993 | (AATA)3 |
| OR5T2 | ENSG00000181718 | ENST00000641661 | (TA)3 |
| OR6A2 | ENSG00000184933 | ENST00000641196 | (CT)3 |
|  |  |  | (AG)3 |
| OR6C6 | ENSG00000188324 | ENST00000358433 | (AT)3 |
|  |  |  |  |
| OR6N1 | ENSG00000197403 | ENST00000641846 | (GA)3 |
| OR6Y1 | ENSG00000197532 | ENST00000641622 | (TGG)3 |
|  |  | ENST00000641282 |  |
| OR7A17 | ENSG00000185385 | ENST00000642123 | (GA)3 |
|  |  | ENST00000641113 |  |
| OR7C1 | ENSG00000127530 | ENST00000641666 | (GT)3 |
|  |  |  | (CT)3 |
|  |  | ENST00000642030 | (GT)3 |
|  |  |  | (CT)3 |
|  |  | ENST00000642000 | (GT)3 |
|  |  |  | (CT)3 |
|  |  | ENST00000248073 | (GT)3 |
|  |  |  | (CT)3 |
| OR7D2 | ENSG00000188000 | ENST00000642043 | (CCA)3 |
|  |  | ENST00000641288 |  |
| OR7G2 | ENSG00000170923 | ENST00000641081 | (GCT)3 |
|  |  |  | (TCA)3 |
| OR8A1 | ENSG00000196119 | ENST00000641670 | (AG)3 |
|  |  | ENST00000284287 |  |
|  |  | ENST00000642111 |  |
| OR8D4 | ENSG00000181518 | ENST00000641687 |  |
|  |  |  | (CT)3 |
| OR8G5 | ENSG00000255298 | ENST00000641992 | (AT)3 |
|  |  | ENST00000641707 |  |
| OR8J1 | ENSG00000172487 | ENST00000533152 | (CT)3 |
| OR9A2 | ENSG00000179468 | ENST00000350513 |  |
| OR9A4 | ENSG00000258083 | ENST00000641559 |  |
| OR9G4 | ENSG00000172457 | ENST00000641581 | (TG)3 |
|  |  | ENST00000641668 |  |
| ORC4 | ENSG00000115947 | ENST00000536575 | (TAT)3 |
| ORMDL2 | ENSG00000123353 | ENST00000552672 | (CA)3 |
|  |  | ENST00000550836 | (AC)3 |
| OSBP2 | ENSG00000184792 | ENST00000446658 | (GC)3 |
| OSBPL5 | ENSG00000021762 | ENST00000263650 | (TCCT)3 |
|  |  | ENST00000389989 |  |
|  |  | ENST00000348039 |  |
|  |  | ENST00000533234 |  |
|  |  | ENST00000526122 |  |
|  |  | ENST00000530372 |  |
|  |  | ENST00000533721 |  |
|  |  | ENST00000534157 |  |
| OSCP1 | ENSG00000116885 | ENST00000433045 | (CA)3 |
| OSR2 | ENSG00000164920 | ENST00000457907 | (AGG)3 |
| OXA1L | ENSG00000155463 | ENST00000431881 | (GA)3 |
| P2RX5 | ENSG00000083454 | ENST00000552276 | (GC)3 |
|  |  | ENST00000551178 |  |
|  |  | ENST00000547178 |  |
|  |  | ENST00000225328 |  |
|  |  | ENST00000345901 |  |
| PA2G4 | ENSG00000170515 | ENST00000551061 | (GT)3 |
|  |  |  | (TG)3 |
| PAAF1 | ENSG00000175575 | ENST00000541951 | (GT)3 |
|  |  | ENST00000504441 | (CT)3 |
|  |  | ENST00000543814 |  |
|  |  | ENST00000535604 | (GT)3 |
|  |  | ENST00000542293 |  |
|  |  | ENST00000536003 | (CT)3 |
|  |  | ENST00000544552 |  |
|  |  | ENST00000544909 | (AG)3 |
| PABPC3 | ENSG00000151846 | ENST00000281589 | (TG)3 |
| PABPC4L | ENSG00000254535 | ENST00000421491 |  |
| PACRGL | ENSG00000163138 | ENST00000510051 | (CTCC)3 |
|  |  | ENST00000510700 |  |
|  |  | ENST00000509625 |  |
| PACS1 | ENSG00000175115 | ENST00000533756 | (TG)3 |
| PANK2 | ENSG00000125779 | ENST00000495692 | (GGA)3 |
|  |  | ENST00000610179 | (CTG)3 |
| PAQR6 | ENSG00000160781 | ENST00000623241 | (AC)3 |
|  |  | ENST00000613336 |  |
|  |  | ENST00000540423 |  |
| PARD3 | ENSG00000148498 | ENST00000544292 | (GTTTT)3 |
| PARVB | ENSG00000188677 | ENST00000444029 | (AC)3 |
|  |  |  | (ATTTC)3 |
| PAX9 | ENSG00000198807 | ENST00000554201 | (CGG)3 |
| PCBP3 | ENSG00000183570 | ENST00000449640 | (AGC)3 |
| PCDH1 | ENSG00000156453 | ENST00000287008 | (AGCCGG)3 |
|  |  | ENST00000503492 |  |
|  |  | ENST00000394536 |  |
| PCDHA1 | ENSG00000204970 | ENST00000378133 | (GA)3 |
| PCDHA5 | ENSG00000204965 | ENST00000614258 | (TA)3 |
|  |  |  | (TG)3 |
|  |  | ENST00000529619 | (TA)3 |
|  |  |  | (TG)3 |
| PCDHA7 | ENSG00000204963 | ENST00000356878 | (CA)3 |
| PCDHA9 | ENSG00000204961 | ENST00000532602 | (AGA)3 |
|  |  | ENST00000378122 |  |
| PCDHB1 | ENSG00000171815 | ENST00000306549 | (AG)3 |
| PCDHB14 | ENSG00000120327 | ENST00000624896 | (TG)3 |
| PCDHGC4 | ENSG00000242419 | ENST00000610539 |  |
| PCLO | ENSG00000186472 | ENST00000618073 | (TGGAA)3 |
| PCSK6 | ENSG00000140479 | ENST00000611716 | (GGC)3 |
|  |  | ENST00000331826 |  |
| PDCD2L | ENSG00000126249 | ENST00000246535 | (AG)3 |
| PDCD6 | ENSG00000249915 | ENST00000264933 | (TC)3 |
|  |  | ENST00000618970 |  |
|  |  | ENST00000628729 |  |
|  |  | ENST00000505221 |  |
|  |  | ENST00000509581 |  |
|  |  | ENST00000507528 |  |
| PDCL3 | ENSG00000115539 | ENST00000416255 | (TG)3 |
| PDE11A | ENSG00000128655 | ENST00000409504 | (CT)3 |
| PDE1C | ENSG00000154678 | ENST00000396191 | (CG)3 |
|  |  | ENST00000321453 |  |
|  |  | ENST00000396184 |  |
|  |  | ENST00000396182 |  |
|  |  | ENST00000396189 |  |
| PDE2A | ENSG00000186642 | ENST00000540345 | (CA)3 |
|  |  | ENST00000542223 | (TC)3 |
| PDE4D | ENSG00000113448 | ENST00000340635 | (GCAGCA)3 |
|  |  | ENST00000360047 | (AG)3 |
|  |  | ENST00000507116 | (CAG)3 |
|  |  | ENST00000502575 |  |
| PDE8B | ENSG00000113231 | ENST00000503963 | (TA)3 |
|  |  |  | (CT)3 |
|  |  |  | (TA)3 |
| PDGFRA | ENSG00000134853 | ENST00000512143 | (CAC)3 |
| PDHA1 | ENSG00000131828 | ENST00000379804 | (CTAA)3 |
| PDHX | ENSG00000110435 | ENST00000448838 | (GC)3 |
| PDIA5 | ENSG00000065485 | ENST00000484644 | (AG)3 |
| PDIA6 | ENSG00000143870 | ENST00000404371 | (GA)3 |
| PDK1 | ENSG00000152256 | ENST00000443353 | (CT)3 |
|  |  | ENST00000439519 |  |
| PDLIM4 | ENSG00000131435 | ENST00000253754 | (CTC)3 |
| PDSS1 | ENSG00000148459 | ENST00000376215 | (GCC)3 |
| PEA15 | ENSG00000162734 | ENST00000368076 | (AG)3 |
| PEBP4 | ENSG00000134020 | ENST00000522278 | (GC)3 |
| PECR | ENSG00000115425 | ENST00000265322 | (CCGC)3 |
| PEG10 | ENSG00000242265 | ENST00000482108 | (GT)3 |
|  |  | ENST00000613043 |  |
|  |  | ENST00000617526 |  |
| PELI2 | ENSG00000139946 | ENST00000267460 | (CGG)3 |
| PEMT | ENSG00000133027 | ENST00000395782 | (GT)3 |
|  |  | ENST00000395783 |  |
| PFN1 | ENSG00000108518 | ENST00000574872 | (TA)3 |
| PGA3 | ENSG00000229859 | ENST00000325558 |  |
|  |  |  | (TGC)3 |
|  |  | ENST00000543505 | (AG)3 |
| PGA4 | ENSG00000229183 | ENST00000378149 | (TA)3 |
|  |  |  | (TGC)3 |
| PGA5 | ENSG00000256713 | ENST00000312403 | (TA)3 |
|  |  |  | (TGC)3 |
|  |  | ENST00000451616 | (TG)3 |
| PGAP3 | ENSG00000161395 | ENST00000584856 | (CT)3 |
| PGRMC2 | ENSG00000164040 | ENST00000394276 | (TC)3 |
|  |  | ENST00000512483 |  |
|  |  | ENST00000503872 |  |
|  |  | ENST00000503588 | (CT)3 |
| PHF12 | ENSG00000109118 | ENST00000583747 | (GA)3 |
| PHF3 | ENSG00000118482 | ENST00000514822 | (CT)3 |
| PID1 | ENSG00000153823 | ENST00000392054 | (GA)3 |
| PIGG | ENSG00000174227 | ENST00000510235 | (AT)3 |
| PIGZ | ENSG00000119227 | ENST00000412723 |  |
|  |  | ENST00000443835 |  |
| PIH1D1 | ENSG00000104872 | ENST00000601807 | (GCT)3 |
|  |  | ENST00000597415 |  |
|  |  | ENST00000595550 |  |
| PIK3AP1 | ENSG00000155629 | ENST00000371109 | (GA)3 |
| PIK3C2G | ENSG00000139144 | ENST00000535651 | (AT)3 |
|  |  | ENST00000538779 |  |
|  |  | ENST00000433979 |  |
| PIM1 | ENSG00000137193 | ENST00000373509 | (CAGCCA)3 |
| PIPOX | ENSG00000179761 | ENST00000466889 | (TC)3 |
|  |  | ENST00000469082 |  |
| PITHD1 | ENSG00000057757 | ENST00000246151 | (CGAGC)3 |
|  |  | ENST00000415372 | (CA)3 |
| PITPNB | ENSG00000180957 | ENST00000415296 | (TC)3 |
| PKD2 | ENSG00000118762 | ENST00000508588 | (TA)3 |
| PLA2G4D | ENSG00000159337 | ENST00000290472 | (AG)3 |
| PLAGL1 | ENSG00000118495 | ENST00000437412 | (CT)3 |
|  |  | ENST00000392307 | (CA)3 |
|  |  | ENST00000367572 | (CT)3 |
|  |  | ENST00000417959 |  |
|  |  | ENST00000628651 |  |
|  |  | ENST00000626294 |  |
|  |  | ENST00000626462 |  |
|  |  | ENST00000627449 |  |
|  |  | ENST00000626373 |  |
| PLAUR | ENSG00000011422 | ENST00000599892 | (CCTCT)3 |
|  |  | ENST00000602141 |  |
| PLCB1 | ENSG00000182621 | ENST00000378641 | (GCGCCCC)3 |
|  |  | ENST00000338037 |  |
|  |  | ENST00000629992 |  |
|  |  | ENST00000404098 |  |
| PLCH2 | ENSG00000149527 | ENST00000419816 | (GA)3 |
|  |  | ENST00000378486 |  |
| PLCZ1 | ENSG00000139151 | ENST00000539072 | (TG)3 |
| PLEC | ENSG00000178209 | ENST00000354958 | (TGG)3 |
| PLEKHG4B | ENSG00000153404 | ENST00000283426 | (TG)3 |
| PLEKHG5 | ENSG00000171680 | ENST00000377748 |  |
|  |  |  | (AG)3 |
|  |  | ENST00000537245 |  |
|  |  | ENST00000377740 | (TG)3 |
|  |  |  | (AG)3 |
| PLEKHG6 | ENSG00000008323 | ENST00000449001 | (TG)3 |
|  |  |  | (CA)3 |
| PLEKHM2 | ENSG00000116786 | ENST00000375799 | (GGTGGC)3 |
|  |  | ENST00000375793 |  |
| PLGLB1 | ENSG00000183281 | ENST00000409310 | (AC)3 |
|  |  | ENST00000355705 |  |
| PLGLB2 | ENSG00000125551 | ENST00000359481 |  |
| PLIN3 | ENSG00000105355 | ENST00000589034 | (TC)3 |
| PLOD2 | ENSG00000152952 | ENST00000469350 | (AG)3 |
| PLXNC1 | ENSG00000136040 | ENST00000550080 | (CT)3 |
|  |  |  | (GTTTTT)3 |
|  |  | ENST00000547057 | (CT)3 |
|  |  |  | (GTTTTT)3 |
|  |  | ENST00000545312 | (CT)3 |
| PMM2 | ENSG00000140650 | ENST00000566983 | (TG)3 |
| PNCK | ENSG00000130822 | ENST00000340888 | (GC)3 |
|  |  | ENST00000370150 |  |
|  |  | ENST00000370142 |  |
|  |  | ENST00000439087 |  |
|  |  | ENST00000419804 |  |
|  |  | ENST00000458354 |  |
|  |  | ENST00000418241 |  |
|  |  | ENST00000434652 |  |
|  |  | ENST00000425526 |  |
|  |  | ENST00000423545 |  |
| POC1B | ENSG00000139323 | ENST00000549035 | (AC)3 |
| POFUT2 | ENSG00000186866 | ENST00000615172 | (GGCG)3 |
| POLR3E | ENSG00000058600 | ENST00000565358 | (GA)3 |
| POM121C | ENSG00000272391 | ENST00000439629 | (AG)3 |
| PPEF1 | ENSG00000086717 | ENST00000472826 | (ACCCCC)3 |
| PPHLN1 | ENSG00000134283 | ENST00000256678 | (TA)3 |
|  |  | ENST00000551658 | (AC)3 |
| PPM1L | ENSG00000163590 | ENST00000497343 | (TGG)3 |
|  |  | ENST00000498165 |  |
|  |  | ENST00000295839 | (AG)3 |
| PPM1M | ENSG00000164088 | ENST00000409502 | (GA)3 |
| PPP1R12C | ENSG00000125503 | ENST00000263433 | (GCGG)3 |
| PPP1R14A | ENSG00000167641 | ENST00000587515 | (GAGG)3 |
| PPP1R14B | ENSG00000173457 | ENST00000392210 | (TC)3 |
|  |  |  |  |
|  |  | ENST00000542235 |  |
| PPP2CA | ENSG00000113575 | ENST00000523082 | (CTA)3 |
| PRAMEF13 | ENSG00000279169 | ENST00000638454 | (GT)3 |
| PRKCH | ENSG00000027075 | ENST00000556778 | (AT)3 |
|  |  | ENST00000555542 |  |
|  |  | ENST00000555906 |  |
|  |  | ENST00000555082 |  |
|  |  | ENST00000553831 |  |
|  |  | ENST00000553265 |  |
|  |  | ENST00000556164 |  |
|  |  | ENST00000557585 |  |
|  |  | ENST00000557473 |  |
| PRKCZ | ENSG00000067606 | ENST00000378567 | (CGG)3 |
|  |  | ENST00000468310 |  |
|  |  | ENST00000486681 | (TC)3 |
|  |  | ENST00000497183 |  |
| PRKRIP1 | ENSG00000128563 | ENST00000462601 | (CAT)3 |
| PRMT5 | ENSG00000100462 | ENST00000554910 | (CTG)3 |
| PROS1 | ENSG00000184500 | ENST00000407433 | (AC)3 |
|  |  | ENST00000472684 |  |
| PRPS1 | ENSG00000147224 | ENST00000372428 | (CA)3 |
| PRR18 | ENSG00000176381 | ENST00000322583 | (CT)3 |
| PRR20A | ENSG00000204919 | ENST00000377931 |  |
| PRR20C | ENSG00000229665 | ENST00000614894 |  |
| PRR20D | ENSG00000227151 | ENST00000452123 |  |
| PRR20E | ENSG00000234278 | ENST00000434815 |  |
| PRSS23 | ENSG00000150687 | ENST00000527521 | (CTG)3 |
|  |  | ENST00000280258 |  |
| PRSS3 | ENSG00000010438 | ENST00000379405 | (CCA)3 |
| PRSS50 | ENSG00000206549 | ENST00000460241 | (GA)3 |
| PRUNE2 | ENSG00000106772 | ENST00000376717 | (AG)3 |
|  |  | ENST00000223609 | (GTT)3 |
|  |  |  | (GCTTT)3 |
| PRY | ENSG00000169789 | ENST00000303728 | (AG)3 |
| PRY2 | ENSG00000169807 | ENST00000303804 |  |
| PSD | ENSG00000059915 | ENST00000461698 | (GC)3 |
| PSD3 | ENSG00000156011 | ENST00000519851 | (CT)3 |
| PSEN2 | ENSG00000143801 | ENST00000472139 | (AC)3 |
| PSG1 | ENSG00000231924 | ENST00000436291 | (AG)3 |
|  |  |  | (AC)3 |
|  |  | ENST00000595124 | (AG)3 |
|  |  |  | (AC)3 |
|  |  | ENST00000595356 | (AG)3 |
|  |  |  | (AC)3 |
|  |  | ENST00000403380 | (AG)3 |
|  |  |  | (AC)3 |
|  |  | ENST00000312439 | (AG)3 |
|  |  |  | (AC)3 |
|  |  | ENST00000244296 | (AG)3 |
|  |  |  | (AC)3 |
| PSG4 | ENSG00000243137 | ENST00000405312 |  |
|  |  | ENST00000433626 |  |
|  |  | ENST00000244295 |  |
|  |  | ENST00000596907 |  |
| PSG5 | ENSG00000204941 | ENST00000366175 | (GA)3 |
|  |  | ENST00000342951 |  |
| PSG6 | ENSG00000170848 | ENST00000187910 | (AC)3 |
|  |  | ENST00000402603 |  |
|  |  | ENST00000292125 |  |
|  |  | ENST00000594375 |  |
| PSG8 | ENSG00000124467 | ENST00000404209 |  |
|  |  | ENST00000406636 |  |
|  |  | ENST00000401467 |  |
|  |  | ENST00000306511 |  |
| PSG9 | ENSG00000183668 | ENST00000418820 |  |
|  |  | ENST00000621109 |  |
|  |  | ENST00000593948 |  |
|  |  | ENST00000270077 |  |
|  |  | ENST00000443718 |  |
|  |  | ENST00000244293 |  |
| PSMA5 | ENSG00000143106 | ENST00000538610 | (TA)3 |
| PSMB2 | ENSG00000126067 | ENST00000621781 | (CCT)3 |
| PSMB9 | ENSG00000240065 | ENST00000395330 | (CT)3 |
|  |  | ENST00000414474 |  |
| PSMC1 | ENSG00000100764 | ENST00000543772 | (AG)3 |
| PSMD4 | ENSG00000159352 | ENST00000437736 | (GA)3 |
|  |  |  | (TC)3 |
| PTCD1 | ENSG00000106246 | ENST00000292478 | (TTCT)3 |
|  |  |  | (CT)3 |
|  |  |  | (AT)3 |
|  |  | ENST00000430982 | (TTCT)3 |
|  |  |  | (CT)3 |
|  |  |  | (AT)3 |
|  |  | ENST00000430029 | (TTCT)3 |
|  |  |  | (CT)3 |
|  |  |  | (AT)3 |
|  |  | ENST00000419981 | (TTCT)3 |
|  |  |  | (CT)3 |
|  |  |  | (AT)3 |
| PTCD2 | ENSG00000049883 | ENST00000510676 | (TTC)3 |
| PTGES2 | ENSG00000148334 | ENST00000617202 | (CT)3 |
|  |  | ENST00000277462 |  |
| PTGIR | ENSG00000160013 | ENST00000597185 | (CAG)3 |
|  |  | ENST00000594275 | (CT)3 |
| PTK2 | ENSG00000169398 | ENST00000430260 | (TC)3 |
|  |  | ENST00000522424 |  |
|  |  | ENST00000521562 |  |
|  |  | ENST00000523388 |  |
| PTPN2 | ENSG00000175354 | ENST00000591497 | (GA)3 |
| PTPRJ | ENSG00000149177 | ENST00000613246 | (GGA)3 |
|  |  | ENST00000418331 |  |
|  |  | ENST00000615445 |  |
|  |  | ENST00000440289 |  |
|  |  | ENST00000534219 |  |
|  |  | ENST00000527952 |  |
| PTPRM | ENSG00000173482 | ENST00000580170 | (CG)3 |
|  |  |  | (CCG)3 |
|  |  | ENST00000332175 | (CG)3 |
|  |  |  | (CCG)3 |
| PTPRN2 | ENSG00000155093 | ENST00000389416 | (CG)3 |
| PTPRT | ENSG00000196090 | ENST00000373190 |  |
|  |  |  | (GCC)3 |
|  |  | ENST00000373198 | (CG)3 |
|  |  |  | (GCC)3 |
|  |  | ENST00000373201 | (CG)3 |
|  |  |  | (GCC)3 |
|  |  | ENST00000373193 | (CG)3 |
|  |  |  | (GCC)3 |
| PUM1 | ENSG00000134644 | ENST00000373741 | (CG)3 |
| PUS1 | ENSG00000177192 | ENST00000443358 | (GCCCA)3 |
|  |  | ENST00000322060 |  |
|  |  | ENST00000538037 |  |
|  |  | ENST00000456665 |  |
|  |  | ENST00000535067 |  |
|  |  | ENST00000537484 |  |
| RAB15 | ENSG00000139998 | ENST00000554593 | (CTTT)3 |
|  |  | ENST00000646728 |  |
| RAB17 | ENSG00000124839 | ENST00000409822 | (GGA)3 |
| RABAC1 | ENSG00000105404 | ENST00000601078 | (CTC)3 |
| RABGAP1 | ENSG00000011454 | ENST00000616002 | (AC)3 |
| RACGAP1 | ENSG00000161800 | ENST00000548644 | (AT)3 |
| RAD21 | ENSG00000164754 | ENST00000523986 |  |
| RAD51C | ENSG00000108384 | ENST00000461271 | (CA)3 |
| RAD54L | ENSG00000085999 | ENST00000493985 | (CAG)3 |
|  |  |  | (GT)3 |
|  |  | ENST00000493032 | (CAG)3 |
|  |  |  | (GT)3 |
| RANBP9 | ENSG00000010017 | ENST00000011619 | (GA)3 |
| RAP1GAP | ENSG00000076864 | ENST00000495204 | (GCG)3 |
| RB1CC1 | ENSG00000023287 | ENST00000517963 | (GGCG)3 |
|  |  |  | (GGC)3 |
| RBBP7 | ENSG00000102054 | ENST00000468092 | (GAA)3 |
| RBCK1 | ENSG00000125826 | ENST00000353660 | (CA)3 |
|  |  | ENST00000400247 |  |
| RBFOX1 | ENSG00000078328 | ENST00000570626 | (GC)3 |
| RBFOX3 | ENSG00000167281 | ENST00000580508 | (CG)3 |
|  |  |  | (GCC)3 |
| RCAN1 | ENSG00000159200 | ENST00000381135 | (AG)3 |
| RCC1 | ENSG00000180198 | ENST00000486790 | (GC)3 |
| RDH14 | ENSG00000240857 | ENST00000381249 | (GGC)3 |
| RESP18 | ENSG00000182698 | ENST00000392083 | (GA)3 |
| REXO1 | ENSG00000079313 | ENST00000170168 | (GCG)3 |
| REXO2 | ENSG00000076043 | ENST00000544827 | (AT)3 |
| RFX2 | ENSG00000087903 | ENST00000586806 | (CA)3 |
|  |  | ENST00000592473 | (TG)3 |
|  |  | ENST00000593241 |  |
| RFX8 | ENSG00000196460 | ENST00000646446 | (CCT)3 |
|  |  | ENST00000428343 |  |
| RGL4 | ENSG00000159496 | ENST00000290691 | (GA)3 |
|  |  |  | (TCC)3 |
|  |  | ENST00000423392 | (GA)3 |
|  |  |  | (TCC)3 |
| RGS22 | ENSG00000132554 | ENST00000520117 | (CCA)3 |
| RGS6 | ENSG00000182732 | ENST00000554782 | (GATG)3 |
| RHCE | ENSG00000188672 | ENST00000294413 | (GA)3 |
|  |  | ENST00000349320 | (CT)3 |
|  |  |  | (TG)3 |
| RHD | ENSG00000187010 | ENST00000328664 | (GA)3 |
|  |  | ENST00000622561 |  |
| RIC8B | ENSG00000111785 | ENST00000549643 | (AG)3 |
| RIMBP3B | ENSG00000274600 | ENST00000620804 | (CGG)3 |
| RIMBP3C | ENSG00000183246 | ENST00000433039 |  |
| RIMKLB | ENSG00000166532 | ENST00000537189 | (GC)3 |
|  |  |  | (CT)3 |
| RIT1 | ENSG00000143622 | ENST00000539040 | (TC)3 |
| RLN2 | ENSG00000107014 | ENST00000381627 | (GT)3 |
| RNASEH2A | ENSG00000104889 | ENST00000221486 | (TGG)3 |
| RNF111 | ENSG00000157450 | ENST00000559160 | (ATTT)3 |
| RNF130 | ENSG00000113269 | ENST00000521389 | (GCC)3 |
|  |  | ENST00000261947 |  |
| RNF138 | ENSG00000134758 | ENST00000578914 | (AG)3 |
| RNF150 | ENSG00000170153 | ENST00000306799 | (AGC)3 |
|  |  | ENST00000507500 |  |
| RNF40 | ENSG00000103549 | ENST00000565995 | (CAG)3 |
| RNF8 | ENSG00000112130 | ENST00000487950 | (TTG)3 |
| RNPS1 | ENSG00000205937 | ENST00000566397 | (CT)3 |
|  |  | ENST00000561718 |  |
| ROCK2 | ENSG00000134318 | ENST00000401753 | (TC)3 |
| RPA1 | ENSG00000132383 | ENST00000571058 | (TA)3 |
|  |  | ENST00000570451 |  |
| RPL10L | ENSG00000165496 | ENST00000298283 |  |
|  |  |  | (GC)3 |
|  |  |  | (AG)3 |
| RPL27A | ENSG00000166441 | ENST00000524496 | (GT)3 |
|  |  | ENST00000530022 |  |
|  |  | ENST00000526562 |  |
|  |  | ENST00000530913 |  |
| RPL3 | ENSG00000100316 | ENST00000401609 | (GCA)3 |
|  |  | ENST00000402527 |  |
| RPL30 | ENSG00000156482 | ENST00000523172 | (ATTT)3 |
| RPL36AL | ENSG00000165502 | ENST00000298289 | (TA)3 |
| RPL7A | ENSG00000148303 | ENST00000426651 | (AG)3 |
| RPP38 | ENSG00000152464 | ENST00000378203 | (ATAA)3 |
|  |  |  | (AG)3 |
|  |  | ENST00000616640 | (ATAA)3 |
|  |  |  | (AG)3 |
|  |  | ENST00000378201 | (ATAA)3 |
|  |  |  | (AG)3 |
|  |  | ENST00000378202 | (ATAA)3 |
|  |  |  | (AG)3 |
|  |  | ENST00000378197 | (ATAA)3 |
|  |  |  | (AG)3 |
|  |  | ENST00000441850 | (ATAA)3 |
|  |  |  | (AG)3 |
| RPRML | ENSG00000179673 | ENST00000322329 | (GC)3 |
| RPS15 | ENSG00000115268 | ENST00000586686 | (GA)3 |
|  |  | ENST00000586656 |  |
|  |  | ENST00000591804 |  |
|  |  | ENST00000617694 |  |
| RRAGD | ENSG00000025039 | ENST00000359203 | (TC)3 |
| RRAS2 | ENSG00000133818 | ENST00000531807 | (GA)3 |
| RSAD2 | ENSG00000134321 | ENST00000442639 | (CTC)3 |
| RSL1D1 | ENSG00000171490 | ENST00000573791 | (CT)3 |
| RSPH10B | ENSG00000155026 | ENST00000539903 | (TA)3 |
| RSPH6A | ENSG00000104941 | ENST00000221538 | (TC)3 |
| RSPO2 | ENSG00000147655 | ENST00000517939 | (TTC)3 |
|  |  | ENST00000521502 |  |
|  |  | ENST00000521757 |  |
| RSRC2 | ENSG00000111011 | ENST00000526560 | (AG)3 |
| RTTN | ENSG00000176225 | ENST00000578780 | (GA)3 |
| RWDD4 | ENSG00000182552 | ENST00000510968 | (CT)3 |
|  |  | ENST00000506467 | (TA)3 |
| S100A1 | ENSG00000160678 | ENST00000368698 | (AC)3 |
| SACM1L | ENSG00000211456 | ENST00000433336 | (TC)3 |
| SAGE1 | ENSG00000181433 | ENST00000324447 | (AT)3 |
|  |  |  | (AC)3 |
|  |  | ENST00000537770 | (AT)3 |
|  |  |  | (AC)3 |
| SALL3 | ENSG00000256463 | ENST00000536229 | (GC)3 |
| SAMD1 | ENSG00000141858 | ENST00000533683 |  |
| SAP30BP | ENSG00000161526 | ENST00000584240 | (TC)3 |
|  |  | ENST00000583063 |  |
| SCAND1 | ENSG00000171222 | ENST00000305978 | (CA)3 |
|  |  | ENST00000615116 |  |
|  |  | ENST00000373991 |  |
| SCARB2 | ENSG00000138760 | ENST00000638295 | (GA)3 |
| SCFD1 | ENSG00000092108 | ENST00000544052 | (TC)3 |
|  |  | ENST00000557076 | (ATG)3 |
| SCGB1A1 | ENSG00000149021 | ENST00000534397 | (TC)3 |
| SCNN1D | ENSG00000162572 | ENST00000470022 | (AG)3 |
|  |  |  | (CA)3 |
|  |  |  |  |
|  |  | ENST00000325425 | (AG)3 |
|  |  |  | (CA)3 |
|  |  |  |  |
| SCYL2 | ENSG00000136021 | ENST00000548392 | (ATT)3 |
| SDC3 | ENSG00000162512 | ENST00000336798 | (AG)3 |
|  |  | ENST00000339394 | (CGC)3 |
|  |  |  | (CGCC)3 |
|  |  |  | (CGC)3 |
| SEC14L2 | ENSG00000100003 | ENST00000429917 | (GT)3 |
|  |  | ENST00000415072 | (CT)3 |
| SEC16A | ENSG00000148396 | ENST00000313084 | (GT)3 |
| SEC24B | ENSG00000138802 | ENST00000504968 | (CT)3 |
| SEC61B | ENSG00000106803 | ENST00000498603 |  |
| SEMA6B | ENSG00000167680 | ENST00000586582 | (CCT)3 |
| SERF2 | ENSG00000140264 | ENST00000409291 | (GT)3 |
|  |  | ENST00000402131 |  |
|  |  | ENST00000403425 |  |
|  |  | ENST00000430901 |  |
|  |  | ENST00000409614 |  |
| SERHL2 | ENSG00000183569 | ENST00000447870 | (TC)3 |
| SERPINA1 | ENSG00000197249 | ENST00000554720 | (TCT)3 |
| SERPINA9 | ENSG00000170054 | ENST00000448305 |  |
|  |  | ENST00000546329 | (AT)3 |
| SERPINB3 | ENSG00000057149 | ENST00000283752 |  |
| SERPINB4 | ENSG00000206073 | ENST00000341074 |  |
|  |  | ENST00000436264 |  |
| SET | ENSG00000119335 | ENST00000409104 | (GAG)3 |
|  |  | ENST00000322030 | (TC)3 |
| SETD2 | ENSG00000181555 | ENST00000638947 | (AG)3 |
| SETMAR | ENSG00000170364 | ENST00000358065 | (CG)3 |
| SFN | ENSG00000175793 | ENST00000339276 | (GA)3 |
|  |  |  | (TG)3 |
| SFRP1 | ENSG00000104332 | ENST00000379845 | (AG)3 |
| SGK3 | ENSG00000104205 | ENST00000521960 | (TG)3 |
| SH2D4A | ENSG00000104611 | ENST00000518040 | (CCCT)3 |
| SH3GL3 | ENSG00000140600 | ENST00000324537 | (CT)3 |
| SHANK2 | ENSG00000162105 | ENST00000601538 | (GT)3 |
| SHC4 | ENSG00000185634 | ENST00000396535 | (GA)3 |
|  |  | ENST00000537958 | (TA)3 |
|  |  | ENST00000558220 |  |
| SIAH2 | ENSG00000181788 | ENST00000482706 | (GT)3 |
| SIGIRR | ENSG00000185187 | ENST00000528058 | (CT)3 |
| SIRPB1 | ENSG00000101307 | ENST00000563840 | (TC)3 |
| SIRT6 | ENSG00000077463 | ENST00000594279 | (CT)3 |
|  |  |  |  |
|  |  |  | (TC)3 |
| SKAP1 | ENSG00000141293 | ENST00000579336 | (AG)3 |
|  |  |  | (TC)3 |
| SKOR1 | ENSG00000188779 | ENST00000554240 | (GC)3 |
|  |  |  | (CGG)3 |
| SLAMF9 | ENSG00000162723 | ENST00000368092 | (ACTG)3 |
| SLC11A2 | ENSG00000110911 | ENST00000546743 | (CT)3 |
|  |  | ENST00000547579 |  |
|  |  | ENST00000547732 |  |
| SLC12A9 | ENSG00000146828 | ENST00000418037 | (TG)3 |
| SLC16A11 | ENSG00000174326 | ENST00000447225 | (TC)3 |
| SLC16A2 | ENSG00000147100 | ENST00000587091 | (GGCAGC)3 |
| SLC1A4 | ENSG00000115902 | ENST00000531327 | (CT)3 |
| SLC22A1 | ENSG00000175003 | ENST00000540443 | (TC)3 |
| SLC22A16 | ENSG00000004809 | ENST00000434949 | (AG)3 |
|  |  | ENST00000437378 |  |
|  |  | ENST00000424139 |  |
| SLC22A18AS | ENSG00000254827 | ENST00000625099 | (GA)3 |
| SLC24A3 | ENSG00000185052 | ENST00000328041 | (GCC)3 |
|  |  |  |  |
| SLC25A10 | ENSG00000183048 | ENST00000545862 | (CGCGGGG)3 |
|  |  | ENST00000331531 |  |
| SLC25A21 | ENSG00000183032 | ENST00000331299 | (CT)3 |
| SLC25A24 | ENSG00000085491 | ENST00000370041 | (GT)3 |
| SLC25A26 | ENSG00000144741 | ENST00000336733 | (AT)3 |
| SLC25A27 | ENSG00000153291 | ENST00000603486 | (TG)3 |
|  |  |  | (AG)3 |
| SLC25A29 | ENSG00000197119 | ENST00000359232 | (CG)3 |
|  |  | ENST00000392908 |  |
|  |  | ENST00000554060 |  |
| SLC26A10 | ENSG00000135502 | ENST00000320442 | (GA)3 |
| SLC27A5 | ENSG00000083807 | ENST00000594786 | (CT)3 |
| SLC2A14 | ENSG00000173262 | ENST00000542505 | (TTC)3 |
| SLC30A5 | ENSG00000145740 | ENST00000621204 | (AG)3 |
| SLC35F1 | ENSG00000196376 | ENST00000360388 | (CGC)3 |
| SLC38A10 | ENSG00000157637 | ENST00000539748 | (GT)3 |
|  |  |  | (CTG)3 |
| SLC38A6 | ENSG00000139974 | ENST00000526105 | (TG)3 |
| SLC3A1 | ENSG00000138079 | ENST00000409380 | (GT)3 |
| SLC3A2 | ENSG00000168003 | ENST00000539891 | (TC)3 |
|  |  | ENST00000536981 |  |
| SLC41A1 | ENSG00000133065 | ENST00000367137 | (AGA)3 |
| SLC43A3 | ENSG00000134802 | ENST00000529113 | (TG)3 |
| SLC4A4 | ENSG00000080493 | ENST00000639096 | (AGA)3 |
|  |  | ENST00000638464 | (CT)3 |
| SLC6A15 | ENSG00000072041 | ENST00000552192 | (GTT)3 |
| SLC6A9 | ENSG00000196517 | ENST00000475075 | (GAAAA)3 |
| SLC7A8 | ENSG00000092068 | ENST00000422941 | (TG)3 |
| SLC8A2 | ENSG00000118160 | ENST00000542837 | (AG)3 |
| SLC9A3R2 | ENSG00000065054 | ENST00000565855 |  |
|  |  | ENST00000566198 |  |
| SMARCD2 | ENSG00000108604 | ENST00000323347 | (GT)3 |
| SMC4 | ENSG00000113810 | ENST00000485867 | (TCC)3 |
| SMG1 | ENSG00000157106 | ENST00000330588 | (AT)3 |
| SNAP47 | ENSG00000143740 | ENST00000617596 | (CA)3 |
| SNRPC | ENSG00000124562 | ENST00000374018 | (AC)3 |
|  |  | ENST00000374017 | (GTTT)3 |
| SNX12 | ENSG00000147164 | ENST00000374274 | (CCG)3 |
| SNX19 | ENSG00000120451 | ENST00000528555 | (CT)3 |
|  |  | ENST00000530356 |  |
| SNX5 | ENSG00000089006 | ENST00000377759 | (CTT)3 |
|  |  | ENST00000377768 |  |
|  |  | ENST00000419004 |  |
|  |  | ENST00000606557 |  |
|  |  | ENST00000606602 |  |
|  |  | ENST00000481323 |  |
|  |  | ENST00000486039 |  |
| SORL1 | ENSG00000137642 | ENST00000525532 | (TG)3 |
| SOS1 | ENSG00000115904 | ENST00000451331 | (ATG)3 |
| SOX3 | ENSG00000134595 | ENST00000370536 | (TA)3 |
| SPACA3 | ENSG00000141316 | ENST00000580599 | (GTG)3 |
| SPACA7 | ENSG00000153498 | ENST00000443541 | (CT)3 |
| SPAG11A | ENSG00000178287 | ENST00000400125 | (CA)3 |
|  |  | ENST00000434307 |  |
|  |  | ENST00000642566 |  |
| SPDYE3 | ENSG00000214300 | ENST00000332397 | (AC)3 |
|  |  |  | (AGA)3 |
| SPHK2 | ENSG00000063176 | ENST00000340932 | (CT)3 |
| SPIB | ENSG00000269404 | ENST00000439922 | (TC)3 |
| SPIN2A | ENSG00000147059 | ENST00000374908 | (CT)3 |
|  |  | ENST00000374906 |  |
| SPINK13 | ENSG00000214510 | ENST00000511106 | (AG)3 |
| SPINK9 | ENSG00000204909 | ENST00000511717 | (TC)3 |
| SPIRE1 | ENSG00000134278 | ENST00000409402 | (CGA)3 |
|  |  | ENST00000410092 |  |
|  |  | ENST00000497844 | (AT)3 |
| SPOCK3 | ENSG00000196104 | ENST00000421836 | (CTTT)3 |
| SPRED3 | ENSG00000188766 | ENST00000587013 | (ACC)3 |
| SPTB | ENSG00000070182 | ENST00000556626 | (AG)3 |
|  |  | ENST00000644917 |  |
|  |  | ENST00000389721 |  |
|  |  | ENST00000389720 |  |
| SRD5A3 | ENSG00000128039 | ENST00000264228 | (CG)3 |
| SREBF1 | ENSG00000072310 | ENST00000395757 | (AC)3 |
|  |  | ENST00000577897 |  |
| SRGAP1 | ENSG00000196935 | ENST00000631006 | (TC)3 |
|  |  | ENST00000543397 |  |
| SRP68 | ENSG00000167881 | ENST00000307877 | (CGG)3 |
| SRPK3 | ENSG00000184343 | ENST00000489426 | (TC)3 |
|  |  | ENST00000370100 |  |
| SRRM5 | ENSG00000226763 | ENST00000607544 | (CA)3 |
| SRSF11 | ENSG00000116754 | ENST00000370949 |  |
| SS18L1 | ENSG00000184402 | ENST00000450482 | (ACC)3 |
| SSBP3 | ENSG00000157216 | ENST00000417664 | (CT)3 |
|  |  | ENST00000371320 | (GA)3 |
|  |  | ENST00000357475 |  |
|  |  | ENST00000525990 | (CT)3 |
| SSBP4 | ENSG00000130511 | ENST00000597724 | (TG)3 |
|  |  |  |  |
|  |  | ENST00000601357 | (AG)3 |
|  |  | ENST00000602088 |  |
| SSH1 | ENSG00000084112 | ENST00000326470 | (GAG)3 |
|  |  | ENST00000546697 | (GGA)3 |
| SSSCA1 | ENSG00000173465 | ENST00000531405 | (CT)3 |
|  |  | ENST00000527920 |  |
| SSX2IP | ENSG00000117155 | ENST00000603677 | (TG)3 |
|  |  |  | (GAA)3 |
| ST20 | ENSG00000180953 | ENST00000562759 | (AG)3 |
|  |  | ENST00000478497 |  |
|  |  | ENST00000485386 |  |
| ST3GAL4 | ENSG00000110080 | ENST00000524860 | (TCC)3 |
| ST6GALNAC3 | ENSG00000184005 | ENST00000621530 | (AT)3 |
| ST6GALNAC4 | ENSG00000136840 | ENST00000361444 | (CT)3 |
| ST7 | ENSG00000004866 | ENST00000421345 | (ATC)3 |
|  |  | ENST00000434836 |  |
|  |  | ENST00000477742 |  |
|  |  | ENST00000420755 |  |
| ST7L | ENSG00000007341 | ENST00000369669 | (CT)3 |
| STAR | ENSG00000147465 | ENST00000521236 | (TC)3 |
|  |  |  | (CT)3 |
| STAT3 | ENSG00000168610 | ENST00000389272 | (CTG)3 |
| STK3 | ENSG00000104375 | ENST00000523601 | (GA)3 |
|  |  |  | (CT)3 |
| STK32B | ENSG00000152953 | ENST00000512636 | (TC)3 |
|  |  | ENST00000510398 |  |
| STRA6 | ENSG00000137868 | ENST00000535552 | (CAG)3 |
|  |  | ENST00000563965 | (AC)3 |
|  |  | ENST00000574278 | (TG)3 |
| STX5 | ENSG00000162236 | ENST00000394690 | (TC)3 |
|  |  |  | (AGC)3 |
| STX6 | ENSG00000135823 | ENST00000542060 | (TAA)3 |
|  |  |  | (TG)3 |
| STX8 | ENSG00000170310 | ENST00000574431 | (AT)3 |
| STXBP1 | ENSG00000136854 | ENST00000637521 | (AG)3 |
|  |  | ENST00000625363 |  |
|  |  | ENST00000626539 |  |
|  |  | ENST00000637173 |  |
|  |  | ENST00000630492 |  |
|  |  | ENST00000626333 |  |
| STXBP2 | ENSG00000076944 | ENST00000414284 | (GGC)3 |
|  |  | ENST00000602355 | (GA)3 |
| SULT1A1 | ENSG00000196502 | ENST00000350842 | (CG)3 |
| SULT1A3 | ENSG00000261052 | ENST00000338971 | (GA)3 |
|  |  | ENST00000395138 |  |
| SULT1A4 | ENSG00000213648 | ENST00000360423 |  |
| SULT6B1 | ENSG00000138068 | ENST00000407963 | (CT)3 |
|  |  | ENST00000420611 |  |
|  |  | ENST00000416345 |  |
|  |  | ENST00000433192 |  |
| SYNE1 | ENSG00000131018 | ENST00000448038 | (TA)3 |
| SYNGR3 | ENSG00000127561 | ENST00000618464 | (ATCAGC)3 |
| SYT16 | ENSG00000139973 | ENST00000430451 | (AG)3 |
| SYTL2 | ENSG00000137501 | ENST00000359152 | (GT)3 |
|  |  | ENST00000389960 |  |
|  |  | ENST00000316356 |  |
|  |  | ENST00000528231 |  |
| TAF6 | ENSG00000106290 | ENST00000437822 | (TCC)3 |
| TAGLN3 | ENSG00000144834 | ENST00000486460 | (TC)3 |
| TARBP2 | ENSG00000139546 | ENST00000456234 | (AG)3 |
|  |  | ENST00000547064 | (TC)3 |
|  |  | ENST00000552817 |  |
|  |  | ENST00000394357 | (AG)3 |
| TARM1 | ENSG00000248385 | ENST00000616041 | (GAAA)3 |
| TAS2R1 | ENSG00000169777 | ENST00000514078 | (GTG)3 |
|  |  | ENST00000506620 |  |
| TAS2R9 | ENSG00000121381 | ENST00000240691 | (TCA)3 |
| TBC1D2 | ENSG00000095383 | ENST00000375063 | (CT)3 |
| TBC1D26 | ENSG00000214946 | ENST00000584301 | (GT)3 |
|  |  | ENST00000580596 |  |
|  |  | ENST00000612583 |  |
|  |  | ENST00000464963 |  |
|  |  | ENST00000437605 |  |
|  |  | ENST00000579428 |  |
|  |  | ENST00000578506 |  |
| TBC1D28 | ENSG00000189375 | ENST00000345096 |  |
|  |  | ENST00000405044 |  |
|  |  | ENST00000575220 |  |
|  |  | ENST00000572213 |  |
|  |  | ENST00000573652 |  |
|  |  | ENST00000575211 |  |
| TBC1D3 | ENSG00000274611 | ENST00000620215 | (CAGC)3 |
| TBC1D3B | ENSG00000274808 | ENST00000611257 |  |
| TBC1D3C | ENSG00000278299 | ENST00000622206 |  |
| TBC1D3F | ENSG00000275954 | ENST00000620210 |  |
| TBC1D3G | ENSG00000260287 | ENST00000569055 |  |
| TBC1D3H | ENSG00000274226 | ENST00000610350 |  |
| TBC1D5 | ENSG00000131374 | ENST00000429924 | (TA)3 |
|  |  |  | (TC)3 |
| TBCA | ENSG00000171530 | ENST00000522370 | (GA)3 |
| TBCC | ENSG00000124659 | ENST00000372876 |  |
| TBCD | ENSG00000141556 | ENST00000576160 | (GT)3 |
|  |  | ENST00000571712 | (TG)3 |
|  |  |  | (AC)3 |
|  |  | ENST00000576996 | (GT)3 |
|  |  | ENST00000576760 |  |
|  |  | ENST00000572984 |  |
|  |  | ENST00000574422 |  |
| TBL1Y | ENSG00000092377 | ENST00000383032 | (CA)3 |
|  |  | ENST00000355162 |  |
|  |  | ENST00000346432 |  |
| TBL3 | ENSG00000183751 | ENST00000615855 | (AC)3 |
| TBP | ENSG00000112592 | ENST00000540980 | (AGC)3 |
| TCF7L2 | ENSG00000148737 | ENST00000542695 | (CT)3 |
|  |  | ENST00000637574 | (GGA)3 |
| TCFL5 | ENSG00000101190 | ENST00000217162 | (CCG)3 |
| TCP10L | ENSG00000242220 | ENST00000472557 | (CAG)3 |
| TCP10L2 | ENSG00000166984 | ENST00000486697 | (AG)3 |
|  |  | ENST00000366832 |  |
| TCTN1 | ENSG00000204852 | ENST00000549123 | (TC)3 |
| TDP1 | ENSG00000042088 | ENST00000553617 | (TGA)3 |
| TEAD2 | ENSG00000074219 | ENST00000539846 | (CCT)3 |
|  |  | ENST00000596757 |  |
| TEAD3 | ENSG00000007866 | ENST00000402886 | (CTGT)3 |
|  |  | ENST00000338863 |  |
| TEX11 | ENSG00000120498 | ENST00000395889 | (CTTT)3 |
| TEX12 | ENSG00000150783 | ENST00000530752 | (AG)3 |
| TF | ENSG00000091513 | ENST00000466911 | (CT)3 |
| TGFBI | ENSG00000120708 | ENST00000508076 | (TA)3 |
| TGM1 | ENSG00000092295 | ENST00000544573 | (TG)3 |
| THAP6 | ENSG00000174796 | ENST00000507557 | (TA)3 |
|  |  | ENST00000508105 |  |
|  |  | ENST00000504190 |  |
|  |  | ENST00000507885 |  |
|  |  | ENST00000502620 |  |
| THOC5 | ENSG00000100296 | ENST00000455450 | (GCC)3 |
| THRB | ENSG00000151090 | ENST00000280696 | (TGTA)3 |
| TIA1 | ENSG00000116001 | ENST00000454815 | (TG)3 |
| TIMP2 | ENSG00000035862 | ENST00000585421 |  |
|  |  |  | (TCC)3 |
|  |  | ENST00000536189 | (TG)3 |
|  |  |  | (TCC)3 |
|  |  | ENST00000586057 | (TG)3 |
|  |  |  | (TCC)3 |
| TINAGL1 | ENSG00000142910 | ENST00000537531 | (GA)3 |
| TLR3 | ENSG00000164342 | ENST00000504367 | (CCA)3 |
| TLR6 | ENSG00000174130 | ENST00000436693 | (GAA)3 |
|  |  | ENST00000381950 |  |
|  |  | ENST00000508254 |  |
| TM4SF1 | ENSG00000169908 | ENST00000472441 | (TG)3 |
| TM9SF3 | ENSG00000077147 | ENST00000371142 | (GGA)3 |
|  |  | ENST00000443638 | (AC)3 |
|  |  |  | (GT)3 |
| TMBIM6 | ENSG00000139644 | ENST00000547798 | (GCA)3 |
| TMC6 | ENSG00000141524 | ENST00000589553 | (CT)3 |
| TMEM132B | ENSG00000139364 | ENST00000613307 | (TGT)3 |
| TMEM132E | ENSG00000181291 | ENST00000321639 | (CT)3 |
|  |  | ENST00000631683 |  |
| TMEM154 | ENSG00000170006 | ENST00000304385 | (AG)3 |
|  |  | ENST00000504064 |  |
| TMEM176A | ENSG00000002933 | ENST00000461345 | (TTCT)3 |
|  |  | ENST00000468689 |  |
| TMEM179 | ENSG00000258986 | ENST00000616017 | (GT)3 |
|  |  |  |  |
|  |  |  |  |
|  |  |  |  |
| TMEM182 | ENSG00000170417 | ENST00000639249 | (CA)3 |
|  |  | ENST00000409528 |  |
|  |  | ENST00000640575 |  |
| TMEM184A | ENSG00000164855 | ENST00000297477 | (CCTC)3 |
|  |  | ENST00000319010 |  |
|  |  | ENST00000414730 |  |
|  |  | ENST00000441933 |  |
| TMEM209 | ENSG00000146842 | ENST00000462753 | (GA)3 |
| TMEM30A | ENSG00000112697 | ENST00000370050 | (CT)3 |
| TMEM44 | ENSG00000145014 | ENST00000330115 |  |
| TMEM52 | ENSG00000178821 | ENST00000378602 | (GA)3 |
| TMEM63A | ENSG00000196187 | ENST00000537914 | (AC)3 |
| TMPPE | ENSG00000188167 | ENST00000416695 | (CAT)3 |
| TNFAIP8 | ENSG00000145779 | ENST00000388882 | (CT)3 |
| TNFRSF8 | ENSG00000120949 | ENST00000417814 | (GA)3 |
|  |  | ENST00000413146 | (GCA)3 |
| TNFSF13 | ENSG00000161955 | ENST00000483039 | (AC)3 |
| TNRC18 | ENSG00000182095 | ENST00000434361 | (TC)3 |
|  |  | ENST00000399434 |  |
| TNXB | ENSG00000168477 | ENST00000451343 | (AC)3 |
| TOR1AIP1 | ENSG00000143337 | ENST00000435319 | (GA)3 |
| TOX3 | ENSG00000103460 | ENST00000407228 | (AG)3 |
|  |  | ENST00000563091 | (CT)3 |
| TP53I11 | ENSG00000175274 | ENST00000525138 | (CAG)3 |
| TP53TG3 | ENSG00000183632 | ENST00000398682 | (GA)3 |
|  |  | ENST00000569420 |  |
| TP53TG3B | ENSG00000261509 | ENST00000341305 |  |
|  |  | ENST00000569741 |  |
| TPI1 | ENSG00000111669 | ENST00000488464 | (TG)3 |
|  |  | ENST00000535434 |  |
|  |  | ENST00000493987 |  |
|  |  | ENST00000462761 |  |
|  |  | ENST00000495834 |  |
| TPM3 | ENSG00000143549 | ENST00000302206 | (AG)3 |
|  |  |  | (AC)3 |
| TPM4 | ENSG00000167460 | ENST00000586499 | (CT)3 |
| TRAM1L1 | ENSG00000174599 | ENST00000310754 | (GGC)3 |
| TRAPPC2 | ENSG00000196459 | ENST00000458511 | (CT)3 |
| TRAPPC2L | ENSG00000167515 | ENST00000561840 | (CA)3 |
| TREX2 | ENSG00000183479 | ENST00000393862 | (TC)3 |
|  |  | ENST00000330912 |  |
|  |  | ENST00000338525 |  |
|  |  | ENST00000370231 |  |
| TRIM15 | ENSG00000204610 | ENST00000619857 | (AC)3 |
| TRIM29 | ENSG00000137699 | ENST00000526881 | (CT)3 |
| TRIM43 | ENSG00000144015 | ENST00000272395 | (TC)3 |
| TRIM65 | ENSG00000141569 | ENST00000269383 | (GC)3 |
|  |  |  | (CGC)3 |
| TRIM68 | ENSG00000167333 | ENST00000526337 | (AGC)3 |
| TRIM69 | ENSG00000185880 | ENST00000560442 | (GT)3 |
|  |  | ENST00000558329 |  |
| TRO | ENSG00000067445 | ENST00000411534 |  |
|  |  | ENST00000452830 |  |
|  |  | ENST00000430420 |  |
|  |  | ENST00000453081 |  |
|  |  | ENST00000449980 |  |
|  |  | ENST00000427099 |  |
| TRPM8 | ENSG00000144481 | ENST00000355722 |  |
|  |  | ENST00000409625 | (TCCT)3 |
|  |  |  | (TG)3 |
| TSEN54 | ENSG00000182173 | ENST00000434205 | (GCCCTCCCT)3 |
| TSFM | ENSG00000123297 | ENST00000434359 | (CA)3 |
|  |  | ENST00000457189 |  |
| TSKU | ENSG00000182704 | ENST00000533752 | (TG)3 |
|  |  | ENST00000612930 |  |
|  |  | ENST00000333090 |  |
|  |  | ENST00000525167 |  |
| TSNAXIP1 | ENSG00000102904 | ENST00000388833 | (GA)3 |
|  |  |  |  |
| TSPAN32 | ENSG00000064201 | ENST00000612299 |  |
|  |  | ENST00000451520 | (TC)3 |
|  |  |  | (GA)3 |
| TSPAN4 | ENSG00000214063 | ENST00000397396 | (CA)3 |
|  |  |  | (TCA)3 |
|  |  | ENST00000525334 | (CA)3 |
|  |  |  | (TCA)3 |
|  |  | ENST00000525201 | (CA)3 |
|  |  |  | (TCA)3 |
| TSPAN5 | ENSG00000168785 | ENST00000505184 | (TG)3 |
|  |  | ENST00000515287 |  |
|  |  | ENST00000511651 |  |
| TSPY1 | ENSG00000258992 | ENST00000451548 | (CG)3 |
| TSPY10 | ENSG00000236424 | ENST00000428845 |  |
| TSPY2 | ENSG00000168757 | ENST00000429039 |  |
|  |  | ENST00000320701 |  |
| TSPY3 | ENSG00000228927 | ENST00000457222 |  |
| TSPY4 | ENSG00000233803 | ENST00000426950 |  |
| TSPY8 | ENSG00000229549 | ENST00000287721 |  |
| TSPYL5 | ENSG00000180543 | ENST00000322128 | (GA)3 |
| TSSK3 | ENSG00000162526 | ENST00000574315 | (TC)3 |
| TTC27 | ENSG00000018699 | ENST00000448773 | (AG)3 |
| TTC28 | ENSG00000100154 | ENST00000397906 | (GCG)3 |
| TTC29 | ENSG00000137473 | ENST00000513335 | (TC)3 |
|  |  | ENST00000515315 |  |
| TTLL11 | ENSG00000175764 | ENST00000321582 | (CG)3 |
| TTN | ENSG00000155657 | ENST00000342992 | (GT)3 |
|  |  | ENST00000460472 |  |
|  |  | ENST00000589042 |  |
|  |  | ENST00000591111 |  |
|  |  | ENST00000360870 |  |
|  |  | ENST00000412264 |  |
| TUBB3 | ENSG00000258947 | ENST00000555810 | (TC)3 |
|  |  | ENST00000554444 |  |
|  |  | ENST00000556565 |  |
| TUBGCP2 | ENSG00000130640 | ENST00000368562 | (GC)3 |
| TUFM | ENSG00000178952 | ENST00000313511 | (TTC)3 |
| TWF1 | ENSG00000151239 | ENST00000552521 | (AT)3 |
|  |  | ENST00000546506 |  |
| TXNRD1 | ENSG00000198431 | ENST00000529751 |  |
| TYK2 | ENSG00000105397 | ENST00000524462 | (GAG)3 |
| TYW3 | ENSG00000162623 | ENST00000479111 | (TA)3 |
|  |  | ENST00000483990 |  |
| UBR4 | ENSG00000127481 | ENST00000375225 | (AT)3 |
|  |  | ENST00000375218 | (CAC)3 |
| UBXN11 | ENSG00000158062 | ENST00000450041 | (TCC)3 |
| UGT1A6 | ENSG00000167165 | ENST00000373424 | (AG)3 |
|  |  | ENST00000406651 |  |
| UIMC1 | ENSG00000087206 | ENST00000510698 | (AT)3 |
| ULBP2 | ENSG00000131015 | ENST00000367351 | (TC)3 |
| ULK1 | ENSG00000177169 | ENST00000321867 | (GCCC)3 |
| UNC13C | ENSG00000137766 | ENST00000539562 | (CA)3 |
| UNC5C | ENSG00000182168 | ENST00000453304 | (TC)3 |
|  |  | ENST00000513796 |  |
|  |  | ENST00000506749 |  |
|  |  | ENST00000504962 |  |
| UNC5D | ENSG00000156687 | ENST00000404895 | (CT)3 |
| UPP2 | ENSG00000007001 | ENST00000005756 | (TC)3 |
| UQCR11 | ENSG00000127540 | ENST00000591899 | (GC)3 |
| USO1 | ENSG00000138768 | ENST00000514213 | (GT)3 |
|  |  | ENST00000264904 |  |
| USP34 | ENSG00000115464 | ENST00000398571 | (TC)3 |
| USP35 | ENSG00000118369 | ENST00000528910 | (GAG)3 |
| USP49 | ENSG00000164663 | ENST00000394253 | (CTTTTT)3 |
|  |  | ENST00000373010 |  |
|  |  | ENST00000373006 |  |
|  |  | ENST00000423567 |  |
| USP7 | ENSG00000187555 | ENST00000566273 | (GT)3 |
| USP9Y | ENSG00000114374 | ENST00000338981 | (AT)3 |
| UTP15 | ENSG00000164338 | ENST00000543251 | (AC)3 |
| UTP3 | ENSG00000132467 | ENST00000254803 | (GC)3 |
| VAMP2 | ENSG00000220205 | ENST00000404970 | (TG)3 |
| VARS2 | ENSG00000137411 | ENST00000625423 | (TC)3 |
| VASH2 | ENSG00000143494 | ENST00000366966 | (GAG)3 |
|  |  | ENST00000366968 |  |
|  |  | ENST00000490792 |  |
| VCX | ENSG00000182583 | ENST00000381059 | (TA)3 |
|  |  | ENST00000620630 |  |
| VCX2 | ENSG00000177504 | ENST00000317103 |  |
| VCX3A | ENSG00000169059 | ENST00000381089 |  |
|  |  | ENST00000612369 |  |
| VCX3B | ENSG00000205642 | ENST00000381032 |  |
|  |  | ENST00000453306 |  |
|  |  | ENST00000444481 |  |
| VKORC1 | ENSG00000167397 | ENST00000498155 | (GA)3 |
|  |  |  |  |
| VN1R1 | ENSG00000178201 | ENST00000321039 | (AC)3 |
| VPS37B | ENSG00000139722 | ENST00000371248 | (GA)3 |
| VWA2 | ENSG00000165816 | ENST00000392982 | (CTTT)3 |
| VWCE | ENSG00000167992 | ENST00000535710 | (CT)3 |
| WBP2 | ENSG00000132471 | ENST00000589642 | (TC)3 |
|  |  | ENST00000587374 |  |
| WDR48 | ENSG00000114742 | ENST00000441361 | (AT)3 |
|  |  |  | (TC)3 |
|  |  |  | (AT)3 |
| WDR60 | ENSG00000126870 | ENST00000397143 | (AG)3 |
| WDR78 | ENSG00000152763 | ENST00000371026 | (CG)3 |
|  |  | ENST00000488333 | (TA)3 |
|  |  |  | (CA)3 |
| WDR81 | ENSG00000167716 | ENST00000419248 | (GTGGG)3 |
| WDR87 | ENSG00000171804 | ENST00000447313 | (TTG)3 |
|  |  | ENST00000303868 |  |
| WDR88 | ENSG00000166359 | ENST00000592765 | (GCGGGC)3 |
|  |  | ENST00000361680 |  |
| WDR92 | ENSG00000243667 | ENST00000406245 | (AT)3 |
| WFDC2 | ENSG00000101443 | ENST00000342873 | (AGG)3 |
| WRB | ENSG00000182093 | ENST00000398753 | (CA)3 |
|  |  | ENST00000442773 |  |
|  |  | ENST00000380713 |  |
|  |  | ENST00000380708 |  |
| WSCD1 | ENSG00000179314 | ENST00000573634 | (CAC)3 |
| XAGE1A | ENSG00000204379 | ENST00000375602 | (CA)3 |
|  |  | ENST00000375600 |  |
| XAGE1B | ENSG00000204382 | ENST00000375616 |  |
|  |  | ENST00000375613 |  |
|  |  | ENST00000518075 |  |
| XBP1 | ENSG00000100219 | ENST00000405219 | (CT)3 |
| XPNPEP1 | ENSG00000108039 | ENST00000369683 | (CTT)3 |
| XPO6 | ENSG00000169180 | ENST00000565698 | (CT)3 |
|  |  | ENST00000570033 |  |
|  |  | ENST00000566073 |  |
| XPO7 | ENSG00000130227 | ENST00000517551 | (AT)3 |
| XRCC1 | ENSG00000073050 | ENST00000598165 | (CT)3 |
| YIF1A | ENSG00000174851 | ENST00000496746 | (TCC)3 |
|  |  | ENST00000471387 | (TC)3 |
| YIPF4 | ENSG00000119820 | ENST00000238831 | (CGC)3 |
| YTHDC1 | ENSG00000083896 | ENST00000505251 | (TA)3 |
| YY1AP1 | ENSG00000163374 | ENST00000368339 | (GGT)3 |
|  |  |  | (GC)3 |
|  |  | ENST00000368340 | (GGT)3 |
|  |  |  | (GC)3 |
|  |  | ENST00000405763 |  |
| ZBBX | ENSG00000169064 | ENST00000392767 | (TA)3 |
|  |  | ENST00000392764 |  |
|  |  | ENST00000485651 |  |
| ZBTB34 | ENSG00000177125 | ENST00000319119 | (TTG)3 |
| ZBTB42 | ENSG00000179627 | ENST00000555360 | (GC)3 |
|  |  |  |  |
|  |  |  | (CGG)3 |
| ZBTB6 | ENSG00000186130 | ENST00000373659 | (TCT)3 |
| ZCCHC17 | ENSG00000121766 | ENST00000615916 | (GC)3 |
| ZDHHC13 | ENSG00000177054 | ENST00000399351 | (TC)3 |
| ZDHHC17 | ENSG00000186908 | ENST00000550876 | (AG)3 |
| ZFP36L1 | ENSG00000185650 | ENST00000557086 | (ACC)3 |
| ZFP41 | ENSG00000181638 | ENST00000520584 | (AG)3 |
|  |  | ENST00000330701 |  |
| ZFYVE1 | ENSG00000165861 | ENST00000394207 | (CT)3 |
|  |  | ENST00000555072 |  |
| ZFYVE28 | ENSG00000159733 | ENST00000508471 | (AC)3 |
| ZMYND10 | ENSG00000004838 | ENST00000442887 | (CA)3 |
| ZNF100 | ENSG00000197020 | ENST00000305570 | (GT)3 |
|  |  | ENST00000594401 |  |
| ZNF140 | ENSG00000196387 | ENST00000429434 | (TA)3 |
| ZNF160 | ENSG00000170949 | ENST00000601421 | (TC)3 |
|  |  | ENST00000601982 |  |
| ZNF180 | ENSG00000167384 | ENST00000592529 |  |
|  |  | ENST00000586637 |  |
|  |  | ENST00000591064 |  |
| ZNF19 | ENSG00000157429 | ENST00000288177 |  |
|  |  | ENST00000564230 |  |
|  |  | ENST00000568815 |  |
|  |  | ENST00000564225 |  |
|  |  | ENST00000566202 |  |
| ZNF208 | ENSG00000160321 | ENST00000597040 | (TG)3 |
|  |  |  | (TC)3 |
| ZNF226 | ENSG00000167380 | ENST00000585678 | (GGA)3 |
| ZNF254 | ENSG00000213096 | ENST00000613065 | (TG)3 |
|  |  | ENST00000616028 | (ATTT)3 |
| ZNF257 | ENSG00000197134 | ENST00000597927 | (TA)3 |
| ZNF28 | ENSG00000198538 | ENST00000339844 | (TC)3 |
| ZNF283 | ENSG00000167637 | ENST00000618787 | (TG)3 |
|  |  | ENST00000588797 |  |
|  |  | ENST00000593268 |  |
| ZNF296 | ENSG00000170684 | ENST00000303809 | (TGCC)3 |
|  |  | ENST00000622376 | (GCCT)3 |
| ZNF324 | ENSG00000083812 | ENST00000535298 | (TTC)3 |
|  |  | ENST00000536459 |  |
|  |  | ENST00000196482 |  |
| ZNF345 | ENSG00000251247 | ENST00000589046 | (TC)3 |
|  |  | ENST00000586933 |  |
|  |  | ENST00000614069 |  |
|  |  | ENST00000532141 |  |
|  |  | ENST00000420450 |  |
|  |  | ENST00000526123 |  |
|  |  | ENST00000529555 |  |
|  |  | ENST00000331800 |  |
|  |  | ENST00000612719 |  |
|  |  | ENST00000586646 |  |
|  |  | ENST00000585396 |  |
| ZNF346 | ENSG00000113761 | ENST00000506693 | (CT)3 |
|  |  | ENST00000358149 |  |
|  |  | ENST00000512315 |  |
|  |  | ENST00000503425 |  |
| ZNF347 | ENSG00000197937 | ENST00000595967 | (TC)3 |
| ZNF407 | ENSG00000215421 | ENST00000582337 | (GT)3 |
| ZNF410 | ENSG00000119725 | ENST00000556179 | (GA)3 |
| ZNF415 | ENSG00000170954 | ENST00000601493 | (AT)3 |
| ZNF418 | ENSG00000196724 | ENST00000599852 | (GT)3 |
| ZNF420 | ENSG00000197050 | ENST00000590332 | (TC)3 |
| ZNF433 | ENSG00000197647 | ENST00000455504 | (AG)3 |
|  |  | ENST00000478765 |  |
| ZNF439 | ENSG00000171291 | ENST00000455282 | (AGA)3 |
|  |  |  | (GT)3 |
| ZNF440 | ENSG00000171295 | ENST00000457526 |  |
| ZNF442 | ENSG00000198342 | ENST00000242804 | (AC)3 |
| ZNF45 | ENSG00000124459 | ENST00000588140 | (GT)3 |
|  |  |  | (GAG)3 |
| ZNF454 | ENSG00000178187 | ENST00000320129 | (TC)3 |
|  |  | ENST00000519564 |  |
| ZNF468 | ENSG00000204604 | ENST00000601847 |  |
| ZNF480 | ENSG00000198464 | ENST00000595962 | (GT)3 |
|  |  | ENST00000598016 |  |
|  |  | ENST00000334564 |  |
|  |  | ENST00000490272 |  |
|  |  | ENST00000335090 | (TC)3 |
| ZNF488 | ENSG00000265763 | ENST00000585316 | (GT)3 |
| ZNF492 | ENSG00000229676 | ENST00000456783 | (TG)3 |
|  |  |  | (TC)3 |
| ZNF493 | ENSG00000196268 | ENST00000596302 | (CT)3 |
|  |  | ENST00000392288 |  |
|  |  | ENST00000594390 |  |
|  |  | ENST00000355504 | (GA)3 |
| ZNF497 | ENSG00000174586 | ENST00000595763 | (TG)3 |
| ZNF525 | ENSG00000203326 | ENST00000467003 | (TC)3 |
| ZNF528 | ENSG00000167555 | ENST00000391788 | (TG)3 |
|  |  | ENST00000493272 |  |
| ZNF546 | ENSG00000187187 | ENST00000599504 | (AC)3 |
|  |  | ENST00000601138 |  |
| ZNF552 | ENSG00000178935 | ENST00000391701 | (GTG)3 |
| ZNF565 | ENSG00000196357 | ENST00000355114 | (CCT)3 |
| ZNF585B | ENSG00000245680 | ENST00000531805 | (AG)3 |
|  |  | ENST00000591492 | (TA)3 |
| ZNF589 | ENSG00000164048 | ENST00000354698 | (CA)3 |
|  |  |  | (CGTG)3 |
|  |  |  | (GC)3 |
|  |  | ENST00000427617 | (CA)3 |
|  |  |  | (CGTG)3 |
|  |  |  | (GC)3 |
|  |  | ENST00000412564 | (CA)3 |
|  |  |  | (CGTG)3 |
|  |  |  | (GC)3 |
|  |  | ENST00000440261 | (CA)3 |
|  |  |  | (CGTG)3 |
|  |  |  | (GC)3 |
| ZNF595 | ENSG00000272602 | ENST00000608255 | (GA)3 |
| ZNF598 | ENSG00000167962 | ENST00000563630 | (GGC)3 |
|  |  | ENST00000562103 |  |
| ZNF600 | ENSG00000189190 | ENST00000338230 | (AC)3 |
| ZNF627 | ENSG00000198551 | ENST00000585493 | (CA)3 |
|  |  | ENST00000587939 |  |
| ZNF638 | ENSG00000075292 | ENST00000466975 | (AG)3 |
|  |  | ENST00000466330 | (AT)3 |
| ZNF648 | ENSG00000179930 | ENST00000339948 | (CTC)3 |
| ZNF676 | ENSG00000196109 | ENST00000397121 | (AT)3 |
|  |  |  | (TC)3 |
| ZNF701 | ENSG00000167562 | ENST00000611267 | (GA)3 |
|  |  | ENST00000301093 | (CAC)3 |
|  |  | ENST00000593941 |  |
|  |  | ENST00000540331 |  |
| ZNF714 | ENSG00000160352 | ENST00000616183 | (TA)3 |
|  |  |  | (GA)3 |
|  |  | ENST00000596053 | (GT)3 |
|  |  |  | (TC)3 |
|  |  | ENST00000618422 | (GT)3 |
|  |  |  | (TC)3 |
|  |  | ENST00000456283 | (GT)3 |
|  |  |  | (TC)3 |
|  |  | ENST00000618008 | (GT)3 |
|  |  |  | (TC)3 |
|  |  | ENST00000425625 | (GT)3 |
|  |  |  | (TC)3 |
|  |  | ENST00000620627 | (GT)3 |
|  |  |  | (TC)3 |
| ZNF717 | ENSG00000227124 | ENST00000477374 |  |
|  |  | ENST00000468296 |  |
| ZNF730 | ENSG00000183850 | ENST00000593635 | (TG)3 |
|  |  |  | (TC)3 |
| ZNF749 | ENSG00000186230 | ENST00000415248 | (GT)3 |
| ZNF765 | ENSG00000196417 | ENST00000505866 |  |
| ZNF766 | ENSG00000196214 | ENST00000439461 | (GC)3 |
|  |  | ENST00000595149 | (TC)3 |
|  |  | ENST00000595000 |  |
| ZNF768 | ENSG00000169957 | ENST00000562803 |  |
| ZNF778 | ENSG00000170100 | ENST00000306502 | (TG)3 |
|  |  |  | (AG)3 |
| ZNF780A | ENSG00000197782 | ENST00000450241 | (TC)3 |
| ZNF781 | ENSG00000196381 | ENST00000358582 | (TG)3 |
|  |  | ENST00000590008 |  |
| ZNF804A | ENSG00000170396 | ENST00000302277 | (GC)3 |
|  |  |  | (GGC)3 |
| ZNF808 | ENSG00000198482 | ENST00000486474 | (AC)3 |
| ZNF837 | ENSG00000152475 | ENST00000427624 | (TC)3 |
| ZNF841 | ENSG00000197608 | ENST00000426391 |  |
|  |  |  | (TG)3 |
| ZNF844 | ENSG00000223547 | ENST00000550826 | (CA)3 |
| ZNF846 | ENSG00000196605 | ENST00000588267 | (GA)3 |
|  |  | ENST00000592859 |  |
| ZNF85 | ENSG00000105750 | ENST00000599064 | (AT)3 |
|  |  | ENST00000598862 |  |
| ZNF91 | ENSG00000167232 | ENST00000599743 | (TG)3 |
|  |  | ENST00000300619 | (TC)3 |
|  |  |  | (TG)3 |
|  |  | ENST00000397082 |  |
| ZNF98 | ENSG00000197360 | ENST00000357774 | (TC)3 |
|  |  | ENST00000593657 | (TG)3 |
|  |  |  | (TC)3 |
|  |  | ENST00000601553 |  |
| ZSCAN18 | ENSG00000121413 | ENST00000421612 | (TTG)3 |
| ZSCAN30 | ENSG00000186814 | ENST00000610712 | (AATA)3 |
| ZXDA | ENSG00000198205 | ENST00000358697 | (CCT)3 |
| ZXDB | ENSG00000198455 | ENST00000374888 |  |
