# Supplementary material for: Link between short tandem repeats and translation initiation site selection
Source: Hum Genomics. 2018 Oct 29;12:47. doi: 10.1186/s40246-018-0181-3 (PMC6206671; doi:10.1186/s40246-018-0181-3)
Supplement: Supplementary file 5 — List of all human protein-coding genes which contain human-specific STRs in their TIS-flanking cDNA sequence. (DOCX 393 kb) [file 40246_2018_181_MOESM5_ESM.docx]

| Gene Symbol | Ensembl ID | Transcript ID | STR |
| --- | --- | --- | --- |
| KCTD19 | ENSG00000168676 | ENST00000566295 | (A)20 |
| ATP8B1 | ENSG00000081923 | ENST00000585322 | (A)17 |
| C1QTNF1 | ENSG00000173918 | ENST00000583904 |  |
| SEC11A | ENSG00000140612 | ENST00000558196 |  |
| SHQ1 | ENSG00000144736 | ENST00000463369 |  |
| SPRY1 | ENSG00000164056 | ENST00000505319 |  |
|  |  | ENST00000610581 |  |
| DDX20 | ENSG00000064703 | ENST00000475700 | (A)16 |
| NAB1 | ENSG00000138386 | ENST00000409641 | (T)16 |
| SGIP1 | ENSG00000118473 | ENST00000435165 | (A)16 |
| SOX6 | ENSG00000110693 | ENST00000528252 | (A)14 |
| DPP6 | ENSG00000130226 | ENST00000406326 | (T)13 |
|  |  | ENST00000377770 |  |
| MLF1 | ENSG00000178053 | ENST00000482628 | (G)13 |
| SHC4 | ENSG00000185634 | ENST00000558220 | (T)13 |
| ITGB1BP2 | ENSG00000147166 | ENST00000538820 | (T)12 |
| NELL2 | ENSG00000184613 | ENST00000548531 |  |
| GIPC1 | ENSG00000123159 | ENST00000527578 | (GCG)11 |
|  |  | ENST00000556376 |  |
|  |  | ENST00000331351 |  |
| HBS1L | ENSG00000112339 | ENST00000642043 | (T)11 |
| HOPX | ENSG00000171476 | ENST00000520638 | (A)11 |
| OR7D2 | ENSG00000188000 | ENST00000468958 | (T)11 |
| RNF145 | ENSG00000145860 | ENST00000359920 |  |
| TXNL4A | ENSG00000141759 | ENST00000320767 |  |
| ABCF1 | ENSG00000204574 | ENST00000448127 | (A)10 |
| ARHGEF18 | ENSG00000104880 | ENST00000466499 | (T)10 |
| ARL14 | ENSG00000179674 | ENST00000437025 | (A)10 |
| ASNS | ENSG00000070669 | ENST00000239026 | (T)10 |
| EIF2S1 | ENSG00000134001 | ENST00000371332 |  |
| GABRA1 | ENSG00000022355 | ENST00000526028 | (G)10 |
| HELLS | ENSG00000119969 | ENST00000642123 | (A)10 |
|  |  | ENST00000641113 |  |
| HYLS1 | ENSG00000198331 | ENST00000361132 | (T)10 |
| OR7A17 | ENSG00000185385 | ENST00000439214 | (AATA)10 |
|  |  | ENST00000564644 |  |
| RASGEF1C | ENSG00000146090 | ENST00000563793 | (GCG)10 |
| SNAP29 | ENSG00000099940 | ENST00000260010 | (T)10 |
| SNUPN | ENSG00000169371 | ENST00000544599 | (A)10 |
|  |  | ENST00000641119 |  |
| TLR2 | ENSG00000137462 | ENST00000641787 | (GT)10 |
| TMBIM4 | ENSG00000155957 | ENST00000354504 | (T)10 |
| TNNT3 | ENSG00000130595 | ENST00000374694 | (CT)10 |
|  |  | ENST00000425380 |  |
| VPS54 | ENSG00000143952 | ENST00000551524 | (A)10 |
| FZD8 | ENSG00000177283 | ENST00000572156 | (C)10 |
| HYLS1 | ENSG00000198331 | ENST00000571570 | (T)10 |
| NAP1L1 | ENSG00000187109 | ENST00000644939 |  |
| AKAP1 | ENSG00000121057 | ENST00000406775 | (A)9 |
| ANAPC11 | ENSG00000141552 | ENST00000342771 |  |
| AUTS2 | ENSG00000158321 | ENST00000526815 | (T)9 |
|  |  | ENST00000377392 |  |
|  |  | ENST00000579490 |  |
| BBS1 | ENSG00000174483 | ENST00000411537 |  |
| CBWD5 | ENSG00000147996 | ENST00000371205 |  |
| DNAI2 | ENSG00000171595 | ENST00000335071 |  |
| EIF2AK2 | ENSG00000055332 | ENST00000396836 | (CGG)9 |
| ENTPD1 | ENSG00000138185 | ENST00000543446 | (G)9 |
| FOXE3 | ENSG00000186790 | ENST00000393930 |  |
| IL31RA | ENSG00000164509 | ENST00000434945 | (A)9 |
| IPO8 | ENSG00000133704 | ENST00000636178 |  |
| JUP | ENSG00000173801 | ENST00000549808 | (C)9 |
| KDELC2 | ENSG00000178202 | ENST00000565250 | (T)9 |
| MAGI2 | ENSG00000187391 | ENST00000290208 |  |
| METAP2 | ENSG00000111142 |  |  |
| MLST8 | ENSG00000167965 | ENST00000554903 | (G)9 |
| MRPL10 | ENSG00000159111 | ENST00000642123  ENST00000641113 | (CT)9 |
|  |  |  | (T)9 |
| NFATC4 | ENSG00000100968 | ENST00000438716 | (G)9 |
| OR7A17 | ENSG00000185385 | ENST00000507554 | (A)9 |
|  |  | ENST00000547125 |  |
| OSBP2 | ENSG00000184792 | ENST00000448497 |  |
| PGM3 | ENSG00000013375 | ENST00000625099 | (C)9 |
| PRKAG1 | ENSG00000181929 | ENST00000546697 | (A)9 |
| RFC4 | ENSG00000163918 | ENST00000503245 | (T)9 |
| SLC22A18AS | ENSG00000254827 | ENST00000417523 |  |
| SSH1 | ENSG00000084112 | ENST00000394511 |  |
| TAF9 | ENSG00000273841 | ENST00000594401 | (G)9 |
| TRRAP | ENSG00000196367 | ENST00000601711 | (C)9 |
| UGT8 | ENSG00000174607 | ENST00000593612 | (T)9 |
| ZNF100 | ENSG00000197020 | ENST00000358697 |  |
| ZNF766 | ENSG00000196214 | ENST00000449239 | (A)9 |
|  |  | ENST00000557515 |  |
| ZXDA | ENSG00000198205 |  | (T)9 |
| SEPT2 | ENSG00000168385 |  |  |
| ACIN1 | ENSG00000100813 | ENST00000397341 | (T)8 |
|  |  |  |  |
|  |  |  |  |
|  |  | ENST00000561795  ENST00000547400  ENST00000550683 |  |
|  |  |  |  |
|  |  |  |  |
| ACSM3 | ENSG00000005187 | ENST00000419526 | (A)8 |
| ACVRL1 | ENSG00000139567 | ENST00000556843 |  |
|  |  | ENST00000381333 |  |
|  |  | ENST00000306011 |  |
| AP1G2 | ENSG00000213983 | ENST00000526092 |  |
| ASMTL | ENSG00000169093 | ENST00000588168 | (T)8 |
| ATOH1 | ENSG00000172238 | ENST00000525908 |  |
| ATP8B3 | ENSG00000130270 | ENST00000438029 | (C)8 |
| BCAS3 | ENSG00000141376 | ENST00000473555 | (A)8 |
| C11orf80 | ENSG00000173715 | ENST00000619887 | (CGG)8 |
| C7orf25 | ENSG00000136197 | ENST00000398788 | (T)8 |
| CDC6 | ENSG00000094804 | ENST00000614565 |  |
| CDH23 | ENSG00000107736 | ENST00000369207 | (GAG)8 |
|  |  | ENST00000489298 |  |
| CDH4 | ENSG00000179242 | ENST00000528737 | (CGG)8 |
| ENO4 | ENSG00000188316 | ENST00000533703 | (A)8 |
| FAM104B | ENSG00000182518 | ENST00000427372 |  |
| FAM111A | ENSG00000166801 | ENST00000478136 | (T)8 |
|  |  | ENST00000370674 |  |
| FAM133B | ENSG00000234545 | ENST00000509100 | (C)8 |
| FAM19A1 | ENSG00000183662 | ENST00000437025 | (A)8 |
| FHL1 | ENSG00000022267 | ENST00000591349 |  |
| G3BP2 | ENSG00000138757 | ENST00000479011 | (G)8 |
| GABRA1 | ENSG00000022355 | ENST00000380763 | (AG)8 |
| GIPC1 | ENSG00000123159 | ENST00000397596 | (CGG)8 |
| HBP1 | ENSG00000105856 | ENST00000619416 | (A)8 |
| HCCS | ENSG00000004961 | ENST00000418176 | (GGC)8 |
| HRAS | ENSG00000174775 | ENST00000423542 | (GCG)8 |
| KIAA0586 | ENSG00000100578 | ENST00000366272 | (A)8 |
| KRBOX1 | ENSG00000240747 | ENST00000436410 | (T)8 |
| LGR6 | ENSG00000133067 | ENST00000223167 |  |
| MLXIP | ENSG00000175727 | ENST00000338909 |  |
| MX2 | ENSG00000183486 | ENST00000526637 | (A)8 |
| MYL10 | ENSG00000106436 | ENST00000394480 |  |
| MYOM3 | ENSG00000142661 | ENST00000355254 |  |
| NAALAD2 | ENSG00000077616 | ENST00000360948 |  |
| NTRK3 | ENSG00000140538 | ENST00000357724 | (GGC)8 |
|  |  | ENST00000540489 |  |
|  |  | ENST00000641380 |  |
|  |  | ENST00000641576 |  |
|  |  | ENST00000641288 |  |
| OR5H14 | ENSG00000236032 | ENST00000509469 | (T)8 |
| OR6C75 | ENSG00000187857 | ENST00000513861 | (A)8 |
| OR7D2 | ENSG00000188000 | ENST00000502374 | (T)8 |
| PACRGL | ENSG00000163138 | ENST00000507634 | (C)8 |
|  |  | ENST00000615790 |  |
|  |  | ENST00000612941 |  |
|  |  | ENST00000439674 |  |
| PEG10 | ENSG00000242265 | ENST00000508595 | (A)8 |
|  |  | ENST00000513323 |  |
| PHF19 | ENSG00000119403 | ENST00000577053 |  |
| RPL9 | ENSG00000163682 | ENST00000533632 |  |
| SEC31A | ENSG00000138674 | ENST00000216185 |  |
| SERPINF1 | ENSG00000132386 | ENST00000396703 | (ATAA)8 |
| TRAPPC4 | ENSG00000196655 | ENST00000338981 | (GGA)8 |
| TXN2 | ENSG00000100348 | ENST00000420673 | (C)8 |
| UBE2E2 | ENSG00000182247 | ENST00000570033 |  |
| USP9Y | ENSG00000114374 | ENST00000358582 | (A)8 |
| XPO1 | ENSG00000082898 | ENST00000590008 | (T)8 |
| XPO6 | ENSG00000169180 | ENST00000371602 |  |
| ZNF781 | ENSG00000196381 | ENST00000306011 | (A)8 |
|  |  | ENST00000400163 |  |
| ADNP | ENSG00000101126 | ENST00000448959 | (T)8 |
| ATOH1 | ENSG00000172238 | ENST00000409817 | (A)8 |
| COL4A2 | ENSG00000134871 | ENST00000586770 | (T)8 |
| CPVL | ENSG00000106066 | ENST00000489329 |  |
| CXCR4 | ENSG00000121966 | ENST00000641364 |  |
| EML2 | ENSG00000125746 | ENST00000540783 |  |
| MFN1 | ENSG00000171109 | ENST00000538974 |  |
| OR6C3 | ENSG00000205329 | ENST00000405158 | (T)4 |
| RAPGEF4 | ENSG00000091428 | ENST00000533683 | (A)8 |
|  |  | ENST00000383045 |  |
| SAA1 | ENSG00000173432 | ENST00000438754 |  |
| SAMD1 | ENSG00000141858 | ENST00000520734 | (G)8 |
| SEPT5 | ENSG00000184702 | ENST00000520052 | (G)7 |
|  |  | ENST00000454629 |  |
| ANGPT1 | ENSG00000154188 | ENST00000599990 | (A)7 |
|  |  | ENST00000510085 |  |
| AOX1 | ENSG00000138356 | ENST00000447005 |  |
| AP2S1 | ENSG00000042753 | ENST00000284694 | (G)7 |
| ATG10 | ENSG00000152348 | ENST00000432642 | (T)7 |
| BBIP1 | ENSG00000214413 | ENST00000422619 |  |
| C10orf90 | ENSG00000154493 | ENST00000637424 |  |
|  |  | ENST00000538450 |  |
| C3orf18 | ENSG00000088543 | ENST00000397204 | (A)7 |
| CACNA1D | ENSG00000157388 | ENST00000446140 |  |
| CACNA2D4 | ENSG00000151062 | ENST00000596938 | (T)7 |
| CAPN3 | ENSG00000092529 | ENST00000367036 | (A)7 |
| CCDC51 | ENSG00000164051 | ENST00000513274 | (G)7 |
| CCDC9 | ENSG00000105321 | ENST00000512242 | (T)7 |
| CD34 | ENSG00000174059 | ENST00000514508 |  |
| COPB2 | ENSG00000184432 | ENST00000409603 | (A)7 |
|  |  | ENST00000438788 |  |
|  |  | ENST00000498635 |  |
| CREB5 | ENSG00000146592 | ENST00000611925 | (T)7 |
| CRYZL1 | ENSG00000205758 | ENST00000269703 |  |
| CSAD | ENSG00000139631 | ENST00000620214 | (A)7 |
| CYFIP2 | ENSG00000055163 | ENST00000475733 | (T)7 |
| CYP4F22 | ENSG00000171954 | ENST00000407642 |  |
| CYTH2 | ENSG00000105443 | ENST00000552375 | (A)7 |
| DCAF8 | ENSG00000132716 | ENST00000614082 | (C)7 |
|  |  | ENST00000495027 |  |
| DDX54 | ENSG00000123064 | ENST00000351747 | (A)7 |
| DNAH10 | ENSG00000197653 | ENST00000389536 | (T)7 |
| DNAH12 | ENSG00000174844 | ENST00000311202 | (TC)7 |
|  |  | ENST00000559405 |  |
|  |  | ENST00000316292 |  |
|  |  | ENST00000466499 |  |
| DTWD1 | ENSG00000104047 | ENST00000580989 | (T)7 |
| EEF1A1 | ENSG00000156508 | ENST00000411471 |  |
| EIF2S1 | ENSG00000134001 | ENST00000580032 |  |
| EPB41L3 | ENSG00000082397 | ENST00000504595 |  |
| EPHB6 | ENSG00000106123 | ENST00000581349 | (C)7 |
| FAM104A | ENSG00000133193 | ENST00000339140 |  |
| FBXL7 | ENSG00000183580 | ENST00000524754 | (G)7 |
| FLII | ENSG00000177731 | ENST00000286955 |  |
| FOXR2 | ENSG00000189299 | ENST00000480918 | (A)7 |
| FUT6 | ENSG00000156413 | ENST00000484042 | (C)7 |
|  |  | ENST00000358713 |  |
| FXR1 | ENSG00000114416 | ENST00000624495 | (T)7 |
|  |  | ENST00000441491 |  |
| GATAD2A | ENSG00000167491 | ENST00000603546 | (A)7 |
| GDF9 | ENSG00000164404 | ENST00000429670 | (T)7 |
| GET4 | ENSG00000239857 | ENST00000502664 |  |
| GNAS | ENSG00000087460 | ENST00000358615 | (G)7 |
| GYPB | ENSG00000250361 | ENST00000529882 | (T)7 |
|  |  | ENST00000523628 |  |
| GYPE | ENSG00000197465 | ENST00000579861 |  |
| HBS1L | ENSG00000112339 | ENST00000612898 | (G)7 |
| HDAC2 | ENSG00000196591 | ENST00000606613 | (T)7 |
| HELZ | ENSG00000198265 | ENST00000440865 | (A)7 |
| HIST1H2BN | ENSG00000233822 | ENST00000352456 | (T)7 |
|  |  | ENST00000375003 |  |
| HNRNPU | ENSG00000153187 | ENST00000331351 |  |
| HNRNPUL1 | ENSG00000105323 | ENST00000507221 | (GA)7 |
| HP1BP3 | ENSG00000127483 | ENST00000531939 | (A)7 |
| HS3ST4 | ENSG00000182601 | ENST00000358724 | (GCC)7 |
| IFT122 | ENSG00000163913 | ENST00000579662 | (T)7 |
| IFT46 | ENSG00000118096 | ENST00000541852 |  |
| IPO8 | ENSG00000133704 | ENST00000543957 | (A)7 |
| ITGB4 | ENSG00000132470 | ENST00000409704 | (C)7 |
| ITM2C | ENSG00000135916 | ENST00000418408 | (G)7 |
|  |  | ENST00000382148 |  |
|  |  | ENST00000457623 |  |
|  |  | ENST00000371876 |  |
| KCNIP4 | ENSG00000185774 | ENST00000305641 | (ATT)7 |
| KIAA1324 | ENSG00000116299 | ENST00000396076 | (T)7 |
| KLHL13 | ENSG00000003096 | ENST00000563189 | (A)7 |
| LCMT2 | ENSG00000168806 | ENST00000435835 | (T)7 |
| LDHB | ENSG00000111716 | ENST00000373313 | (G)7 |
| LRRC36 | ENSG00000159708 | ENST00000329006 | (A)7 |
|  |  | ENST00000598608 |  |
| MAFB | ENSG00000204103 | ENST00000452930 | (C)7 |
| MANEAL | ENSG00000185090 | ENST00000582920 | (A)7 |
| MED26 | ENSG00000105085 | ENST00000513169 | (G)7 |
| MFF | ENSG00000168958 | ENST00000442588 | (C)7 |
| MSL1 | ENSG00000188895 | ENST00000391974 | (A)7 |
| MXD3 | ENSG00000213347 | ENST00000587891 | (C)7 |
| NEK7 | ENSG00000151414 | ENST00000592654 | (A)7 |
|  |  | ENST00000641067 |  |
| NLRP4 | ENSG00000160505 | ENST00000641398 |  |
| NMT1 | ENSG00000136448 | ENST00000641124 |  |
| OR10R2 | ENSG00000198965 | ENST00000641169 |  |
| OR1I1 | ENSG00000094661 | ENST00000641114 | (G)7 |
| OR2AG2 | ENSG00000188124 | ENST00000377173 | (A)7 |
|  |  | ENST00000408955 |  |
| OR2AP1 | ENSG00000179615 | ENST00000641945 | (T)7 |
| OR2B3 | ENSG00000204703 | ENST00000427338 |  |
| OR2F2 | ENSG00000221910 | ENST00000641576 |  |
| OR2T8 | ENSG00000177462 | ENST00000543457 | (A)7 |
| OR5K2 | ENSG00000231861 | ENST00000541518 | (T)7 |
| OR6C75 | ENSG00000187857 | ENST00000374519 | (A)7 |
| OXGR1 | ENSG00000165621 | ENST00000495692 |  |
|  |  | ENST00000396193 |  |
| P2RY4 | ENSG00000186912 | ENST00000573076 | (T)7 |
| PANK2 | ENSG00000125779 | ENST00000381551 |  |
| PDE1C | ENSG00000154678 | ENST00000506783 | (G)7 |
| PDE6G | ENSG00000185527 | ENST00000355496 | (C)7 |
| PDGFB | ENSG00000100311 | ENST00000263265 | (AG)7 |
| PHF3 | ENSG00000118482 | ENST00000481701 | (A)7 |
| PLEKHA4 | ENSG00000105559 | ENST00000624204 | (C)7 |
|  |  | ENST00000432292 |  |
| PLSCR4 | ENSG00000114698 | ENST00000441246 | (T)7 |
| PMS1 | ENSG00000064933 | ENST00000433463 | (A)7 |
|  |  | ENST00000493290 |  |
| POLR2B | ENSG00000047315 | ENST00000548950 | (C)7 |
|  |  | ENST00000548605 |  |
| PON2 | ENSG00000105854 | ENST00000357068 | (T)7 |
| PRKAG1 | ENSG00000181929 | ENST00000547221 |  |
|  |  | ENST00000534093 |  |
| PRODH | ENSG00000100033 | ENST00000382933 | (GCGGG)7 |
| PSORS1C1 | ENSG00000204540 | ENST00000557351 | (C)7 |
| ROM1 | ENSG00000149489 | ENST00000359203 | (G)7 |
| RPGRIP1 | ENSG00000092200 | ENST00000430715 | (A)7 |
|  |  | ENST00000536859 |  |
| RRAGD | ENSG00000025039 | ENST00000556091 | (GGA)7 |
| RTN2 | ENSG00000125744 | ENST00000513453 | (C)7 |
| SERINC2 | ENSG00000168528 | ENST00000564778 | (A)7 |
| SERPINA1 | ENSG00000197249 | ENST00000631130 | (C)7 |
| SIL1 | ENSG00000120725 | ENST00000461354 | (G)7 |
| SIN3A | ENSG00000169375 | ENST00000466255 | (C)7 |
| SLC4A1 | ENSG00000004939 | ENST00000455277 | (T)7 |
| SLMAP | ENSG00000163681 | ENST00000543631 |  |
|  |  | ENST00000531917 |  |
| SMYD3 | ENSG00000185420 | ENST00000601879 |  |
| SPARCL1 | ENSG00000152583 | ENST00000636456 | (A)7 |
| SSR2 | ENSG00000163479 | ENST00000437822 | (C)7 |
| SUGP2 | ENSG00000064607 | ENST00000513596 | (T)7 |
| TAB2 | ENSG00000055208 | ENST00000470080 | (G)7 |
| TAF6 | ENSG00000106290 | ENST00000380636 | (A)7 |
| TBC1D19 | ENSG00000109680 | ENST00000532437 | (T)7 |
| TM4SF18 | ENSG00000163762 | ENST00000444177 | (GA)7 |
| TMSB4X | ENSG00000205542 | ENST00000415766 | (T)7 |
| TNKS1BP1 | ENSG00000149115 | ENST00000450533 |  |
| TREX1 | ENSG00000213689 | ENST00000438055 | (G)7 |
| TSNAXIP1 | ENSG00000102904 | ENST00000355666 |  |
| TTC3 | ENSG00000182670 | ENST00000399010 | (GGC)7 |
|  |  | ENST00000360271 |  |
|  |  | ENST00000507845 |  |
|  |  | ENST00000502404 |  |
| UAP1L1 | ENSG00000197355 | ENST00000508476 | (C)7 |
| UBE2D3 | ENSG00000109332 | ENST00000512704 | (A)7 |
|  |  | ENST00000593597 |  |
|  |  | ENST00000427112 |  |
| UGT2A1 | ENSG00000173610 | ENST00000404970 | (T)7 |
| USE1 | ENSG00000053501 | ENST00000370596 | (C)7 |
| USP40 | ENSG00000085982 | ENST00000342873 | (T)7 |
| VAMP2 | ENSG00000220205 | ENST00000471387 | (C)7 |
| WDR63 | ENSG00000162643 | ENST00000586748 | (CTG)7 |
| WFDC2 | ENSG00000101443 | ENST00000590329 | (G)7 |
| YIF1A | ENSG00000174851 | ENST00000588347 | (C)7 |
| YIPF2 | ENSG00000130733 | ENST00000575984 | (G)7 |
|  |  | ENST00000509562 |  |
|  |  | ENST00000251269 |  |
| ZC3H7A | ENSG00000122299 | ENST00000588797 | (T)7 |
| ZFYVE16 | ENSG00000039319 | ENST00000560079 | (A)7 |
| ZNF221 | ENSG00000159905 | ENST00000591164 |  |
| ZNF283 | ENSG00000167637 | ENST00000601711 |  |
| ZNF592 | ENSG00000166716 | ENST00000593612 | (G)7 |
| ZNF628 | ENSG00000197483 | ENST00000562803 | (C)7 |
| ZNF766 | ENSG00000196214 | ENST00000425191 | (A)7 |
|  |  | ENST00000569156 |  |
| ZNF768 | ENSG00000169957 | ENST00000527942 | (G)7 |
| ABAT | ENSG00000183044 | ENST00000398322 | (G)6 |
|  |  | ENST00000524990 |  |
| ACAT1 | ENSG00000075239 | ENST00000526597 | (T)6 |
| ACBD4 | ENSG00000181513 | ENST00000556433 | (G)6 |
| ACCS | ENSG00000110455 | ENST00000521741 |  |
| ACER3 | ENSG00000078124 | ENST00000430656 | (T)6 |
| ACTN1 | ENSG00000072110 |  | (G)6 |
| ADAM32 | ENSG00000197140 | ENST00000509482 | (T)6 |
| ADD2 | ENSG00000075340 | ENST00000369295  ENST00000412973 |  |
|  |  |  | (C)6 |
| ADRA2C | ENSG00000184160 | ENST00000478407 | (G)6 |
| ADRB1 | ENSG00000043591 | ENST00000380554 | (C)6 |
| AGR2 | ENSG00000106541 | ENST00000596634 | (A)6 |
| AK5 | ENSG00000154027 | ENST00000460201 | (G)6 |
| AKR1C3 | ENSG00000196139 | ENST00000340333 |  |
| AKT2 | ENSG00000105221 | ENST00000525870 |  |
| ALDOC | ENSG00000109107 | ENST00000527099 | (T)6 |
| ALG1L | ENSG00000189366 | ENST00000556298 |  |
| ALG8 | ENSG00000159063 |  | (C)6 |
|  |  | ENST00000562196 |  |
| ANGEL1 | ENSG00000013523 | ENST00000611388  ENST00000357336 | (A)6 |
|  |  |  | (C)6 |
| ANKRD20A1 | ENSG00000260691 | ENST00000615302 | (G)6 |
| ANKRD20A3 | ENSG00000276203 | ENST00000531349 |  |
| ANKRD20A4 | ENSG00000172014 | ENST00000592316 |  |
|  |  | ENST00000546731 |  |
| ANO1 | ENSG00000131620 | ENST00000447660 | (C)6 |
| APLP1 | ENSG00000105290 | ENST00000525234 |  |
| APPL2 | ENSG00000136044 | ENST00000378944 | (T)6 |
| AQP7 | ENSG00000165269 | ENST00000396582 | (G)6 |
| ARHGAP32 | ENSG00000134909 | ENST00000502271 | (CGG)6 |
| ARHGAP33 | ENSG00000004777 | ENST00000518908 | (C)6 |
| ARHGAP5 | ENSG00000100852 | ENST00000395619 | (G)6 |
| ARL15 | ENSG00000185305 | ENST00000545791 | (A)6 |
| ARMC1 | ENSG00000104442 | ENST00000540212 | (C)6 |
| ARSA | ENSG00000100299 | ENST00000402096 |  |
| ASB8 | ENSG00000177981 | ENST00000419606 |  |
|  |  | ENST00000430617 |  |
| ATG4B | ENSG00000168397 | ENST00000429899 | (G)6 |
|  |  | ENST00000376151 |  |
|  |  | ENST00000303892 |  |
|  |  | ENST00000585322 |  |
| ATP6V1G2 | ENSG00000213760 | ENST00000338821 |  |
|  |  | ENST00000644506 |  |
| ATP8B1 | ENSG00000081923 | ENST00000644359 | (A)6 |
| ATP9A | ENSG00000054793 | ENST00000392779 | (G)6 |
| AUTS2 | ENSG00000158321 | ENST00000498216 | (C)6 |
|  |  | ENST00000547141 |  |
| B3GALNT1 | ENSG00000169255 | ENST00000493798 | (A)6 |
|  |  | ENST00000571530 |  |
| BACH1 | ENSG00000156273 | ENST00000418647 | (T)6 |
| BAD | ENSG00000002330 | ENST00000588874 | (C)6 |
| BAIAP2 | ENSG00000175866 | ENST00000438077 | (T)6 |
| BCAR1 | ENSG00000050820 | ENST00000559501 | (A)6 |
| BCAS3 | ENSG00000141376 | ENST00000357607 | (T)6 |
| BCL6 | ENSG00000113916 | ENST00000518611 | (G)6 |
| BMP4 | ENSG00000125378 | ENST00000618469 | (A)6 |
| BMX | ENSG00000102010 | ENST00000545507 | (T)6 |
| BNIP3L | ENSG00000104765 | ENST00000573784 | (A)6 |
| BRCA1 | ENSG00000012048 | ENST00000576984 | (CAAAA)6 |
| BSG | ENSG00000172270 | ENST00000527417 | (G)6 |
|  |  | ENST00000541014 |  |
|  |  | ENST00000580558 |  |
| BTN3A2 | ENSG00000186470 | ENST00000581747 | (A)6 |
| C12orf4 | ENSG00000047621 | ENST00000583334 |  |
| C17orf75 | ENSG00000108666 | ENST00000379320 | (T)6 |
|  |  | ENST00000528368 |  |
|  |  | ENST00000402681 |  |
| C1orf159 | ENSG00000131591 | ENST00000542978 | (C)6 |
| C1QTNF5 | ENSG00000223953 | ENST00000490358 | (G)6 |
| C1S | ENSG00000182326 | ENST00000487113 | (C)6 |
|  |  | ENST00000382097 |  |
| C21orf62 | ENSG00000205929 | ENST00000417865 | (G)6 |
|  |  | ENST00000374023 |  |
| C22orf42 | ENSG00000205856 | ENST00000374026 | (T)6 |
| C2orf42 | ENSG00000115998 | ENST00000608326 | (G)6 |
| C6orf106 | ENSG00000196821 | ENST00000611854 | (C)6 |
|  |  | ENST00000376854 |  |
| C6orf163 | ENSG00000203872 | ENST00000372478 | (A)6 |
| C8orf59 | ENSG00000176731 | ENST00000382387 | (T)6 |
| C9orf40 | ENSG00000135045 | ENST00000591725 | (C)6 |
| C9orf50 | ENSG00000179058 | ENST00000335762 |  |
| C9orf66 | ENSG00000183784 | ENST00000399655 |  |
| CA4 | ENSG00000167434 | ENST00000637284 | (A)6 |
| CACNA1C | ENSG00000151067 | ENST00000637514 | (G)6 |
|  |  | ENST00000613879 |  |
| CACNB4 | ENSG00000182389 | ENST00000409670 | (A)6 |
|  |  | ENST00000409724 |  |
| CADPS | ENSG00000163618 | ENST00000541765 |  |
| CAPG | ENSG00000042493 | ENST00000543380 | (G)6 |
|  |  | ENST00000403299 |  |
| CAPRIN2 | ENSG00000110888 | ENST00000611932 | (A)6 |
|  |  | ENST00000614449 |  |
| CARD10 | ENSG00000100065 | ENST00000565287 | (C)6 |
| CARHSP1 | ENSG00000153048 | ENST00000569398 |  |
|  |  | ENST00000503684 |  |
|  |  | ENST00000621345 |  |
|  |  | ENST00000429617 |  |
| CASP6 | ENSG00000138794 | ENST00000369331 | (T)6 |
| CASP7 | ENSG00000165806 | ENST00000552562 | (C)6 |
|  |  | ENST00000602648 |  |
|  |  | ENST00000423344 |  |
| CBX5 | ENSG00000094916 | ENST00000389176 | (T)6 |
| CC2D2B | ENSG00000188649 | ENST00000278520 | (A)6 |
|  |  | ENST00000646818 |  |
| CCDC154 | ENSG00000197599 | ENST00000646638 | (C)6 |
| CCDC82 | ENSG00000149231 | ENST00000645366 | (A)6 |
|  |  | ENST00000645500 |  |
|  |  | ENST00000645402 |  |
|  |  | ENST00000647080 | (T)6 |
|  |  | ENST00000646050 | (A)6 |
|  |  | ENST00000643839 |  |
|  |  | ENST00000530106 |  |
|  |  | ENST00000530203 |  |
|  |  | ENST00000596938 |  |
|  |  | ENST00000513222 |  |
|  |  | ENST00000280326 | (T)6 |
| CCDC9 | ENSG00000105321 | ENST00000503026 | (G)6 |
| CCHCR1 | ENSG00000204536 | ENST00000515390 | (C)6 |
| CCT5 | ENSG00000150753 | ENST00000607597 | (G)6 |
|  |  | ENST00000606471 | (A)6 |
|  |  | ENST00000642928 | (G)6 |
| CD200 | ENSG00000091972 | ENST00000377795 | (A)6 |
|  |  | ENST00000353334 |  |
| CD59 | ENSG00000085063 | ENST00000492252 | (G)6 |
| CD74 | ENSG00000019582 | ENST00000400259 |  |
|  |  | ENST00000344548 |  |
| CD81 | ENSG00000110651 | ENST00000366766 |  |
| CDC42 | ENSG00000070831 | ENST00000561555 |  |
|  |  | ENST00000356200 | (C)6 |
| CDC42BPA | ENSG00000143776 | ENST00000378638 | (A)6 |
| CDIPT | ENSG00000103502 | ENST00000530151 | (T)6 |
| CDK11A | ENSG00000008128 | ENST00000526419 | (A)6 |
|  |  | ENST00000292672 |  |
| CELF1 | ENSG00000149187 | ENST00000379751 | (G)6 |
|  |  | ENST00000518853 |  |
| CELF5 | ENSG00000161082 | ENST00000519407 | (CCG)6 |
| CENPB | ENSG00000125817 | ENST00000522490 | (G)6 |
| CEP57L1 | ENSG00000183137 | ENST00000523209 | (A)6 |
|  |  | ENST00000301408 |  |
|  |  | ENST00000596965 |  |
|  |  | ENST00000597853 |  |
| CGB5 | ENSG00000189052 | ENST00000377280 | (C)6 |
| CGB7 | ENSG00000196337 | ENST00000448456 |  |
|  |  | ENST00000615216 |  |
|  |  | ENST00000445067 |  |
| CGB8 | ENSG00000213030 | ENST00000368476 |  |
| CHD9 | ENSG00000177200 | ENST00000637900 | (A)6 |
| CHI3L2 | ENSG00000064886 | ENST00000536961 | (T)6 |
| CHRNB2 | ENSG00000160716 | ENST00000553878 | (C)6 |
|  |  | ENST00000416322 |  |
| CINP | ENSG00000100865 | ENST00000437826 | (T)6 |
| CKB | ENSG00000166165 |  | (G)6 |
| CLDN12 | ENSG00000157224 | ENST00000402813 | (T)6 |
| CLPB | ENSG00000162129 | ENST00000551483  ENST00000523698 | (G)6 |
|  |  |  |  |
| CNGA1 | ENSG00000198515 | ENST00000636446 | (A)6 |
| CNOT2 | ENSG00000111596 | ENST00000547736 |  |
| CNOT8 | ENSG00000155508 | ENST00000314133 | (GC)6 |
| CNPY1 | ENSG00000146910 | ENST00000388925 | (A)6 |
| COQ5 | ENSG00000110871 | ENST00000566259 | (G)6 |
| COX8A | ENSG00000176340 | ENST00000505881 | (T)6 |
| CPAMD8 | ENSG00000160111 | ENST00000357727 | (C)6 |
| CPNE2 | ENSG00000140848 | ENST00000374721 |  |
| CPNE4 | ENSG00000196353 | ENST00000429130 | (T)6 |
| CREB5 | ENSG00000146592 | ENST00000543948 | (A)6 |
| CREM | ENSG00000095794 | ENST00000219599 |  |
|  |  | ENST00000569915 |  |
| CRYM | ENSG00000103316 | ENST00000373381 | (C)6 |
|  |  | ENST00000396832 |  |
| CSK | ENSG00000103653 | ENST00000403904 | (G)6 |
| CSMD2 | ENSG00000121904 | ENST00000413574 | (T)6 |
| CSNK1E | ENSG00000213923 | ENST00000405675 | (C)6 |
|  |  | ENST00000375865 |  |
|  |  | ENST00000542107 |  |
|  |  | ENST00000435290 |  |
| CSNK2B | ENSG00000204435 | ENST00000286758 | (G)6 |
| CX3CR1 | ENSG00000168329 | ENST00000409817 | (C)6 |
|  |  | ENST00000359293 |  |
| CXCL13 | ENSG00000156234 | ENST00000581573 | (A)6 |
| CXCR4 | ENSG00000121966 |  | (T)6 |
| CXorf40A | ENSG00000197620 | ENST00000369679 | (G)6 |
| CYB561 | ENSG00000008283 | ENST00000494864  ENST00000620214 |  |
|  |  |  |  |
| CYB5R4 | ENSG00000065615 | ENST00000556847 |  |
| CYP1B1 | ENSG00000138061 | ENST00000510307 | (A)6 |
| CYTH2 | ENSG00000105443 | ENST00000421670 |  |
| DCAF5 | ENSG00000139990 | ENST00000503715 |  |
| DCTD | ENSG00000129187 | ENST00000439211 | (C)6 |
| DDX55 | ENSG00000111364 | ENST00000505337 | (TG)6 |
| DEK | ENSG00000124795 | ENST00000520226 | (A)6 |
| DHFR | ENSG00000228716 | ENST00000503161 | (G)6 |
|  |  | ENST00000446837 |  |
| DLC1 | ENSG00000164741 | ENST00000338492 | (T)6 |
|  |  | ENST00000462981 |  |
| DNAI2 | ENSG00000171595 | ENST00000305904 | (C)6 |
| DPYSL4 | ENSG00000151640 | ENST00000327532 | (GCCC)6 |
| DSG1 | ENSG00000134760 | ENST00000469243 | (T)6 |
| DYNLRB2 | ENSG00000168589 | ENST00000467030 |  |
| DZIP1L | ENSG00000158163 | ENST00000405108 | (C)6 |
|  |  | ENST00000506135 |  |
|  |  | ENST00000636027 |  |
| EFR3B | ENSG00000084710 | ENST00000637318 | (T)6 |
| EGFLAM | ENSG00000164318 | ENST00000478940 | (G)6 |
| EHMT1 | ENSG00000181090 | ENST00000390013 | (A)6 |
|  |  | ENST00000395587 |  |
|  |  | ENST00000569690 |  |
| EIF2AK2 | ENSG00000055332 | ENST00000327921 | (GCG)6 |
| EIF3C | ENSG00000184110 | ENST00000556199 | (T)6 |
|  |  | ENST00000453258 |  |
| EML1 | ENSG00000066629 | ENST00000582703 | (C)6 |
|  |  | ENST00000405271 |  |
| ENTPD1 | ENSG00000138185 | ENST00000273854 | (T)6 |
| EPB41L3 | ENSG00000082397 | ENST00000613740 |  |
| EPCAM | ENSG00000119888 | ENST00000622150 | (C)6 |
| EPHA5 | ENSG00000145242 | ENST00000432638 | (G)6 |
|  |  | ENST00000354839 |  |
|  |  | ENST00000511294 |  |
|  |  | ENST00000493838 |  |
|  |  | ENST00000591636 |  |
|  |  | ENST00000013807 |  |
| EPHB1 | ENSG00000154928 | ENST00000589381 | (C)6 |
| ERCC1 | ENSG00000012061 | ENST00000589214 | (G)6 |
|  |  |  |  |
|  |  | ENST00000360012 |  |
|  |  | ENST00000615781  ENST00000538992 | (A)6 |
|  |  |  | (C)6 |
| ESRRG | ENSG00000196482 | ENST00000590078 | (T)6 |
| ETV7 | ENSG00000010030 | ENST00000476773 | (G)6 |
|  |  | ENST00000272610 | (C)6 |
| EZH1 | ENSG00000108799 | ENST00000630384 | (A)6 |
| EZH2 | ENSG00000106462 | ENST00000375662 | (C)6 |
| FAHD2B | ENSG00000144199 | ENST00000555748 | (GCC)6 |
| FAM120B | ENSG00000112584 | ENST00000510197 | (C)6 |
| FAM131C | ENSG00000185519 | ENST00000598389 |  |
| FAM174B | ENSG00000185442 | ENST00000526555 | (G)6 |
| FAM47E | ENSG00000189157 | ENST00000270509 | (A)6 |
| FARP1 | ENSG00000152767 | ENST00000424163 |  |
| FAU | ENSG00000149806 | ENST00000440021 | (G)6 |
| FBN3 | ENSG00000142449 | ENST00000395665 |  |
| FBXO11 | ENSG00000138081 | ENST00000308799 |  |
| FBXO34 | ENSG00000178974 | ENST00000301938 |  |
| FBXW10 | ENSG00000171931 | ENST00000525777 | (C)6 |
|  |  |  |  |
|  |  | ENST00000566739 |  |
| FDFT1 | ENSG00000079459 | ENST00000382333  ENST00000533668 | (A)6 |
|  |  |  | (T)6 |
| FEM1B | ENSG00000169018 | ENST00000510644 | (C)6 |
| FGFBP1 | ENSG00000137440 | ENST00000420362 |  |
| FGFR1 | ENSG00000077782 | ENST00000493452 | (GAT)6 |
| FGFRL1 | ENSG00000127418 | ENST00000319653 | (C)6 |
| FHL1 | ENSG00000022267 | ENST00000577128 |  |
| FLNB | ENSG00000136068 | ENST00000375123 | (A)6 |
| FMN2 | ENSG00000155816 | ENST00000525156 | (C)6 |
| FN3KRP | ENSG00000141560 | ENST00000531199 | (G)6 |
| FOXE1 | ENSG00000178919 | ENST00000423817 | (C)6 |
| FRMD8 | ENSG00000126391 | ENST00000511146 |  |
| FUT6 | ENSG00000156413 | ENST00000509992 | (A)6 |
| FXYD5 | ENSG00000089327 | ENST00000361507 | (C)6 |
| G3BP2 | ENSG00000138757 | ENST00000377016 |  |
| GAB1 | ENSG00000109458 | ENST00000377034 |  |
| GAB2 | ENSG00000033327 | ENST00000437025 | (G)6 |
| GABBR1 | ENSG00000204681 |  |  |
|  |  | ENST00000622697 |  |
| GABRA1 | ENSG00000022355 | ENST00000402369  ENST00000559454 |  |
|  |  |  |  |
| GABRB3 | ENSG00000166206 | ENST00000540933 | (T)6 |
| GAL3ST1 | ENSG00000128242 | ENST00000428622 | (G)6 |
| GALK2 | ENSG00000156958 | ENST00000580865 | (A)6 |
| GANAB | ENSG00000089597 | ENST00000583882 | (T)6 |
| GAS2L1 | ENSG00000185340 | ENST00000428024 | (G)6 |
| GAS7 | ENSG00000007237 | ENST00000247005 |  |
|  |  | ENST00000391951 |  |
| GBA | ENSG00000177628 | ENST00000464732 | (C)6 |
| GDF1 | ENSG00000130283 | ENST00000447581 |  |
| GEMIN7 | ENSG00000142252 | ENST00000559420 | (G)6 |
| GFM1 | ENSG00000168827 | ENST00000309971 | (A)6 |
| GJB1 | ENSG00000169562 | ENST00000372770 |  |
| GLCE | ENSG00000138604 | ENST00000550491 |  |
| GLE1 | ENSG00000119392 | ENST00000526351 | (G)6 |
|  |  | ENST00000366598 |  |
| GLIPR1 | ENSG00000139278 | ENST00000433809 | (T)6 |
| GLYATL1 | ENSG00000166840 | ENST00000507534 |  |
| GNG4 | ENSG00000168243 | ENST00000640608 | (C)6 |
| GNL1 | ENSG00000204590 | ENST00000638634 |  |
| GNPDA2 | ENSG00000163281 | ENST00000623037 | (A)6 |
| GOSR2 | ENSG00000108433 | ENST00000225567 | (G)6 |
|  |  | ENST00000415811 |  |
|  |  | ENST00000576910 |  |
|  |  | ENST00000640051 |  |
|  |  | ENST00000573224 |  |
|  |  | ENST00000534502 |  |
|  |  | ENST00000462900 |  |
|  |  | ENST00000566822 |  |
| GPR89A | ENSG00000117262 | ENST00000570142 | (T)6 |
|  |  | ENST00000534764 |  |
| GPRC5B | ENSG00000167191 | ENST00000389825 |  |
|  |  | ENST00000293190 |  |
| GRAMD1B | ENSG00000023171 | ENST00000509336 | (C)6 |
| GREB1 | ENSG00000196208 | ENST00000470836 |  |
| GRIN2C | ENSG00000161509 | ENST00000587560 | (G)6 |
| GSTCD | ENSG00000138780 |  | (A)6 |
| GTPBP1 | ENSG00000100226 | ENST00000251287 | (T)6 |
| H3F3B | ENSG00000132475 | ENST00000440653  ENST00000367010 | (C)6 |
|  |  |  | (G)6 |
| HCN2 | ENSG00000099822 | ENST00000503968 | (CGG)6 |
| HDAC6 | ENSG00000094631 | ENST00000366695 | (C)6 |
| HHAT | ENSG00000054392 | ENST00000324411 | (A)6 |
| HIPK1 | ENSG00000163349 | ENST00000590582 | (T)6 |
| HIST3H2A | ENSG00000181218 | ENST00000426847 |  |
| HKR1 | ENSG00000181666 | ENST00000558651 | (A)6 |
|  |  | ENST00000519684 | (C)6 |
| HLA-DRB3 | ENSG00000230463 | ENST00000522796 | (G)6 |
| HMG20A | ENSG00000140382 | ENST00000615446 | (C)6 |
| HMGB4 | ENSG00000176256 | ENST00000521790 | (A)6 |
|  |  | ENST00000600077 |  |
| HNF1A | ENSG00000135100 | ENST00000599097 | (C)6 |
| HNRNPH1 | ENSG00000169045 | ENST00000446825 | (A)6 |
| HOMER3 | ENSG00000051128 | ENST00000263278 | (C)6 |
|  |  | ENST00000370416 |  |
| HS1BP3 | ENSG00000118960 | ENST00000468935 | (T)6 |
| HSD17B14 | ENSG00000087076 | ENST00000345042 | (C)6 |
| HSFX1 | ENSG00000171116 | ENST00000430176 |  |
| HSPA12A | ENSG00000165868 | ENST00000443101 | (T)6 |
| HSPD1 | ENSG00000144381 | ENST00000531939 | (A)6 |
|  |  | ENST00000517866 |  |
| IFRD1 | ENSG00000006652 | ENST00000490985 |  |
| IFT46 | ENSG00000118096 | ENST00000405709 |  |
| IFT74 | ENSG00000096872 | ENST00000450877 |  |
| IL31RA | ENSG00000164509 | ENST00000617571 | (T)6 |
| IMMP2L | ENSG00000184903 | ENST00000375774 |  |
|  |  | ENST00000631485 | (C)6 |
| INF2 | ENSG00000203485 | ENST00000631555 | (G)6 |
| ING1 | ENSG00000153487 | ENST00000310227 | (C)6 |
| INPP5F | ENSG00000198825 | ENST00000543446 | (A)6 |
|  |  | ENST00000560418 |  |
| INSM1 | ENSG00000173404 | ENST00000431837 | (G)6 |
| IPO8 | ENSG00000133704 | ENST00000378076 | (A)6 |
| IQGAP1 | ENSG00000140575 | ENST00000433736 | (T)6 |
| IRAK4 | ENSG00000198001 | ENST00000414670 | (A)6 |
| ITGA8 | ENSG00000077943 | ENST00000523663 | (C)6 |
| ITGAV | ENSG00000138448 | ENST00000559060 |  |
| ITGB1 | ENSG00000150093 | ENST00000437176 | (A)6 |
| ITGB2 | ENSG00000160255 | ENST00000371222 | (TG)6 |
| JDP2 | ENSG00000140044 | ENST00000523207 | (C)6 |
|  |  | ENST00000529694 |  |
| JUN | ENSG00000177606 | ENST00000639601 | (G)6 |
| KCNB2 | ENSG00000182674 | ENST00000615047 | (T)6 |
| KCNJ5 | ENSG00000120457 | ENST00000355265 | (C)6 |
| KCNMA1 | ENSG00000156113 | ENST00000476829 | (GGC)6 |
| KCNN4 | ENSG00000104783 | ENST00000433730 | (G)6 |
| KEL | ENSG00000197993 | ENST00000251343 |  |
|  |  | ENST00000535378 |  |
| KHDC1 | ENSG00000135314 | ENST00000440579 | (C)6 |
| KHNYN | ENSG00000100441 | ENST00000572370 | (G)6 |
| KIAA0319 | ENSG00000137261 | ENST00000376462 | (C)6 |
| KIAA0319L | ENSG00000142687 | ENST00000430453 | (A)6 |
| KIAA0753 | ENSG00000198920 | ENST00000374118 |  |
| KIAA1217 | ENSG00000120549 | ENST00000561482 | (G)6 |
|  |  | ENST00000569636 |  |
| KIF12 | ENSG00000136883 | ENST00000400751 | (C)6 |
| KIF22 | ENSG00000079616 | ENST00000569382 |  |
|  |  | ENST00000156499 |  |
|  |  | ENST00000326856 |  |
|  |  | ENST00000325321 |  |
| KLK14 | ENSG00000129437 | ENST00000600690 |  |
| KLK15 | ENSG00000174562 | ENST00000594641 | (A)6 |
| KLK2 | ENSG00000167751 | ENST00000544265 | (C)6 |
|  |  | ENST00000570980 |  |
| KLK6 | ENSG00000167755 | ENST00000470811 | (G)6 |
| KRT85 | ENSG00000135443 | ENST00000379992 | (A)6 |
| LCAT | ENSG00000213398 | ENST00000308675 | (G)6 |
| LEKR1 | ENSG00000197980 | ENST00000599783 | (A)6 |
| LINGO2 | ENSG00000174482 | ENST00000533809 |  |
|  |  | ENST00000590729 |  |
| LIPE | ENSG00000079435 | ENST00000389484 | (G)6 |
| LMO7 | ENSG00000136153 | ENST00000434794 | (A)6 |
| LONP1 | ENSG00000196365 | ENST00000328559 | (G)6 |
| LRP1B | ENSG00000168702 | ENST00000569499 | (GCC)6 |
|  |  | ENST00000568804 |  |
| LRP2BP | ENSG00000109771 | ENST00000537729 | (T)6 |
| LRRC36 | ENSG00000159708 | ENST00000518304 | (A)6 |
|  |  | ENST00000345363 |  |
| LRRC43 | ENSG00000158113 | ENST00000443357 | (C)6 |
| LRRC69 | ENSG00000214954 | ENST00000354212 | (T)6 |
| LTB4R | ENSG00000213903 | ENST00000419488 | (C)6 |
| LUC7L | ENSG00000007392 | ENST00000637441 | (T)6 |
| MAGI2 | ENSG00000187391 | ENST00000522391 | (C)6 |
|  |  | ENST00000509479 |  |
|  |  | ENST00000543674 |  |
|  |  | ENST00000540169 |  |
| MAML3 | ENSG00000196782 | ENST00000587605 | (G)6 |
| MARK2 | ENSG00000072518 | ENST00000585595 | (T)6 |
|  |  | ENST00000431606 |  |
| MBD1 | ENSG00000141644 | ENST00000337672 | (G)6 |
|  |  | ENST00000598608 |  |
| MED19 | ENSG00000156603 | ENST00000292035 | (A)6 |
|  |  | ENST00000372455 |  |
| MED26 | ENSG00000105085 | ENST00000559085 | (G)6 |
| MED27 | ENSG00000160563 | ENST00000577166 | (T)6 |
| MED8 | ENSG00000159479 | ENST00000489329 | (A)6 |
| MEIS2 | ENSG00000134138 | ENST00000497673 | (C)6 |
| METTL4 | ENSG00000101574 |  | (G)6 |
| MFN1 | ENSG00000171109 | ENST00000539062 | (A)6 |
| MGAM | ENSG00000257335 | ENST00000407029  ENST00000402630 | (T)6 |
|  |  |  | (A)6 |
| MIPOL1 | ENSG00000151338 | ENST00000482628 | (T)6 |
| MKL1 | ENSG00000196588 |  |  |
|  |  | ENST00000478894 |  |
| MLF1 | ENSG00000178053 | ENST00000565250  ENST00000473730 | (G)6 |
|  |  |  |  |
|  |  | ENST00000462837 | (A)6 |
| MLST8 | ENSG00000167965 | ENST00000508731 | (C)6 |
| MME | ENSG00000196549 | ENST00000526712 | (A)6 |
|  |  | ENST00000569610 |  |
| MND1 | ENSG00000121211 | ENST00000520305 | (T)6 |
| MOGAT2 | ENSG00000166391 | ENST00000357186 | (G)6 |
| MON1B | ENSG00000103111 | ENST00000252602 | (C)6 |
| MPP2 | ENSG00000108852 | ENST00000519120 |  |
| MPV17 | ENSG00000115204 | ENST00000582920 | (A)6 |
| MRPL34 | ENSG00000130312 | ENST00000521209 | (T)6 |
| MRPS28 | ENSG00000147586 | ENST00000455339 | (A)6 |
| MSL1 | ENSG00000188895 | ENST00000452359 | (G)6 |
| MSRA | ENSG00000175806 | ENST00000529681 | (A)6 |
| MTOR | ENSG00000198793 | ENST00000539696 | (T)6 |
| MTRF1 | ENSG00000120662 | ENST00000435990 | (A)6 |
| MUC5B | ENSG00000117983 | ENST00000418404 | (C)6 |
| MVK | ENSG00000110921 | ENST00000532183 |  |
| MXD1 | ENSG00000059728 | ENST00000223167 | (A)6 |
| MYH13 | ENSG00000006788 | ENST00000418908 | (G)6 |
| MYH2 | ENSG00000125414 | ENST00000302541 | (T)6 |
| MYL10 | ENSG00000106436 | ENST00000396217 |  |
| MYO1B | ENSG00000128641 | ENST00000425621 | (G)6 |
| MYRIP | ENSG00000170011 | ENST00000529840 | (C)6 |
|  |  | ENST00000530055 | (A)6 |
|  |  | ENST00000415280 | (C)6 |
| NADSYN1 | ENSG00000172890 | ENST00000544816 | (T)6 |
|  |  | ENST00000551524 |  |
| NANS | ENSG00000095380 | ENST00000554415 | (A)6 |
| NAP1L1 | ENSG00000187109 | ENST00000470123 |  |
|  |  | ENST00000423583 |  |
| NDRG2 | ENSG00000165795 | ENST00000413692 | (G)6 |
| NDUFA5 | ENSG00000128609 | ENST00000554591 | (A)6 |
| NEU4 | ENSG00000204099 | ENST00000510170 | (C)6 |
| NFATC4 | ENSG00000100968 | ENST00000482491 | (TG)6 |
|  |  | ENST00000597836 |  |
| NR3C1 | ENSG00000113580 | ENST00000416608 | (G)6 |
| NVL | ENSG00000143748 | ENST00000550708 | (T)6 |
| OCEL1 | ENSG00000099330 | ENST00000641517 | (C)6 |
| OCM | ENSG00000122543 | ENST00000318244 | (G)6 |
| OR11H12 | ENSG00000257115 | ENST00000641802 | (A)6 |
| OR11H2 | ENSG00000258453 | ENST00000366487 |  |
| OR2AJ1 | ENSG00000177275 | ENST00000641211 |  |
| OR2C3 | ENSG00000196242 | ENST00000307033 | (T)6 |
|  |  | ENST00000641896 |  |
| OR2M2 | ENSG00000198601 | ENST00000641486 |  |
| OR4D5 | ENSG00000171014 | ENST00000641905 |  |
| OR52I2 | ENSG00000226288 | ENST00000641160 | (A)6 |
|  |  | ENST00000641850 |  |
| OR56A3 | ENSG00000184478 | ENST00000358433 |  |
|  |  | ENST00000248073 |  |
| OR5AN1 | ENSG00000176495 | ENST00000641707 | (T)6 |
| OR6C6 | ENSG00000188324 | ENST00000325795 | (A)6 |
| OR7C1 | ENSG00000127530 | ENST00000458355 | (T)6 |
| OR8G5 | ENSG00000255298 | ENST00000428266 |  |
| ORMDL1 | ENSG00000128699 | ENST00000536582 | (A)6 |
|  |  | ENST00000421491 |  |
| OXSM | ENSG00000151093 | ENST00000453643 |  |
| PAAF1 | ENSG00000175575 | ENST00000508537 | (T)6 |
| PABPC4L | ENSG00000254535 | ENST00000602004 | (C)6 |
| PACSIN2 | ENSG00000100266 | ENST00000568219 | (A)6 |
| PAIP1 | ENSG00000172239 |  |  |
| PAK4 | ENSG00000130669 | ENST00000547572 | (G)6 |
| PALB2 | ENSG00000083093 | ENST00000576565  ENST00000562220 | (A)6 |
|  |  |  |  |
| PAN2 | ENSG00000135473 | ENST00000614258 | (T)6 |
| PAQR4 | ENSG00000162073 | ENST00000529619 | (CCG)6 |
| PCDHA10 | ENSG00000250120 | ENST00000416255 | (A)6 |
| PCDHA5 | ENSG00000204965 | ENST00000542223 |  |
|  |  | ENST00000371045 |  |
| PDCL3 | ENSG00000115539 |  | (C)6 |
| PDE2A | ENSG00000186642 | ENST00000533550 | (A)6 |
| PDE4B | ENSG00000184588 | ENST00000429812  ENST00000566543 | (G)6 |
|  |  |  |  |
| PDHX | ENSG00000110435 | ENST00000311765 |  |
| PDLIM2 | ENSG00000120913 | ENST00000561704 | (C)6 |
| PDP2 | ENSG00000172840 | ENST00000568398 | (T)6 |
|  |  | ENST00000566776 |  |
|  |  | ENST00000563522 |  |
|  |  | ENST00000481275 |  |
|  |  | ENST00000419155 |  |
| PDXDC1 | ENSG00000179889 | ENST00000529734 | (C)6 |
| PFN2 | ENSG00000070087 | ENST00000430292 | (T)6 |
| PHF19 | ENSG00000119403 | ENST00000372006 | (G)6 |
| PHF21A | ENSG00000135365 | ENST00000546049 |  |
| PIGV | ENSG00000060642 | ENST00000535956 |  |
| PIK3R3 | ENSG00000117461 | ENST00000334686 | (C)6 |
| PITPNM2 | ENSG00000090975 | ENST00000594195 | (G)6 |
| PIWIL1 | ENSG00000125207 | ENST00000624204 | (C)6 |
| PLCH1 | ENSG00000114805 | ENST00000432292 | (A)6 |
| PLEKHA4 | ENSG00000105559 | ENST00000421798 | (C)6 |
| PMS1 | ENSG00000064933 | ENST00000269582 | (A)6 |
|  |  | ENST00000449930 |  |
| PNMA6A | ENSG00000235961 | ENST00000520008 | (C)6 |
| PNMT | ENSG00000141744 |  | (A)6 |
| POGK | ENSG00000143157 | ENST00000399696 | (CGC)6 |
| POLB | ENSG00000070501 | ENST00000404875  ENST00000441334 | (A)6 |
|  |  |  |  |
| POLR1D | ENSG00000186184 | ENST00000415075 |  |
| POMT1 | ENSG00000130714 | ENST00000430619 | (T)6 |
|  |  | ENST00000286719 |  |
|  |  | ENST00000440068 |  |
|  |  | ENST00000334933 |  |
| PPEF2 | ENSG00000156194 | ENST00000417085 | (A)6 |
| PPIH | ENSG00000171960 | ENST00000590418 | (G)6 |
| PPIP5K1 | ENSG00000168781 | ENST00000435544 | (C)6 |
|  |  | ENST00000394267 |  |
| PPM1D | ENSG00000170836 | ENST00000534631 | (A)6 |
| PPP1R12C | ENSG00000125503 | ENST00000555185 | (C)6 |
| PPP1R1B | ENSG00000131771 | ENST00000415329 | (G)6 |
| PRCP | ENSG00000137509 | ENST00000622405 |  |
| PRKCH | ENSG00000027075 | ENST00000581842 | (T)6 |
| PROCA1 | ENSG00000167525 | ENST00000585123 | (C)6 |
| PSMA6 | ENSG00000100902 | ENST00000580864 | (T)6 |
| PSMC5 | ENSG00000087191 | ENST00000375812 | (C)6 |
|  |  | ENST00000370931 |  |
|  |  | ENST00000628037 |  |
|  |  | ENST00000370932 |  |
| PTGER3 | ENSG00000050628 | ENST00000356595 | (G)6 |
|  |  | ENST00000306666 |  |
|  |  | ENST00000351052 |  |
|  |  | ENST00000354608 |  |
|  |  | ENST00000367379 |  |
|  |  | ENST00000303270 |  |
|  |  | ENST00000481914 |  |
| PTPRC | ENSG00000081237 | ENST00000218249 | (T)6 |
| RAB24 | ENSG00000169228 | ENST00000325589 | (G)6 |
| RAB3A | ENSG00000105649 | ENST00000549777 | (C)6 |
| RAB40AL | ENSG00000102128 | ENST00000550651 | (G)6 |
| RABGAP1L | ENSG00000152061 | ENST00000393371 | (A)6 |
| RACGAP1 | ENSG00000161800 | ENST00000553876 | (G)6 |
|  |  | ENST00000425446 |  |
| RASGEF1C | ENSG00000146090 | ENST00000393897 |  |
| RBM23 | ENSG00000100461 | ENST00000393580 | (A)6 |
| RBM24 | ENSG00000112183 | ENST00000354373 | (C)6 |
| REG3G | ENSG00000143954 |  | (A)6 |
| RELT | ENSG00000054967 | ENST00000248980 | (G)6 |
| RFPL1 | ENSG00000128250 | ENST00000249007 |  |
|  |  |  |  |
| RFPL2 | ENSG00000128253 | ENST00000316163 |  |
|  |  |  |  |
| RFPL3 | ENSG00000128276 | ENST00000262316  ENST00000619374 |  |
|  |  |  |  |
| RGS11 | ENSG00000076344 | ENST00000356143 | (C)6 |
| RHBDF1 | ENSG00000007384 | ENST00000521606 |  |
| RIMKLB | ENSG00000166532 | ENST00000368414 | (T)6 |
| RNF14 | ENSG00000013561 | ENST00000371080 |  |
| RNF145 | ENSG00000145860 | ENST00000371079 |  |
| RNF217 | ENSG00000146373 | ENST00000429921 | (A)6 |
| ROR1 | ENSG00000185483 | ENST00000453724 | (GCC)6 |
|  |  | ENST00000441588 |  |
| RPE | ENSG00000197713 | ENST00000326199 | (T)6 |
|  |  | ENST00000526522 |  |
|  |  | ENST00000529806 |  |
| RPS10 | ENSG00000124614 | ENST00000521262 | (G)6 |
| RPS2 | ENSG00000140988 | ENST00000523936 |  |
|  |  | ENST00000596046 |  |
| RPS20 | ENSG00000008988 | ENST00000315289 |  |
|  |  | ENST00000526230 |  |
| RPS5 | ENSG00000083845 | ENST00000398846 | (C)6 |
| RTKN2 | ENSG00000182010 | ENST00000533683 | (A)6 |
| S100PBP | ENSG00000116497 |  |  |
| SAC3D1 | ENSG00000168061 | ENST00000637729 | (C)6 |
| SAMD1 | ENSG00000141858 | ENST00000601038  ENST00000377533 |  |
|  |  |  | (G)6 |
| SASH1 | ENSG00000111961 |  |  |
| SCAF1 | ENSG00000126461 | ENST00000404826 | (C)6 |
| SCRIB | ENSG00000180900 | ENST00000429917  ENST00000419255 | (T)6 |
|  |  |  |  |
| SDK1 | ENSG00000146555 | ENST00000368282 | (G)6 |
| SEC14L2 | ENSG00000100003 | ENST00000431153 | (C)6 |
| SEMA3C | ENSG00000075223 | ENST00000441827 | (A)6 |
| SEMA4A | ENSG00000196189 | ENST00000571149 | (G)6 |
| SERPINB13 | ENSG00000197641 | ENST00000525611 | (T)6 |
| SERPINB8 | ENSG00000166401 | ENST00000379845 | (G)6 |
| SERPINF1 | ENSG00000132386 | ENST00000417041 |  |
| SERPINH1 | ENSG00000149257 | ENST00000237305 | (A)6 |
| SFRP1 | ENSG00000104332 | ENST00000427482 | (G)6 |
| SFTPA2 | ENSG00000185303 | ENST00000644750 | (C)6 |
| SGK1 | ENSG00000118515 | ENST00000564778 | (T)6 |
| SH3GL3 | ENSG00000140600 | ENST00000568190 | (G)6 |
| SIK1 | ENSG00000142178 | ENST00000414941 |  |
| SIN3A | ENSG00000169375 | ENST00000407552 |  |
|  |  | ENST00000587091 | (T)6 |
| SIRT2 | ENSG00000068903 | ENST00000393265 | (A)6 |
|  |  | ENST00000522438 |  |
| SLC16A2 | ENSG00000147100 | ENST00000535344 | (C)6 |
| SLC24A4 | ENSG00000140090 | ENST00000369556 | (T)6 |
| SLC25A30 | ENSG00000174032 | ENST00000369557 |  |
| SLC2A14 | ENSG00000173262 | ENST00000369552 | (G)6 |
| SLC35A1 | ENSG00000164414 | ENST00000513993 |  |
|  |  | ENST00000418005 |  |
|  |  | ENST00000527091 |  |
| SLC38A9 | ENSG00000177058 | ENST00000425938 | (T)6 |
| SLC39A10 | ENSG00000196950 | ENST00000644314 | (C)6 |
| SLC39A13 | ENSG00000165915 | ENST00000404574 | (A)6 |
| SLC6A1 | ENSG00000157103 | ENST00000601545 | (G)6 |
|  |  | ENST00000564644 |  |
| SMTN | ENSG00000183963 | ENST00000563793 | (C)6 |
| SNRPA | ENSG00000077312 | ENST00000412416 | (A)6 |
| SNUPN | ENSG00000169371 | ENST00000428383 |  |
|  |  | ENST00000458341 |  |
| SNX10 | ENSG00000086300 | ENST00000341656 |  |
| SNX31 | ENSG00000174226 | ENST00000557795 |  |
| SP140L | ENSG00000185404 | ENST00000593308 |  |
| SPDYE2 | ENSG00000205238 | ENST00000374884 | (C)6 |
| SPG21 | ENSG00000090487 | ENST00000257100 | (T)6 |
| SPHK2 | ENSG00000063176 | ENST00000528791 | (G)6 |
| SPIN4 | ENSG00000186767 | ENST00000338502 | (A)6 |
| SPOCD1 | ENSG00000134668 | ENST00000574987 | (G)6 |
|  |  | ENST00000370949 |  |
| SPRED3 | ENSG00000188766 | ENST00000381962 | (C)6 |
| SRR | ENSG00000167720 | ENST00000599699 | (A)6 |
| SRSF11 | ENSG00000116754 | ENST00000409136 |  |
| SRXN1 | ENSG00000271303 | ENST00000480567 | (G)6 |
| SSBP4 | ENSG00000130511 | ENST00000531917 | (C)6 |
| SSFA2 | ENSG00000138434 | ENST00000371924 | (CAA)6 |
| SSR2 | ENSG00000163479 | ENST00000371922 | (G)6 |
|  |  | ENST00000424722 |  |
| STAMBPL1 | ENSG00000138134 | ENST00000288513 | (A)6 |
|  |  | ENST00000436657 |  |
| STAT1 | ENSG00000115415 | ENST00000378644 | (G)6 |
| SVOPL | ENSG00000157703 | ENST00000600252 | (T)6 |
|  |  | ENST00000555241 |  |
| SYCE1L | ENSG00000205078 | ENST00000636905 |  |
| SYDE1 | ENSG00000105137 | ENST00000275191 | (C)6 |
| SYNE2 | ENSG00000054654 | ENST00000636456 | (T)6 |
| SYNGAP1 | ENSG00000197283 | ENST00000514539 | (GGC)6 |
| TAAR2 | ENSG00000146378 | ENST00000320307 | (T)6 |
| TAB2 | ENSG00000055208 | ENST00000473118 | (G)6 |
| TACC2 | ENSG00000138162 | ENST00000616041 | (T)6 |
| TAGLN2 | ENSG00000158710 | ENST00000382492 | (C)6 |
| TARDBP | ENSG00000120948 |  | (G)6 |
| TARM1 | ENSG00000248385 | ENST00000523888 | (GAAA)6 |
| TAS2R1 | ENSG00000169777 | ENST00000615497  ENST00000410035 | (T)6 |
|  |  |  |  |
| TATDN1 | ENSG00000147687 | ENST00000537390 | (G)6 |
| TBC1D1 | ENSG00000065882 | ENST00000536394 | (C)6 |
| TBR1 | ENSG00000136535 | ENST00000392793 | (A)6 |
| TCP1 | ENSG00000120438 | ENST00000207457 |  |
|  |  | ENST00000549468 |  |
| TECTA | ENSG00000109927 | ENST00000400167 | (T)6 |
| TEKT2 | ENSG00000092850 | ENST00000313516 | (G)6 |
| TGIF1 | ENSG00000177426 | ENST00000585338 | (T)6 |
|  |  | ENST00000447875 |  |
| THAP5 | ENSG00000177683 | ENST00000428492 | (C)6 |
| THOP1 | ENSG00000172009 | ENST00000418247 |  |
| THRB | ENSG00000151090 | ENST00000412524 | (G)6 |
|  |  |  |  |
|  |  | ENST00000248244 |  |
| TIAL1 | ENSG00000151923 | ENST00000481065  ENST00000473702 | (A)6 |
|  |  |  | (GAAA)6 |
| TICAM1 | ENSG00000127666 | ENST00000568335 | (G)6 |
| TIGIT | ENSG00000181847 | ENST00000544065 | (A)6 |
| TIPARP | ENSG00000163659 |  | (T)6 |
| TMEM114 | ENSG00000232258 | ENST00000569540 | (G)6 |
| TMEM132A | ENSG00000006118 | ENST00000250930  ENST00000427313 |  |
|  |  |  | (C)6 |
| TMEM170A | ENSG00000166822 | ENST00000380635 | (G)6 |
| TMEM8A | ENSG00000129925 | ENST00000482202 | (C)6 |
|  |  | ENST00000486728 |  |
| TMSB4X | ENSG00000205542 | ENST00000520931 | (A)6 |
| TMUB1 | ENSG00000164897 | ENST00000518281 | (G)6 |
| TNFRSF18 | ENSG00000186891 | ENST00000530920 |  |
| TNIP1 | ENSG00000145901 | ENST00000563091 |  |
| TNKS | ENSG00000173273 | ENST00000461509 | (T)6 |
| TNKS1BP1 | ENSG00000149115 | ENST00000211076 | (G)6 |
| TOX3 | ENSG00000103460 | ENST00000317378 |  |
| TPSAB1 | ENSG00000172236 | ENST00000426877 | (C)6 |
| TPSD1 | ENSG00000095917 | ENST00000414641 |  |
| TRAPPC5 | ENSG00000181029 | ENST00000369461 |  |
|  |  | ENST00000422067 |  |
| TRIM22 | ENSG00000132274 | ENST00000437766 | (A)6 |
| TRIM45 | ENSG00000134253 | ENST00000221504 | (C)6 |
| TRIM7 | ENSG00000146054 | ENST00000433712 |  |
| TRMT1 | ENSG00000104907 | ENST00000434205 |  |
|  |  | ENST00000333090 |  |
| TRPM8 | ENSG00000144481 | ENST00000492617 | (T)6 |
| TSEN54 | ENSG00000182173 | ENST00000495660 | (G)6 |
| TSKU | ENSG00000182704 | ENST00000532583 |  |
| TTC14 | ENSG00000163728 | ENST00000426827 | (A)6 |
|  |  | ENST00000416740 |  |
| TTC9C | ENSG00000162222 | ENST00000395839 | (G)6 |
| TTLL3 | ENSG00000214021 |  | (C)6 |
| TUBA8 | ENSG00000183785 | ENST00000536769 | (G)6 |
| UBB | ENSG00000170315 | ENST00000503431 |  |
|  |  |  | (GC)6 |
| UBC | ENSG00000150991 | ENST00000506179  ENST00000507089 | (T)6 |
|  |  |  |  |
| UCHL1 | ENSG00000154277 | ENST00000472047 | (G)6 |
| UGDH | ENSG00000109814 | ENST00000612630 |  |
|  |  | ENST00000453304 | (A)6 |
| UGP2 | ENSG00000169764 | ENST00000513796 | (T)6 |
| UHRF1 | ENSG00000276043 | ENST00000506749 | (C)6 |
| UNC5C | ENSG00000182168 | ENST00000504962 |  |
|  |  | ENST00000548836 |  |
|  |  | ENST00000465073 |  |
|  |  | ENST00000549639 |  |
| USP15 | ENSG00000135655 | ENST00000338981 | (A)6 |
| USP42 | ENSG00000106346 | ENST00000366967 | (GCG)6 |
| USP44 | ENSG00000136014 | ENST00000458499 | (C)6 |
| USP9Y | ENSG00000114374 | ENST00000433647 | (A)6 |
| VASH2 | ENSG00000143494 | ENST00000489794 | (AGC)6 |
| VGLL4 | ENSG00000144560 | ENST00000354504 | (A)6 |
| VIPR1 | ENSG00000114812 | ENST00000553395 | (T)6 |
| VPS26A | ENSG00000122958 | ENST00000556338 |  |
| VPS54 | ENSG00000143952 | ENST00000428943 | (A)6 |
| WARS | ENSG00000140105 | ENST00000488333 | (C)6 |
|  |  | ENST00000596159 |  |
| WBP1 | ENSG00000239779 | ENST00000551638 | (G)6 |
| WDR78 | ENSG00000152763 | ENST00000566780 | (T)6 |
| WIZ | ENSG00000011451 | ENST00000627394 | (G)6 |
| WSCD2 | ENSG00000075035 | ENST00000402655 | (T)6 |
| WWOX | ENSG00000186153 | ENST00000406884 | (G)6 |
|  |  | ENST00000539474 |  |
|  |  | ENST00000569818 |  |
|  |  | ENST00000355860 |  |
|  |  | ENST00000408984 |  |
|  |  | ENST00000395946 |  |
|  |  | ENST00000409043 |  |
|  |  | ENST00000409728 |  |
| XCR1 | ENSG00000173578 | ENST00000409195 | (C)6 |
| XIRP2 | ENSG00000163092 | ENST00000331428 | (A)6 |
|  |  | ENST00000562532 |  |
|  |  | ENST00000400935 |  |
| XKR3 | ENSG00000172967 | ENST00000395953 |  |
| XKR7 | ENSG00000260903 | ENST00000395948 | (C)6 |
| XPOT | ENSG00000184575 | ENST00000613979 | (T)6 |
| YWHAZ | ENSG00000164924 | ENST00000620596 | (CGG)6 |
|  |  | ENST00000618565 | (A)6 |
| ZAN | ENSG00000146839 | ENST00000557607 | (G)6 |
|  |  | ENST00000318308 |  |
|  |  | ENST00000555900 |  |
| ZC3H14 | ENSG00000100722 | ENST00000406216 | (A)6 |
|  |  | ENST00000557737 |  |
|  |  | ENST00000409871 |  |
|  |  | ENST00000343936 |  |
|  |  | ENST00000396516 |  |
| ZC3H6 | ENSG00000188177 | ENST00000334554 | (G)6 |
|  |  | ENST00000509562 |  |
| ZC3H7A | ENSG00000122299 | ENST00000593711 | (T)6 |
| ZDHHC8 | ENSG00000099904 | ENST00000629319 | (CGC)6 |
| ZFYVE16 | ENSG00000039319 | ENST00000601070 | (A)6 |
| ZIM2 | ENSG00000269699 | ENST00000599935 |  |
|  |  | ENST00000441447 |  |
|  |  | ENST00000373333 |  |
|  |  | ENST00000544426 |  |
| ZMYM4 | ENSG00000146463 | ENST00000414351 |  |
| ZMYM6 | ENSG00000163867 | ENST00000580488 | (C)6 |
| ZNF140 | ENSG00000196387 | ENST00000594668 | (A)6 |
| ZNF205 | ENSG00000122386 | ENST00000589208 | (G)6 |
| ZNF521 | ENSG00000198795 | ENST00000392150 | (C)6 |
| ZNF548 | ENSG00000188785 | ENST00000391794 | (T)6 |
| ZNF559 | ENSG00000188321 | ENST00000599683 |  |
| ZNF569 | ENSG00000196437 | ENST00000391785 |  |
| ZNF613 | ENSG00000176024 | ENST00000616183 |  |
|  |  | ENST00000595773 |  |
| ZNF701 | ENSG00000167562 | ENST00000615484 | (A)6 |
| ZNF714 | ENSG00000160352 | ENST00000527645 |  |
| ZNF780A | ENSG00000197782 | ENST00000565516 | (G)6 |
| ZNF790 | ENSG00000197863 | ENST00000586121 |  |
|  |  | ENST00000372523 |  |
| ZNF821 | ENSG00000102984 | ENST00000432293 | (T)6 |
| ZNF823 | ENSG00000197933 | ENST00000559241 | (C)6 |
| ZSWIM1 | ENSG00000168612 | ENST00000523400 | (A)6 |
| SEPT7 | ENSG00000122545 | ENST00000560468 |  |
| ACSBG1 | ENSG00000103740 | ENST00000558169 | (G)6 |
| ADAM32 | ENSG00000197140 | ENST00000421665 | (A)6 |
| ANXA2 | ENSG00000182718 | ENST00000617612 |  |
|  |  | ENST00000574974 |  |
| BAIAP3 | ENSG00000007516 | ENST00000592864 | (G)6 |
| C11orf96 | ENSG00000187479 | ENST00000543114 |  |
| CACNA1A | ENSG00000141837 | ENST00000588780 | (C)6 |
|  |  | ENST00000377392 |  |
| CACNA1C | ENSG00000151067 | ENST00000549606 | (A)6 |
| CAPNS1 | ENSG00000126247 | ENST00000553878 | (T)6 |
| CBWD5 | ENSG00000147996 | ENST00000493259 |  |
| CDK4 | ENSG00000135446 | ENST00000543464 | (C)6 |
| CKB | ENSG00000166165 | ENST00000368336 | (G)6 |
| CPA4 | ENSG00000128510 | ENST00000444280 | (T)6 |
| CPD | ENSG00000108582 | ENST00000514111 | (A)6 |
| DAP3 | ENSG00000132676 | ENST00000590018 | (T) |
| DHRSX | ENSG00000169084 | ENST00000578503 | (C)6 |
| EMB | ENSG00000170571 | ENST00000538992 | (A)6 |
| EML2 | ENSG00000125746 | ENST00000380274 |  |
| EPB41L3 | ENSG00000082397 | ENST00000372572 | (T)6 |
| ETV7 | ENSG00000010030 | ENST00000545068 |  |
| FAM50B | ENSG00000145945 | ENST00000510861 | (C)6 |
| FOXJ3 | ENSG00000198815 | ENST00000559932 | (A)6 |
|  |  | ENST00000378692 |  |
| GABRA2 | ENSG00000151834 | ENST00000554230 |  |
| GCHFR | ENSG00000137880 | ENST00000300098 |  |
| GLIPR1L2 | ENSG00000180481 | ENST00000510901 |  |
| GNPNAT1 | ENSG00000100522 | ENST00000506910 | (T)6 |
| GPR182 | ENSG00000166856 | ENST00000514584 | (C)6 |
| HPGD | ENSG00000164120 | ENST00000468935 | (A)6 |
|  |  | ENST00000533540 |  |
|  |  | ENST00000534624 |  |
| HSPA12A | ENSG00000165868 | ENST00000453788 | (G)6 |
| HSPA8 | ENSG00000109971 | ENST00000525463 | (T)6 |
|  |  | ENST00000544829 |  |
|  |  | ENST00000638749 |  |
|  |  | ENST00000420176 |  |
| IPO8 | ENSG00000133704 | ENST00000536814 | (A)6 |
| IQCJ-SCHIP1 | ENSG00000283154 | ENST00000547825 | (T)6 |
| ITPRIPL1 | ENSG00000198885 | ENST00000533809 | (A)6 |
|  |  | ENST00000443357 |  |
| LIMA1 | ENSG00000050405 | ENST00000530275 |  |
| LMO7 | ENSG00000136153 | ENST00000366735 |  |
| LUC7L | ENSG00000007392 | ENST00000223167 | (G)6 |
| MACF1 | ENSG00000127603 | ENST00000554903 |  |
| MRPL55 | ENSG00000162910 | ENST00000575301 | (A)6 |
| MYL10 | ENSG00000106436 | ENST00000359242 | (T)6 |
| NFATC4 | ENSG00000100968 | ENST00000479890 | (A)6 |
| NLGN2 | ENSG00000169992 | ENST00000641114 | (C)6 |
| ODF2L | ENSG00000122417 | ENST00000446524 | (T)6 |
|  |  | ENST00000642104 |  |
| OR2AP1 | ENSG00000179615 | ENST00000452681 |  |
| OR4A47 | ENSG00000237388 | ENST00000537360 | (C)6 |
| OR6C1 | ENSG00000205330 | ENST00000297373 | (A)6 |
| PHKG1 | ENSG00000164776 | ENST00000446428 | (G)6 |
|  |  | ENST00000432123 |  |
|  |  | ENST00000447555 |  |
|  |  | ENST00000494598 |  |
|  |  | ENST00000498739 |  |
| PIP5K1A | ENSG00000143398 | ENST00000545225 | (T)6 |
| PLCH1 | ENSG00000114805 | ENST00000586114 | (C)6 |
| PRKCB | ENSG00000166501 | ENST00000585805 |  |
| PSME3 | ENSG00000131467 | ENST00000591152 | (T)6 |
|  |  | ENST00000518055 |  |
|  |  | ENST00000593230 |  |
|  |  | ENST00000455270 |  |
| RAD21 | ENSG00000164754 | ENST00000296277 | (A)6 |
| RNF165 | ENSG00000141622 | ENST00000380384 | (C)6 |
| RPL39L | ENSG00000163923 | ENST00000397109 | (A)6 |
|  |  | ENST00000436949 |  |
| RPS6 | ENSG00000137154 | ENST00000521960 | (C)6 |
| SEC13 | ENSG00000157020 | ENST00000406900 | (G)6 |
| SEMA3A | ENSG00000075213 | ENST00000584995 | (T)6 |
| SGK3 | ENSG00000104205 | ENST00000261367 | (A)6 |
| SIRT1 | ENSG00000096717 | ENST00000412416 | (G)6 |
| SLC46A1 | ENSG00000076351 | ENST00000347458 |  |
| SNCAIP | ENSG00000064692 | ENST00000371802 | (A)6 |
| SNX10 | ENSG00000086300 | ENST00000371792 |  |
| STAU1 | ENSG00000124214 | ENST00000369001 | (T)6 |
|  |  | ENST00000369000 |  |
|  |  | ENST00000396862 |  |
| TACC2 | ENSG00000138162 | ENST00000549919 | (C)6 |
|  |  | ENST00000478943 |  |
| TIGD7 | ENSG00000140993 | ENST00000583940 | (A)6 |
| TMTC2 | ENSG00000179104 | ENST00000580183 | (T)6 |
| TOM1L2 | ENSG00000175662 | ENST00000578749 |  |
| TP53I13 | ENSG00000167543 | ENST00000582829 | (C)6 |
|  |  | ENST00000341495 |  |
|  |  | ENST00000448827 |  |
|  |  | ENST00000427099 |  |
| TREML4 | ENSG00000188056 | ENST00000400518 | (T)6 |
|  |  | ENST00000409945 |  |
| TRO | ENSG00000067445 | ENST00000628285 |  |
| TXNRD2 | ENSG00000184470 | ENST00000525407 |  |
| USP40 | ENSG00000085982 | ENST00000575984 |  |
| WAC | ENSG00000095787 | ENST00000421016 | (G)6 |
| XRRA1 | ENSG00000166435 | ENST00000580259 |  |
| ZC3H7A | ENSG00000122299 | ENST00000583566 | (A)6 |
| ZNF286A | ENSG00000187607 | ENST00000395894 | (G)6 |
|  |  | ENST00000585194 |  |
|  |  | ENST00000583031 |  |
|  |  | ENST00000464847 |  |
|  |  | ENST00000395893 |  |
|  |  | ENST00000528032 |  |
|  |  | ENST00000580488 |  |
|  |  | ENST00000477374 |  |
| ZNF48 | ENSG00000180035 | ENST00000455784 |  |
| ZNF521 | ENSG00000198795 | ENST00000468958 | (C)6 |
| ZNF717 | ENSG00000227124 | ENST00000347800 | (G)6 |
| SEPT5 | ENSG00000184702 | ENST00000389568 | (CGG)5 |
| ABCF1 | ENSG00000204574 | ENST00000373827 | (AGC)5 |
| ABCG1 | ENSG00000160179 | ENST00000430041 | (CT)5 |
| ALKBH8 | ENSG00000137760 | ENST00000552604 | (ATT)5 |
| ANK3 | ENSG00000151150 | ENST00000549602 | (AGC)5 |
| ARHGAP9 | ENSG00000123329 | ENST00000574428 | (GA)5 |
|  |  | ENST00000253968 |  |
|  |  | ENST00000540737 |  |
| ATPAF1 | ENSG00000123472 | ENST00000602766 | (GAG)5 |
| BARX1 | ENSG00000131668 | ENST00000546495 | (CGC)5 |
| C11orf80 | ENSG00000173715 | ENST00000546760 | (GGC)5 |
| C16orf74 | ENSG00000154102 | ENST00000552402 | (AGC)5 |
| C17orf49 | ENSG00000258315 | ENST00000439424 | (GCG)5 |
|  |  | ENST00000540794 |  |
|  |  | ENST00000538586 |  |
|  |  | ENST00000607597 |  |
| CCDC91 | ENSG00000123106 | ENST00000606471 | (CT)5 |
|  |  | ENST00000541430 |  |
| CD200 | ENSG00000091972 | ENST00000395145 | (AAG)5 |
|  |  | ENST00000318978 |  |
| CELF5 | ENSG00000161082 | ENST00000402357 | (CGC)5 |
| CLDN3 | ENSG00000165215 | ENST00000329759 | (GTCC)5 |
| CTXN1 | ENSG00000178531 | ENST00000309608 | (CCG)5 |
| FAM19A5 | ENSG00000219438 | ENST00000592811 | (GCG)5 |
| FAM43A | ENSG00000185112 | ENST00000586615 | (CCG)5 |
| FAM86B2 | ENSG00000145002 | ENST00000317991 | (TG)5 |
| FOSB | ENSG00000125740 | ENST00000393815 |  |
|  |  | ENST00000510882 |  |
| GRAMD1A | ENSG00000089351 | ENST00000595018 | (GCCCT)5 |
| GRIA2 | ENSG00000120251 | ENST00000566117 | (CG)5 |
| HDGFL1 | ENSG00000112273 | ENST00000496200 | (GC)5 |
| HNRNPUL1 | ENSG00000105323 | ENST00000470772 | (AG)5 |
| IL4R | ENSG00000077238 | ENST00000480861 | (CGG)5 |
| IMPDH1 | ENSG00000106348 | ENST00000337975 | (AGC)5 |
|  |  | ENST00000334067 |  |
|  |  | ENST00000439271 |  |
| KLHL25 | ENSG00000183655 | ENST00000532110 | (GGC)5 |
| KRTAP15-1 | ENSG00000186970 | ENST00000290208 | (CA)5 |
| MASP1 | ENSG00000127241 | ENST00000275015 | (GA)5 |
| MKNK1 | ENSG00000079277 | ENST00000641364 | (AG)5 |
| MRPL10 | ENSG00000159111 | ENST00000641740 | (TTCC)5 |
| NFKBIE | ENSG00000146232 | ENST00000611716 | (AATG)5 |
| OR6C3 | ENSG00000205329 | ENST00000331826 | (GA)5 |
|  |  | ENST00000550924 |  |
| PCSK6 | ENSG00000140479 | ENST00000498307 | (GGC)5 |
|  |  | ENST00000461497 |  |
| PFKM | ENSG00000152556 | ENST00000484063 | (GA)5 |
| PFN2 | ENSG00000070087 | ENST00000376048 | (CG)5 |
| PLOD2 | ENSG00000152952 | ENST00000611394 | (AT)5 |
| PRDM2 | ENSG00000116731 | ENST00000432186 | (GCG)5 |
|  |  | ENST00000561501 |  |
| PRR5 | ENSG00000186654 | ENST00000566762 |  |
|  |  | ENST00000425247 |  |
| RABEP2 | ENSG00000177548 | ENST00000489473 | (CAG)5 |
|  |  | ENST00000422358 |  |
| RAP1B | ENSG00000127314 | ENST00000537460 | (CAA)5 |
|  |  | ENST00000542018 |  |
|  |  | ENST00000534899 |  |
|  |  | ENST00000538877 |  |
|  |  | ENST00000547338 |  |
|  |  | ENST00000378096 |  |
|  |  | ENST00000366574 |  |
| RBFOX1 | ENSG00000078328 | ENST00000544052 | (TG)5 |
| RPL23 | ENSG00000125691 | ENST00000557076 | (GA)5 |
| RYR2 | ENSG00000198626 | ENST00000406900 | (CCG)5 |
| SCFD1 | ENSG00000092108 | ENST00000552192 | (CAG)5 |
|  |  | ENST00000368765 |  |
| SIRT1 | ENSG00000096717 | ENST00000559677 | (CA)5 |
| SLC6A15 | ENSG00000072041 | ENST00000425944 | (CTG)5 |
| SMCP | ENSG00000163206 | ENST00000377254 | (TG)5 |
| SPG21 | ENSG00000090487 | ENST00000449349 | (CCT)5 |
| TBC1D5 | ENSG00000131374 | ENST00000343544 | (AG)5 |
| THNSL2 | ENSG00000144115 | ENST00000591529 | (CGCGCCC)5 |
|  |  | ENST00000636222 |  |
|  |  | ENST00000348124 |  |
| TLE2 | ENSG00000065717 | ENST00000306085 | (TAA)5 |
| TNRC6C | ENSG00000078687 | ENST00000412507 | (CCG)5 |
| TRAF6 | ENSG00000175104 | ENST00000467847 | (AT)5 |
| TRIM56 | ENSG00000169871 | ENST00000372865 | (GGA)5 |
|  |  | ENST00000190983 |  |
|  |  | ENST00000331737 |  |
| WISP2 | ENSG00000064205 | ENST00000452305 | (GC)5 |
|  |  | ENST00000591319 |  |
| ZNF438 | ENSG00000183621 | ENST00000417235 | (GGA)5 |
|  |  | ENST00000573854 |  |
| ACP5 | ENSG00000102575 | ENST00000219054 | (AC)4 |
| ACSM2A | ENSG00000183747 | ENST00000606865 | (CT)4 |
|  |  | ENST00000617043 |  |
|  |  | ENST00000381312 |  |
| ACYP2 | ENSG00000170634 | ENST00000550422 | (AG)4 |
| ADAP1 | ENSG00000105963 | ENST00000409236 | (TG)4 |
| ADARB2 | ENSG00000185736 | ENST00000613322 | (GCG)4 |
| ADCY6 | ENSG00000174233 | ENST00000307864 | (CGGC)4 |
| AFF3 | ENSG00000144218 | ENST00000458219 | (CT)4 |
| AIFM2 | ENSG00000042286 | ENST00000478407 | (AGCGGG)4 |
|  |  | ENST00000600247 |  |
| AIG1 | ENSG00000146416 | ENST00000572639 | (TC)4 |
| AK5 | ENSG00000154027 | ENST00000358984 | (GA)4 |
| AKAP8L | ENSG00000011243 | ENST00000376911 | (CTATGG)4 |
| ANAPC11 | ENSG00000141552 | ENST00000422982 | (GC)4 |
| ANKRD30B | ENSG00000180777 | ENST00000617500 | (CT)4 |
| ANXA1 | ENSG00000135046 | ENST00000592999 | (TG)4 |
| ANXA11 | ENSG00000122359 | ENST00000396582 | (GT)4 |
| AOC3 | ENSG00000131471 | ENST00000389774 | (GTG)4 |
|  |  | ENST00000396119 |  |
| ARHGAP5 | ENSG00000100852 | ENST00000336963 | (GCG)4 |
| ARHGAP8 | ENSG00000241484 | ENST00000356099 | (GGC)4 |
|  |  | ENST00000412433 |  |
|  |  | ENST00000320767 |  |
|  |  |  |  |
|  |  | ENST00000308647 |  |
| ARL14 | ENSG00000179674 | ENST00000429899  ENST00000396761 | (AAGA)4 |
|  |  |  | (AG)4 |
| ATAD3B | ENSG00000160072 | ENST00000443672 | (GCG)4 |
| ATG4B | ENSG00000168397 | ENST00000574508 | (TC)4 |
| ATG9A | ENSG00000198925 | ENST00000527716 | (GCCGA)4 |
| AUTS2 | ENSG00000158321 | ENST00000465919 | (CAGCAC)4 |
| B9D1 | ENSG00000108641 | ENST00000434143 | (AC)4 |
| BATF2 | ENSG00000168062 | ENST00000554140 | (GA)4 |
| BCAP29 | ENSG00000075790 | ENST00000580709 | (AC)4 |
| BDH1 | ENSG00000161267 | ENST00000496384 | (CAAAA)4 |
| BEGAIN | ENSG00000183092 | ENST00000646891 | (GCG)4 |
| BLMH | ENSG00000108578 | ENST00000449085 | (GC)4 |
| BRAF | ENSG00000157764 | ENST00000602675 | (CCTCCG)4 |
|  |  | ENST00000585675 |  |
| BRD2 | ENSG00000204256 | ENST00000346736 | (CT)4 |
| C16orf74 | ENSG00000154102 | ENST00000397881 | (GCA)4 |
| C19orf25 | ENSG00000119559 | ENST00000435614 | (GT)4 |
| C19orf57 | ENSG00000132016 | ENST00000412910 | (GC)4 |
| C19orf66 | ENSG00000130813 | ENST00000638005 | (TC)4 |
| C3orf20 | ENSG00000131379 | ENST00000636442 | (CCA)4 |
|  |  | ENST00000417287 |  |
| CACNB4 | ENSG00000182389 | ENST00000345633 | (TCC)4 |
|  |  | ENST00000614447 |  |
| CAP1 | ENSG00000131236 | ENST00000369318 | (CTG)4 |
| CASP7 | ENSG00000165806 | ENST00000424464 | (GA)4 |
|  |  | ENST00000410012 |  |
|  |  | ENST00000344386 |  |
| CC2D2B | ENSG00000188649 | ENST00000389609 | (AT)4 |
|  |  | ENST00000343253 |  |
|  |  | ENST00000398163 |  |
| CCDC149 | ENSG00000181982 | ENST00000642746 | (TCA)4 |
| CCDC18 | ENSG00000122483 | ENST00000645565 | (GCG)4 |
| CCNB1IP1 | ENSG00000100814 | ENST00000382515 | (AG)4 |
| CD9 | ENSG00000010278 | ENST00000269141 | (TAA)4 |
|  |  | ENST00000644511 |  |
|  |  | ENST00000543233 |  |
| CDH2 | ENSG00000170558 | ENST00000542174 | (CCGCCG)4 |
| CDH23 | ENSG00000107736 | ENST00000619038 | (GCGAGCG)4 |
| CDH4 | ENSG00000179242 | ENST00000379751 | (GCT)4 |
| CDK2AP1 | ENSG00000111328 | ENST00000404067 | (CT)4 |
| CDRT4 | ENSG00000239704 | ENST00000402338 | (AG)4 |
| CENPB | ENSG00000125817 | ENST00000402420 | (CGG)4 |
| CENPM | ENSG00000100162 | ENST00000320493 | (CT)4 |
|  |  | ENST00000367424 |  |
|  |  | ENST00000476712 |  |
| CFHR1 | ENSG00000244414 | ENST00000367415 | (CAC)4 |
|  |  | ENST00000281282 |  |
| CFHR2 | ENSG00000080910 | ENST00000517636 |  |
|  |  | ENST00000258711 |  |
| CGNL1 | ENSG00000128849 | ENST00000262315 | (GGC)4 |
| CHCHD7 | ENSG00000170791 | ENST00000455171 | (TC)4 |
| CHST12 | ENSG00000136213 | ENST00000317063 | (CGG)4 |
| CHTF18 | ENSG00000127586 | ENST00000576601 |  |
|  |  | ENST00000629610 |  |
|  |  | ENST00000566126 |  |
| CIITA | ENSG00000179583 | ENST00000534772 | (GC)4 |
| CIZ1 | ENSG00000148337 | ENST00000361866 | (GCA)4 |
| CLK3 | ENSG00000179335 | ENST00000612273 | (GCG)4 |
| COG4 | ENSG00000103051 | ENST00000551769 | (GA)4 |
| COL6A1 | ENSG00000142156 | ENST00000321949 | (CGG)4 |
|  |  | ENST00000295728 |  |
| COQ5 | ENSG00000110871 | ENST00000453769 | (TA)4 |
| CRTC1 | ENSG00000105662 | ENST00000259003 | (GAG)4 |
| CRYBA2 | ENSG00000163499 | ENST00000400186 | (TG)4 |
|  |  | ENST00000602723 |  |
| CSHL1 | ENSG00000204414 | ENST00000635120 | (GA)4 |
| CSMD1 | ENSG00000183117 | ENST00000520002 | (CT)4 |
|  |  | ENST00000602557 |  |
|  |  | ENST00000373377 |  |
|  |  | ENST00000617556 |  |
|  |  | ENST00000396404 |  |
| CSMD2 | ENSG00000121904 | ENST00000539050 | (TG)4 |
| CYFIP1 | ENSG00000273749 | ENST00000334651 | (TC)4 |
| CYP19A1 | ENSG00000137869 | ENST00000412516 | (CA)4 |
| CYP2C8 | ENSG00000138115 | ENST00000359357 | (GT)4 |
| DHRSX | ENSG00000169084 | ENST00000341772 | (GCG)4 |
|  |  | ENST00000518197 |  |
| DNAH8 | ENSG00000124721 | ENST00000541083 | (AT)4 |
| DNER | ENSG00000187957 | ENST00000543292 | (CCG)4 |
| DOK2 | ENSG00000147443 | ENST00000425780 | (CAG)4 |
| DPY19L2 | ENSG00000177990 | ENST00000451769 | (ATA)4 |
| DRD2 | ENSG00000149295 | ENST00000457529 | (GT)4 |
| DTX2 | ENSG00000091073 | ENST00000423646 | (CCGC)4 |
|  |  | ENST00000438930 |  |
|  |  | ENST00000429179 |  |
|  |  | ENST00000454362 |  |
|  |  | ENST00000527888 |  |
|  |  | ENST00000637315 |  |
| DYDC1 | ENSG00000170788 | ENST00000457202 | (TGGC)4 |
| EED | ENSG00000074266 | ENST00000380876 | (CT)4 |
| EFHC1 | ENSG00000096093 | ENST00000406771 | (AC)4 |
| EHD1 | ENSG00000110047 | ENST00000430276 | (AG)4 |
| EIF3CL | ENSG00000205609 | ENST00000441639 | (TC)4 |
| EPB41L1 | ENSG00000088367 | ENST00000628415 | (CGC)4 |
|  |  | ENST00000460895 |  |
|  |  | ENST00000586329 |  |
|  |  | ENST00000519638 |  |
| EPHB1 | ENSG00000154928 | ENST00000540767 | (GA)4 |
| EPS8L1 | ENSG00000131037 | ENST00000393034 | (TCC)4 |
| ERLIN2 | ENSG00000147475 | ENST00000402357 | (CA)4 |
| FADS1 | ENSG00000149485 | ENST00000332947 |  |
| FAIM | ENSG00000158234 | ENST00000393600 | (CGG)4 |
| FAM19A5 | ENSG00000219438 | ENST00000519378 | (GC)4 |
| FAM43B | ENSG00000183114 | ENST00000518752 | (CGC)4 |
| FAM50A | ENSG00000071859 | ENST00000536936 | (CCGCCG)4 |
| FBXL8 | ENSG00000135722 | ENST00000541252 | (TC)4 |
| FBXW11 | ENSG00000072803 | ENST00000259989 |  |
| FERMT1 | ENSG00000101311 | ENST00000510911 | (AAG)4 |
| FERMT3 | ENSG00000149781 | ENST00000627578 | (AGA)4 |
| FGFBP2 | ENSG00000137441 |  | (TC)4 |
| FGFR4 | ENSG00000160867 | ENST00000550424 | (AGG)4 |
| FHL1 | ENSG00000022267 | ENST00000592811 | (GCC)4 |
|  |  |  |  |
| FMNL3 | ENSG00000161791 | ENST00000586615 | (TA)4 |
| FOSB | ENSG00000125740 | ENST00000339140 | (GT)4 |
|  |  |  | (TG)4 |
|  |  | ENST00000504750  ENST00000443774 | (GT)4 |
|  |  |  | (TG)4 |
| FOXR2 | ENSG00000189299 | ENST00000460709 | (TC)4 |
| FRG2 | ENSG00000205097 | ENST00000405801 | (GA)4 |
| FRG2B | ENSG00000225899 | ENST00000265342 |  |
| FRMD4B | ENSG00000114541 | ENST00000358752 | (GC)4 |
| FSCN1 | ENSG00000075618 | ENST00000437025 | (CG)4 |
| FSTL4 | ENSG00000053108 | ENST00000519621 | (CGC)4 |
| FUT4 | ENSG00000196371 | ENST00000636573 | (GA)4 |
| GABRA1 | ENSG00000022355 | ENST00000639975 | (GAGC)4 |
|  |  | ENST00000639683 |  |
|  |  | ENST00000640985 | (ATT)4 |
| GABRG2 | ENSG00000113327 | ENST00000428024 | (AC)4 |
|  |  | ENST00000621295 |  |
|  |  | ENST00000296875 |  |
| GBA | ENSG00000177628 | ENST00000498181 | (GT)4 |
| GDF9 | ENSG00000164404 | ENST00000358647 | (GA)4 |
|  |  | ENST00000322315 |  |
| GIMAP5 | ENSG00000196329 | ENST00000553333 | (CT)4 |
|  |  | ENST00000371100 |  |
| GIPC3 | ENSG00000179855 | ENST00000526619 | (GCG)4 |
| GMFB | ENSG00000197045 | ENST00000270590 | (TG)4 |
| GNAS | ENSG00000087460 | ENST00000411896 | (TAA)4 |
| GOLGA8F | ENSG00000153684 | ENST00000398016 | (CT)4 |
| GPR32 | ENSG00000142511 | ENST00000589536 | (AAT)4 |
| GRAMD1A | ENSG00000089351 | ENST00000412805 | (CTGCC)4 |
| GRIP1 | ENSG00000155974 | ENST00000472590 | (CTG)4 |
| GRN | ENSG00000030582 | ENST00000576444 | (TGG)4 |
| HADHB | ENSG00000138029 | ENST00000614378 | (GT)4 |
| HHEX | ENSG00000152804 | ENST00000359426 | (TC)4 |
| HIC1 | ENSG00000177374 | ENST00000558176 | (GT)4 |
| HIST1H3G | ENSG00000273983 | ENST00000559099 | (TA)4 |
| HK1 | ENSG00000156515 | ENST00000460160 | (GAG)4 |
| HMG20A | ENSG00000140382 | ENST00000264735 | (TC)4 |
|  |  | ENST00000446825 | (GGT)4 |
| HOXB3 | ENSG00000120093 | ENST00000549334 | (AT)4 |
| HRASLS | ENSG00000127252 | ENST00000372425 | (CGGG)4 |
| HS1BP3 | ENSG00000118960 | ENST00000583037 | (GGA)4 |
| HSP90B1 | ENSG00000166598 | ENST00000372432 | (AG)4 |
| HYI | ENSG00000178922 | ENST00000412356 | (GCCCGCC)4 |
|  |  | ENST00000579687 |  |
|  |  | ENST00000578313 |  |
| ICAM2 | ENSG00000108622 | ENST00000428740 | (AG)4 |
|  |  | ENST00000326427 |  |
|  |  | ENST00000164247 |  |
| IMP4 | ENSG00000136718 | ENST00000295082 | (GC)4 |
| ITM2C | ENSG00000135916 | ENST00000615244 | (AG)4 |
| KCNAB2 | ENSG00000069424 | ENST00000587698 | (CT)4 |
| KCNF1 | ENSG00000162975 | ENST00000354386 | (GCG)4 |
| KCNJ16 | ENSG00000153822 | ENST00000328879 | (AG)4 |
|  |  | ENST00000252999 |  |
| KIAA0586 | ENSG00000100578 | ENST00000443314 | (TAAA)4 |
| KLHL22 | ENSG00000099910 | ENST00000360090 | (CGCCTCC)4 |
| LAMA5 | ENSG00000130702 | ENST00000373755 | (CG)4 |
| LANCL1 | ENSG00000115365 | ENST00000373754 | (CGC)4 |
| LCE1B | ENSG00000196734 | ENST00000497245 | (CT)4 |
| LHX6 | ENSG00000106852 | ENST00000541170 | (GCC)4 |
|  |  | ENST00000602421 |  |
| LRCH4 | ENSG00000077454 | ENST00000376667 | (GCA)4 |
| LUC7L2 | ENSG00000146963 | ENST00000589130 | (GC)4 |
| MACF1 | ENSG00000127603 | ENST00000641862 | (GCC)4 |
| MAD2L2 | ENSG00000116670 | ENST00000641072 | (CGC)4 |
| MAP4K1 | ENSG00000104814 | ENST00000641873 | (GC)4 |
| MAPK10 | ENSG00000109339 | ENST00000641116 | (GA)4 |
|  |  | ENST00000641675 |  |
|  |  | ENST00000373759 |  |
|  |  | ENST00000616552 |  |
|  |  | ENST00000419835 |  |
| MAPK13 | ENSG00000156711 | ENST00000466623 | (GCT)4 |
| MCM3 | ENSG00000112118 | ENST00000507601 | (GAA)4 |
|  |  | ENST00000407029 |  |
| MECOM | ENSG00000085276 | ENST00000402630 | (TC)4 |
| MFAP3L | ENSG00000198948 | ENST00000630269 | (AG)4 |
| MKL1 | ENSG00000196588 | ENST00000491222 | (TC)4 |
|  |  | ENST00000399784 |  |
| MLLT3 | ENSG00000171843 | ENST00000375750 | (GCC)4 |
| MPZ | ENSG00000158887 | ENST00000404297 | (TGC)4 |
| MRAP | ENSG00000170262 | ENST00000578017 | (AG)4 |
| MSH5 | ENSG00000204410 | ENST00000475276 | (GC)4 |
| MTIF2 | ENSG00000085760 | ENST00000609507 | (TTAT)4 |
| MYH2 | ENSG00000125414 | ENST00000421929 | (GTT)4 |
| NDUFB2 | ENSG00000090266 | ENST00000448584 | (CAAA)4 |
| NFATC2 | ENSG00000101096 | ENST00000537859 | (AG)4 |
| NFE2L2 | ENSG00000116044 | ENST00000427260 | (CG)4 |
| NLRP2 | ENSG00000022556 | ENST00000449394 | (GA)4 |
|  |  | ENST00000592782 |  |
|  |  | ENST00000594670 |  |
| NLRX1 | ENSG00000160703 | ENST00000510170 | (CA)4 |
| NMT1 | ENSG00000136448 | ENST00000284548 | (GC)4 |
| NPAS1 | ENSG00000130751 | ENST00000422127 |  |
| NR3C1 | ENSG00000113580 | ENST00000570156 | (GA)4 |
| OBSCN | ENSG00000154358 | ENST00000597836 | (AG)4 |
|  |  | ENST00000382349 |  |
|  |  | ENST00000444197 |  |
| OCEL1 | ENSG00000099330 | ENST00000641693 | (CG)4 |
| ONECUT3 | ENSG00000205922 | ENST00000358120 | (GC)4 |
| OR10C1 | ENSG00000206474 | ENST00000641220 | (AG)4 |
| OR2A5 | ENSG00000221836 | ENST00000641528 | (CA)4 |
| OR2L13 | ENSG00000196071 | ENST00000633214 |  |
| OR2T33 | ENSG00000177212 | ENST00000372824 | (AT)4 |
| OR52K1 | ENSG00000196778 | ENST00000372819 | (TA)4 |
| OVOL3 | ENSG00000105261 | ENST00000374968 | (TG)4 |
| PABPC1L | ENSG00000101104 | ENST00000402038 | (CT)4 |
|  |  | ENST00000616448 |  |
| PAGE2 | ENSG00000234068 | ENST00000621505 | (GA)4 |
| PARG | ENSG00000227345 | ENST00000532602 | (TC)4 |
|  |  | ENST00000378122 |  |
| PCDH11Y | ENSG00000099715 | ENST00000623915 | (AG)4 |
| PCDHA9 | ENSG00000204961 | ENST00000538749 | (CT)4 |
|  |  | ENST00000412480 |  |
| PCDHB5 | ENSG00000113209 | ENST00000340635 | (AG)4 |
| PDE2A | ENSG00000186642 | ENST00000522278 |  |
| PDE4B | ENSG00000184588 | ENST00000482108 | (GCA)4 |
| PDE4D | ENSG00000113448 | ENST00000613043 | (GCG)4 |
| PEBP4 | ENSG00000134020 | ENST00000617526 | (CG)4 |
| PEG10 | ENSG00000242265 | ENST00000393524 | (CCT)4 |
|  |  | ENST00000529142 |  |
|  |  | ENST00000430800 |  |
| PHLPP2 | ENSG00000040199 | ENST00000489223 | (AG)4 |
| PI4KB | ENSG00000143393 | ENST00000409527 | (GA)4 |
|  |  | ENST00000477593 |  |
|  |  | ENST00000576722 |  |
| PIGQ | ENSG00000007541 | ENST00000575895 | (TC)4 |
| PIK3CB | ENSG00000051382 | ENST00000576010 | (GGC)4 |
| PITPNA | ENSG00000174238 | ENST00000573231 | (AC)4 |
|  |  | ENST00000262304 |  |
|  |  | ENST00000423118 |  |
|  |  | ENST00000292614 |  |
| PKD1 | ENSG00000008710 | ENST00000440068 | (CG)4 |
|  |  | ENST00000055335 |  |
| POLR2J | ENSG00000005075 | ENST00000591228 | (GCG)4 |
| PPIH | ENSG00000171960 | ENST00000487082 | (AGG)4 |
| PPP1R3F | ENSG00000049769 | ENST00000415329 | (CGC)4 |
| PPY | ENSG00000108849 | ENST00000441887 | (GT)4 |
| PRKRA | ENSG00000180228 | ENST00000403581 | (CT)4 |
| PROCA1 | ENSG00000167525 | ENST00000389413 | (GAG)4 |
| PRPSAP2 | ENSG00000141127 | ENST00000409483 | (ATG)4 |
| PRR5 | ENSG00000186654 | ENST00000395595 | (TAA)4 |
| PTPRN2 | ENSG00000155093 | ENST00000493985 | (GC)4 |
|  |  | ENST00000469835 |  |
| RABL2B | ENSG00000079974 | ENST00000493032 | (CT)4 |
| RAD54L | ENSG00000085999 | ENST00000374761 | (AG)4 |
|  |  | ENST00000521947 | (GA)4 |
|  |  | ENST00000545678 | (AG)4 |
| RAP1GAP | ENSG00000076864 | ENST00000534478 | (GC)4 |
| RBM12B | ENSG00000183808 | ENST00000368805 | (GT)4 |
| RBM7 | ENSG00000076053 | ENST00000571750 | (CGG)4 |
| RCOR3 | ENSG00000117625 | ENST00000572990 | (GGC)4 |
| REV3L | ENSG00000009413 | ENST00000576652 | (CAT)4 |
| RFWD3 | ENSG00000168411 | ENST00000575113 | (CT)4 |
|  |  | ENST00000609447 |  |
|  |  | ENST00000477776 |  |
|  |  | ENST00000609944 |  |
| RNF146 | ENSG00000118518 | ENST00000529368 |  |
|  |  | ENST00000525801 |  |
|  |  | ENST00000534093 |  |
| RNH1 | ENSG00000023191 | ENST00000490373 | (GCG)4 |
| ROM1 | ENSG00000149489 | ENST00000519209 | (GA)4 |
|  |  | ENST00000380927 | (AG)4 |
| RUSC1 | ENSG00000160753 | ENST00000633494 | (CAC)4 |
| SCIN | ENSG00000006747 | ENST00000355814 | (TGA)4 |
| SECISBP2L | ENSG00000138593 | ENST00000556955 | (TC)4 |
| SEMA4A | ENSG00000196189 | ENST00000361972 | (AG)4 |
| SERPINA1 | ENSG00000197249 | ENST00000367857 | (TGC)4 |
|  |  | ENST00000429490 |  |
| SFMBT2 | ENSG00000198879 | ENST00000361781 | (TG)4 |
| SGK1 | ENSG00000118515 | ENST00000361543 | (GT)4 |
| SGMS1 | ENSG00000198964 | ENST00000619438 | (AG)4 |
|  |  | ENST00000452765 |  |
|  |  | ENST00000511747 |  |
|  |  | ENST00000612611 |  |
| SH3BP2 | ENSG00000087266 | ENST00000233535 | (GCG)4 |
|  |  | ENST00000465289 | (GGC)4 |
| SHISA5 | ENSG00000164054 | ENST00000421082 | (CAC)4 |
| SLC30A3 | ENSG00000115194 | ENST00000482950 | (CT)4 |
| SLC35A3 | ENSG00000117620 | ENST00000642639 | (GGA)4 |
| SLC35E1 | ENSG00000127526 | ENST00000636347 | (AT)4 |
| SLC4A2 | ENSG00000164889 | ENST00000636092 | (CT)4 |
| SLC6A1 | ENSG00000157103 | ENST00000637195 | (TC)4 |
| SLC9A6 | ENSG00000198689 | ENST00000637234 | (CGG)4 |
|  |  | ENST00000637581 |  |
|  |  | ENST00000370701 |  |
|  |  | ENST00000627534 |  |
|  |  | ENST00000630721 |  |
|  |  | ENST00000325089 |  |
|  |  | ENST00000567248 |  |
|  |  | ENST00000564644 |  |
| SLITRK5 | ENSG00000165300 | ENST00000563793 | (TG)4 |
| SLX1B | ENSG00000181625 | ENST00000321765 | (GA)4 |
| SNUPN | ENSG00000169371 | ENST00000435853 | (CAA)4 |
|  |  | ENST00000638712 |  |
| SPAG5 | ENSG00000076382 | ENST00000374884 | (CG)4 |
| SPEG | ENSG00000072195 | ENST00000309836 | (GT)4 |
| SPIN3 | ENSG00000204271 | ENST00000453447 | (GA)4 |
| SPIN4 | ENSG00000186767 | ENST00000497844 | (TA)4 |
| SPIRE1 | ENSG00000134278 | ENST00000521232 | (GAG)4 |
|  |  | ENST00000337502 |  |
|  |  | ENST00000616191 |  |
| SQLE | ENSG00000104549 | ENST00000596480 | (GCC)4 |
| SSX2 | ENSG00000241476 | ENST00000376893 | (CT)4 |
| SSX2B | ENSG00000268447 | ENST00000562759 |  |
|  |  | ENST00000467087 |  |
| SSX3 | ENSG00000165584 | ENST00000467011 |  |
| ST20 | ENSG00000180953 | ENST00000465503 | (TTAT)4 |
| STIM2 | ENSG00000109689 | ENST00000495504 | (GCG)4 |
|  |  | ENST00000350842 |  |
|  |  | ENST00000606202 |  |
| STXBP5L | ENSG00000145087 | ENST00000612220 | (AG)4 |
| SULT1A1 | ENSG00000196502 | ENST00000485989 | (GC)4 |
| TAB2 | ENSG00000055208 | ENST00000313288 | (TC)4 |
| TACC3 | ENSG00000013810 | ENST00000617535 | (GGC)4 |
|  |  | ENST00000536979 |  |
|  |  | ENST00000425944 |  |
|  |  | ENST00000335351 |  |
| TAOK3 | ENSG00000135090 | ENST00000421427 | (GT)4 |
| TBC1D5 | ENSG00000131374 | ENST00000453013 | (AGG)4 |
| TCFL5 | ENSG00000101190 | ENST00000399133 | (CGCCTCC)4 |
| TFPI | ENSG00000003436 | ENST00000515662 | (AG)4 |
|  |  | ENST00000559191 |  |
| THAP7 | ENSG00000184436 | ENST00000513002 | (GC)4 |
| THUMPD3 | ENSG00000134077 | ENST00000613307 | (TC)4 |
| TLE3 | ENSG00000140332 | ENST00000521452 | (CGC)4 |
| TMCO6 | ENSG00000113119 | ENST00000534025 | (GA)4 |
| TMEM132B | ENSG00000139364 | ENST00000549919 | (TC)4 |
| TMEM200B | ENSG00000253304 | ENST00000447595 | (CG)4 |
| TMEM9B | ENSG00000175348 | ENST00000581411 | (GCCTGA)4 |
| TMTC2 | ENSG00000179104 | ENST00000548465 | (CAA)4 |
| TOR3A | ENSG00000186283 | ENST00000490290 | (GA)4 |
| TP53I13 | ENSG00000167543 | ENST00000400103 | (AG)4 |
| TPCN1 | ENSG00000186815 | ENST00000558569 | (TC)4 |
| TPRA1 | ENSG00000163870 | ENST00000450533 | (CT)4 |
| TPTE2 | ENSG00000132958 | ENST00000438055 | (GA)4 |
| TSHZ3 | ENSG00000121297 | ENST00000355666 | (GGC)4 |
| TTC3 | ENSG00000182670 | ENST00000399010 | (GCT)4 |
|  |  | ENST00000520977 |  |
|  |  | ENST00000360802 |  |
|  |  | ENST00000412543 |  |
| TULP2 | ENSG00000104804 | ENST00000535859 | (TC)4 |
| UBAP2 | ENSG00000137073 | ENST00000602959 | (GCC)4 |
|  |  | ENST00000377080 |  |
| UBC | ENSG00000150991 | ENST00000377078 | (GC)4 |
| UBE2E3 | ENSG00000170035 | ENST00000338981 | (CG)4 |
| USP11 | ENSG00000102226 | ENST00000541562 | (AC)4 |
|  |  | ENST00000420567 |  |
| USP9Y | ENSG00000114374 |  | (TA)4 |
| VARS2 | ENSG00000137411 | ENST00000341702 | (GC)4 |
| VAT1 | ENSG00000108828 | ENST00000495558  ENST00000309182 | (TC)4 |
|  |  |  | (CCT)4 |
| VN1R2 | ENSG00000196131 | ENST00000418841 | (TC)4 |
| VWA1 | ENSG00000179403 | ENST00000547944 |  |
| WDR81 | ENSG00000167716 | ENST00000315764 | (GAG)4 |
|  |  | ENST00000375602 | (TG)4 |
| WDR90 | ENSG00000161996 | ENST00000375600 | (AC)4 |
|  |  | ENST00000375616 |  |
| XAGE1A | ENSG00000204379 | ENST00000375613 |  |
|  |  | ENST00000518075 |  |
| XAGE1B | ENSG00000204382 | ENST00000443240 |  |
|  |  | ENST00000584307 |  |
|  |  | ENST00000451498 |  |
| XPO1 | ENSG00000082898 | ENST00000319119 | (CTC)4 |
| YES1 | ENSG00000176105 | ENST00000636289 | (GC)4 |
| ZAP70 | ENSG00000115085 | ENST00000513258 | (TG)4 |
| ZBTB34 | ENSG00000177125 | ENST00000514251 | (CGG)4 |
| ZBTB38 | ENSG00000177311 | ENST00000504673 | (TC)4 |
|  |  | ENST00000300101 |  |
|  |  | ENST00000374142 |  |
|  |  | ENST00000522520 |  |
| ZBTB39 | ENSG00000166860 | ENST00000521895 | (GCG)4 |
| ZDHHC18 | ENSG00000204160 | ENST00000521287 | (GC)4 |
| ZFAND1 | ENSG00000104231 | ENST00000517588 | (GA)4 |
|  |  | ENST00000520604 |  |
|  |  | ENST00000523361 |  |
|  |  | ENST00000521742 |  |
|  |  | ENST00000518419 |  |
|  |  | ENST00000520076 |  |
|  |  | ENST00000591213 |  |
|  |  | ENST00000359467 |  |
|  |  | ENST00000598288 |  |
| ZNF266 | ENSG00000174652 | ENST00000398612 | (TG)4 |
| ZNF33B | ENSG00000196693 | ENST00000518414 | (AG)4 |
| ZNF43 | ENSG00000198521 | ENST00000466975 | (GA)4 |
| ZNF596 | ENSG00000172748 | ENST00000397121 | (CG)4 |
|  |  | ENST00000268154 |  |
| ZNF638 | ENSG00000075292 | ENST00000525288 |  |
| ZNF676 | ENSG00000196109 | ENST00000450231 | (GT)4 |
| ZNF710 | ENSG00000140548 | ENST00000538082 | (GGCCC)4 |
| ZNF790 | ENSG00000197863 | ENST00000510792 | (TG)4 |
| ZPBP | ENSG00000042813 |  | (GCT)4 |
| ZSCAN10 | ENSG00000130182 | ENST00000311269 | (GGA)4 |
| MARCH6 | ENSG00000145495 | ENST00000583600  ENST00000449239 | (TA)3 |
|  |  |  | (GA)3 |
| MARCH10 | ENSG00000173838 | ENST00000412544 | (GCCC)3 |
|  |  | ENST00000493445 |  |
| SEPT2 | ENSG00000168385 | ENST00000461295 | (ATAC)3 |
| SEPT5 | ENSG00000184702 | ENST00000591934 | (GACCCC)3 |
| SEPT10 | ENSG00000186522 | ENST00000591020 | (TG)3 |
|  |  | ENST00000586521 |  |
| SEPT9 | ENSG00000184640 | ENST00000590938 | (AT)3 |
|  |  | ENST00000586433 | (GT)3 |
|  |  | ENST00000381278 | (AC)3 |
|  |  | ENST00000561452 | (CAC)3 |
|  |  | ENST00000542650 | (TC)3 |
| A4GALT | ENSG00000128274 | ENST00000560362 | (GT)3 |
| AAGAB | ENSG00000103591 | ENST00000536513 | (CA)3 |
|  |  | ENST00000588877 |  |
|  |  | ENST00000593153 |  |
| ABCA4 | ENSG00000198691 | ENST00000592568 | (CT)3 |
| ABCA5 | ENSG00000154265 | ENST00000524850 | (TTTG)3 |
|  |  |  | (GT)3 |
|  |  | ENST00000265723 | (TTTG)3 |
| ABCA7 | ENSG00000064687 | ENST00000540971  ENST00000536976 | (GC)3 |
|  |  |  | (CTG)3 |
| ABCB4 | ENSG00000005471 | ENST00000541424 | (CGGGGG)3 |
| ABCB9 | ENSG00000150967 | ENST00000372515 | (GT)3 |
|  |  | ENST00000326684 | (GA)3 |
|  |  | ENST00000468958 |  |
| ABCC10 | ENSG00000124574 | ENST00000587994 | (CTG)3 |
| ABCC9 | ENSG00000069431 | ENST00000552965 | (GAAA)3 |
| ABCF1 | ENSG00000204574 | ENST00000375897 | (GAT)3 |
| ACAA2 | ENSG00000167315 | ENST00000375888 | (GC)3 |
| ACAD10 | ENSG00000111271 | ENST00000582627 | (TA)3 |
| ACBD5 | ENSG00000107897 | ENST00000301452 | (CCT)3 |
|  |  | ENST00000534206 |  |
| ACE | ENSG00000159640 | ENST00000241069 | (GC)3 |
| ACER1 | ENSG00000167769 | ENST00000419336 | (TC)3 |
| ACER3 | ENSG00000078124 | ENST00000302913 | (GCG)3 |
| ACHE | ENSG00000087085 | ENST00000411582 | (CGGCC)3 |
|  |  | ENST00000608083 |  |
|  |  | ENST00000590832 |  |
|  |  | ENST00000591319 |  |
| ACOT7 | ENSG00000097021 | ENST00000546114 | (CCG)3 |
| ACP5 | ENSG00000102575 | ENST00000560817 | (TC)3 |
|  |  | ENST00000515030 | (AC)3 |
| ACRBP | ENSG00000111644 | ENST00000379246 | (TC)3 |
| ACSBG1 | ENSG00000103740 | ENST00000573854 | (AT)3 |
| ACSL1 | ENSG00000151726 | ENST00000252071 | (GC)3 |
| ACSL6 | ENSG00000164398 | ENST00000477367 | (CG)3 |
| ACSM2A | ENSG00000183747 | ENST00000636209 | (CT)3 |
| ACTR3C | ENSG00000106526 | ENST00000449296 | (AG)3 |
|  |  |  |  |
| ADAMTSL1 | ENSG00000178031 | ENST00000389863 | (GCC)3 |
| ADAP1 | ENSG00000105963 | ENST00000348831  ENST00000437626 | (GT)3 |
|  |  |  | (TC)3 |
| ADARB1 | ENSG00000197381 | ENST00000449478 | (CT)3 |
|  |  | ENST00000515681 |  |
|  |  | ENST00000466617 |  |
|  |  | ENST00000483566 |  |
| ADCY2 | ENSG00000078295 | ENST00000437033 | (TC)3 |
| ADCY5 | ENSG00000173175 | ENST00000209665 | (CAG)3 |
|  |  | ENST00000621696 |  |
| ADH7 | ENSG00000196344 | ENST00000645081 | (TA)3 |
|  |  | ENST00000534467 |  |
| ADNP | ENSG00000101126 | ENST00000559951 | (ATG)3 |
|  |  | ENST00000612156 | (AG)3 |
|  |  | ENST00000375418 | (ATG)3 |
| ADNP2 | ENSG00000101544 | ENST00000449162 | (TC)3 |
| ADPRHL1 | ENSG00000153531 | ENST00000221561 | (GAG)3 |
|  |  |  |  |
| AEBP1 | ENSG00000106624 | ENST00000441400 | (CT)3 |
| AES | ENSG00000104964 | ENST00000581191  ENST00000443782 | (CAC)3 |
|  |  |  | (CA)3 |
| AFF3 | ENSG00000144218 | ENST00000374094 | (CGC)3 |
| AGAP5 | ENSG00000172650 | ENST00000375065 | (GT)3 |
|  |  | ENST00000456594 |  |
|  |  |  |  |
| AGER | ENSG00000204305 | ENST00000395496 | (TGG)3 |
| AGFG1 | ENSG00000173744 | ENST00000457068  ENST00000404754 | (TG)3 |
|  |  |  | (AC)3 |
| AGPAT1 | ENSG00000204310 | ENST00000474935 | (CA)3 |
| AGPAT3 | ENSG00000160216 | ENST00000376637 | (AG)3 |
| AGTR1 | ENSG00000144891 | ENST00000265602 | (CG)3 |
|  |  | ENST00000488690 | (TA)3 |
| AGTRAP | ENSG00000177674 | ENST00000534469 | (CGGGG)3 |
| AHI1 | ENSG00000135541 | ENST00000458219 | (CA)3 |
|  |  | ENST00000611749 |  |
|  |  | ENST00000576591 | (TG)3 |
| AIG1 | ENSG00000146416 | ENST00000560256 |  |
| AK3 | ENSG00000147853 | ENST00000560340 | (GT)3 |
| AKAP1 | ENSG00000121057 | ENST00000434623 | (TC)3 |
| AKAP13 | ENSG00000170776 | ENST00000359579 | (AC)3 |
|  |  | ENST00000380554 | (ACC)3 |
| AKAP2 | ENSG00000241978 | ENST00000533295 | (GC)3 |
| AKR1B10 | ENSG00000198074 | ENST00000420396 | (AG)3 |
| AKR1C3 | ENSG00000196139 | ENST00000448137 | (AGC)3 |
| AKR1E2 | ENSG00000165568 | ENST00000573368 | (AG)3 |
| AKR7L | ENSG00000211454 | ENST00000435638 | (GC)3 |
| ALAD | ENSG00000148218 |  | (AG)3 |
| ALDH3A1 | ENSG00000108602 | ENST00000445626 | (AGC)3 |
| ALDOC | ENSG00000109107 | ENST00000443765  ENST00000239891 | (TC)3 |
|  |  |  | (CT)3 |
| ALG3 | ENSG00000214160 | ENST00000389568 | (GA)3 |
| ALG5 | ENSG00000120697 |  | (CG)3 |
|  |  | ENST00000568263 |  |
| ALKBH8 | ENSG00000137760 | ENST00000563664  ENST00000372057 | (TC)3 |
|  |  |  | (GCCT)3 |
| AMDHD2 | ENSG00000162066 | ENST00000537562 | (TG)3 |
| AMFR | ENSG00000159461 | ENST00000541931 | (GAG)3 |
| AMMECR1 | ENSG00000101935 | ENST00000542781 | (GCC)3 |
| AMN1 | ENSG00000151743 | ENST00000514516 | (GTG)3 |
|  |  | ENST00000510560 | (GT)3 |
|  |  |  |  |
| AMOTL2 | ENSG00000114019 | ENST00000515172 | (CT)3 |
|  |  | ENST00000511751 | (CG)3 |
|  |  |  | (AG)3 |
|  |  | ENST00000397990  ENST00000555079 | (CG)3 |
|  |  |  | (AG)3 |
| ANAPC13 | ENSG00000129055 | ENST00000371129 | (TC)3 |
| ANG | ENSG00000214274 | ENST00000503271 | (CA)3 |
| ANGEL1 | ENSG00000013523 | ENST00000503423 | (AG)3 |
| ANGPTL3 | ENSG00000132855 | ENST00000506722 | (AT)3 |
| ANK2 | ENSG00000145362 | ENST00000280772 | (AG)3 |
|  |  | ENST00000373827 |  |
|  |  | ENST00000297183 |  |
| ANK3 | ENSG00000151150 | ENST00000528722 | (GT)3 |
|  |  | ENST00000358990 | (GA)3 |
| ANKHD1 | ENSG00000131503 | ENST00000406423 | (GGT)3 |
| ANKRD42 | ENSG00000137494 | ENST00000609454 | (CA)3 |
| ANKRD46 | ENSG00000186106 | ENST00000446014 | (GCT)3 |
| ANKRD54 | ENSG00000100124 | ENST00000453432 | (TC)3 |
|  |  | ENST00000416565 | (CT)3 |
| ANKS3 | ENSG00000168096 | ENST00000436226 | (GT)3 |
| ANKZF1 | ENSG00000163516 | ENST00000546188 | (AG)3 |
|  |  | ENST00000438331 | (CAGG)3 |
|  |  | ENST00000265447 |  |
| ANO2 | ENSG00000047617 | ENST00000437799 | (GC)3 |
| ANXA11 | ENSG00000122359 | ENST00000559818 | (TG)3 |
|  |  | ENST00000503570 | (ATG)3 |
|  |  | ENST00000372921 | (GTG)3 |
| ANXA2 | ENSG00000182718 | ENST00000372919 | (GA)3 |
| ANXA3 | ENSG00000138772 | ENST00000569748 | (CAC)3 |
| ANXA7 | ENSG00000138279 | ENST00000567583 | (GCT)3 |
|  |  | ENST00000556843 |  |
| AP1G1 | ENSG00000166747 | ENST00000444408 | (TA)3 |
|  |  | ENST00000372745 | (CG)3 |
| AP1G2 | ENSG00000213983 | ENST00000592316 | (CGCTGC)3 |
| AP1S3 | ENSG00000152056 | ENST00000463812 | (TC)3 |
| AP3M1 | ENSG00000185009 | ENST00000402255 | (CT)3 |
| APLP1 | ENSG00000105290 | ENST00000249116 | (GAAA)3 |
| APOA2 | ENSG00000158874 | ENST00000618553 | (TG)3 |
| APOBEC3A | ENSG00000128383 | ENST00000590360 | (GA)3 |
|  |  | ENST00000397278 | (AT)3 |
|  |  | ENST00000422706 |  |
| APOC2 | ENSG00000234906 |  | (GAT)3 |
| APOL1 | ENSG00000100342 | ENST00000426053 | (CA)3 |
|  |  | ENST00000427990 |  |
|  |  |  | (AGG)3 |
|  |  | ENST00000397279 | (CA)3 |
|  |  | ENST00000433768 |  |
|  |  |  | (AGG)3 |
|  |  | ENST00000424878 | (CA)3 |
|  |  | ENST00000352371  ENST00000447660 |  |
|  |  |  | (AGG)3 |
| APOL3 | ENSG00000128284 | ENST00000290277 | (TC)3 |
| APOL4 | ENSG00000100336 | ENST00000377045 | (TA)3 |
| AQP7 | ENSG00000165269 | ENST00000377039 | (AG)3 |
| ARAF | ENSG00000078061 | ENST00000393605 | (CGG)3 |
|  |  |  |  |
|  |  | ENST00000504448 |  |
| ARAP1 | ENSG00000186635 | ENST00000550188  ENST00000565905 | (TG)3 |
|  |  |  |  |
| ARAP3 | ENSG00000120318 | ENST00000543522 | (AG)3 |
| ARFGAP1 | ENSG00000101199 | ENST00000525234 | (AT)3 |
| ARHGAP11A | ENSG00000198826 | ENST00000396582 | (CA)3 |
|  |  |  |  |
| ARHGAP32 | ENSG00000134909 | ENST00000433497 | (GCG)3 |
| ARHGAP5 | ENSG00000100852 | ENST00000380736  ENST00000337414 | (GC)3 |
|  |  |  | (GGA)3 |
|  |  | ENST00000380718 | (GAG)3 |
| ARHGAP6 | ENSG00000047648 | ENST00000389774 | (GC)3 |
|  |  | ENST00000396119 | (AGG)3 |
|  |  | ENST00000336963 |  |
| ARHGAP8 | ENSG00000241484 | ENST00000356099 | (AG)3 |
|  |  | ENST00000412433 |  |
|  |  | ENST00000378378 |  |
|  |  | ENST00000326016 |  |
|  |  | ENST00000611048 |  |
| ARHGEF16 | ENSG00000130762 | ENST00000428230 | (CAGGA)3 |
| ARHGEF4 | ENSG00000136002 | ENST00000525839 | (CG)3 |
|  |  | ENST00000637178 |  |
|  |  | ENST00000635967 |  |
|  |  | ENST00000374152 |  |
| ARHGEF9 | ENSG00000131089 | ENST00000615191 | (AC)3 |
|  |  | ENST00000346085 | (TC)3 |
| ARID1A | ENSG00000117713 |  | (TA)3 |
|  |  | ENST00000366603 |  |
| ARID1B | ENSG00000049618 | ENST00000536227  ENST00000428992 | (GC)3 |
|  |  |  | (CAC)3 |
| ARID4B | ENSG00000054267 | ENST00000518908 | (TG)3 |
| ARL1 | ENSG00000120805 | ENST00000541725 | (CT)3 |
| ARL5A | ENSG00000162980 | ENST00000461600 | (GCG)3 |
| ARMC1 | ENSG00000104442 | ENST00000446447 | (TC)3 |
| ARMC6 | ENSG00000105676 | ENST00000401424 | (CT)3 |
| ARMC8 | ENSG00000114098 | ENST00000529388 | (GA)3 |
| ARMC9 | ENSG00000135931 | ENST00000443222 | (TC)3 |
| ARNTL | ENSG00000133794 | ENST00000414376 | (CT)3 |
|  |  | ENST00000369880 | (GCGGGC)3 |
| ARPC1B | ENSG00000130429 |  | (GC)3 |
|  |  | ENST00000615257 |  |
| AS3MT | ENSG00000214435 | ENST00000357668 |  |
|  |  |  |  |
|  |  | ENST00000428113  ENST00000317126 |  |
|  |  |  |  |
| ASAP1 | ENSG00000153317 | ENST00000342331 | (GA)3 |
| ASB4 | ENSG00000005981 | ENST00000355035 | (TC)3 |
| ASCC1 | ENSG00000138303 | ENST00000254850 | (TGA)3 |
| ASCL4 | ENSG00000187855 | ENST00000343823 | (TCT)3 |
| ASGR2 | ENSG00000161944 | ENST00000526096 | (CAGCTC)3 |
|  |  | ENST00000288520 |  |
| ASH2L | ENSG00000129691 | ENST00000361477 | (CA)3 |
| ASRGL1 | ENSG00000162174 | ENST00000404843 | (CGG)3 |
| ASTN2 | ENSG00000148219 | ENST00000521903 | (AG)3 |
|  |  | ENST00000548446 | (CAG)3 |
| ASXL2 | ENSG00000143970 | ENST00000420353 | (GA)3 |
| ATAD2 | ENSG00000156802 | ENST00000456903 | (ACG)3 |
| ATF7 | ENSG00000170653 | ENST00000588232 | (CT)3 |
|  |  | ENST00000551480 |  |
|  |  | ENST00000588078 |  |
|  |  | ENST00000548118 | (GCT)3 |
|  |  | ENST00000591397 |  |
|  |  | ENST00000533325 | (CT)3 |
|  |  | ENST00000578626 |  |
|  |  | ENST00000451513 |  |
| ATG13 | ENSG00000175224 |  | (GA)3 |
|  |  | ENST00000435760 | (TC)3 |
| ATG7 | ENSG00000197548 | ENST00000444619 | (AC)3 |
|  |  |  | (GAA)3 |
|  |  | ENST00000409422 | (AC)3 |
|  |  |  | (GAA)3 |
|  |  | ENST00000434939  ENST00000557735 | (AC)3 |
|  |  |  | (GAA)3 |
| ATG9A | ENSG00000198925 | ENST00000530958 | (TCT)3 |
|  |  |  | (CT)3 |
| ATL1 | ENSG00000198513 | ENST00000526567 | (TC)3 |
| ATM | ENSG00000149311 | ENST00000373673  ENST00000522314 | (TA)3 |
|  |  |  | (CT)3 |
|  |  | ENST00000617114 |  |
| ATOH7 | ENSG00000179774 | ENST00000400270 | (GA)3 |
| ATOX1 | ENSG00000177556 |  | (TG)3 |
| ATP13A2 | ENSG00000159363 | ENST00000620313 | (ACT)3 |
| ATP13A4 | ENSG00000127249 | ENST00000475483 | (TC)3 |
|  |  |  | (CT)3 |
| ATP13A5 | ENSG00000187527 | ENST00000515854 | (TA)3 |
|  |  |  |  |
| ATP1B3 | ENSG00000069849 | ENST00000504192  ENST00000534943 | (TCC)3 |
|  |  |  | (TAT)3 |
| ATP2C1 | ENSG00000017260 | ENST00000472505 | (AG)3 |
| ATP6V0A2 | ENSG00000185344 | ENST00000236067 | (GA)3 |
|  |  | ENST00000588255 | (AC)3 |
| ATP6V0B | ENSG00000117410 | ENST00000525591 | (GA)3 |
|  |  | ENST00000477775 | (GC)3 |
| ATP8B1 | ENSG00000081923 | ENST00000448930 | (CT)3 |
| ATP8B3 | ENSG00000130270 | ENST00000415300 | (GC)3 |
| ATXN7L1 | ENSG00000146776 | ENST00000598785 | (GT)3 |
| AURKC | ENSG00000105146 |  | (CAGCCCAG)3 |
|  |  |  | (CT)3 |
|  |  | ENST00000392781  ENST00000492353  ENST00000473142 |  |
|  |  |  | (TC)3 |
|  |  |  | (CAGCCCAG)3 |
| B3GALNT1 | ENSG00000169255 | ENST00000494173 | (TC)3 |
|  |  | ENST00000460353 |  |
|  |  |  |  |
|  |  | ENST00000528622 |  |
|  |  | ENST00000547141  ENST00000299204 | (GA)3 |
|  |  |  | (GC)3 |
| B3GNT6 | ENSG00000198488 | ENST00000557666 | (GT)3 |
| BACH1 | ENSG00000156273 | ENST00000456286 | (TGG)3 |
| BAG5 | ENSG00000166170 | ENST00000573677 | (CG)3 |
|  |  | ENST00000444316 |  |
| BAG6 | ENSG00000204463 | ENST00000423252 | (CCTT)3 |
| BAIAP2 | ENSG00000175866 | ENST00000439096 | (TG)3 |
| BANK1 | ENSG00000153064 | ENST00000524705 | (CA)3 |
| BANP | ENSG00000172530 | ENST00000295240 | (GCA)3 |
| BBC3 | ENSG00000105327 | ENST00000392663 | (CAG)3 |
| BBS1 | ENSG00000174483 | ENST00000425868 | (GCT)3 |
| BBS5 | ENSG00000163093 | ENST00000449213 | (GA)3 |
|  |  | ENST00000465919 |  |
| BBX | ENSG00000114439 | ENST00000569006 | (CAC)3 |
|  |  | ENST00000586705 |  |
| BCAP29 | ENSG00000075790 | ENST00000586484 | (GA)3 |
| BCAR1 | ENSG00000050820 | ENST00000305877 | (TC)3 |
| BCAS3 | ENSG00000141376 | ENST00000359540 |  |
|  |  | ENST00000380033 | (GCT)3 |
| BCR | ENSG00000186716 | ENST00000543160 | (CCG)3 |
|  |  | ENST00000333643 |  |
| BEND2 | ENSG00000177324 | ENST00000612929 | (CA)3 |
| BEX5 | ENSG00000184515 | ENST00000584603 | (AG)3 |
|  |  | ENST00000331593 |  |
| BHLHE23 | ENSG00000125533 | ENST00000523515 | (GCAGCG)3 |
| BLMH | ENSG00000108578 | ENST00000418040 | (GGA)3 |
| BMP8A | ENSG00000183682 | ENST00000449085 | (CGG)3 |
| BNIP3L | ENSG00000104765 | ENST00000594841 | (CT)3 |
| BPGM | ENSG00000172331 | ENST00000467963 |  |
| BRD2 | ENSG00000204256 | ENST00000489093 | (AGC)3 |
| BRD4 | ENSG00000141867 |  | (GC)3 |
| BRD9 | ENSG00000028310 | ENST00000619151 | (CCG)3 |
|  |  | ENST00000550208  ENST00000454960 | (CAGA)3 |
|  |  |  | (CT)3 |
| BRF1 | ENSG00000185024 | ENST00000296452 | (AG)3 |
|  |  | ENST00000494235 |  |
| BRPF3 | ENSG00000096070 | ENST00000314100 | (TG)3 |
| BSN | ENSG00000164061 |  | (GCG)3 |
| BTBD11 | ENSG00000151136 | ENST00000481247 | (CA)3 |
| BTBD9 | ENSG00000183826 | ENST00000419706  ENST00000494184 | (CT)3 |
|  |  |  | (AG)3 |
|  |  | ENST00000483410 |  |
|  |  | ENST00000438834 | (CT)3 |
| BTN2A2 | ENSG00000124508 | ENST00000310799 | (CTC)3 |
|  |  | ENST00000623205 |  |
| BZW2 | ENSG00000136261 | ENST00000330828 | (GA)3 |
| C11orf45 | ENSG00000174370 |  |  |
| C11orf71 | ENSG00000180425 | ENST00000378090 | (TG)3 |
| C12orf60 | ENSG00000182993 | ENST00000547975  ENST00000546819 | (AGA)3 |
|  |  |  | (TC)3 |
| C12orf73 | ENSG00000204954 | ENST00000319074 | (AG)3 |
|  |  | ENST00000554203 |  |
|  |  | ENST00000553764 |  |
| C14orf119 | ENSG00000179933 | ENST00000360639 | (GA)3 |
|  |  | ENST00000564848 |  |
| C14orf132 | ENSG00000227051 | ENST00000558435 | (TC)3 |
| C15orf39 | ENSG00000167173 | ENST00000559817 | (CA)3 |
|  |  | ENST00000602719 | (GAG)3 |
| C15orf48 | ENSG00000166920 |  | (AT)3 |
| C15orf59 | ENSG00000205363 |  | (GTG)3 |
| C16orf74 | ENSG00000154102 | ENST00000602583 | (CCG)3 |
|  |  |  | (CAC)3 |
|  |  |  | (CG)3 |
|  |  | ENST00000602675  ENST00000284245 | (TC)3 |
|  |  |  | (GT)3 |
|  |  |  |  |
|  |  |  | (CAT)3 |
|  |  | ENST00000602914 | (CCG)3 |
|  |  |  | (CAC)3 |
|  |  |  | (CG)3 |
|  |  | ENST00000602766  ENST00000542475  ENST00000546495 | (CCG)3 |
|  |  |  | (CAC)3 |
|  |  |  | (CG)3 |
|  |  | ENST00000546760 | (CAT)3 |
| C17orf100 | ENSG00000256806 | ENST00000552402 | (CT)3 |
| C17orf49 | ENSG00000258315 | ENST00000439424 | (GC)3 |
|  |  | ENST00000552775 |  |
|  |  | ENST00000582392 |  |
|  |  |  |  |
|  |  | ENST00000623113 | (GA)3 |
| C18orf32 | ENSG00000177576 | ENST00000614091 | (TAT)3 |
|  |  |  | (AG)3 |
| C19orf12 | ENSG00000131943 | ENST00000409293 | (CA)3 |
|  |  | ENST00000592872  ENST00000585755 | (CT)3 |
|  |  |  | (AG)3 |
| C19orf24 | ENSG00000228300 | ENST00000467127 | (CG)3 |
| C19orf25 | ENSG00000119559 | ENST00000427697 | (AG)3 |
| C19orf57 | ENSG00000132016 | ENST00000369102 | (CGC)3 |
| C1orf185 | ENSG00000204006 | ENST00000368243 | (GA)3 |
| C1orf198 | ENSG00000119280 |  | (AGG)3 |
| C1orf54 | ENSG00000118292 | ENST00000438241 | (AG)3 |
| C1orf61 | ENSG00000125462 | ENST00000510260  ENST00000580474 | (AAG)3 |
|  |  |  | (CT)3 |
| C1QA | ENSG00000173372 | ENST00000302514 | (AGGG)3 |
| C1QB | ENSG00000173369 |  | (AGA)3 |
| C1QTNF1 | ENSG00000173918 | ENST00000246199 | (TG)3 |
| C1QTNF4 | ENSG00000172247 | ENST00000382097 | (GC)3 |
|  |  |  | (GCAGCCC)3 |
| C20orf173 | ENSG00000125975 | ENST00000329623 | (AG)3 |
| C22orf42 | ENSG00000205856 | ENST00000380392  ENST00000419381 | (AGG)3 |
|  |  |  | (GT)3 |
| C2CD2 | ENSG00000157617 | ENST00000488469 | (GGT)3 |
| C2CD4B | ENSG00000205502 | ENST00000640868 | (AG)3 |
| C2orf42 | ENSG00000115998 | ENST00000640331 | (AGG)3 |
| C2orf74 | ENSG00000237651 | ENST00000435614 | (GA)3 |
| C2orf81 | ENSG00000284308 |  | (CT)3 |
|  |  | ENST00000412910 |  |
| C3orf20 | ENSG00000131379 | ENST00000315691 | (CAC)3 |
|  |  |  | (TC)3 |
|  |  | ENST00000434826  ENST00000442114 | (CAC)3 |
|  |  |  | (TC)3 |
| C3orf58 | ENSG00000181744 | ENST00000460742 | (GC)3 |
| C4orf45 | ENSG00000164123 | ENST00000379586 | (GAA)3 |
| C6orf223 | ENSG00000181577 | ENST00000503680 | (TC)3 |
| C6orf52 | ENSG00000137434 | ENST00000518698 | (AC)3 |
|  |  | ENST00000337103 |  |
|  |  |  |  |
| C8orf34 | ENSG00000165084 |  | (CTG)3 |
|  |  | ENST00000521889  ENST00000390159  ENST00000409586 | (TA)3 |
|  |  |  | (TTA)3 |
|  |  |  | (TG)3 |
| C8orf44 | ENSG00000213865 | ENST00000614574 | (CA)3 |
|  |  | ENST00000289989 |  |
| C8orf58 | ENSG00000241852 | ENST00000615223 | (GGCCGG)3 |
|  |  | ENST00000376854 |  |
|  |  | ENST00000372478 |  |
|  |  | ENST00000647196 | (AG)3 |
| C9orf40 | ENSG00000135045 |  | (TC)3 |
| C9orf50 | ENSG00000179058 | ENST00000318737 | (CG)3 |
| C9orf72 | ENSG00000147894 | ENST00000380685  ENST00000591725 | (CT)3 |
|  |  |  | (AT)3 |
| C9orf84 | ENSG00000165181 | ENST00000337989 | (AGT)3 |
| C9orf92 | ENSG00000205549 | ENST00000453000 | (AG)3 |
| CA4 | ENSG00000167434 | ENST00000496818 | (CTT)3 |
| CABIN1 | ENSG00000099991 | ENST00000637424 | (GA)3 |
| CABP1 | ENSG00000157782 | ENST00000638120 | (TG)3 |
| CACNA1C | ENSG00000151067 |  | (CA)3 |
| CACNA1D | ENSG00000157388 | ENST00000360963 | (AG)3 |
|  |  | ENST00000538450  ENST00000538027 | (GA)3 |
|  |  |  | (CAT)3 |
| CACNA2D2 | ENSG00000007402 | ENST00000324631 | (AG)3 |
| CACNA2D4 | ENSG00000151062 | ENST00000637284 | (TC)3 |
|  |  | ENST00000637514 |  |
| CACNB2 | ENSG00000165995 | ENST00000518457 | (GAG)3 |
| CACNB4 | ENSG00000182389 | ENST00000548177 | (TTC)3 |
|  |  | ENST00000395275 |  |
| CALB1 | ENSG00000104327 | ENST00000395276 | (ATT)3 |
| CALCOCO1 | ENSG00000012822 | ENST00000348335 | (AG)3 |
| CALN1 | ENSG00000183166 | ENST00000158166 | (CA)3 |
|  |  | ENST00000404169 |  |
| CAMKK1 | ENSG00000004660 | ENST00000402834 | (GCGGG)3 |
|  |  | ENST00000540525 |  |
| CAMKK2 | ENSG00000110931 | ENST00000449311 | (AGCCG)3 |
|  |  | ENST00000414281 |  |
| CAND1 | ENSG00000111530 | ENST00000420216 | (CT)3 |
| CAP1 | ENSG00000131236 | ENST00000427843 | (AG)3 |
|  |  | ENST00000424977 | (GCT)3 |
|  |  | ENST00000524773 |  |
|  |  | ENST00000328867 |  |
|  |  | ENST00000295055 |  |
| CAPN1 | ENSG00000014216 | ENST00000433674 | (GA)3 |
| CAPN12 | ENSG00000182472 | ENST00000434648 | (AG)3 |
| CAPN13 | ENSG00000162949 | ENST00000592354 | (AC)3 |
| CAPN2 | ENSG00000162909 | ENST00000341394 | (AAAG)3 |
|  |  | ENST00000389645 |  |
| CAPNS1 | ENSG00000126247 | ENST00000529307 | (CA)3 |
| CAPRIN1 | ENSG00000135387 |  | (ACC)3 |
|  |  | ENST00000397367 |  |
|  |  | ENST00000403299  ENST00000254691 | (GA)3 |
|  |  |  | (GT)3 |
| CAPSL | ENSG00000152611 | ENST00000611932 | (AG)3 |
| CARD10 | ENSG00000100065 | ENST00000614449 | (GCC)3 |
| CARD6 | ENSG00000132357 | ENST00000565287 | (AT)3 |
| CARHSP1 | ENSG00000153048 | ENST00000567908 | (CT)3 |
|  |  | ENST00000569398 |  |
|  |  | ENST00000531040 |  |
|  |  | ENST00000559222 | (TC)3 |
|  |  | ENST00000503684 | (CT)3 |
| CARNS1 | ENSG00000172508 |  | (GCC)3 |
| CASC4 | ENSG00000166734 | ENST00000621345 | (AG)3 |
| CASP6 | ENSG00000138794 | ENST00000429617  ENST00000369331 | (TC)3 |
|  |  |  | (CTG)3 |
| CASP7 | ENSG00000165806 | ENST00000429881 | (CG)3 |
|  |  | ENST00000556661 |  |
|  |  |  |  |
| CASP8 | ENSG00000064012 | ENST00000570046 | (CT)3 |
| CATSPERB | ENSG00000133962 | ENST00000314367  ENST00000382389 | (CTG)3 |
|  |  |  | (CA)3 |
| CBFA2T3 | ENSG00000129993 | ENST00000613716 | (TG)3 |
| CBWD1 | ENSG00000172785 | ENST00000536391 | (GAG)3 |
|  |  | ENST00000584775 |  |
| CBWD6 | ENSG00000215126 | ENST00000507501 |  |
| CCAR1 | ENSG00000060339 | ENST00000506962 | (GC)3 |
| CCDC102B | ENSG00000150636 | ENST00000615280 | (GT)3 |
| CCDC110 | ENSG00000168491 | ENST00000502801 | (CAG)3 |
|  |  | ENST00000343253 |  |
| CCDC129 | ENSG00000180347 | ENST00000522915 | (AC)3 |
| CCDC149 | ENSG00000181982 | ENST00000442740 | (CGG)3 |
| CCDC18 | ENSG00000122483 | ENST00000545036 | (CT)3 |
| CCDC25 | ENSG00000147419 | ENST00000335185 | (CAG)3 |
| CCDC51 | ENSG00000164051 | ENST00000307522 | (TG)3 |
| CCDC63 | ENSG00000173093 | ENST00000439685 | (GAA)3 |
| CCDC73 | ENSG00000186714 | ENST00000645366 | (GC)3 |
| CCDC8 | ENSG00000169515 | ENST00000530203 | (AGG)3 |
| CCDC80 | ENSG00000091986 | ENST00000621814 | (GAA)3 |
| CCDC82 | ENSG00000149231 | ENST00000359902 | (GT)3 |
|  |  |  |  |
| CCDC88A | ENSG00000115355 | ENST00000545135 | (AG)3 |
| CCDC88B | ENSG00000168071 | ENST00000614009  ENST00000502771 | (TC)3 |
|  |  |  | (CT)3 |
| CCDC92 | ENSG00000119242 | ENST00000395640 | (TC)3 |
| CCL14 | ENSG00000276409 | ENST00000509972 | (TGA)3 |
| CCND3 | ENSG00000112576 | ENST00000504878 | (AC)3 |
| CCNG2 | ENSG00000138764 | ENST00000507788 | (TG)3 |
|  |  | ENST00000557441 |  |
| CCNH | ENSG00000134480 | ENST00000374706 | (AG)3 |
| CCNI | ENSG00000118816 | ENST00000326306 | (GCGCCC)3 |
| CCNK | ENSG00000090061 |  | (TC)3 |
| CCNY | ENSG00000108100 | ENST00000515676 | (CT)3 |
| CCR8 | ENSG00000179934 | ENST00000506600  ENST00000626972 | (AG)3 |
|  |  |  | (GCT)3 |
| CCT5 | ENSG00000150753 | ENST00000607597 |  |
|  |  | ENST00000606471 |  |
| CCT8 | ENSG00000156261 | ENST00000426978 | (CG)3 |
| CD200 | ENSG00000091972 | ENST00000432207 | (AGAAGG)3 |
|  |  | ENST00000445143 |  |
| CD36 | ENSG00000135218 | ENST00000437761 | (AG)3 |
|  |  | ENST00000423145 |  |
| CD59 | ENSG00000085063 | ENST00000245903 | (AC)3 |
|  |  | ENST00000525210 |  |
| CD70 | ENSG00000125726 | ENST00000400259 | (CCTT)3 |
|  |  | ENST00000344548 |  |
| CD82 | ENSG00000085117 | ENST00000315554 | (AC)3 |
| CDC42 | ENSG00000070831 | ENST00000411827 | (TCA)3 |
|  |  | ENST00000366766 |  |
|  |  | ENST00000580824 |  |
|  |  | ENST00000380665 |  |
| CDC42BPA | ENSG00000143776 | ENST00000566827 | (GAA)3 |
| CDC6 | ENSG00000094804 | ENST00000418492 | (GTG)3 |
| CDCA2 | ENSG00000184661 | ENST00000224721 | (AG)3 |
| CDH11 | ENSG00000140937 |  | (TC)3 |
| CDH2 | ENSG00000170558 | ENST00000622827 | (GA)3 |
| CDH23 | ENSG00000107736 | ENST00000398809 | (GAGGC)3 |
|  |  |  | (CA)3 |
|  |  | ENST00000461841 | (GAGGC)3 |
|  |  |  | (CA)3 |
|  |  | ENST00000299366 | (GAGGC)3 |
|  |  |  | (CA)3 |
|  |  | ENST00000398842 | (GAGGC)3 |
|  |  |  | (CA)3 |
|  |  | ENST00000616684 | (GAGGC)3 |
|  |  |  | (CA)3 |
|  |  | ENST00000614565 | (GAGGC)3 |
|  |  |  | (CA)3 |
|  |  | ENST00000611855  ENST00000514738 | (GAGGC)3 |
|  |  |  | (CA)3 |
| CDH4 | ENSG00000179242 |  | (GCG)3 |
|  |  | ENST00000510636 | (GT)3 |
| CDH6 | ENSG00000113361 | ENST00000531088  ENST00000569956 | (CT)3 |
|  |  |  |  |
| CDHR2 /PCDH24 | ENSG00000074276 | ENST00000353379 |  |
| CDHR5 | ENSG00000099834 | ENST00000579632 | (GGA)3 |
| CDIPT | ENSG00000103502 | ENST00000536708 |  |
| CDK10 | ENSG00000185324 | ENST00000414822 | (AG)3 |
| CDK5RAP3 | ENSG00000108465 | ENST00000430149 | (TC)3 |
|  |  | ENST00000578845 | (CT)3 |
| CDKN1C | ENSG00000129757 |  | (CTC)3 |
|  |  | ENST00000494262 |  |
| CDKN2A | ENSG00000147889 | ENST00000370532  ENST00000563573 | (TG)3 |
|  |  |  | (TC)3 |
|  |  | ENST00000440139 | (CA)3 |
| CDR1 | ENSG00000184258 | ENST00000161559 | (AT)3 |
| CDR2 | ENSG00000140743 | ENST00000403444 | (TG)3 |
| CDYL | ENSG00000153046 | ENST00000599389 | (GAG)3 |
| CEACAM1 | ENSG00000079385 | ENST00000637215 | (AG)3 |
|  |  | ENST00000636488 |  |
|  |  | ENST00000508421 |  |
| CELF2 | ENSG00000048740 | ENST00000510693 | (CCT)3 |
|  |  | ENST00000533153 |  |
| CENPK | ENSG00000123219 | ENST00000525416 | (GAA)3 |
|  |  | ENST00000490813 |  |
| CEP164 | ENSG00000110274 | ENST00000522461 | (GC)3 |
|  |  | ENST00000524064 | (GA)3 |
| CEP170 | ENSG00000143702 | ENST00000540947 | (TG)3 |
| CEP57L1 | ENSG00000183137 | ENST00000540579 | (GAAT)3 |
|  |  | ENST00000535696 |  |
| CES4A | ENSG00000172824 | ENST00000541580 | (CA)3 |
|  |  | ENST00000475617 | (GCC)3 |
|  |  |  |  |
| CES5A | ENSG00000159398 | ENST00000425368 | (AG)3 |
| CFB | ENSG00000243649 | ENST00000320493 | (AC)3 |
|  |  |  | (TC)3 |
|  |  | ENST00000367424  ENST00000476712 | (AC)3 |
|  |  |  | (TC)3 |
| CFHR1 | ENSG00000244414 | ENST00000367415 | (TA)3 |
|  |  | ENST00000534769 |  |
| CFHR2 | ENSG00000080910 | ENST00000532134 |  |
|  |  | ENST00000534784 |  |
| CFL1 | ENSG00000172757 | ENST00000469388 | (TC)3 |
|  |  | ENST00000625577 |  |
|  |  | ENST00000474913 | (TGC)3 |
| CFP | ENSG00000126759 |  | (TG)3 |
| CGA | ENSG00000135346 | ENST00000448456 | (AT)3 |
| CGB2 | ENSG00000104818 | ENST00000312734  ENST00000640822 | (CTG)3 |
|  |  |  | (TGC)3 |
| CGB8 | ENSG00000213030 | ENST00000353665 | (AC)3 |
| CGREF1 | ENSG00000138028 | ENST00000435603 |  |
| CHAT | ENSG00000070748 | ENST00000526991 | (TC)3 |
| CHIA | ENSG00000134216 | ENST00000529987 | (CA)3 |
| CHL1 | ENSG00000134121 | ENST00000622063 | (AGC)3 |
| CHMP1B | ENSG00000255112 | ENST00000299847 | (GC)3 |
| CHORDC1 | ENSG00000110172 | ENST00000562729 | (AG)3 |
| CHRDL2 | ENSG00000054938 | ENST00000636168 | (GT)3 |
| CHRFAM7A | ENSG00000166664 | ENST00000567751 | (GA)3 |
|  |  | ENST00000565786 |  |
| CHRNA1 | ENSG00000138435 | ENST00000588411 | (TA)3 |
| CIAPIN1 | ENSG00000005194 | ENST00000629610 | (TGC)3 |
|  |  | ENST00000420484 |  |
| CIRBP | ENSG00000099622 | ENST00000553039 | (AGG)3 |
| CIZ1 | ENSG00000148337 | ENST00000553878 | (CAG)3 |
|  |  | ENST00000445369 | (GA)3 |
| CKAP4 | ENSG00000136026 | ENST00000395892 | (AG)3 |
| CKB | ENSG00000166165 | ENST00000185206 | (CGG)3 |
| CLDN9 | ENSG00000213937 | ENST00000540539 | (AC)3 |
| CLIC1 | ENSG00000213719 | ENST00000566126 |  |
| CLIC5 | ENSG00000112782 | ENST00000562670 | (TC)3 |
| CLIP1 | ENSG00000130779 | ENST00000564096 | (CTG)3 |
| CLK3 | ENSG00000179335 | ENST00000357857 | (GCG)3 |
|  |  | ENST00000395653 | (CT)3 |
|  |  | ENST00000567963 |  |
| CLN3 | ENSG00000188603 | ENST00000636228 | (GCG)3 |
|  |  | ENST00000381322 |  |
|  |  | ENST00000522413 |  |
|  |  | ENST00000519472 |  |
| CLOCK | ENSG00000134852 | ENST00000581619 | (GAT)3 |
| CLU | ENSG00000120885 |  | (CT)3 |
|  |  | ENST00000566462 | (CG)3 |
| CLUL1 | ENSG00000079101 | ENST00000562707  ENST00000565666 | (TC)3 |
|  |  |  | (CT)3 |
| CMIP | ENSG00000153815 | ENST00000504722 |  |
| CMTM3 | ENSG00000140931 | ENST00000514520 | (GT)3 |
|  |  |  | (AAG)3 |
| CNGA1 | ENSG00000198515 | ENST00000531191 | (CTG)3 |
|  |  | ENST00000585869 | (TA)3 |
|  |  |  |  |
| CNKSR1 | ENSG00000142675 | ENST00000348419 | (GGT)3 |
| CNN1 | ENSG00000130176 | ENST00000356162  ENST00000523698 | (AG)3 |
|  |  |  |  |
| CNN2 | ENSG00000064666 |  | (TCCCG)3 |
| CNOT4 | ENSG00000080802 | ENST00000521583 | (TC)3 |
| CNOT8 | ENSG00000155508 | ENST00000518775  ENST00000589772 | (AG)3 |
|  |  |  |  |
|  |  | ENST00000605888 | (GA)3 |
|  |  | ENST00000418500 |  |
| CNP | ENSG00000173786 |  | (AC)3 |
| COG5 | ENSG00000164597 | ENST00000400337 | (CTG)3 |
| COL10A1 | ENSG00000123500 | ENST00000370819  ENST00000390654 | (AC)3 |
|  |  |  | (TC)3 |
| COL18A1 | ENSG00000182871 | ENST00000646779 | (GCG)3 |
| COL21A1 | ENSG00000124749 | ENST00000622134 | (TC)3 |
| COL23A1 | ENSG00000050767 | ENST00000399132 | (CG)3 |
|  |  | ENST00000399126 | (CA)3 |
| COL25A1 | ENSG00000188517 | ENST00000372216 | (AG)3 |
|  |  | ENST00000422991 | (GA)3 |
|  |  | ENST00000515539 |  |
| COL4A6 | ENSG00000197565 | ENST00000542601 | (AG)3 |
| COL7A1 | ENSG00000114270 | ENST00000534771 | (GT)3 |
| COMMD10 | ENSG00000145781 | ENST00000517736 | (GCT)3 |
| COMP | ENSG00000105664 | ENST00000566110 | (TGG)3 |
| COPB1 | ENSG00000129083 | ENST00000544894 | (GC)3 |
| COPS5 | ENSG00000121022 | ENST00000561858 | (AC)3 |
| COQ7 | ENSG00000167186 | ENST00000563778 | (GAA)3 |
|  |  | ENST00000551044 |  |
|  |  | ENST00000584602 |  |
| CORO1A | ENSG00000102879 | ENST00000572549 | (CTC)3 |
| CORO1C | ENSG00000110880 | ENST00000571052 | (GT)3 |
| CORO6 | ENSG00000167549 | ENST00000570645 | (CA)3 |
| CORO7 | ENSG00000262246 | ENST00000575850 |  |
|  |  | ENST00000576051 | (AGC)3 |
|  |  | ENST00000388925 |  |
|  |  | ENST00000543464 | (CA)3 |
|  |  | ENST00000507071 | (AGC)3 |
| CPAMD8 | ENSG00000160111 | ENST00000345451 | (GGC)3 |
| CPD | ENSG00000108582 | ENST00000502265 | (AC)3 |
| CPEB2 | ENSG00000137449 | ENST00000546373 | (AGCCGC)3 |
|  |  | ENST00000565874 |  |
| CPLX2 | ENSG00000145920 | ENST00000523469 | (GC)3 |
| CPM | ENSG00000135678 | ENST00000522240 | (CT)3 |
| CPNE2 | ENSG00000140848 | ENST00000514999 |  |
| CPNE3 | ENSG00000085719 | ENST00000376618 | (GTG)3 |
|  |  | ENST00000265641 | (CTG)3 |
| CPNE4 | ENSG00000196353 | ENST00000405237 | (TA)3 |
| CPT1A | ENSG00000110090 | ENST00000409850 | (CTGCCG)3 |
|  |  | ENST00000458405 |  |
| CPT1B | ENSG00000205560 | ENST00000449801 | (GC)3 |
| CPVL | ENSG00000106066 | ENST00000455544 | (TA)3 |
|  |  | ENST00000437527 | (AG)3 |
|  |  | ENST00000542893 | (TA)3 |
|  |  | ENST00000448277 |  |
|  |  | ENST00000426500 |  |
| CRADD | ENSG00000169372 | ENST00000494479 | (AC)3 |
| CREB1 | ENSG00000118260 | ENST00000495301 | (TG)3 |
| CREB5 | ENSG00000146592 | ENST00000276571 | (CT)3 |
| CREM | ENSG00000095794 | ENST00000563066 | (AG)3 |
|  |  | ENST00000566789 |  |
| CRH | ENSG00000147571 | ENST00000392386 | (TC)3 |
| CRISPLD2 | ENSG00000103196 | ENST00000533709 | (AG)3 |
|  |  | ENST00000398133 |  |
| CRLF1 | ENSG00000006016 | ENST00000533280 | (CCG)3 |
| CRTAM | ENSG00000109943 | ENST00000227251 | (AC)3 |
| CRYAA | ENSG00000160202 | ENST00000526167 | (AG)3 |
| CRYAB | ENSG00000109846 |  | (TCT)3 |
|  |  | ENST00000550655 | (TC)3 |
|  |  | ENST00000370291  ENST00000370287 | (AG)3 |
|  |  |  | (TCT)3 |
| CS | ENSG00000062485 | ENST00000412290 | (CGC)3 |
| CSAG1 | ENSG00000198930 | ENST00000453363 | (TA)3 |
|  |  | ENST00000316193 |  |
| CSF2RA | ENSG00000198223 | ENST00000392886 | (GA)3 |
| CSH1 | ENSG00000136488 | ENST00000336844 | (AG)3 |
|  |  | ENST00000560142 |  |
| CSH2 | ENSG00000213218 | ENST00000345366 |  |
|  |  | ENST00000359867 |  |
|  |  | ENST00000400217 |  |
|  |  |  |  |
| CSNK1E | ENSG00000213923 | ENST00000645091 | (CT)3 |
| CSNK2A1 | ENSG00000101266 | ENST00000375865 | (TG)3 |
|  |  |  | (GCC)3 |
|  |  | ENST00000619238  ENST00000594565 | (TG)3 |
|  |  |  | (GCC)3 |
| CSNK2B | ENSG00000204435 | ENST00000593606 | (TG)3 |
| CSTL1 | ENSG00000125823 | ENST00000328435 | (TGC)3 |
| CT45A1 | ENSG00000268940 | ENST00000309035 | (TC)3 |
| CTAG1A | ENSG00000268651 | ENST00000609232 |  |
| CTAG1B | ENSG00000184033 | ENST00000429804 |  |
| CTBP2 | ENSG00000175029 | ENST00000243914 | (CA)3 |
| CTCFL | ENSG00000124092 | ENST00000558966 | (GGC)3 |
|  |  | ENST00000523912 |  |
|  |  | ENST00000302763 |  |
| CTDSPL2 | ENSG00000137770 | ENST00000518381 | (TCC)3 |
| CTNNA1 | ENSG00000044115 | ENST00000520260 | (CTC)3 |
|  |  | ENST00000520865 | (TCC)3 |
|  |  | ENST00000519634 | (GA)3 |
|  |  | ENST00000523685 |  |
|  |  | ENST00000519768 |  |
|  |  | ENST00000517656 |  |
|  |  | ENST00000521683 |  |
|  |  | ENST00000521640 |  |
|  |  | ENST00000519116 |  |
|  |  | ENST00000520522 |  |
|  |  | ENST00000409971 |  |
|  |  | ENST00000529526 |  |
|  |  | ENST00000426142 |  |
| CTNNA2 | ENSG00000066032 | ENST00000532463 | (AC)3 |
| CTNND1 | ENSG00000198561 | ENST00000529986 | (GGA)3 |
|  |  | ENST00000532787 | (TA)3 |
|  |  | ENST00000532649 |  |
|  |  | ENST00000528621 |  |
|  |  | ENST00000530748 |  |
|  |  | ENST00000528232 | (GGA)3 |
|  |  | ENST00000529873 |  |
|  |  | ENST00000532844 |  |
|  |  | ENST00000526357 | (TA)3 |
|  |  | ENST00000530094 | (GGA)3 |
|  |  | ENST00000415361 |  |
|  |  | ENST00000532245 |  |
|  |  | ENST00000534579 | (TA)3 |
|  |  | ENST00000530068 |  |
|  |  | ENST00000534647 |  |
|  |  | ENST00000577589 | (GGA)3 |
|  |  | ENST00000541347 |  |
|  |  | ENST00000428565 |  |
| CUEDC1 | ENSG00000180891 | ENST00000373691 | (AGG)3 |
| CX3CR1 | ENSG00000168329 | ENST00000378962 | (CA)3 |
| CXCR2 | ENSG00000180871 | ENST00000428236 | (CAC)3 |
| CXCR3 | ENSG00000186810 | ENST00000359293 | (GA)3 |
| CXorf21 | ENSG00000120280 | ENST00000581573 | (AGA)3 |
| CXorf40A | ENSG00000197620 | ENST00000494131 | (GA)3 |
|  |  | ENST00000524608 |  |
| CYB561 | ENSG00000008283 | ENST00000569662 | (TG)3 |
| CYB5A | ENSG00000166347 | ENST00000396402 | (GC)3 |
| CYB5R2 | ENSG00000166394 | ENST00000561075 | (CT)3 |
| CYP11A1 | ENSG00000140459 | ENST00000453807 | (GC)3 |
| CYP19A1 | ENSG00000137869 | ENST00000557858 | (GAA)3 |
|  |  | ENST00000559980 |  |
|  |  | ENST00000412253 | (AG)3 |
|  |  |  | (GAA)3 |
|  |  | ENST00000335247 | (AG)3 |
| CYP26B1 | ENSG00000003137 | ENST00000593831  ENST00000508453 | (AC)3 |
|  |  |  | (GC)3 |
| CYP27C1 | ENSG00000186684 | ENST00000619708 | (CTC)3 |
| CYP2B6 | ENSG00000197408 | ENST00000586043 | (GA)3 |
| CYP2U1 | ENSG00000155016 | ENST00000587308 | (TC)3 |
| CYP39A1 | ENSG00000146233 | ENST00000436575 | (GAA)3 |
| CYTH1 | ENSG00000108669 | ENST00000329625 | (AGA)3 |
|  |  |  | (CA)3 |
| DAGLB | ENSG00000164535 | ENST00000230895 | (CTG)3 |
| DAOA | ENSG00000182346 | ENST00000432074  ENST00000441323 | (CT)3 |
|  |  |  | (AG)3 |
| DAP | ENSG00000112977 | ENST00000456565 | (CGC)3 |
|  |  |  |  |
| DARS | ENSG00000115866 | ENST00000449218 | (AG)3 |
|  |  | ENST00000372723  ENST00000372722 |  |
|  |  |  |  |
|  |  | ENST00000357275 |  |
| DBNDD2 | ENSG00000244274 | ENST00000448521 | (GC)3 |
|  |  | ENST00000456905 |  |
|  |  | ENST00000521999 |  |
| DBNL | ENSG00000136279 | ENST00000556847 | (CGGCC)3 |
|  |  | ENST00000554681 |  |
| DCAF13 | ENSG00000164934 | ENST00000447377 | (AG)3 |
| DCAF5 | ENSG00000139990 | ENST00000441525 | (TC)3 |
|  |  | ENST00000633691 | (GAG)3 |
| DCAF8 | ENSG00000132716 | ENST00000409438 | (AG)3 |
| DCAF8L1 | ENSG00000226372 | ENST00000413111 | (CT)3 |
| DCTN1 | ENSG00000204843 | ENST00000421392 | (GT)3 |
|  |  | ENST00000440727 |  |
|  |  | ENST00000566298 | (AG)3 |
|  |  | ENST00000416410 | (GA)3 |
|  |  | ENST00000527834 | (AG)3 |
| DCTN5 | ENSG00000166847 | ENST00000520272 | (TG)3 |
| DDAH2 | ENSG00000213722 | ENST00000480945 | (CT)3 |
| DDHD2 | ENSG00000085788 | ENST00000332395 | (TGC)3 |
|  |  | ENST00000359353 | (GA)3 |
| DDI2 | ENSG00000197312 | ENST00000552375 | (CCGAG)3 |
| DDX28 | ENSG00000182810 | ENST00000505696 | (CA)3 |
| DDX42 | ENSG00000198231 | ENST00000519410 | (GAT)3 |
| DDX54 | ENSG00000123064 | ENST00000334391 | (GA)3 |
| DDX60L | ENSG00000181381 | ENST00000519018 |  |
| DECR1 | ENSG00000104325 | ENST00000264093 | (AGA)3 |
| DEFB128 | ENSG00000185982 | ENST00000348222 | (CA)3 |
| DERL1 | ENSG00000136986 | ENST00000629438 | (TAA)3 |
| DGUOK | ENSG00000114956 | ENST00000597152 | (TG)3 |
|  |  | ENST00000396813 |  |
|  |  | ENST00000534993 |  |
| DHPS | ENSG00000095059 | ENST00000543805 | (GCC)3 |
| DHRS1 | ENSG00000157379 | ENST00000481941 | (CTC)3 |
| DHRS4L2 | ENSG00000187630 |  | (TG)3 |
|  |  | ENST00000566794 |  |
| DHX36 | ENSG00000174953 | ENST00000645606  ENST00000253811 | (AG)3 |
|  |  |  |  |
| DHX38 | ENSG00000140829 | ENST00000553968 | (CT)3 |
| DIABLO | ENSG00000284934 | ENST00000585334 | (GT)3 |
| DIAPH1 | ENSG00000131504 | ENST00000375765 | (AAG)3 |
| DIO2 | ENSG00000211448 | ENST00000513208 | (AG)3 |
| DIRAS1 | ENSG00000176490 | ENST00000510463 | (CT)3 |
| DIRAS2 | ENSG00000165023 | ENST00000530800 | (CA)3 |
| DKK2 | ENSG00000155011 | ENST00000527088 | (AT)3 |
|  |  | ENST00000637795 |  |
| DLG2 | ENSG00000150672 | ENST00000338354 | (AGG)3 |
|  |  | ENST00000344338 | (TG)3 |
| DLGAP2 | ENSG00000198010 | ENST00000330163 | (GGAC)3 |
| DMBT1 | ENSG00000187908 | ENST00000368955 | (AC)3 |
|  |  | ENST00000368909 |  |
|  |  | ENST00000368956 |  |
|  |  | ENST00000378677 |  |
|  |  | ENST00000472252 |  |
|  |  | ENST00000492341 |  |
| DMD | ENSG00000198947 | ENST00000461300 | (GT)3 |
| DMKN | ENSG00000161249 | ENST00000596067 | (CTC)3 |
|  |  | ENST00000600017 |  |
|  |  | ENST00000596258 |  |
| DMPK | ENSG00000104936 | ENST00000572933 | (GA)3 |
| DMRTC2 | ENSG00000142025 | ENST00000327475 |  |
|  |  | ENST00000579490 | (TCC)3 |
| DNAH2 | ENSG00000183914 | ENST00000394852 | (AC)3 |
| DNAH8 | ENSG00000124721 | ENST00000396969 | (AG)3 |
| DNAI2 | ENSG00000171595 | ENST00000596853 |  |
| DNAJA4 | ENSG00000140403 | ENST00000595992 | (CG)3 |
| DNAJB1 | ENSG00000132002 | ENST00000425450 | (GA)3 |
|  |  | ENST00000547445 |  |
|  |  | ENST00000479269 |  |
| DNAJB2 | ENSG00000135924 | ENST00000296097 | (GC)3 |
| DNAJC14 | ENSG00000135392 | ENST00000420191 | (TA)3 |
| DNAJC19 | ENSG00000205981 | ENST00000402462 | (TGC)3 |
| DNAJC5G | ENSG00000163793 | ENST00000340748 | (GAA)3 |
|  |  | ENST00000359526 |  |
|  |  | ENST00000519905 |  |
| DNMT1 | ENSG00000130816 | ENST00000616445 | (GCC)3 |
|  |  | ENST00000564944 |  |
| DNPEP | ENSG00000123992 | ENST00000258390 | (AC)3 |
| DOC2A | ENSG00000149927 | ENST00000540750 | (GT)3 |
|  |  | ENST00000410074 |  |
| DOCK10 | ENSG00000135905 | ENST00000481100 | (GC)3 |
| DOCK2 | ENSG00000134516 | ENST00000427887 | (CA)3 |
| DOCK5 | ENSG00000147459 | ENST00000510380 | (CGG)3 |
|  |  | ENST00000506493 |  |
| DOCK9 | ENSG00000088387 | ENST00000502885 | (AGGCGG)3 |
| DOK3 | ENSG00000146094 | ENST00000410059 | (CG)3 |
|  |  |  |  |
|  |  | ENST00000532019 |  |
| DPP10 | ENSG00000175497 | ENST00000598800  ENST00000324472 | (GCA)3 |
|  |  |  | (CAG)3 |
| DPP3 | ENSG00000254986 | ENST00000542616 | (GAG)3 |
| DPP9 | ENSG00000142002 | ENST00000400454 | (GA)3 |
| DPY19L2 | ENSG00000177990 | ENST00000523817 | (TC)3 |
| DRD2 | ENSG00000149295 | ENST00000591816 | (AG)3 |
| DSCAM | ENSG00000171587 | ENST00000532982 | (GGCG)3 |
| DST | ENSG00000151914 | ENST00000558996 | (GA)3 |
| DTNA | ENSG00000134769 | ENST00000559988 |  |
| DTX4 | ENSG00000110042 | ENST00000486996 | (CT)3 |
| DUOXA1 | ENSG00000140254 | ENST00000398084 | (GC)3 |
|  |  | ENST00000398083 |  |
| DUSP15 | ENSG00000149599 | ENST00000339042 | (TC)3 |
|  |  | ENST00000547291 |  |
|  |  | ENST00000558813 |  |
| DUSP21 | ENSG00000189037 | ENST00000558367 | (AG)3 |
| DUSP6 | ENSG00000139318 | ENST00000457059 | (GT)3 |
| DUT | ENSG00000128951 | ENST00000430778 | (GA)3 |
|  |  | ENST00000647425 | (AG)3 |
| DYNC1I1 | ENSG00000158560 | ENST00000643551 | (ATG)3 |
| DYNC1I2 | ENSG00000077380 | ENST00000426672 | (TA)3 |
| DYRK1A | ENSG00000157540 | ENST00000554792 | (AG)3 |
|  |  | ENST00000495186 | (CCGGG)3 |
|  |  | ENST00000473505 |  |
| EAPP | ENSG00000129518 | ENST00000422887 | (TTG)3 |
| EBP | ENSG00000147155 | ENST00000326842 | (AG)3 |
| ECE1 | ENSG00000117298 | ENST00000326783 | (TG)3 |
| ECHDC3 | ENSG00000134463 | ENST00000423316 | (GT)3 |
| EDDM3A | ENSG00000181562 | ENST00000419152 | (GGA)3 |
| EDDM3B | ENSG00000181552 | ENST00000534380 | (CA)3 |
| EEF1D | ENSG00000104529 | ENST00000533204 | (GT)3 |
|  |  | ENST00000530191 | (GA)3 |
|  |  | ENST00000366522 |  |
|  |  | ENST00000638075 |  |
|  |  | ENST00000397202 |  |
| EFCAB2 | ENSG00000203666 | ENST00000530028 | (TG)3 |
| EFHC1 | ENSG00000096093 |  | (CA)3 |
| EGFLAM | ENSG00000164318 | ENST00000390658 | (AG)3 |
| EID1 | ENSG00000255302 | ENST00000532707  ENST00000620860 | (GC)3 |
|  |  |  |  |
| EID2 | ENSG00000176396 | ENST00000360403 |  |
| EIF1AD | ENSG00000175376 | ENST00000372183 | (CT)3 |
| EIF2B3 | ENSG00000070785 | ENST00000431643 | (CA)3 |
|  |  | ENST00000566501 |  |
|  |  | ENST00000563139 |  |
| EIF3B | ENSG00000106263 | ENST00000568426 |  |
| EIF3C | ENSG00000184110 | ENST00000398944 | (CT)3 |
|  |  | ENST00000518345 | (AG)3 |
|  |  | ENST00000518949 |  |
| EIF3CL | ENSG00000205609 | ENST00000593149 | (CT)3 |
| EIF3E | ENSG00000104408 | ENST00000538434 | (AT)3 |
| EIF3H | ENSG00000147677 | ENST00000504432 | (AAAGGC)3 |
| EIF3K | ENSG00000178982 | ENST00000544538 | (CA)3 |
|  |  | ENST00000521099 |  |
| EIF4E | ENSG00000151247 | ENST00000339841 | (CT)3 |
| ELAVL2 | ENSG00000107105 |  | (CAG)3 |
| ELP3 | ENSG00000134014 | ENST00000596043 | (AG)3 |
| ELSPBP1 | ENSG00000169393 | ENST00000597519 | (GA)3 |
|  |  |  |  |
|  |  | ENST00000303221 |  |
|  |  |  |  |
|  |  | ENST00000508934  ENST00000590018 |  |
|  |  |  |  |
| EMB | ENSG00000170571 | ENST00000554922 | (GCG)3 |
|  |  | ENST00000599704 |  |
| EML2 | ENSG00000125746 | ENST00000597279 | (ATAA)3 |
| EML5 | ENSG00000165521 | ENST00000480266 | (CGC)3 |
| EMP3 | ENSG00000142227 |  | (CG)3 |
|  |  | ENST00000369207 |  |
| ENG | ENSG00000106991 | ENST00000251101  ENST00000230565 | (CTG)3 |
|  |  |  | (TCC)3 |
| ENO4 | ENSG00000188316 | ENST00000373946 | (CCA)3 |
| ENOSF1 | ENSG00000132199 | ENST00000452261 | (CTCCCGCC)3 |
| ENPP5 | ENSG00000112796 | ENST00000447825 | (GC)3 |
| EPB41L1 | ENSG00000088367 | ENST00000526983 | (TG)3 |
|  |  | ENST00000342933 |  |
|  |  | ENST00000341928 | (TC)3 |
| EPB41L2 | ENSG00000079819 | ENST00000540638 | (CA)3 |
| EPB41L3 | ENSG00000082397 | ENST00000580308 | (CCG)3 |
|  |  | ENST00000580989 |  |
|  |  | ENST00000476620 |  |
|  |  | ENST00000419964 | (GT)3 |
|  |  | ENST00000514100 |  |
| EPDR1 | ENSG00000086289 | ENST00000502694 | (AT)3 |
| EPHA4 | ENSG00000116106 | ENST00000473867 | (GA)3 |
| EPHA6 | ENSG00000080224 | ENST00000411471 | (TCA)3 |
|  |  | ENST00000445856 |  |
| EPHB1 | ENSG00000154928 | ENST00000272167 | (GA)3 |
| EPHB6 | ENSG00000106123 | ENST00000521780 | (TG)3 |
| EPHX1 | ENSG00000143819 | ENST00000380476 | (GCC)3 |
|  |  | ENST00000411543 |  |
| EPHX2 | ENSG00000120915 | ENST00000543363 | (GC)3 |
|  |  | ENST00000586329 |  |
| EPN1 | ENSG00000063245 | ENST00000636362 | (CT)3 |
| EPS8 | ENSG00000151491 | ENST00000549061 | (TA)3 |
| EPS8L1 | ENSG00000131037 | ENST00000549672 | (TCC)3 |
| ERAS | ENSG00000187682 | ENST00000415288 | (GC)3 |
| ERBB3 | ENSG00000065361 | ENST00000342788 | (GT)3 |
|  |  | ENST00000436443 | (TG)3 |
|  |  | ENST00000423698 |  |
| ERBB4 | ENSG00000178568 | ENST00000515869 | (CG)3 |
|  |  |  |  |
| ERCC1 | ENSG00000012061 | ENST00000546839 | (TC)3 |
| ERCC6 | ENSG00000225830 | ENST00000421367  ENST00000420317 | (GC)3 |
|  |  |  | (AG)3 |
| ERGIC2 | ENSG00000087502 | ENST00000411762 | (GA)3 |
| ERLIN1 | ENSG00000107566 | ENST00000622333 |  |
| ERMN | ENSG00000136541 |  | (AC)3 |
|  |  | ENST00000423032 |  |
| ESCO1 | ENSG00000141446 | ENST00000560726  ENST00000586826 |  |
|  |  |  | (AAGA)3 |
| ESPNL | ENSG00000144488 | ENST00000306376 | (TTC)3 |
| ETFA | ENSG00000140374 | ENST00000440773 | (GA)3 |
| ETV4 | ENSG00000175832 | ENST00000421809 | (TCC)3 |
| ETV5 | ENSG00000244405 | ENST00000620358 | (GA)3 |
|  |  | ENST00000615781 |  |
|  |  | ENST00000556318 |  |
| ETV7 | ENSG00000010030 | ENST00000420116 | (CT)3 |
|  |  | ENST00000442951 |  |
| EXOC5 | ENSG00000070367 | ENST00000527954 | (AT)3 |
| EXOC7 | ENSG00000182473 | ENST00000622749 | (TC)3 |
|  |  | ENST00000536991 |  |
| EXOSC4 | ENSG00000178896 | ENST00000539419 | (GT)3 |
| F8A3 | ENSG00000277150 | ENST00000355484 | (GCG)3 |
| FADS1 | ENSG00000149485 | ENST00000550890 | (TC)3 |
|  |  | ENST00000468747 | (AG)3 |
| FADS2 | ENSG00000134824 | ENST00000381941 | (TC)3 |
| FAIM2 | ENSG00000135472 | ENST00000304189 | (CAG)3 |
| FAM107B | ENSG00000065809 | ENST00000381939 | (CGC)3 |
| FAM110A | ENSG00000125898 | ENST00000246100 | (TC)3 |
|  |  | ENST00000541082 |  |
|  |  | ENST00000411426 |  |
|  |  | ENST00000513602 |  |
|  |  | ENST00000646004 |  |
| FAM111B | ENSG00000189057 |  | (GA)3 |
| FAM117A | ENSG00000121104 | ENST00000370784 | (TCC)3 |
| FAM122C | ENSG00000156500 | ENST00000370785 | (CT)3 |
|  |  |  |  |
|  |  | ENST00000475361 |  |
|  |  |  |  |
|  |  | ENST00000643150 |  |
|  |  |  |  |
|  |  | ENST00000449408 |  |
|  |  |  |  |
|  |  | ENST00000445716  ENST00000398106 |  |
|  |  |  |  |
| FAM129C | ENSG00000167483 | ENST00000620373 | (CGA)3 |
| FAM133B | ENSG00000234545 | ENST00000622447 | (TC)3 |
| FAM153C | ENSG00000204677 | ENST00000617970 | (AT)3 |
|  |  | ENST00000611661 |  |
| FAM156A | ENSG00000268350 | ENST00000596733 | (CA)3 |
|  |  | ENST00000623782 |  |
|  |  | ENST00000615092 |  |
|  |  | ENST00000612915 |  |
|  |  | ENST00000612846 |  |
|  |  | ENST00000622197 |  |
|  |  | ENST00000619373 |  |
|  |  | ENST00000622732 |  |
|  |  | ENST00000619518 |  |
|  |  | ENST00000618601 |  |
|  |  | ENST00000622323 |  |
|  |  | ENST00000612083 |  |
|  |  | ENST00000232125 |  |
|  |  | ENST00000514200 |  |
|  |  | ENST00000553393 |  |
| FAM162A | ENSG00000114023 | ENST00000621231 | (AG)3 |
| FAM169A | ENSG00000198780 | ENST00000265018 | (CG)3 |
| FAM174B | ENSG00000185442 | ENST00000590648 | (TG)3 |
| FAM184A | ENSG00000111879 |  | (GCA)3 |
| FAM184B | ENSG00000047662 | ENST00000592586 | (GA)3 |
| FAM198B | ENSG00000164125 | ENST00000552075 | (AG)3 |
|  |  |  | (GA)3 |
|  |  | ENST00000549958 | (CAT)3 |
| FAM19A2 | ENSG00000198673 | ENST00000548780  ENST00000295569 | (CT)3 |
|  |  |  | (AT)3 |
|  |  | ENST00000495737 | (TC)3 |
|  |  | ENST00000634242 |  |
| FAM19A4 | ENSG00000163377 | ENST00000406880 | (GT)3 |
|  |  | ENST00000332947 |  |
|  |  | ENST00000510197 |  |
| FAM19A5 | ENSG00000219438 | ENST00000393600 | (AG)3 |
| FAM43B | ENSG00000183114 | ENST00000558348 | (GC)3 |
| FAM47E | ENSG00000189157 |  | (GGA)3 |
| FAM50A | ENSG00000071859 | ENST00000560394 | (GCCGCT)3 |
| FAM81A | ENSG00000157470 | ENST00000333407 | (AG)3 |
|  |  |  | (GA)3 |
|  |  | ENST00000473717  ENST00000381003 | (AG)3 |
|  |  |  | (GA)3 |
| FAM83F | ENSG00000133477 | ENST00000562892 | (CCGGGG)3 |
|  |  | ENST00000433829 | (CT)3 |
| FAM9A | ENSG00000183304 | ENST00000327470 | (AG)3 |
| FAN1 | ENSG00000198690 |  | (GAA)3 |
| FANCC | ENSG00000158169 | ENST00000445510 | (GA)3 |
| FANCF | ENSG00000183161 | ENST00000368689  ENST00000598389 | (AG)3 |
|  |  |  |  |
| FANK1 | ENSG00000203780 | ENST00000324331 | (AT)3 |
|  |  | ENST00000588025 | (CA)3 |
| FARP1 | ENSG00000152767 | ENST00000445110 | (AC)3 |
| FARS2 | ENSG00000145982 | ENST00000601739 | (GA)3 |
| FARSA | ENSG00000179115 | ENST00000585379 | (CA)3 |
| FBLN1 | ENSG00000077942 | ENST00000562563 | (TG)3 |
| FBN3 | ENSG00000142449 | ENST00000620087 | (TC)3 |
| FBXL12 | ENSG00000127452 |  | (GT)3 |
| FBXL16 | ENSG00000127585 | ENST00000424163 | (CGCC)3 |
| FBXL18 | ENSG00000155034 | ENST00000595329  ENST00000518240 | (CG)3 |
|  |  |  |  |
| FBXO11 | ENSG00000138081 | ENST00000526785 | (TGA)3 |
| FBXO17 | ENSG00000269190 | ENST00000586899 | (AG)3 |
| FBXO25 | ENSG00000147364 | ENST00000436231 | (CG)3 |
| FBXO3 | ENSG00000110429 | ENST00000534938 | (TC)3 |
| FBXO46 | ENSG00000177051 | ENST00000594202 | (TG)3 |
| FBXW12 | ENSG00000164049 | ENST00000600209 | (TC)3 |
| FCF1 | ENSG00000119616 | ENST00000598067 | (GA)3 |
| FCHO1 | ENSG00000130475 | ENST00000596536 | (TG)3 |
|  |  | ENST00000598932 |  |
|  |  | ENST00000594068 |  |
|  |  | ENST00000596507 |  |
|  |  | ENST00000597718 |  |
|  |  | ENST00000596462 |  |
|  |  | ENST00000596865 |  |
|  |  | ENST00000518499 |  |
|  |  | ENST00000536257 |  |
|  |  | ENST00000321935 |  |
| FCHSD1 | ENSG00000197948 | ENST00000525777 | (CT)3 |
| FCRL6 | ENSG00000181036 | ENST00000522917 | (AC)3 |
|  |  | ENST00000327809 |  |
| FDFT1 | ENSG00000079459 | ENST00000608478 | (AT)3 |
| FER1L6 | ENSG00000214814 | ENST00000644866 | (TG)3 |
| FFAR3 | ENSG00000185897 | ENST00000560979 | (GA)3 |
| FGF2 | ENSG00000138685 | ENST00000530568 | (GGCCGG)3 |
|  |  | ENST00000544111 |  |
| FGF7 | ENSG00000140285 | ENST00000510644 | (AC)3 |
| FGFR1 | ENSG00000077782 | ENST00000374004 | (CGC)3 |
| FGFR1OP2 | ENSG00000111790 | ENST00000628568 | (GT)3 |
| FGFRL1 | ENSG00000127418 | ENST00000372338 | (CCGC)3 |
| FGR | ENSG00000000938 | ENST00000354552 | (GT)3 |
| FHL1 | ENSG00000022267 | ENST00000487087 | (AT)3 |
| FIBCD1 | ENSG00000130720 | ENST00000331335 | (CCCGCG)3 |
| FILIP1L | ENSG00000168386 | ENST00000398326 | (GTT)3 |
|  |  | ENST00000400137 | (TGG)3 |
|  |  | ENST00000618612 | (GTT)3 |
|  |  | ENST00000439640 |  |
| FKBP1A | ENSG00000088832 | ENST00000381719 | (CCGC)3 |
|  |  | ENST00000614856 |  |
|  |  | ENST00000535135 |  |
|  |  | ENST00000596558 |  |
|  |  | ENST00000593800 |  |
| FKBP2 | ENSG00000173486 | ENST00000493452 | (TG)3 |
| FKBP8 | ENSG00000105701 | ENST00000438162 | (TC)3 |
| FKRP | ENSG00000181027 | ENST00000454845 |  |
| FLNB | ENSG00000136068 | ENST00000615611 | (AT)3 |
| FLOT1 | ENSG00000137312 | ENST00000319653 | (AG)3 |
|  |  | ENST00000540172 |  |
| FLT1 | ENSG00000102755 | ENST00000529117 | (GA)3 |
| FMN2 | ENSG00000155816 |  | (GCC)3 |
| FNBP4 | ENSG00000109920 | ENST00000546166 | (CT)3 |
| FOLH1 | ENSG00000086205 | ENST00000592811 |  |
|  |  |  | (TG)3 |
| FOLR3 | ENSG00000110203 | ENST00000586615 | (AG)3 |
|  |  |  | (CT)3 |
| FOSB | ENSG00000125740 | ENST00000589593 | (TG)3 |
|  |  |  | (GT)3 |
|  |  | ENST00000436647  ENST00000396057 | (TG)3 |
|  |  |  | (GT)3 |
|  |  |  | (AGG)3 |
| FOSL2 | ENSG00000075426 | ENST00000334793 | (GT)3 |
| FOXB1 | ENSG00000171956 | ENST00000335071  ENST00000422278 | (GA)3 |
|  |  |  | (GC)3 |
| FOXD2 | ENSG00000186564 | ENST00000339140 |  |
| FOXE3 | ENSG00000186790 | ENST00000600815 | (CG)3 |
| FOXJ3 | ENSG00000198815 | ENST00000524583 | (AT)3 |
| FOXR2 | ENSG00000189299 |  | (TC)3 |
| FPR1 | ENSG00000171051 | ENST00000531991 | (CT)3 |
| FRG1 | ENSG00000109536 | ENST00000460709 | (AGA)3 |
|  |  |  | (TA)3 |
|  |  | ENST00000417257 | (AGA)3 |
| FRMD4B | ENSG00000114541 | ENST00000402883  ENST00000636859 | (ACAC)3 |
|  |  |  | (CT)3 |
| FRMD5 | ENSG00000171877 |  | (GGGC)3 |
|  |  | ENST00000547414 |  |
|  |  | ENST00000506685  ENST00000505759 | (CT)3 |
|  |  |  | (TC)3 |
| FRS2 | ENSG00000166225 | ENST00000592947 | (GAT)3 |
| FRYL | ENSG00000075539 |  | (GCT)3 |
|  |  | ENST00000588773 | (TG)3 |
| FSTL3 | ENSG00000070404 | ENST00000591573 | (GT)3 |
|  |  |  | (CAA)3 |
|  |  | ENST00000510685 | (GT)3 |
|  |  |  | (CAA)3 |
|  |  | ENST00000291670  ENST00000397748 | (GT)3 |
|  |  |  | (CAA)3 |
| FSTL4 | ENSG00000053108 | ENST00000397746 | (CA)3 |
| FTCD | ENSG00000160282 | ENST00000397743 | (AG)3 |
|  |  | ENST00000431610 |  |
|  |  | ENST00000588525 |  |
|  |  | ENST00000527106 |  |
| FTO | ENSG00000140718 | ENST00000531085 | (AT)3 |
| FUT5 | ENSG00000130383 |  | (TG)3 |
| FUT6 | ENSG00000156413 | ENST00000377270 | (AC)3 |
|  |  | ENST00000588607  ENST00000588081 | (TC)3 |
|  |  |  | (AC)3 |
| FXN | ENSG00000165060 | ENST00000392218 | (CT)3 |
| FXYD1 | ENSG00000266964 | ENST00000543307 | (CA)3 |
|  |  | ENST00000392219 |  |
| FXYD5 | ENSG00000089327 | ENST00000541435 | (TC)3 |
|  |  | ENST00000590686 |  |
|  |  | ENST00000438446 |  |
|  |  | ENST00000287934 |  |
|  |  | ENST00000295417 |  |
| FYCO1 | ENSG00000163820 | ENST00000374694 |  |
| FZD1 | ENSG00000157240 | ENST00000509100 | (AG)3 |
| FZD5 | ENSG00000163251 | ENST00000340149 | (TG)3 |
| FZD8 | ENSG00000177283 | ENST00000368918 | (CT)3 |
| G3BP2 | ENSG00000138757 | ENST00000638159 | (GC)3 |
| GAB2 | ENSG00000033327 | ENST00000637827 | (AG)3 |
| GABPB2 | ENSG00000143458 | ENST00000522651 | (CA)3 |
| GABRA1 | ENSG00000022355 | ENST00000638099 | (AT)3 |
|  |  | ENST00000545868 | (GT)3 |
|  |  | ENST00000640757 | (AT)3 |
| GABRB3 | ENSG00000166206 | ENST00000638660 | (TA)3 |
|  |  | ENST00000638552 | (TG)3 |
| GABRG2 | ENSG00000113327 | ENST00000640574 | (GC)3 |
|  |  | ENST00000519196 | (CA)3 |
|  |  | ENST00000610680 |  |
|  |  |  |  |
| GABRP | ENSG00000094755 | ENST00000381700 | (AG)3 |
| GAGE1 | ENSG00000205777 | ENST00000407599 | (TG)3 |
|  |  |  |  |
|  |  | ENST00000420398 |  |
|  |  |  |  |
| GAGE10 | ENSG00000215274 | ENST00000405679 |  |
|  |  |  |  |
| GAGE12C | ENSG00000237671 | ENST00000639028 |  |
|  |  |  |  |
| GAGE12D | ENSG00000227488 | ENST00000445148 |  |
|  |  |  |  |
| GAGE12F | ENSG00000236362 | ENST00000381722 |  |
|  |  |  |  |
| GAGE12G | ENSG00000215269 | ENST00000442437 |  |
|  |  |  |  |
| GAGE12H | ENSG00000224902 | ENST00000612958 |  |
|  |  |  |  |
| GAGE12J | ENSG00000224659 | ENST00000621907 |  |
|  |  |  |  |
| GAGE13 | ENSG00000274274 | ENST00000411821 |  |
|  |  |  |  |
| GAGE2E | ENSG00000275113 | ENST00000591924  ENST00000252318 |  |
|  |  |  |  |
| GAL3ST1 | ENSG00000128242 | ENST00000397325 | (CT)3 |
| GALNT1 | ENSG00000141429 | ENST00000541995 | (CG)3 |
| GALNT8 | ENSG00000130035 | ENST00000538356 | (CT)3 |
| GALNT9 | ENSG00000182870 | ENST00000542942 | (TG)3 |
|  |  | ENST00000265956 |  |
|  |  | ENST00000381831 |  |
|  |  | ENST00000381839 |  |
| GAPVD1 | ENSG00000165219 | ENST00000426819 | (AC)3 |
| GART | ENSG00000159131 | ENST00000532398 |  |
|  |  | ENST00000266754 |  |
|  |  | ENST00000578599 |  |
| GAS2 | ENSG00000148935 | ENST00000584389 | (GA)3 |
| GAS2L3 | ENSG00000139354 | ENST00000269216 | (GCG)3 |
| GAS7 | ENSG00000007237 | ENST00000358713 | (AC)3 |
|  |  | ENST00000634544 | (GTT)3 |
| GATA6 | ENSG00000141448 | ENST00000378088 | (TC)3 |
| GATAD2A | ENSG00000167491 | ENST00000564665 | (GA)3 |
| GATAD2B | ENSG00000143614 | ENST00000370459 | (CG)3 |
| GBA2 | ENSG00000070610 | ENST00000446271 | (TG)3 |
| GBP3 | ENSG00000117226 | ENST00000559932 | (TC)3 |
| GBP5 | ENSG00000154451 | ENST00000559189 | (AT)3 |
| GCA | ENSG00000115271 | ENST00000560585 | (GA)3 |
| GCHFR | ENSG00000137880 | ENST00000396065 |  |
| GCNT3 | ENSG00000140297 | ENST00000559626 | (TCC)3 |
|  |  | ENST00000559200 |  |
|  |  | ENST00000243913 |  |
|  |  | ENST00000398355 |  |
|  |  | ENST00000588957 |  |
| GCNT7 | ENSG00000124091 | ENST00000253778 | (AT)3 |
| GEMIN8 | ENSG00000046647 | ENST00000518906 | (TC)3 |
| GFAP | ENSG00000131095 |  | (GAG)3 |
| GFPT2 | ENSG00000131459 | ENST00000391855 | (CGGAGCCCA)3 |
|  |  | ENST00000424414  ENST00000445650 | (AG)3 |
|  |  |  | (TC)3 |
| GGPS1 | ENSG00000152904 |  | (ATT)3 |
| GIGYF2 | ENSG00000204120 | ENST00000427649 | (AG)3 |
|  |  | ENST00000498181 | (CT)3 |
|  |  |  | (TG)3 |
|  |  | ENST00000358647 | (AG)3 |
| GIMAP5 | ENSG00000196329 | ENST00000587969 | (GA)3 |
|  |  |  |  |
|  |  | ENST00000447581  ENST00000525899 |  |
|  |  |  |  |
| GIPC1 | ENSG00000123159 | ENST00000590758 | (CT)3 |
| GJB1 | ENSG00000169562 | ENST00000587239 | (TG)3 |
| GJB7 | ENSG00000164411 | ENST00000559420 | (GA)3 |
| GJC1 | ENSG00000182963 | ENST00000639318 | (GC)3 |
|  |  | ENST00000640208 | (GAAG)3 |
| GLCE | ENSG00000138604 | ENST00000565474 | (AGC)3 |
| GLDC | ENSG00000178445 | ENST00000521682 | (TC)3 |
|  |  | ENST00000340042 |  |
| GLG1 | ENSG00000090863 | ENST00000509282 | (AG)3 |
| GLI4 | ENSG00000250571 | ENST00000394783 | (TCC)3 |
|  |  | ENST00000497953 |  |
| GLRB | ENSG00000109738 | ENST00000331872 | (GA)3 |
| GLT8D1 | ENSG00000016864 | ENST00000532258 | (GTG)3 |
|  |  | ENST00000523466 | (CT)3 |
| GLUL | ENSG00000135821 | ENST00000370069 | (CTCCT)3 |
| GLYATL2 | ENSG00000156689 | ENST00000553333 | (CAC)3 |
| GM2A | ENSG00000196743 | ENST00000558865 | (TG)3 |
| GMEB2 | ENSG00000101216 | ENST00000420554 | (TC)3 |
| GMFB | ENSG00000197045 | ENST00000557854 | (AT)3 |
| GMPR2 | ENSG00000100938 | ENST00000559104 | (TA)3 |
|  |  | ENST00000541118 | (AG)3 |
|  |  | ENST00000313949 |  |
|  |  | ENST00000371098 |  |
| GNA13 | ENSG00000120063 | ENST00000371075 | (GA)3 |
| GNAS | ENSG00000087460 | ENST00000604005 | (GT)3 |
|  |  | ENST00000371081 |  |
|  |  | ENST00000335281 |  |
|  |  | ENST00000366598 | (CGG)3 |
|  |  | ENST00000409696 | (CCG)3 |
| GNG2 | ENSG00000186469 | ENST00000610329 | (GGCTG)3 |
| GNG4 | ENSG00000168243 |  | (CG)3 |
| GNLY | ENSG00000115523 | ENST00000290438 | (CT)3 |
| GOLGA2 | ENSG00000167110 | ENST00000434739  ENST00000614055 | (GA)3 |
|  |  |  | (GAA)3 |
| GOLGA6A | ENSG00000159289 |  | (CA)3 |
| GOLGA6D | ENSG00000140478 | ENST00000619213 |  |
| GOLGA6L1 | ENSG00000273976 | ENST00000357743 |  |
|  |  |  | (TA)3 |
| GOLGA6L6 | ENSG00000277322 | ENST00000432566  ENST00000526619 | (CA)3 |
|  |  |  | (TA)3 |
| GOLGA7 | ENSG00000147533 |  | (CGG)3 |
| GOLGA8A | ENSG00000175265 | ENST00000542038 | (GA)3 |
| GOLGA8F | ENSG00000153684 | ENST00000416741  ENST00000638892 | (CA)3 |
|  |  |  | (GAA)3 |
| GOLT1B | ENSG00000111711 |  | (TTC)3 |
| GORASP1 | ENSG00000114745 | ENST00000638374 | (TCA)3 |
| GOSR2 | ENSG00000108433 | ENST00000640138  ENST00000639031 | (GA)3 |
|  |  |  | (AGA)3 |
|  |  | ENST00000329125 | (GA)3 |
|  |  | ENST00000401815 |  |
|  |  | ENST00000434632 |  |
| GP1BA | ENSG00000185245 | ENST00000514387 |  |
| GP5 | ENSG00000178732 | ENST00000420138 | (TG)3 |
| GPAT2 | ENSG00000186281 | ENST00000590375 | (CG)3 |
| GPBP1 | ENSG00000062194 | ENST00000515090 | (GT)3 |
| GPC1 | ENSG00000063660 | ENST00000512610 | (CCG)3 |
| GPI | ENSG00000105220 | ENST00000502754 | (AG)3 |
| GPM6A | ENSG00000150625 | ENST00000507520 | (GA)3 |
|  |  | ENST00000513667 | (CT)3 |
|  |  | ENST00000512509 |  |
|  |  | ENST00000505561 |  |
|  |  | ENST00000505375 |  |
|  |  | ENST00000513365 |  |
|  |  | ENST00000509865 |  |
|  |  | ENST00000512897 |  |
|  |  | ENST00000507540 | (TGG)3 |
|  |  | ENST00000505304 | (CT)3 |
|  |  | ENST00000539851 |  |
|  |  | ENST00000382315 |  |
|  |  | ENST00000299092 | (GA)3 |
| GPR137 | ENSG00000173264 | ENST00000561100 | (CTC)3 |
| GPR162 | ENSG00000250510 | ENST00000319838 | (GA)3 |
| GPR176 | ENSG00000166073 | ENST00000403859 | (GGA)3 |
|  |  | ENST00000407714 |  |
| GPR35 | ENSG00000178623 | ENST00000454971 | (TC)3 |
|  |  | ENST00000568214 |  |
|  |  | ENST00000582444 | (CA)3 |
| GPR42 | ENSG00000126251 | ENST00000609438 | (GA)3 |
| GPRC5B | ENSG00000167191 | ENST00000645164 | (TG)3 |
| GPRC5C | ENSG00000170412 | ENST00000375043 | (GCCCT)3 |
| GPRIN3 | ENSG00000185477 | ENST00000612794 | (AG)3 |
| GPSM2 | ENSG00000121957 | ENST00000389614 | (TAATT)3 |
| GPSM3 | ENSG00000213654 | ENST00000599564 | (TA)3 |
| GPX2 | ENSG00000176153 |  | (CT)3 |
|  |  | ENST00000424536 |  |
| GRAMD1A | ENSG00000089351 | ENST00000358160  ENST00000462838 | (TG)3 |
|  |  |  | (GAG)3 |
|  |  | ENST00000402578 | (GC)3 |
| GRAMD1C | ENSG00000178075 | ENST00000424693 | (CGGTG)3 |
|  |  | ENST00000395927 | (AC)3 |
| GRB10 | ENSG00000106070 | ENST00000393815 | (TC)3 |
| GRB14 | ENSG00000115290 |  | (TA)3 |
| GRHL2 | ENSG00000083307 |  | (GCT)3 |
| GRIA2 | ENSG00000120251 | ENST00000505888  ENST00000503437  ENST00000512631 | (GT)3 |
|  |  |  | (GA)3 |
|  |  |  |  |
|  |  |  | (AG)3 |
|  |  |  | (GA)3 |
|  |  | ENST00000452113 | (CG)3 |
|  |  | ENST00000389125 | (CA)3 |
| GRID2 | ENSG00000152208 | ENST00000399913 | (TG)3 |
| GRID2IP | ENSG00000215045 | ENST00000399914 | (ACC)3 |
| GRIK1 | ENSG00000171189 | ENST00000399907 | (TC)3 |
|  |  | ENST00000399909 |  |
|  |  | ENST00000438375 |  |
|  |  | ENST00000636406 |  |
|  |  | ENST00000454217 |  |
| GRIK4 | ENSG00000149403 | ENST00000374177 | (GCC)3 |
| GRIN2A | ENSG00000183454 | ENST00000544773 | (CG)3 |
| GRM3 | ENSG00000198822 | ENST00000639411 | (GC)3 |
| GRM4 | ENSG00000124493 | ENST00000398249 | (CT)3 |
|  |  | ENST00000476065 | (AC)3 |
| GRXCR2 | ENSG00000204928 | ENST00000612885 | (AG)3 |
| GSK3A | ENSG00000105723 | ENST00000627958 | (GAG)3 |
| GSTM1 | ENSG00000134184 | ENST00000432143 | (TC)3 |
| GSTT1 | ENSG00000277656 | ENST00000451013 | (GGTC)3 |
|  |  | ENST00000625377 |  |
| GTF2I | ENSG00000263001 | ENST00000472837 | (AC)3 |
| GTF2IRD2 | ENSG00000196275 | ENST00000619142 | (GCC)3 |
|  |  | ENST00000629105 |  |
| GTF2IRD2B | ENSG00000174428 | ENST00000614064 |  |
|  |  | ENST00000467752 |  |
|  |  | ENST00000473129 |  |
|  |  |  |  |
| GTPBP8 | ENSG00000163607 | ENST00000366726 | (AT)3 |
|  |  | ENST00000366722  ENST00000366716 | (GT)3 |
|  |  |  | (AC)3 |
| GUK1 | ENSG00000143774 | ENST00000355016 | (CT)3 |
|  |  | ENST00000586270 |  |
|  |  | ENST00000602477 |  |
| H2BFM | ENSG00000101812 | ENST00000542051 |  |
| H3F3B | ENSG00000132475 | ENST00000306560 | (GC)3 |
| H6PD | ENSG00000049239 | ENST00000593360 | (TG)3 |
| HABP2 | ENSG00000148702 | ENST00000320868 | (CTC)3 |
| HAS3 | ENSG00000103044 | ENST00000397797 | (GCAG)3 |
| HAUS8 | ENSG00000131351 | ENST00000251595 | (AAG)3 |
| HBA1 | ENSG00000206172 | ENST00000397806 | (AG)3 |
|  |  | ENST00000330597 | (GA)3 |
| HBA2 | ENSG00000188536 | ENST00000222574 | (AG)3 |
|  |  | ENST00000248089 |  |
| HBG1 | ENSG00000213934 | ENST00000574980 | (AC)3 |
| HBP1 | ENSG00000105856 | ENST00000354679 | (CG)3 |
| HCFC1R1 | ENSG00000103145 | ENST00000519108 | (CT)3 |
|  |  |  |  |
|  |  | ENST00000430454 |  |
| HDAC2 | ENSG00000196591 | ENST00000506585  ENST00000373177 | (TGG)3 |
|  |  |  | (TC)3 |
| HDAC9 | ENSG00000048052 | ENST00000631422 | (AT)3 |
| HDX | ENSG00000165259 | ENST00000372168 |  |
|  |  | ENST00000311127 |  |
| HECTD2 | ENSG00000165338 | ENST00000239026 | (ACC)3 |
| HECTD3 | ENSG00000126107 | ENST00000371332 | (TG)3 |
| HEG1 | ENSG00000173706 | ENST00000519389 | (CCGCTGC)3 |
| HELLS | ENSG00000119969 | ENST00000569429 | (AGA)3 |
|  |  | ENST00000563343 |  |
| HEPH | ENSG00000089472 | ENST00000509579 | (TCCT)3 |
| HERPUD1 | ENSG00000051108 | ENST00000571518 | (CA)3 |
|  |  | ENST00000577012 |  |
| HEXB | ENSG00000049860 | ENST00000367010 | (GA)3 |
| HGS | ENSG00000185359 | ENST00000416756 | (AC)3 |
|  |  | ENST00000467562 |  |
| HHAT | ENSG00000054392 | ENST00000482430 | (GCCT)3 |
| HHATL | ENSG00000010282 | ENST00000467761 | (AC)3 |
| HHLA2 | ENSG00000114455 | ENST00000361764 | (GA)3 |
|  |  | ENST00000359875 |  |
|  |  | ENST00000370940 |  |
| HHLA3 | ENSG00000197568 | ENST00000530678 | (GT)3 |
|  |  | ENST00000634733 |  |
|  |  | ENST00000590166 |  |
| HINFP | ENSG00000172273 | ENST00000229829 | (GAG)3 |
| HIST1H3E | ENSG00000274750 | ENST00000399084 | (TA)3 |
| HKR1 | ENSG00000181666 | ENST00000442944 | (GT)3 |
| HLA-DOA | ENSG00000204252 | ENST00000611173 | (CTT)3 |
| HLA-DQB1 | ENSG00000179344 | ENST00000416526 | (TC)3 |
| HMBS | ENSG00000256269 | ENST00000399494 | (CT)3 |
| HMCN2 | ENSG00000148357 | ENST00000519684 | (TG)3 |
| HMG20B | ENSG00000064961 | ENST00000522796 | (GC)3 |
| HMGB1 | ENSG00000189403 | ENST00000511206 | (GGCG)3 |
| HMGB4 | ENSG00000176256 | ENST00000316673 | (TC)3 |
|  |  | ENST00000336053 |  |
| HMGCR | ENSG00000113161 | ENST00000556142 | (CA)3 |
| HNF4A | ENSG00000101076 | ENST00000555883 | (TGC)3 |
| HNRNPC | ENSG00000092199 | ENST00000557201 | (CAG)3 |
|  |  | ENST00000515432 |  |
|  |  | ENST00000505811 |  |
|  |  | ENST00000503664 |  |
| HNRNPD | ENSG00000138668 | ENST00000601336 | (GCT)3 |
| HNRNPH1 | ENSG00000169045 | ENST00000593587 | (GC)3 |
|  |  | ENST00000596482 |  |
| HNRNPUL1 | ENSG00000105323 | ENST00000588052 | (AG)3 |
|  |  | ENST00000503639 |  |
| HOMER3 | ENSG00000051128 | ENST00000555760 | (CGG)3 |
| HOOK2 | ENSG00000095066 | ENST00000509328 | (GA)3 |
| HOPX | ENSG00000171476 | ENST00000508190 | (CCG)3 |
|  |  | ENST00000396253 | (CG)3 |
| HOXC6 | ENSG00000197757 | ENST00000438420 | (TC)3 |
| HOXC9 | ENSG00000180806 | ENST00000544218 | (CA)3 |
| HPS5 | ENSG00000110756 | ENST00000531848 | (ATG)3 |
|  |  | ENST00000354323 |  |
|  |  | ENST00000591456 | (AGA)3 |
|  |  | ENST00000578576 | (ATG)3 |
| HRCT1 | ENSG00000196196 | ENST00000577917 | (AG)3 |
| HS2ST1 | ENSG00000153936 | ENST00000301382 | (GAG)3 |
| HS3ST3A1 | ENSG00000153976 | ENST00000423665 | (CTT)3 |
| HSD11B1L | ENSG00000167733 | ENST00000422535 | (CA)3 |
|  |  | ENST00000339423 |  |
|  |  | ENST00000579559 |  |
|  |  | ENST00000375263 |  |
|  |  | ENST00000375262 |  |
|  |  | ENST00000620073 |  |
| HSD17B3 | ENSG00000130948 |  | (AG)3 |
|  |  | ENST00000549334 |  |
| HSP90AB1 | ENSG00000096384 | ENST00000394709  ENST00000534567 | (TG)3 |
|  |  |  | (TC)3 |
| HSP90B1 | ENSG00000166598 | ENST00000524590 | (AG)3 |
| HSPA2 | ENSG00000126803 | ENST00000374695 | (CT)3 |
| HSPA8 | ENSG00000109971 |  | (CG)3 |
|  |  | ENST00000451739 | (CA)3 |
| HSPG2 | ENSG00000142798 | ENST00000548312  ENST00000242607 | (GAGC)3 |
|  |  |  | (AG)3 |
| HTATIP2 | ENSG00000109854 | ENST00000439744 | (TCC)3 |
| HVCN1 | ENSG00000122986 | ENST00000549442 | (CA)3 |
|  |  | ENST00000546713 |  |
|  |  | ENST00000539973 |  |
|  |  | ENST00000450143 |  |
|  |  | ENST00000589261 |  |
| HYDIN | ENSG00000157423 | ENST00000590569 |  |
| ICA1L | ENSG00000163596 | ENST00000589249 | (CG)3 |
| ICAM3 | ENSG00000076662 | ENST00000380770 | (CT)3 |
|  |  | ENST00000340992 | (CCT)3 |
|  |  | ENST00000559186 | (CT)3 |
| ICAM4 | ENSG00000105371 | ENST00000427898 |  |
|  |  | ENST00000277517 |  |
| IDH3A | ENSG00000166411 | ENST00000292433 | (GGAAA)3 |
| IDI1 | ENSG00000067064 | ENST00000588173 | (AC)3 |
| IDI2 | ENSG00000148377 | ENST00000372491 | (AG)3 |
| IER2 | ENSG00000160888 |  |  |
|  |  |  |  |
| IER5L | ENSG00000188483 | ENST00000555523  ENST00000554166 | (CAG)3 |
|  |  |  | (GGC)3 |
|  |  |  | (CCG)3 |
| IFI27L1 | ENSG00000165948 | ENST00000556381 | (TG)3 |
|  |  |  | (GA)3 |
|  |  | ENST00000553664 | (AG)3 |
|  |  |  | (GA)3 |
|  |  | ENST00000555341 | (AG)3 |
|  |  |  | (GA)3 |
|  |  | ENST00000557218 | (TG)3 |
|  |  |  | (GA)3 |
|  |  | ENST00000554544 | (AG)3 |
|  |  |  | (GA)3 |
|  |  | ENST00000528780  ENST00000476927 | (TG)3 |
|  |  |  | (GA)3 |
|  |  | ENST00000498409 |  |
| IFITM1 | ENSG00000185885 | ENST00000558354 |  |
| IFRD1 | ENSG00000006652 |  | (TC)3 |
| IFT80 | ENSG00000068885 | ENST00000352385 | (CAG)3 |
| IGDCC3 | ENSG00000174498 | ENST00000377693  ENST00000335211 | (TC)3 |
|  |  |  | (CA)3 |
| IGDCC4 | ENSG00000103742 | ENST00000295591 | (GC)3 |
| IGFL2 | ENSG00000204866 | ENST00000425327 | (CTG)3 |
| IGFN1 | ENSG00000163395 |  | (AGC)3 |
|  |  | ENST00000448417 |  |
| IGSF11 | ENSG00000144847 | ENST00000440286  ENST00000413620 | (GT)3 |
|  |  |  | (TC)3 |
| IGSF8 | ENSG00000162729 | ENST00000615186 | (GGCGG)3 |
| IKBKG | ENSG00000269335 | ENST00000547167 | (ACCG)3 |
|  |  | ENST00000547791 | (TG)3 |
|  |  | ENST00000471071 | (CAG)3 |
| IKZF4 | ENSG00000123411 | ENST00000556792 | (GCT)3 |
|  |  | ENST00000318041 |  |
| IL10 | ENSG00000136634 | ENST00000525219 | (TC)3 |
| IL11RA | ENSG00000137070 | ENST00000340758 | (CTG)3 |
|  |  | ENST00000452403 |  |
| IL15RA | ENSG00000134470 | ENST00000428279 | (CT)3 |
| IL19 | ENSG00000142224 | ENST00000412504 | (CA)3 |
| IL1R1 | ENSG00000115594 | ENST00000443369 | (GT)3 |
|  |  | ENST00000264257 | (TG)3 |
| IL1RAP | ENSG00000196083 | ENST00000367748 | (TTA)3 |
|  |  | ENST00000468393 |  |
| IL1RL2 | ENSG00000115598 | ENST00000445595 | (GC)3 |
| IL20RA | ENSG00000016402 | ENST00000331035 | (GCT)3 |
|  |  | ENST00000381469 |  |
| IL2RB | ENSG00000100385 | ENST00000566117 | (CA)3 |
| IL3RA | ENSG00000185291 | ENST00000508941 |  |
|  |  | ENST00000587941 |  |
| IL4R | ENSG00000077238 |  | (CG)3 |
| IL7R | ENSG00000168685 | ENST00000534378 | (GT)3 |
| ILF3 | ENSG00000129351 | ENST00000600984  ENST00000529749 | (AC)3 |
|  |  |  | (CT)3 |
| ILVBL | ENSG00000105135 | ENST00000530023 | (AC)3 |
|  |  |  |  |
| IMMP1L | ENSG00000148950 | ENST00000405709 | (TG)3 |
|  |  | ENST00000314852  ENST00000403490 |  |
|  |  |  | (GT)3 |
| IMMP2L | ENSG00000184903 | ENST00000496200 | (TGTC)3 |
| IMP3 | ENSG00000177971 | ENST00000626419 | (CG)3 |
|  |  | ENST00000470772 |  |
| IMPDH1 | ENSG00000106348 | ENST00000480861 |  |
|  |  | ENST00000586489 | (AC)3 |
|  |  | ENST00000409027 | (CG)3 |
|  |  | ENST00000506200 |  |
| INO80C | ENSG00000153391 | ENST00000460070 | (GA)3 |
| INPP1 | ENSG00000151689 | ENST00000544829 | (GTG)3 |
| IPO11 | ENSG00000086200 | ENST00000460108 | (AG)3 |
| IPO5 | ENSG00000065150 | ENST00000623361 | (CA)3 |
| IPO8 | ENSG00000133704 | ENST00000325979 | (AT)3 |
| IQCB1 | ENSG00000173226 | ENST00000423395 | (TC)3 |
| IQCE | ENSG00000106012 | ENST00000422276 |  |
|  |  | ENST00000423196 |  |
|  |  | ENST00000629425 |  |
|  |  | ENST00000639631 |  |
|  |  | ENST00000639298 | (TG)3 |
| IQCH | ENSG00000103599 | ENST00000514350 | (GA)3 |
| IQCJ-SCHIP1 | ENSG00000283154 | ENST00000646269 | (ACA)3 |
|  |  |  |  |
| IQGAP2 | ENSG00000145703 | ENST00000618604 | (TC)3 |
| IQSEC1 | ENSG00000144711 | ENST00000607739  ENST00000550616 | (AG)3 |
|  |  |  | (GTG)3 |
|  |  | ENST00000302165 | (GAG)3 |
| IRAK1BP1 | ENSG00000146243 | ENST00000366610 | (CT)3 |
| IRAK4 | ENSG00000198001 | ENST00000366609 |  |
| IRF2BP1 | ENSG00000170604 | ENST00000525445 | (GC)3 |
| IRF2BP2 | ENSG00000168264 | ENST00000305123 | (CTC)3 |
|  |  | ENST00000338128 |  |
| IRF7 | ENSG00000185507 | ENST00000457269 | (GC)3 |
| IRS1 | ENSG00000169047 | ENST00000578352 | (TGG)3 |
| ISYNA1 | ENSG00000105655 | ENST00000581800 | (CCG)3 |
|  |  | ENST00000535650 |  |
|  |  | ENST00000557257 |  |
|  |  | ENST00000528877 |  |
| ITCH | ENSG00000078747 | ENST00000417122 | (GT)3 |
| ITGA7 | ENSG00000135424 | ENST00000414670 | (GA)3 |
| ITGB1 | ENSG00000150093 | ENST00000523663 | (CA)3 |
|  |  |  | (TC)3 |
|  |  |  | (AG)3 |
| ITGB2 | ENSG00000160255 | ENST00000422257  ENST00000409704  ENST00000556603 | (TG)3 |
|  |  |  |  |
|  |  |  |  |
| ITGB7 | ENSG00000139626 | ENST00000557309 | (TGC)3 |
| ITM2C | ENSG00000135916 | ENST00000358187 | (GAG)3 |
| ITPK1 | ENSG00000100605 | ENST00000439118 | (CGC)3 |
|  |  | ENST00000331782 | (AC)3 |
| ITPRIP | ENSG00000148841 | ENST00000347004 | (TG)3 |
| ITPRIPL1 | ENSG00000198885 | ENST00000527670 | (AG)3 |
| JAG2 | ENSG00000184916 | ENST00000393930 | (CGGG)3 |
|  |  | ENST00000619269 |  |
| JAK3 | ENSG00000105639 | ENST00000317477 | (TC)3 |
| JUP | ENSG00000173801 | ENST00000562592 | (CT)3 |
| KANK1 | ENSG00000107104 | ENST00000320248 | (CTC)3 |
| KANK4 | ENSG00000132854 | ENST00000382545 | (GCT)3 |
| KATNB1 | ENSG00000140854 | ENST00000369771 |  |
| KBTBD11 | ENSG00000176595 | ENST00000639048 | (CG)3 |
| KCNA1 | ENSG00000111262 | ENST00000640774 |  |
| KCNA10 | ENSG00000143105 | ENST00000639233 | (CTT)3 |
| KCNA2 | ENSG00000177301 | ENST00000638477 | (AG)3 |
|  |  | ENST00000369769 |  |
|  |  | ENST00000252321 |  |
|  |  | ENST00000378111 |  |
| KCNA3 | ENSG00000177272 | ENST00000290310 | (GAG)3 |
| KCNA5 | ENSG00000130037 | ENST00000332142 | (AG)3 |
| KCNAB2 | ENSG00000069424 |  | (AC)3 |
| KCNE2 | ENSG00000159197 | ENST00000328032 | (TG)3 |
| KCNH7 | ENSG00000184611 | ENST00000509207 | (CG)3 |
|  |  |  | (GA)3 |
|  |  | ENST00000638868  ENST00000338350 | (CG)3 |
|  |  |  | (GA)3 |
| KCNIP4 | ENSG00000185774 | ENST00000312350 | (GT)3 |
| KCNJ10 | ENSG00000177807 |  | (AG)3 |
| KCNJ5 | ENSG00000120457 | ENST00000507712 | (TG)3 |
| KCNK10 | ENSG00000100433 | ENST00000637193  ENST00000638588 | (GA)3 |
|  |  |  | (AC)3 |
| KCNK16 | ENSG00000095981 | ENST00000263604 | (AG)3 |
| KCNQ2 | ENSG00000075043 | ENST00000333751 | (GCT)3 |
| KCNQ3 | ENSG00000184156 | ENST00000377474 | (TG)3 |
| KCNT1 | ENSG00000107147 | ENST00000483557 | (GCCCGCC)3 |
| KCTD11 | ENSG00000213859 | ENST00000530018 | (CA)3 |
| KCTD12 | ENSG00000178695 | ENST00000355076 | (GCG)3 |
| KCTD20 | ENSG00000112078 | ENST00000588337 | (TA)3 |
| KCTD21 | ENSG00000188997 | ENST00000433730 | (CT)3 |
| KCTD6 | ENSG00000168301 | ENST00000373266 | (TA)3 |
| KDM4B | ENSG00000127663 | ENST00000588470 | (GC)3 |
| KHDC1 | ENSG00000135314 | ENST00000354386 |  |
| KIAA0319L | ENSG00000142687 | ENST00000619722 | (AG)3 |
| KIAA0355 | ENSG00000166398 | ENST00000619416 | (CA)3 |
| KIAA0586 | ENSG00000100578 | ENST00000423743 | (AG)3 |
|  |  | ENST00000572370 |  |
|  |  | ENST00000453212 |  |
|  |  | ENST00000290881 |  |
| KIAA0753 | ENSG00000198920 | ENST00000533147 | (AT)3 |
| KIAA0895 | ENSG00000164542 |  | (GT)3 |
| KIAA0895L | ENSG00000196123 | ENST00000531664 | (AG)3 |
| KIAA1324 | ENSG00000116299 | ENST00000534476  ENST00000637069 | (CG)3 |
|  |  |  |  |
|  |  | ENST00000612149 | (GC)3 |
|  |  |  |  |
| KIAA1671 | ENSG00000197077 | ENST00000425755 | (CT)3 |
| KIAA1841 | ENSG00000162929 | ENST00000394362  ENST00000229913 | (GA)3 |
|  |  |  | (AT)3 |
| KIF15 | ENSG00000163808 | ENST00000538893 | (AC)3 |
| KIF6 | ENSG00000164627 |  | (TC)3 |
|  |  | ENST00000394065 |  |
|  |  | ENST00000421199  ENST00000446344 | (GAA)3 |
|  |  |  | (AG)3 |
| KLC2 | ENSG00000174996 | ENST00000460687 | (GAG)3 |
| KLF7 | ENSG00000118263 | ENST00000367258 | (TC)3 |
| KLHDC4 | ENSG00000104731 | ENST00000622660 | (AG)3 |
| KLHDC8A | ENSG00000162873 | ENST00000431430 | (GT)3 |
| KLHL12 | ENSG00000117153 | ENST00000337975 | (TA)3 |
| KLHL17 | ENSG00000187961 | ENST00000556500 | (GAG)3 |
| KLHL22 | ENSG00000099910 | ENST00000557468 | (TCCCGCC)3 |
| KLHL25 | ENSG00000183655 | ENST00000376292 | (CG)3 |
| KLHL28 | ENSG00000179454 |  | (CT)3 |
|  |  | ENST00000598673 | (ATA)3 |
| KLHL35 | ENSG00000149243 | ENST00000536833  ENST00000482287 | (GC)3 |
|  |  |  |  |
| KLK15 | ENSG00000174562 | ENST00000261667 | (CT)3 |
| KLRC2 | ENSG00000205809 | ENST00000590425 | (AC)3 |
| KPNA1 | ENSG00000114030 | ENST00000246639 | (GGCGG)3 |
| KPNA3 | ENSG00000102753 | ENST00000546897 | (TC)3 |
| KRT13 | ENSG00000171401 | ENST00000548998 | (GA)3 |
| KRT35 | ENSG00000197079 | ENST00000619952 | (GT)3 |
| KRT8 | ENSG00000170421 | ENST00000293525 | (GCT)3 |
|  |  | ENST00000391621 |  |
|  |  | ENST00000397911 |  |
| KRT86 | ENSG00000170442 | ENST00000335093 | (CT)3 |
| KRTAP10-2 | ENSG00000205445 | ENST00000333822 | (TCAC)3 |
| KRTAP10-9 | ENSG00000221837 | ENST00000391415 | (CA)3 |
| KRTAP21-1 | ENSG00000187005 | ENST00000528743 | (TC)3 |
| KRTAP4-8 | ENSG00000204880 | ENST00000554507 | (CT)3 |
| KRTAP4-9 | ENSG00000212722 | ENST00000554890 |  |
| KRTAP5-9 | ENSG00000254997 | ENST00000553624 | (GT)3 |
| KTN1 | ENSG00000126777 | ENST00000583809 | (GA)3 |
|  |  | ENST00000305544 |  |
|  |  | ENST00000550522 |  |
| L3MBTL4 | ENSG00000154655 | ENST00000548697 | (CG)3 |
| LAMB2 | ENSG00000172037 | ENST00000548993 | (AGG)3 |
| LARP4 | ENSG00000161813 | ENST00000614335 | (AT)3 |
|  |  | ENST00000615080 |  |
|  |  | ENST00000523389 |  |
|  |  | ENST00000518561 |  |
|  |  | ENST00000517559 |  |
|  |  | ENST00000552445 |  |
|  |  | ENST00000507443 |  |
|  |  | ENST00000367217 |  |
|  |  | ENST00000407930 |  |
| LARP7 | ENSG00000174720 | ENST00000460508 | (TC)3 |
| LAX1 | ENSG00000122188 | ENST00000341396 | (CT)3 |
| LBH | ENSG00000213626 | ENST00000438404 | (GC)3 |
| LBX2 | ENSG00000179528 | ENST00000368789 | (TC)3 |
|  |  | ENST00000368777 |  |
| LCA5L | ENSG00000157578 |  | (GA)3 |
| LCE3E | ENSG00000185966 | ENST00000497771 | (CT)3 |
| LCE4A | ENSG00000187170 | ENST00000527229  ENST00000396076 | (GT)3 |
|  |  |  | (CTG)3 |
| LCN10 | ENSG00000187922 | ENST00000547660 | (AG)3 |
|  |  | ENST00000593425 |  |
| LDHB | ENSG00000111716 | ENST00000612127 | (GA)3 |
| LETMD1 | ENSG00000050426 | ENST00000610305 |  |
| LIG1 | ENSG00000105486 | ENST00000427581 | (GCA)3 |
| LILRA3 | ENSG00000278046 | ENST00000434421 | (TG)3 |
|  |  | ENST00000391750 |  |
| LILRB1 | ENSG00000104972 | ENST00000583211 |  |
| LILRB2 | ENSG00000131042 | ENST00000582055 | (AC)3 |
| LILRB3 | ENSG00000204577 | ENST00000544547 | (GA)3 |
| LIMD2 | ENSG00000136490 | ENST00000428064 | (GGC)3 |
|  |  | ENST00000409455 |  |
| LIMS1 | ENSG00000169756 | ENST00000409808 | (CGG)3 |
|  |  | ENST00000446851 |  |
| LIMS2 | ENSG00000072163 | ENST00000371939 | (AGC)3 |
|  |  | ENST00000393471 |  |
| LIN54 | ENSG00000189308 | ENST00000579392 | (TGG)3 |
| LIPJ | ENSG00000204022 |  | (AG)3 |
| LIPT1 | ENSG00000144182 | ENST00000583658 | (CG)3 |
| LLGL2 | ENSG00000073350 | ENST00000562810 | (TG)3 |
|  |  |  | (GCT)3 |
|  |  | ENST00000600059 | (GGT)3 |
| LMAN1L | ENSG00000140506 | ENST00000585374  ENST00000540670 | (CA)3 |
|  |  |  |  |
| LMTK3 | ENSG00000142235 | ENST00000368742 | (CG)3 |
| LONP1 | ENSG00000196365 | ENST00000579038 | (GCC)3 |
|  |  | ENST00000414184 | (GA)3 |
| LOR | ENSG00000203782 | ENST00000524144 | (TC)3 |
| LOXHD1 | ENSG00000167210 |  | (CA)3 |
|  |  | ENST00000520871 |  |
| LOXL2 | ENSG00000134013 | ENST00000316300 | (GT)3 |
|  |  |  | (GA)3 |
|  |  | ENST00000592061  ENST00000256720 | (GT)3 |
|  |  |  | (GA)3 |
| LPA | ENSG00000198670 | ENST00000528489 | (AC)3 |
| LPAR2 | ENSG00000064547 | ENST00000497245 | (CA)3 |
| LPIN1 | ENSG00000134324 | ENST00000536173 | (CGG)3 |
| LPXN | ENSG00000110031 | ENST00000523007 | (GT)3 |
| LRCH4 | ENSG00000077454 | ENST00000510776 | (CCG)3 |
| LRMP | ENSG00000118308 |  | (GT)3 |
| LRP12 | ENSG00000147650 | ENST00000610821 | (AGC)3 |
| LRP2BP | ENSG00000109771 | ENST00000402859 | (AG)3 |
|  |  |  | (TG)3 |
| LRP5L | ENSG00000100068 | ENST00000444995 | (CTG)3 |
|  |  |  | (AC)3 |
|  |  | ENST00000402785 | (CTG)3 |
|  |  |  | (AC)3 |
|  |  | ENST00000535731  ENST00000530854 | (CTG)3 |
|  |  |  | (AC)3 |
|  |  | ENST00000347624 |  |
| LRP6 | ENSG00000070018 | ENST00000331450 | (TG)3 |
| LRRC14 | ENSG00000160959 | ENST00000569499 | (GCA)3 |
| LRRC15 | ENSG00000172061 | ENST00000568804 | (GGCG)3 |
| LRRC28 | ENSG00000168904 | ENST00000563189 | (TCC)3 |
| LRRC36 | ENSG00000159708 | ENST00000334962 | (GA)3 |
|  |  | ENST00000560691 |  |
|  |  | ENST00000558456 | (AC)3 |
| LRRC37A3 | ENSG00000176809 | ENST00000560158 | (CT)3 |
| LRRC49 | ENSG00000137821 |  | (AT)3 |
|  |  | ENST00000558808 |  |
|  |  | ENST00000559806  ENST00000559069 |  |
|  |  |  | (GAA)3 |
|  |  | ENST00000446175 | (AT)3 |
|  |  | ENST00000432635 |  |
|  |  | ENST00000529371 |  |
| LRRC63 | ENSG00000173988 | ENST00000228918 | (TG)3 |
| LTBP1 | ENSG00000049323 | ENST00000536876 | (CAC)3 |
| LTBP3 | ENSG00000168056 | ENST00000619796 | (CG)3 |
| LTBR | ENSG00000111321 | ENST00000371921 | (GCC)3 |
|  |  | ENST00000594013 |  |
| LUC7L2 | ENSG00000146963 | ENST00000414420 | (GC)3 |
| LUZP4 | ENSG00000102021 | ENST00000420982 | (GT)3 |
| LYPD5 | ENSG00000159871 | ENST00000421070 | (CT)3 |
| LYPD6 | ENSG00000187123 | ENST00000374501 | (TG)3 |
| LYPLA2 | ENSG00000011009 | ENST00000219168 |  |
|  |  | ENST00000500869 |  |
|  |  | ENST00000409796 | (CT)3 |
| LYRM1 | ENSG00000102897 | ENST00000481129 | (TCCC)3 |
| LYSMD3 | ENSG00000176018 | ENST00000484793 | (CT)3 |
| LYSMD4 | ENSG00000183060 | ENST00000587541 | (CG)3 |
| LZTS2 | ENSG00000107816 | ENST00000422579 | (TG)3 |
| MACF1 | ENSG00000127603 | ENST00000505839 | (AG)3 |
| MADCAM1 | ENSG00000099866 | ENST00000357736 | (GA)3 |
| MADD | ENSG00000110514 | ENST00000416020 |  |
| MAEA | ENSG00000090316 |  | (AG)3 |
| MAFG | ENSG00000197063 | ENST00000602297 | (GCCCC)3 |
| MAGEA4 | ENSG00000147381 | ENST00000285879  ENST00000443323 | (TC)3 |
|  |  |  |  |
| MAGEB5 | ENSG00000188408 | ENST00000544766 | (CTG)3 |
| MAGEC1 | ENSG00000155495 | ENST00000361470 | (AGA)3 |
| MAGEC3 | ENSG00000165509 | ENST00000354212 |  |
|  |  | ENST00000419488 |  |
| MAGEE1 | ENSG00000198934 | ENST00000637441 | (CT)3 |
| MAGI2 | ENSG00000187391 | ENST00000522391 | (TC)3 |
|  |  | ENST00000644159 |  |
|  |  | ENST00000226578 |  |
|  |  | ENST00000647097 |  |
| MANBA | ENSG00000109323 | ENST00000642252 |  |
|  |  | ENST00000505239 |  |
|  |  | ENST00000329006 |  |
|  |  | ENST00000354498 |  |
|  |  | ENST00000420772 |  |
| MANEAL | ENSG00000185090 |  | (TG)3 |
| MAP2K5 | ENSG00000137764 | ENST00000589130 | (GA)3 |
| MAP4 | ENSG00000047849 | ENST00000427603  ENST00000555216 | (TG)3 |
|  |  |  | (GA)3 |
| MAP4K1 | ENSG00000104814 | ENST00000429533 | (GC)3 |
| MAP4K4 | ENSG00000071054 | ENST00000530729 | (CA)3 |
| MAP4K5 | ENSG00000012983 |  | (AC)3 |
| MAP7D1 | ENSG00000116871 | ENST00000311277 | (ACC)3 |
|  |  | ENST00000515654  ENST00000433024 | (CT)3 |
|  |  |  | (ACC)3 |
| MAP9 | ENSG00000164114 | ENST00000641341 | (CTC)3 |
|  |  | ENST00000641217 |  |
|  |  | ENST00000641647 |  |
| MAPK10 | ENSG00000109339 | ENST00000642006 | (GA)3 |
|  |  | ENST00000641047 | (AG)3 |
|  |  | ENST00000449047 | (GA)3 |
|  |  | ENST00000641607 | (AG)3 |
|  |  | ENST00000641762 |  |
|  |  | ENST00000641862 |  |
|  |  | ENST00000515400 |  |
|  |  | ENST00000641072 | (GA)3 |
|  |  | ENST00000641873 | (AG)3 |
|  |  | ENST00000642019 | (CT)3 |
|  |  | ENST00000641629 | (AG)3 |
|  |  | ENST00000511328 |  |
|  |  | ENST00000639989 |  |
|  |  | ENST00000641061 | (CT)3 |
|  |  | ENST00000641116 |  |
|  |  | ENST00000641675 |  |
|  |  | ENST00000589699 | (GA)3 |
|  |  |  | (AG)3 |
|  |  | ENST00000543220 |  |
| MAPRE2 | ENSG00000166974 | ENST00000567501  ENST00000439271 | (CG)3 |
|  |  |  | (TA)3 |
| MARK2 | ENSG00000072518 |  | (CA)3 |
| MARVELD3 | ENSG00000140832 | ENST00000395045 | (GA)3 |
| MASP1 | ENSG00000127241 | ENST00000590980  ENST00000522025 |  |
|  |  |  | (AG)3 |
| MATK | ENSG00000007264 | ENST00000519585 | (GA)3 |
|  |  |  |  |
| MATN2 | ENSG00000132561 | ENST00000562557 | (TG)3 |
|  |  | ENST00000568544 | (GGT)3 |
|  |  |  | (TG)3 |
| MAZ | ENSG00000103495 | ENST00000569978 | (CA)3 |
|  |  |  | (GT)3 |
|  |  | ENST00000568282 | (CA)3 |
|  |  |  | (GT)3 |
|  |  | ENST00000359787 | (CA)3 |
|  |  |  | (GT)3 |
|  |  | ENST00000443033  ENST00000587605 | (CA)3 |
|  |  |  | (GT)3 |
| MB | ENSG00000198125 | ENST00000546632 | (TG)3 |
|  |  | ENST00000493459 |  |
| MBD1 | ENSG00000141644 | ENST00000459747 | (CG)3 |
| MBD6 | ENSG00000166987 | ENST00000581878 | (CT)3 |
| MBNL1 | ENSG00000152601 | ENST00000589410 | (AAG)3 |
|  |  | ENST00000629669 |  |
| MBP | ENSG00000197971 | ENST00000414978 | (GT)3 |
| MC5R | ENSG00000176136 | ENST00000519895 | (GAG)3 |
| MCCC1 | ENSG00000078070 | ENST00000409954 | (GGC)3 |
| MCF2 | ENSG00000101977 | ENST00000482017 | (AG)3 |
|  |  | ENST00000444761 |  |
| MCF2L | ENSG00000126217 | ENST00000409105 | (GT)3 |
| MCF2L2 | ENSG00000053524 | ENST00000409207 | (GC)3 |
| MCFD2 | ENSG00000180398 | ENST00000409147 | (CCG)3 |
|  |  | ENST00000479669 | (AT)3 |
|  |  |  | (CCG)3 |
|  |  | ENST00000515437 |  |
| MCM10 | ENSG00000065328 | ENST00000486952  ENST00000449792 | (GA)3 |
|  |  |  | (CT)3 |
| MDGA1 | ENSG00000112139 | ENST00000461263 | (TG)3 |
| MDGA2 | ENSG00000139915 | ENST00000315758 |  |
| MDH1B | ENSG00000138400 | ENST00000541686 | (GA)3 |
| MDH2 | ENSG00000146701 | ENST00000494292 | (AG)3 |
|  |  | ENST00000406969 |  |
| MDM1 | ENSG00000111554 | ENST00000428629 | (GT)3 |
| MECOM | ENSG00000085276 | ENST00000424287 | (CA)3 |
| MED15 | ENSG00000099917 | ENST00000494177 | (CGG)3 |
|  |  | ENST00000617849 | (CT)3 |
|  |  | ENST00000620467 |  |
| MED22 | ENSG00000148297 | ENST00000622402 | (AG)3 |
| MED25 | ENSG00000104973 | ENST00000612854 | (GTG)3 |
|  |  | ENST00000312865 |  |
|  |  | ENST00000601478 |  |
|  |  | ENST00000420343 |  |
|  |  | ENST00000409447 |  |
| MED26 | ENSG00000105085 | ENST00000455812 | (AG)3 |
| MED7 | ENSG00000155868 | ENST00000398506 | (TG)3 |
| MEF2B | ENSG00000213999 | ENST00000397624 | (ATC)3 |
| MEGF11 | ENSG00000157890 | ENST00000397620 | (GT)3 |
| MEIS1 | ENSG00000143995 | ENST00000558313 | (TA)3 |
| MEIS2 | ENSG00000134138 | ENST00000536987 | (CA)3 |
|  |  | ENST00000433159 |  |
|  |  | ENST00000570778 |  |
| MELK | ENSG00000165304 | ENST00000571814 | (AT)3 |
| MEST | ENSG00000106484 | ENST00000616852 | (TCC)3 |
| METRNL | ENSG00000176845 |  | (CA)3 |
|  |  | ENST00000480046 |  |
| METTL2A | ENSG00000087995 | ENST00000392640  ENST00000612742 | (GA)3 |
|  |  |  | (GC)3 |
| METTL2B | ENSG00000165055 | ENST00000442778 |  |
| METTL5 | ENSG00000138382 | ENST00000452930 |  |
| METTL8 | ENSG00000123600 | ENST00000263969 | (CG)3 |
|  |  | ENST00000497673 |  |
| MFF | ENSG00000168958 | ENST00000305386 | (CGC)3 |
| MFN1 | ENSG00000171109 |  | (GT)3 |
| MGAM | ENSG00000257335 | ENST00000428789 | (GA)3 |
| MGAT2 | ENSG00000168282 | ENST00000263369 | (GCC)3 |
|  |  |  | (CGCC)3 |
| MGAT5B | ENSG00000167889 | ENST00000340535 | (CT)3 |
| MIA | ENSG00000261857 | ENST00000616296  ENST00000358577 | (AG)3 |
|  |  |  | (CT)3 |
| MIA3 | ENSG00000154305 | ENST00000358807 | (GGA)3 |
| MICA | ENSG00000204520 | ENST00000440000 | (AG)3 |
| MICAL1 | ENSG00000135596 | ENST00000407029 | (GCC)3 |
|  |  | ENST00000402630 |  |
| MIER3 | ENSG00000155545 | ENST00000381476 | (AG)3 |
| MKL1 | ENSG00000196588 | ENST00000629733 | (TC)3 |
|  |  | ENST00000366272 |  |
| MLANA | ENSG00000120215 |  | (CT)3 |
| MLLT3 | ENSG00000171843 | ENST00000492661 | (AT)3 |
| MLXIP | ENSG00000175727 | ENST00000462837  ENST00000609457 | (CT)3 |
|  |  |  | (CA)3 |
| MME | ENSG00000196549 | ENST00000474994 | (AC)3 |
|  |  | ENST00000428261 | (TA)3 |
| MMRN2 | ENSG00000173269 | ENST00000415443 | (GA)3 |
|  |  | ENST00000447324 |  |
| MOBP | ENSG00000168314 | ENST00000441980 | (CCT)3 |
|  |  | ENST00000373195 |  |
|  |  | ENST00000540615 | (TA)3 |
|  |  |  | (CCT)3 |
| MOCS1 | ENSG00000124615 | ENST00000395852 | (CG)3 |
| MOV10L1 | ENSG00000073146 | ENST00000393529  ENST00000409474 | (TC)3 |
|  |  |  |  |
|  |  | ENST00000315506 | (TG)3 |
| MPP1 | ENSG00000130830 | ENST00000396886 | (AG)3 |
| MPP4 | ENSG00000082126 | ENST00000620095 | (TG)3 |
|  |  | ENST00000359962 |  |
|  |  | ENST00000428900 |  |
|  |  | ENST00000409143 |  |
|  |  | ENST00000447335 |  |
|  |  | ENST00000475711 |  |
|  |  | ENST00000464896 |  |
|  |  | ENST00000396275 |  |
| MRAS | ENSG00000158186 | ENST00000531264 | (AG)3 |
|  |  | ENST00000265229 |  |
| MRGPRX3 | ENSG00000179826 | ENST00000595444 | (CT)3 |
|  |  | ENST00000594999 |  |
| MRPL22 | ENSG00000082515 | ENST00000310614 | (GGC)3 |
| MRPL34 | ENSG00000130312 | ENST00000366746 | (CCT)3 |
|  |  | ENST00000366747 | (CTGC)3 |
| MRPL48 | ENSG00000175581 | ENST00000495225 | (TGC)3 |
| MRPL55 | ENSG00000162910 | ENST00000418740 | (GCA)3 |
|  |  | ENST00000508863 |  |
| MRPS22 | ENSG00000175110 | ENST00000469351 | (GA)3 |
| MRPS24 | ENSG00000062582 | ENST00000531787 | (CA)3 |
| MRPS27 | ENSG00000113048 | ENST00000278888 | (GC)3 |
| MRPS33 | ENSG00000090263 | ENST00000375750 | (GGC)3 |
| MS4A14 | ENSG00000166928 | ENST00000425703 | (CA)3 |
| MS4A2 | ENSG00000149534 | ENST00000455383 | (ATA)3 |
| MSH5 | ENSG00000204410 | ENST00000563941 | (GC)3 |
|  |  | ENST00000545450 |  |
| MSH6 | ENSG00000116062 |  | (GA)3 |
| MSLN | ENSG00000102854 | ENST00000566549 | (AG)3 |
|  |  | ENST00000382862 | (CG)3 |
|  |  |  | (AG)3 |
|  |  | ENST00000563651 | (CG)3 |
|  |  |  | (AG)3 |
|  |  | ENST00000308259 | (CG)3 |
|  |  |  | (AG)3 |
|  |  | ENST00000376592  ENST00000641407 | (CG)3 |
|  |  |  | (AG)3 |
| MSRB3 | ENSG00000174099 | ENST00000409459 | (TCTGCC)3 |
| MTHFR | ENSG00000177000 | ENST00000444541 | (TG)3 |
|  |  | ENST00000417581 |  |
| MTMR2 | ENSG00000087053 | ENST00000542829 |  |
|  |  | ENST00000447234 |  |
| MTPAP | ENSG00000107951 | ENST00000436408 |  |
| MTUS2 | ENSG00000132938 | ENST00000445522 | (GCA)3 |
| MUC20 | ENSG00000176945 | ENST00000421673 | (GA)3 |
|  |  | ENST00000539335 |  |
|  |  | ENST00000546277 | (TG)3 |
| MUC6 | ENSG00000184956 | ENST00000592148 | (GCA)3 |
| MVK | ENSG00000110921 |  | (CA)3 |
|  |  | ENST00000357337 |  |
| MXRA7 | ENSG00000182534 | ENST00000262269  ENST00000506838 | (TG)3 |
|  |  |  | (GA)3 |
| MYCBP2 | ENSG00000005810 |  | (CCT)3 |
| MYH14 | ENSG00000105357 | ENST00000505477 | (GCA)3 |
| MYL5 | ENSG00000215375 | ENST00000511290 | (AAG)3 |
|  |  |  | (AG)3 |
|  |  | ENST00000507804 | (AAG)3 |
|  |  |  | (AG)3 |
|  |  | ENST00000508240  ENST00000515803 | (AAG)3 |
|  |  |  | (AG)3 |
|  |  | ENST00000205890 | (TC)3 |
| MYLK | ENSG00000065534 | ENST00000615845 | (GAG)3 |
| MYO10 | ENSG00000145555 | ENST00000647165 | (TC)3 |
| MYO15A | ENSG00000091536 | ENST00000356711 | (AG)3 |
|  |  | ENST00000438652 |  |
|  |  | ENST00000399231 |  |
| MYO16 | ENSG00000041515 | ENST00000613858 | (GA)3 |
| MYO1B | ENSG00000128641 | ENST00000399233 | (AG)3 |
| MYO5A | ENSG00000197535 | ENST00000358212 | (GCCCT)3 |
|  |  | ENST00000566885 |  |
|  |  | ENST00000523438 |  |
|  |  | ENST00000613327 |  |
| MYO9A | ENSG00000066933 | ENST00000396217 | (GA)3 |
| MYOM2 | ENSG00000036448 |  | (AG)3 |
| MYPN | ENSG00000138347 | ENST00000515550 |  |
| MYRIP | ENSG00000170011 | ENST00000575208  ENST00000576384 | (TGA)3 |
|  |  |  | (GAA)3 |
| N4BP2 | ENSG00000078177 | ENST00000416973 | (AT)3 |
| NAA38 | ENSG00000183011 | ENST00000277554 | (GC)3 |
|  |  | ENST00000341426 |  |
| NAB1 | ENSG00000138386 |  | (CG)3 |
| NACC2 | ENSG00000148411 | ENST00000304788 | (GC)3 |
| NADK | ENSG00000008130 | ENST00000618691  ENST00000261182 | (GGC)3 |
|  |  |  | (CGG)3 |
| NANP | ENSG00000170191 | ENST00000393263 | (TC)3 |
| NAP1L1 | ENSG00000187109 | ENST00000547773 | (GCT)3 |
|  |  | ENST00000549596 |  |
|  |  | ENST00000552342 |  |
|  |  | ENST00000550934 |  |
|  |  | ENST00000548273 |  |
|  |  | ENST00000551600 |  |
|  |  | ENST00000547704 |  |
|  |  | ENST00000547479 |  |
|  |  | ENST00000373079 |  |
|  |  | ENST00000528968 |  |
|  |  | ENST00000531291 |  |
| NAP1L3 | ENSG00000186310 | ENST00000534372 | (CG)3 |
| NAP1L4 | ENSG00000205531 | ENST00000417955 | (CTG)3 |
|  |  | ENST00000579198 |  |
|  |  | ENST00000390006 |  |
| NAPEPLD | ENSG00000161048 | ENST00000580296 | (CT)3 |
| NARF | ENSG00000141562 | ENST00000584411 | (GA)3 |
|  |  | ENST00000577410 |  |
|  |  | ENST00000537702 |  |
|  |  | ENST00000379922 |  |
|  |  | ENST00000428975 |  |
| NBEA | ENSG00000172915 | ENST00000454000 | (CA)3 |
|  |  | ENST00000342104 | (AG)3 |
| NBL1 | ENSG00000158747 | ENST00000318249 |  |
| NBPF3 | ENSG00000142794 | ENST00000530396 | (TG)3 |
|  |  | ENST00000455200 |  |
|  |  | ENST00000321256 |  |
| NCAPD3 | ENSG00000151503 | ENST00000639756 | (GT)3 |
| NCAPH | ENSG00000121152 | ENST00000288986 |  |
| NCBP2 | ENSG00000114503 | ENST00000476286 | (CG)3 |
| NCBP2L | ENSG00000170935 | ENST00000368357 | (GTG)3 |
| NCK1 | ENSG00000158092 | ENST00000582490 | (CT)3 |
|  |  |  | (TC)3 |
| NCOA7 | ENSG00000111912 | ENST00000554531 | (CA)3 |
| NDEL1 | ENSG00000166579 | ENST00000563978  ENST00000568424 | (AG)3 |
|  |  |  | (TC)3 |
| NDRG2 | ENSG00000165795 | ENST00000252711 | (CT)3 |
| NDRG4 | ENSG00000103034 | ENST00000303553 | (TC)3 |
|  |  | ENST00000470123 |  |
| NDUFA10 | ENSG00000130414 | ENST00000555441 | (GA)3 |
| NDUFA3 | ENSG00000170906 | ENST00000482954 | (TCG)3 |
| NDUFA5 | ENSG00000128609 | ENST00000460088 | (GA)3 |
| NDUFB1 | ENSG00000183648 | ENST00000472695 | (CT)3 |
| NDUFB2 | ENSG00000090266 | ENST00000476470 | (GGT)3 |
|  |  | ENST00000450023 |  |
|  |  | ENST00000276689 |  |
|  |  | ENST00000518008 |  |
| NDUFB3 | ENSG00000119013 | ENST00000522532 | (CCG)3 |
| NDUFB9 | ENSG00000147684 | ENST00000517367 | (CG)3 |
|  |  | ENST00000565691 |  |
|  |  | ENST00000538004 |  |
|  |  | ENST00000579060 |  |
| NECAB2 | ENSG00000103154 | ENST00000391969 | (CT)3 |
| NEK7 | ENSG00000151414 | ENST00000435934 | (AGG)3 |
| NEK8 | ENSG00000160602 | ENST00000435894 | (GC)3 |
| NEU4 | ENSG00000204099 | ENST00000253506 | (GA)3 |
|  |  | ENST00000591814 |  |
|  |  | ENST00000413692 |  |
| NFATC1 | ENSG00000131196 | ENST00000554591 | (GC)3 |
|  |  | ENST00000557674 |  |
| NFATC4 | ENSG00000100968 | ENST00000556169 | (AG)3 |
|  |  | ENST00000422617 |  |
|  |  | ENST00000555453 | (CT)3 |
|  |  | ENST00000554473 |  |
|  |  | ENST00000556759 |  |
|  |  | ENST00000555167 |  |
|  |  | ENST00000641145 |  |
|  |  | ENST00000297689 |  |
|  |  | ENST00000505458 |  |
| NFIC | ENSG00000141905 | ENST00000509165 | (TG)3 |
| NFIL3 | ENSG00000165030 | ENST00000557389 | (AC)3 |
| NFKB1 | ENSG00000109320 | ENST00000419569 | (GA)3 |
|  |  | ENST00000303698 | (AC)3 |
| NFKBIA | ENSG00000100906 | ENST00000372669 | (CT)3 |
| NFS1 | ENSG00000244005 | ENST00000369506 | (TC)3 |
| NFU1 | ENSG00000169599 | ENST00000533765 |  |
| NFYC | ENSG00000066136 | ENST00000437912 | (AG)3 |
| NHLH2 | ENSG00000177551 |  | (CG)3 |
| NHSL1 | ENSG00000135540 | ENST00000561183 | (GAA)3 |
| NIPA1 | ENSG00000170113 | ENST00000398013  ENST00000539711 | (CA)3 |
|  |  |  | (TG)3 |
|  |  | ENST00000370313 |  |
| NIPA2 | ENSG00000140157 | ENST00000370307 | (GCCG)3 |
|  |  | ENST00000416026 |  |
| NKAIN4 | ENSG00000101198 | ENST00000575301 | (CAT)3 |
|  |  | ENST00000302926 |  |
| NKIRAS1 | ENSG00000197885 | ENST00000587891 | (TC)3 |
| NLGN2 | ENSG00000169992 | ENST00000586553 | (AGGG)3 |
|  |  | ENST00000536624 |  |
| NLRP4 | ENSG00000160505 | ENST00000330537 | (GT)3 |
| NOL4 | ENSG00000101746 | ENST00000399336 | (GA)3 |
| NOL8 | ENSG00000198000 | ENST00000493151 | (CT)3 |
| NOMO2 | ENSG00000185164 | ENST00000599537 | (AGG)3 |
| NOMO3 | ENSG00000103226 | ENST00000347476 |  |
| NOS1AP | ENSG00000198929 | ENST00000424319 | (CTT)3 |
| NOSIP | ENSG00000142546 | ENST00000527956 | (CA)3 |
| NOVA1 | ENSG00000139910 | ENST00000341349 | (TA)3 |
| NOX4 | ENSG00000086991 |  | (CA)3 |
|  |  | ENST00000392815 |  |
| NOXA1 | ENSG00000188747 | ENST00000602189 | (CGC)3 |
|  |  |  | (GGCCCC)3 |
|  |  | ENST00000378156  ENST00000622020 | (CGC)3 |
|  |  |  | (GGCCCC)3 |
| NPAS1 | ENSG00000130751 | ENST00000468260 | (TC)3 |
| NPHP4 | ENSG00000131697 | ENST00000329476 | (CT)3 |
|  |  | ENST00000504572 |  |
| NPRL3 | ENSG00000103148 | ENST00000424077 | (CCA)3 |
| NPY2R | ENSG00000185149 | ENST00000520407 | (CA)3 |
| NR3C1 | ENSG00000113580 | ENST00000372142 | (GA)3 |
| NR4A2 | ENSG00000153234 | ENST00000571172 | (TGAA)3 |
| NRG1 | ENSG00000157168 | ENST00000624579 | (AGG)3 |
| NRG3 | ENSG00000185737 | ENST00000542733 | (TG)3 |
| NSF | ENSG00000073969 | ENST00000537969 | (GGA)3 |
| NTAN1 | ENSG00000157045 | ENST00000566340 | (CA)3 |
| NTRK3 | ENSG00000140538 | ENST00000512216 | (AC)3 |
| NUDT13 | ENSG00000166321 | ENST00000473942 | (GT)3 |
| NUDT21 | ENSG00000167005 | ENST00000534289 |  |
| NUDT9 | ENSG00000170502 |  | (AG)3 |
|  |  | ENST00000445721 |  |
| NUF2 | ENSG00000143228 | ENST00000396096  ENST00000469968 | (CT)3 |
|  |  |  | (AG)3 |
| NUP50 | ENSG00000093000 | ENST00000391875 | (TA)3 |
|  |  | ENST00000537026 | (TG)3 |
| NVL | ENSG00000143748 |  | (TGA)3 |
|  |  | ENST00000602676 |  |
| NXF5 | ENSG00000126952 | ENST00000582888  ENST00000416608 | (AC)3 |
|  |  |  | (AG)3 |
| OAZ1 | ENSG00000104904 | ENST00000257627 | (GA)3 |
|  |  | ENST00000394733 |  |
| OCM | ENSG00000122543 | ENST00000397389 | (TA)3 |
| OCM2 | ENSG00000135175 |  | (TG)3 |
| ODF2L | ENSG00000122417 | ENST00000284719 | (GA)3 |
| OGFOD2 | ENSG00000111325 | ENST00000371796  ENST00000382349 | (TCC)3 |
|  |  |  | (TG)3 |
| OLA1 | ENSG00000138430 | ENST00000544371 | (TC)3 |
| OLFM1 | ENSG00000130558 |  |  |
| ONECUT3 | ENSG00000205922 |  | (CG)3 |
| OPA3 | ENSG00000125741 | ENST00000536387  ENST00000641185  ENST00000641722 | (GA)3 |
|  |  |  | (GCC)3 |
|  |  |  | (CG)3 |
| OPALIN | ENSG00000197430 | ENST00000641521 | (GAA)3 |
| OR10G3 | ENSG00000169208 |  | (TC)3 |
| OR10G4 | ENSG00000254737 | ENST00000641224 | (AG)3 |
|  |  | ENST00000641460 |  |
|  |  |  |  |
| OR10G8 | ENSG00000234560 | ENST00000641042 | (TC)3 |
|  |  |  | (AG)3 |
| OR10K1 | ENSG00000173285 | ENST00000377148  ENST00000641879 | (TC)3 |
|  |  |  | (AG)3 |
| OR10K2 | ENSG00000180708 | ENST00000374401 | (TC)3 |
| OR11A1 | ENSG00000204694 | ENST00000377981 | (AT)3 |
| OR11G2 | ENSG00000196832 | ENST00000641895 | (CTG)3 |
| OR13A1 | ENSG00000256574 | ENST00000641732 | (TC)3 |
| OR13J1 | ENSG00000168828 | ENST00000373688 |  |
| OR14J1 | ENSG00000204695 |  | (GA)3 |
| OR1A1 | ENSG00000172146 | ENST00000641068 | (CT)3 |
| OR1N2 | ENSG00000171501 | ENST00000641841  ENST00000641124 | (CA)3 |
|  |  |  | (AATA)3 |
| OR2A14 | ENSG00000221938 | ENST00000641169 | (CT)3 |
| OR2A7 | ENSG00000243896 | ENST00000641114 | (TG)3 |
| OR2AG2 | ENSG00000188124 | ENST00000641931 | (AT)3 |
|  |  | ENST00000303324 |  |
| OR2AP1 | ENSG00000179615 | ENST00000641802 | (CT)3 |
| OR2AT4 | ENSG00000171561 |  | (AC)3 |
| OR2B2 | ENSG00000168131 | ENST00000366487 | (GT)3 |
| OR2C3 | ENSG00000196242 | ENST00000641417 | (CT)3 |
|  |  |  |  |
|  |  | ENST00000641151 |  |
|  |  |  |  |
| OR2J2 | ENSG00000204700 | ENST00000641893  ENST00000641211 | (AG)3 |
|  |  |  |  |
| OR2J3 | ENSG00000204701 | ENST00000641836 | (GAA)3 |
| OR2L13 | ENSG00000196071 | ENST00000642130 | (GA)3 |
| OR2M2 | ENSG00000198601 | ENST00000641069 | (AG)3 |
|  |  | ENST00000573491 | (AC)3 |
| OR2T2 | ENSG00000196240 | ENST00000576166 | (TTCT)3 |
| OR2T29 | ENSG00000182783 | ENST00000641141 | (CA)3 |
| OR3A2 | ENSG00000221882 | ENST00000300127 | (GGT)3 |
|  |  | ENST00000641278 |  |
| OR3A3 | ENSG00000159961 | ENST00000641962 | (TG)3 |
| OR4D6 | ENSG00000166884 | ENST00000641429 | (AG)3 |
| OR4D9 | ENSG00000172742 | ENST00000641086 | (GA)3 |
|  |  | ENST00000642117 |  |
| OR4K1 | ENSG00000155249 | ENST00000396952 | (GAA)3 |
| OR4N5 | ENSG00000184394 | ENST00000641672 | (GA)3 |
| OR4Q3 | ENSG00000182652 | ENST00000641926 | (AG)3 |
| OR51E1 | ENSG00000180785 |  | (CA)3 |
| OR51F2 | ENSG00000176925 | ENST00000641726 | (AG)3 |
| OR51G2 | ENSG00000176893 | ENST00000641896  ENST00000641528 |  |
|  |  |  | (CA)3 |
| OR52E4 | ENSG00000180974 | ENST00000641350 | (AG)3 |
| OR52I2 | ENSG00000226288 | ENST00000311352 | (TCT)3 |
| OR52K1 | ENSG00000196778 | ENST00000641900 | (AT)3 |
| OR52N4 | ENSG00000181074 | ENST00000641921 | (TC)3 |
| OR52W1 | ENSG00000175485 | ENST00000641342 |  |
| OR56A1 | ENSG00000180934 |  | (AG)3 |
| OR5B12 | ENSG00000172362 | ENST00000641874 | (TA)3 |
| OR5B2 | ENSG00000172365 | ENST00000427338  ENST00000526812 | (TCC)3 |
|  |  |  | (GT)3 |
| OR5H1 | ENSG00000231192 | ENST00000641993 | (AT)3 |
| OR5K2 | ENSG00000231861 | ENST00000641661 | (AG)3 |
| OR5M10 | ENSG00000254834 | ENST00000641196 |  |
| OR5M3 | ENSG00000174937 |  | (AT)3 |
| OR5T2 | ENSG00000181718 | ENST00000641740 | (TA)3 |
| OR6A2 | ENSG00000184933 | ENST00000641851  ENST00000358433 | (CT)3 |
|  |  |  | (AG)3 |
| OR6C3 | ENSG00000205329 | ENST00000641622 | (GA)3 |
| OR6C4 | ENSG00000179626 | ENST00000642123 | (TA)3 |
| OR6C6 | ENSG00000188324 | ENST00000641113 | (CA)3 |
| OR6Y1 | ENSG00000197532 | ENST00000322301 | (TGG)3 |
| OR7A17 | ENSG00000185385 | ENST00000248073 | (GA)3 |
|  |  |  |  |
| OR7A5 | ENSG00000188269 | ENST00000284287 |  |
| OR7C1 | ENSG00000127530 | ENST00000642111  ENST00000641707 | (GT)3 |
|  |  |  | (CT)3 |
| OR8A1 | ENSG00000196119 | ENST00000350513 | (AG)3 |
|  |  | ENST00000641559 |  |
| OR8G5 | ENSG00000255298 | ENST00000566860 | (AT)3 |
| OR9A2 | ENSG00000179468 | ENST00000446658 | (CT)3 |
| OR9A4 | ENSG00000258083 | ENST00000535268 | (GA)3 |
| ORC6 | ENSG00000091651 | ENST00000439951 | (AC)3 |
| OSBP2 | ENSG00000184792 | ENST00000645520 | (GC)3 |
|  |  | ENST00000645442 | (GGCC)3 |
| OSBPL2 | ENSG00000130703 | ENST00000526122 | (GT)3 |
|  |  | ENST00000433045 | (TC)3 |
|  |  | ENST00000457907 | (GT)3 |
| OSBPL5 | ENSG00000021762 | ENST00000428192 | (TCCT)3 |
| OSCP1 | ENSG00000116885 | ENST00000307050 | (CTC)3 |
| OSR2 | ENSG00000164920 | ENST00000431881 | (CG)3 |
| OTUB1 | ENSG00000167770 | ENST00000512084 | (TG)3 |
| OTUD7A | ENSG00000169918 | ENST00000543457 | (TC)3 |
| OXA1L | ENSG00000155463 | ENST00000541518 | (GA)3 |
| OXCT1 | ENSG00000083720 | ENST00000442977 | (TG)3 |
| OXGR1 | ENSG00000165621 | ENST00000552276 | (GA)3 |
|  |  | ENST00000551178 |  |
| OXR1 | ENSG00000164830 | ENST00000547178 | (TGCC)3 |
| P2RX5 | ENSG00000083454 | ENST00000225328 | (GC)3 |
|  |  | ENST00000345901 |  |
|  |  | ENST00000302632 |  |
|  |  | ENST00000439698 |  |
|  |  | ENST00000416053 |  |
| P2RY12 | ENSG00000169313 | ENST00000551061 | (ACT)3 |
| P4HA2 | ENSG00000072682 |  | (TA)3 |
|  |  | ENST00000541951 |  |
| PA2G4 | ENSG00000170515 | ENST00000504441  ENST00000535604 | (GT)3 |
|  |  |  | (TG)3 |
| PAAF1 | ENSG00000175575 | ENST00000542293 | (GT)3 |
|  |  | ENST00000546039 | (CT)3 |
|  |  | ENST00000544909 | (GT)3 |
|  |  | ENST00000281589 |  |
|  |  | ENST00000421491 |  |
|  |  | ENST00000514292 | (AG)3 |
| PABPC3 | ENSG00000151846 | ENST00000509625 | (TG)3 |
| PABPC4L | ENSG00000254535 | ENST00000533756 |  |
| PACRGL | ENSG00000163138 | ENST00000289619 | (CT)3 |
|  |  | ENST00000374955 | (CTCC)3 |
| PACS1 | ENSG00000175115 | ENST00000399688 | (AC)3 |
| PAGE5 | ENSG00000158639 | ENST00000512576 | (TG)3 |
|  |  | ENST00000528203 | (GA)3 |
| PAICS | ENSG00000128050 | ENST00000321944 | (CT)3 |
|  |  | ENST00000593480 |  |
| PAK1 | ENSG00000149269 | ENST00000599657 | (AC)3 |
| PAK4 | ENSG00000130669 | ENST00000511948 | (GT)3 |
|  |  | ENST00000621476 |  |
|  |  | ENST00000527605 |  |
| PALLD | ENSG00000129116 | ENST00000495692 | (TC)3 |
| PAMR1 | ENSG00000149090 | ENST00000610179 | (CT)3 |
|  |  | ENST00000554301 |  |
| PANK2 | ENSG00000125779 | ENST00000555445 | (GGA)3 |
|  |  | ENST00000558684 | (CTG)3 |
| PAPLN | ENSG00000100767 | ENST00000639100 | (AC)3 |
|  |  | ENST00000341484 |  |
| PAQR5 | ENSG00000137819 | ENST00000530478 | (CA)3 |
| PARL | ENSG00000175193 | ENST00000619710 | (TG)3 |
| PARN | ENSG00000140694 | ENST00000444029 | (AG)3 |
| PARP10 | ENSG00000178685 | ENST00000560110 | (CT)3 |
| PARVB | ENSG00000188677 | ENST00000554201 | (AGG)3 |
|  |  | ENST00000560641 | (GTTTT)3 |
| PATL2 | ENSG00000229474 | ENST00000482110 | (AG)3 |
| PAX9 | ENSG00000198807 | ENST00000287008 | (CGG)3 |
| PBX1 | ENSG00000185630 | ENST00000503492 | (AAT)3 |
|  |  | ENST00000394536 | (AT)3 |
| PCDH1 | ENSG00000156453 | ENST00000621505 | (AGCCGG)3 |
|  |  | ENST00000378133 |  |
|  |  | ENST00000614258 |  |
| PCDH11Y | ENSG00000099715 |  | (GA)3 |
| PCDHA1 | ENSG00000204970 | ENST00000529619 |  |
| PCDHA5 | ENSG00000204965 | ENST00000356878 | (TA)3 |
|  |  |  | (TG)3 |
|  |  | ENST00000532602  ENST00000378122 | (TA)3 |
|  |  |  | (TG)3 |
| PCDHA7 | ENSG00000204963 | ENST00000306549 | (CA)3 |
| PCDHA9 | ENSG00000204961 | ENST00000610539 | (AGA)3 |
|  |  | ENST00000560736 |  |
| PCDHB1 | ENSG00000171815 | ENST00000559837 | (AG)3 |
| PCDHGC4 | ENSG00000242419 | ENST00000545054 | (TG)3 |
| PCK2 | ENSG00000100889 | ENST00000561286 | (AG)3 |
|  |  | ENST00000558096 |  |
|  |  | ENST00000618073 |  |
|  |  | ENST00000469941 |  |
|  |  | ENST00000611716 |  |
| PCLO | ENSG00000186472 | ENST00000331826 | (TGGAA)3 |
| PCNP | ENSG00000081154 | ENST00000246535 | (AGC)3 |
| PCSK6 | ENSG00000140479 | ENST00000264933 | (GGC)3 |
|  |  | ENST00000614778 |  |
| PDCD2L | ENSG00000126249 | ENST00000618970 | (AG)3 |
| PDCD6 | ENSG00000249915 | ENST00000628729 | (TC)3 |
|  |  | ENST00000505221 | (CT)3 |
|  |  | ENST00000509581 | (TC)3 |
|  |  | ENST00000507528 |  |
|  |  | ENST00000435564 |  |
|  |  |  |  |
|  |  | ENST00000396191 |  |
| PDE1A | ENSG00000115252 | ENST00000321453  ENST00000396182 | (AG)3 |
|  |  |  | (GT)3 |
| PDE1C | ENSG00000154678 | ENST00000396189 | (CG)3 |
|  |  | ENST00000542223 | (GCT)3 |
|  |  | ENST00000341517 | (CG)3 |
|  |  | ENST00000412480 |  |
| PDE2A | ENSG00000186642 | ENST00000528771 | (TC)3 |
| PDE4B | ENSG00000184588 |  | (AGG)3 |
|  |  | ENST00000340635 | (ACC)3 |
|  |  | ENST00000360047  ENST00000507116 | (AC)3 |
|  |  |  | (GAA)3 |
| PDE4D | ENSG00000113448 | ENST00000502575 | (GCAGCA)3 |
|  |  | ENST00000479408 | (AG)3 |
|  |  | ENST00000428104 | (CAG)3 |
|  |  | ENST00000571224 |  |
| PDE4DIP | ENSG00000178104 | ENST00000448838 | (GAG)3 |
| PDE6D | ENSG00000156973 | ENST00000484644 | (CT)3 |
| PDE6G | ENSG00000185527 | ENST00000404371 |  |
| PDHX | ENSG00000110435 | ENST00000443353 | (GC)3 |
| PDIA5 | ENSG00000065485 | ENST00000456545 | (AG)3 |
| PDIA6 | ENSG00000143870 | ENST00000452226 | (GA)3 |
| PDK1 | ENSG00000152256 | ENST00000397761 | (GT)3 |
| PDLIM2 | ENSG00000120913 | ENST00000253754 | (GA)3 |
|  |  | ENST00000506537 | (TCCC)3 |
|  |  |  |  |
| PDLIM4 | ENSG00000131435 | ENST00000376215 | (CTC)3 |
| PDLIM7 | ENSG00000196923 | ENST00000368076  ENST00000455314 | (GCCT)3 |
|  |  |  | (TC)3 |
| PDSS1 | ENSG00000148459 | ENST00000292357 | (GCC)3 |
| PEA15 | ENSG00000162734 | ENST00000522278 | (AG)3 |
| PEAR1 | ENSG00000187800 | ENST00000265322 | (TCC)3 |
|  |  | ENST00000482108 |  |
| PEBP4 | ENSG00000134020 | ENST00000613043 | (GC)3 |
| PECR | ENSG00000115425 | ENST00000617526 | (CCGC)3 |
| PEG10 | ENSG00000242265 | ENST00000559044 | (GT)3 |
|  |  |  |  |
|  |  | ENST00000267460 |  |
| PELI2 | ENSG00000139946 | ENST00000527230  ENST00000570387 | (GTG)3 |
|  |  |  | (AC)3 |
|  |  | ENST00000540398 | (CGG)3 |
| PELI3 | ENSG00000174516 | ENST00000545845 | (GC)3 |
| PELP1 | ENSG00000141456 | ENST00000468741 | (TG)3 |
| PEX5 | ENSG00000139197 | ENST00000491640 | (CT)3 |
|  |  | ENST00000422701 |  |
| PEX5L | ENSG00000114757 | ENST00000550924 | (AG)3 |
|  |  | ENST00000574872 |  |
| PFKFB4 | ENSG00000114268 | ENST00000494827 | (GT)3 |
| PFKM | ENSG00000152556 | ENST00000489155 | (TC)3 |
| PFN1 | ENSG00000108518 | ENST00000325558 | (TA)3 |
| PFN2 | ENSG00000070087 |  | (TC)3 |
|  |  | ENST00000543505 | (GC)3 |
| PGA3 | ENSG00000229859 | ENST00000378149 | (TA)3 |
|  |  |  | (TGC)3 |
|  |  | ENST00000312403 | (AG)3 |
| PGA4 | ENSG00000229183 | ENST00000451616 | (TA)3 |
|  |  |  | (TGC)3 |
| PGA5 | ENSG00000256713 | ENST00000396993  ENST00000464229 | (TA)3 |
|  |  |  | (TGC)3 |
|  |  | ENST00000490830 | (TG)3 |
| PGAP2 | ENSG00000148985 | ENST00000479072 | (CTC)3 |
|  |  | ENST00000496834 |  |
|  |  | ENST00000465632 |  |
|  |  | ENST00000378919 |  |
|  |  |  |  |
| PGD | ENSG00000142657 | ENST00000394276 | (CT)3 |
| PGPEP1L | ENSG00000183571 | ENST00000512483  ENST00000503872 | (GTG)3 |
|  |  |  | (GT)3 |
| PGRMC2 | ENSG00000164040 | ENST00000503588 | (TC)3 |
|  |  | ENST00000355648 |  |
|  |  | ENST00000395636 |  |
|  |  | ENST00000361300 | (CT)3 |
| PHACTR3 | ENSG00000087495 | ENST00000427004 | (GA)3 |
|  |  | ENST00000583747 | (TC)3 |
|  |  | ENST00000374000 |  |
| PHF1 | ENSG00000112511 | ENST00000449988 | (GCC)3 |
| PHF12 | ENSG00000109118 | ENST00000529734 | (GA)3 |
| PHF20 | ENSG00000025293 | ENST00000529782 |  |
|  |  | ENST00000515594 | (AGG)3 |
| PHF21A | ENSG00000135365 | ENST00000514822 | (CAGC)3 |
|  |  | ENST00000565897 | (AC)3 |
| PHF3 | ENSG00000118482 | ENST00000449120 | (GAA)3 |
|  |  | ENST00000393046 | (CT)3 |
| PHKG2 | ENSG00000156873 | ENST00000525162 | (TCC)3 |
| PI4KA | ENSG00000241973 | ENST00000392054 | (GTC)3 |
| PIAS3 | ENSG00000131788 | ENST00000474980 | (TC)3 |
| PICALM | ENSG00000073921 | ENST00000510235 | (AC)3 |
| PID1 | ENSG00000153823 | ENST00000640145 | (GA)3 |
| PIGF | ENSG00000151665 | ENST00000400334 | (TC)3 |
| PIGG | ENSG00000174227 | ENST00000639902 | (AT)3 |
| PIGN | ENSG00000197563 | ENST00000638936 | (AGC)3 |
|  |  | ENST00000640050 | (GAT)3 |
|  |  | ENST00000639174 |  |
|  |  | ENST00000638329 |  |
|  |  | ENST00000588571 | (TG)3 |
|  |  | ENST00000321878 | (GAT)3 |
|  |  | ENST00000422307 |  |
|  |  | ENST00000026218 |  |
| PIGQ | ENSG00000007541 | ENST00000470411 | (GC)3 |
|  |  | ENST00000614443 |  |
|  |  | ENST00000451319 |  |
|  |  | ENST00000601807 |  |
| PIGW | ENSG00000277161 | ENST00000597415 | (CG)3 |
| PIGX | ENSG00000163964 | ENST00000595550 | (AGT)3 |
| PIH1D1 | ENSG00000104872 | ENST00000371109 | (GCT)3 |
|  |  | ENST00000544716 |  |
|  |  | ENST00000373509 |  |
| PIK3AP1 | ENSG00000155629 | ENST00000545335 | (GA)3 |
| PIK3CB | ENSG00000051382 | ENST00000418435 | (TG)3 |
| PIM1 | ENSG00000137193 |  | (CAGCCA)3 |
| PIP4K2A | ENSG00000150867 | ENST00000246151 | (CAC)3 |
| PIP5K1A | ENSG00000143398 | ENST00000415372  ENST00000415296 | (AG)3 |
|  |  |  |  |
| PITHD1 | ENSG00000057757 | ENST00000503015 | (CGAGC)3 |
|  |  | ENST00000511176 | (CA)3 |
| PITPNB | ENSG00000180957 | ENST00000372889 | (TGG)3 |
| PKD2L2 | ENSG00000078795 |  | (TA)3 |
|  |  | ENST00000349959 |  |
| PKIG | ENSG00000168734 | ENST00000291906  ENST00000628904 | (AG)3 |
|  |  |  | (TG)3 |
|  |  | ENST00000389757 | (CT)3 |
| PKN3 | ENSG00000160447 | ENST00000389759 | (GA)3 |
| PKP4 | ENSG00000144283 | ENST00000290472 | (CGC)3 |
|  |  | ENST00000359237 |  |
|  |  | ENST00000411416 |  |
| PLA2G4D | ENSG00000159337 | ENST00000372270 | (AG)3 |
| PLAC1 | ENSG00000170965 | ENST00000392307 | (AGA)3 |
| PLAC8 | ENSG00000145287 | ENST00000429089 | (GC)3 |
| PLAC9 | ENSG00000189129 | ENST00000540572 | (AG)3 |
| PLAGL1 | ENSG00000118495 | ENST00000378641 | (CA)3 |
| PLAT | ENSG00000104368 | ENST00000338037 | (AGGG)3 |
| PLBD1 | ENSG00000121316 | ENST00000629992 | (GA)3 |
| PLCB1 | ENSG00000182621 | ENST00000404098 | (GCGCCCC)3 |
|  |  | ENST00000334686 |  |
|  |  |  |  |
|  |  | ENST00000419816 |  |
| PLCH1 | ENSG00000114805 | ENST00000378486  ENST00000263088 | (TG)3 |
|  |  |  | (GT)3 |
| PLCH2 | ENSG00000149527 | ENST00000572940 | (GA)3 |
|  |  | ENST00000599685 |  |
| PLD2 | ENSG00000129219 | ENST00000354958 | (CT)3 |
|  |  | ENST00000595867 |  |
| PLD3 | ENSG00000105223 | ENST00000535129 | (GC)3 |
| PLEC | ENSG00000178209 | ENST00000542389 | (TGG)3 |
| PLEKHA4 | ENSG00000105559 | ENST00000283426 | (CAG)3 |
| PLEKHB1 | ENSG00000021300 | ENST00000377748 | (GCT)3 |
|  |  | ENST00000377740 |  |
| PLEKHG4B | ENSG00000153404 | ENST00000011684 | (TG)3 |
| PLEKHG5 | ENSG00000171680 | ENST00000449001 |  |
|  |  |  |  |
| PLEKHG6 | ENSG00000008323 | ENST00000375799 | (CG)3 |
|  |  | ENST00000375793  ENST00000409310 | (TG)3 |
|  |  |  | (CA)3 |
| PLEKHM2 | ENSG00000116786 | ENST00000355705 | (GGTGGC)3 |
|  |  | ENST00000359481 |  |
| PLGLB1 | ENSG00000183281 | ENST00000221957 | (AC)3 |
|  |  | ENST00000592528 |  |
| PLGLB2 | ENSG00000125551 | ENST00000585479 |  |
| PLIN3 | ENSG00000105355 | ENST00000567897 | (CG)3 |
|  |  |  |  |
|  |  | ENST00000568568 |  |
| PLK1 | ENSG00000166851 | ENST00000469350 | (TG)3 |
|  |  |  |  |
|  |  | ENST00000464320  ENST00000551850 |  |
|  |  |  |  |
| PLOD2 | ENSG00000152952 | ENST00000550080 | (AG)3 |
| PLS1 | ENSG00000120756 |  | (CA)3 |
| PLXNC1 | ENSG00000136040 | ENST00000547057 | (AG)3 |
|  |  | ENST00000355636 | (CT)3 |
|  |  |  | (GTTTTT)3 |
|  |  | ENST00000639501  ENST00000418224 | (CT)3 |
|  |  |  | (GTTTTT)3 |
| PMFBP1 | ENSG00000118557 | ENST00000618056 | (TC)3 |
| PMS1 | ENSG00000064933 | ENST00000441310 | (TGCGGG)3 |
|  |  | ENST00000409985 |  |
|  |  | ENST00000420421 |  |
|  |  | ENST00000370150 |  |
|  |  | ENST00000439087 |  |
|  |  |  | (ATT)3 |
| PNCK | ENSG00000130822 | ENST00000422811 | (GC)3 |
|  |  | ENST00000434652 | (TG)3 |
|  |  |  | (AG)3 |
|  |  | ENST00000600910 | (GGA)3 |
|  |  | ENST00000322344  ENST00000600573 | (TG)3 |
|  |  |  | (AG)3 |
| PNKP | ENSG00000039650 | ENST00000596726 | (CCG)3 |
|  |  | ENST00000312917 |  |
|  |  | ENST00000388715 |  |
|  |  | ENST00000530511 |  |
| PNPLA1 | ENSG00000180316 | ENST00000370162 | (GGA)3 |
|  |  | ENST00000541869 |  |
| POLD3 | ENSG00000077514 | ENST00000428025 | (CA)3 |
| POLL | ENSG00000166169 | ENST00000646188 | (TCC)3 |
| POLR1B | ENSG00000125630 | ENST00000399696 | (AG)3 |
| POLR1C | ENSG00000171453 | ENST00000492213 | (ATG)3 |
|  |  | ENST00000439629 |  |
| POLR1D | ENSG00000186184 | ENST00000380842 | (TG)3 |
| POLR2F | ENSG00000100142 | ENST00000449920 | (GGA)3 |
| POM121C | ENSG00000272391 | ENST00000451021 | (AG)3 |
| POMP | ENSG00000132963 | ENST00000448077 |  |
| POR | ENSG00000127948 | ENST00000538433 | (ACC)3 |
| POU6F2 | ENSG00000106536 | ENST00000537927 | (GT)3 |
| PPARD | ENSG00000112033 | ENST00000527790 | (CGG)3 |
| PPFIBP1 | ENSG00000110841 | ENST00000256678 | (GT)3 |
|  |  | ENST00000457063 |  |
| PPFIBP2 | ENSG00000166387 | ENST00000395076 | (AGG)3 |
| PPHLN1 | ENSG00000134283 | ENST00000424647 | (GA)3 |
| PPIL3 | ENSG00000240344 | ENST00000497343 |  |
| PPM1A | ENSG00000100614 | ENST00000498165 | (CGC)3 |
| PPM1F | ENSG00000100034 | ENST00000295839 | (GCT)3 |
| PPM1L | ENSG00000163590 | ENST00000456399 | (TGG)3 |
|  |  | ENST00000396737 |  |
|  |  | ENST00000455580 | (AG)3 |
| PPM1N | ENSG00000213889 | ENST00000420282 |  |
|  |  | ENST00000296122 |  |
| PPP1CB | ENSG00000213639 |  | (GCG)3 |
|  |  | ENST00000263433 |  |
|  |  | ENST00000587515  ENST00000542235 | (CT)3 |
|  |  |  | (ATTT)3 |
| PPP1R12C | ENSG00000125503 | ENST00000529009 | (GCGG)3 |
| PPP1R14A | ENSG00000167641 | ENST00000394265 | (GAGG)3 |
| PPP1R14B | ENSG00000173457 | ENST00000310455 | (TC)3 |
| PPP1R16A | ENSG00000160972 | ENST00000523082 | (CA)3 |
| PPP1R1B | ENSG00000131771 | ENST00000507294 | (AG)3 |
| PPP1R3B | ENSG00000173281 | ENST00000591228 | (AC)3 |
| PPP2CA | ENSG00000113575 | ENST00000638454 | (CTA)3 |
| PPP2R2C | ENSG00000074211 | ENST00000616979 | (AC)3 |
| PPY | ENSG00000108849 | ENST00000357367 | (TG)3 |
| PRAMEF13 | ENSG00000279169 | ENST00000534264 | (GT)3 |
| PRAMEF7 | ENSG00000204510 | ENST00000409411 | (GA)3 |
| PRAMEF8 | ENSG00000182330 | ENST00000410081 |  |
| PRCP | ENSG00000137509 | ENST00000438314 | (CA)3 |
| PREPL | ENSG00000138078 | ENST00000639589 | (TC)3 |
|  |  | ENST00000330452 |  |
|  |  | ENST00000487897 |  |
| PRICKLE1 | ENSG00000139174 | ENST00000555185 | (GCGG)3 |
| PRKCD | ENSG00000163932 | ENST00000593101 | (AGC)3 |
|  |  | ENST00000378567 |  |
| PRKCH | ENSG00000027075 | ENST00000468310 | (CTG)3 |
| PRKCSH | ENSG00000130175 | ENST00000545647 | (TG)3 |
| PRKCZ | ENSG00000067606 | ENST00000462601 | (CGG)3 |
|  |  | ENST00000450579 |  |
| PRKG2 | ENSG00000138669 | ENST00000322583 | (TGA)3 |
| PRKRIP1 | ENSG00000128563 | ENST00000377931 | (CAT)3 |
| PRODH | ENSG00000100033 | ENST00000614894 | (GGA)3 |
| PRR18 | ENSG00000176381 | ENST00000452123 | (CT)3 |
| PRR20A | ENSG00000204919 | ENST00000434815 |  |
| PRR20C | ENSG00000229665 | ENST00000611394 |  |
| PRR20D | ENSG00000227151 |  |  |
| PRR20E | ENSG00000234278 | ENST00000432186 |  |
| PRR5 | ENSG00000186654 | ENST00000562148  ENST00000636246 | (GCGGG)3 |
|  |  |  | (GGCTCC)3 |
|  |  | ENST00000379405 |  |
| PRRT2 | ENSG00000167371 | ENST00000460241 | (GC)3 |
|  |  | ENST00000567164 | (TGG)3 |
| PRSS3 | ENSG00000010438 | ENST00000219301 | (CCA)3 |
| PRSS50 | ENSG00000206549 | ENST00000376717 | (GA)3 |
| PRSS54 | ENSG00000103023 | ENST00000223609 | (CA)3 |
|  |  | ENST00000303728 |  |
| PRUNE2 | ENSG00000106772 | ENST00000303804 | (AG)3 |
|  |  | ENST00000461698 |  |
| PRY | ENSG00000169789 | ENST00000472139 |  |
| PRY2 | ENSG00000169807 | ENST00000436291 |  |
| PSD | ENSG00000059915 |  | (GC)3 |
| PSEN2 | ENSG00000143801 | ENST00000595124 | (AC)3 |
| PSG1 | ENSG00000231924 | ENST00000595356 | (AG)3 |
|  |  |  | (AC)3 |
|  |  | ENST00000403380 | (AG)3 |
|  |  |  | (AC)3 |
|  |  | ENST00000312439 | (AG)3 |
|  |  |  | (AC)3 |
|  |  | ENST00000244296 | (AG)3 |
|  |  |  | (AC)3 |
|  |  | ENST00000405312 | (AG)3 |
|  |  |  | (AC)3 |
|  |  | ENST00000433626  ENST00000244295 | (AG)3 |
|  |  |  | (AC)3 |
| PSG4 | ENSG00000243137 | ENST00000596907 |  |
|  |  | ENST00000451895 |  |
|  |  | ENST00000366175 |  |
|  |  | ENST00000342951 |  |
|  |  | ENST00000187910 | (CA)3 |
| PSG5 | ENSG00000204941 | ENST00000402603 | (GA)3 |
|  |  | ENST00000292125 |  |
| PSG6 | ENSG00000170848 | ENST00000594375 | (AC)3 |
|  |  | ENST00000404209 |  |
|  |  | ENST00000406636 |  |
|  |  | ENST00000401467 |  |
| PSG8 | ENSG00000124467 | ENST00000306511 |  |
|  |  | ENST00000418820 |  |
|  |  | ENST00000621109 |  |
|  |  | ENST00000593948 |  |
| PSG9 | ENSG00000183668 | ENST00000270077 |  |
|  |  | ENST00000443718 |  |
|  |  | ENST00000244293 |  |
|  |  | ENST00000560217 |  |
|  |  | ENST00000044462 |  |
|  |  | ENST00000559146 |  |
| PSMA4 | ENSG00000041357 | ENST00000560737 | (GGT)3 |
|  |  | ENST00000555764 |  |
|  |  | ENST00000621781 |  |
|  |  | ENST00000543772 | (CT)3 |
| PSMA6 | ENSG00000100902 | ENST00000547221 | (CA)3 |
| PSMB2 | ENSG00000126067 | ENST00000619300 | (CCT)3 |
| PSMC1 | ENSG00000100764 | ENST00000617202 | (AG)3 |
| PSORS1C1 | ENSG00000204540 | ENST00000277462 | (CA)3 |
| PSPC1 | ENSG00000121390 | ENST00000597185 | (TCT)3 |
| PTGES2 | ENSG00000148334 | ENST00000594275 | (TCA)3 |
|  |  | ENST00000309195 |  |
| PTGIR | ENSG00000160013 | ENST00000553813 | (CAG)3 |
|  |  | ENST00000313049 | (CT)3 |
| PTGR1 | ENSG00000106853 | ENST00000523388 | (CG)3 |
| PTGR2 | ENSG00000140043 | ENST00000517339 | (CA)3 |
| PTH1R | ENSG00000160801 | ENST00000427191 | (ACC)3 |
| PTK2 | ENSG00000169398 | ENST00000436978 | (TC)3 |
| PTK2B | ENSG00000120899 | ENST00000502971 | (TG)3 |
| PTPN13 | ENSG00000163629 | ENST00000591497 | (GTG)3 |
|  |  | ENST00000613246 |  |
|  |  | ENST00000418331 |  |
| PTPN2 | ENSG00000175354 | ENST00000615445 | (GA)3 |
| PTPRJ | ENSG00000149177 | ENST00000440289 | (GGA)3 |
|  |  | ENST00000534219 |  |
|  |  | ENST00000527952 |  |
|  |  | ENST00000580170 |  |
|  |  |  |  |
|  |  | ENST00000332175 |  |
| PTPRM | ENSG00000173482 | ENST00000389416 | (CG)3 |
|  |  |  | (CCG)3 |
|  |  | ENST00000373190 | (CG)3 |
|  |  |  | (CCG)3 |
| PTPRN2 | ENSG00000155093 | ENST00000373198 | (CG)3 |
| PTPRT | ENSG00000196090 | ENST00000373201 |  |
|  |  |  | (GCC)3 |
|  |  | ENST00000373193 | (CG)3 |
|  |  |  | (GCC)3 |
|  |  | ENST00000520452 | (CG)3 |
|  |  |  | (GCC)3 |
|  |  | ENST00000373741 | (CG)3 |
|  |  |  | (GCC)3 |
| PTTG1 | ENSG00000164611 | ENST00000535067 | (CG)3 |
| PUM1 | ENSG00000134644 | ENST00000416848  ENST00000356297 |  |
|  |  |  | (CA)3 |
| PUS1 | ENSG00000177192 |  | (GCCCA)3 |
| PUS7L | ENSG00000129317 | ENST00000544436 | (CAG)3 |
| PXDNL | ENSG00000147485 | ENST00000537124  ENST00000395384 | (CAC)3 |
|  |  |  | (GCT)3 |
| QKI | ENSG00000112531 | ENST00000452814 | (GTTT)3 |
|  |  | ENST00000409822 | (CTG)3 |
| QPRT | ENSG00000103485 | ENST00000568803 | (CCCT)3 |
| RAB11FIP3 | ENSG00000090565 | ENST00000269347 | (GT)3 |
| RAB17 | ENSG00000124839 | ENST00000509895 | (GGA)3 |
| RAB27A | ENSG00000069974 | ENST00000601078 | (AATGC)3 |
| RAB40B | ENSG00000141542 | ENST00000616002 | (TGC)3 |
| RAB41 | ENSG00000147127 | ENST00000325589 | (GGA)3 |
| RABAC1 | ENSG00000105404 | ENST00000393167 | (TG)3 |
| RABGAP1 | ENSG00000011454 | ENST00000409842 | (AC)3 |
| RABGAP1L | ENSG00000152061 |  | (AT)3 |
| RABL2A | ENSG00000144134 | ENST00000413545 | (TC)3 |
|  |  | ENST00000393165 |  |
|  |  |  | (CA)3 |
|  |  | ENST00000393166  ENST00000409875 | (TC)3 |
|  |  |  | (CA)3 |
|  |  | ENST00000435118 | (TC)3 |
|  |  | ENST00000354869 |  |
|  |  |  | (TG)3 |
| RABL2B | ENSG00000079974 | ENST00000395593 | (TC)3 |
|  |  | ENST00000395598  ENST00000395591 |  |
|  |  |  | (CA)3 |
|  |  |  | (TG)3 |
|  |  | ENST00000395590 | (TC)3 |
|  |  | ENST00000548247  ENST00000523986 |  |
|  |  |  | (CA)3 |
|  |  | ENST00000461271 | (TC)3 |
| RACGAP1 | ENSG00000161800 | ENST00000541619 | (GA)3 |
| RAD21 | ENSG00000164754 | ENST00000523839 | (AT)3 |
| RAD51C | ENSG00000108384 | ENST00000469835 | (CA)3 |
| RAD52 | ENSG00000002016 | ENST00000395840 | (AC)3 |
| RAD54B | ENSG00000197275 | ENST00000514873 | (GAA)3 |
| RAD54L | ENSG00000085999 | ENST00000512305 | (GC)3 |
| RAE1 | ENSG00000101146 | ENST00000458039 | (TA)3 |
| RAI14 | ENSG00000039560 | ENST00000448364 | (CG)3 |
|  |  | ENST00000541630 | (GCA)3 |
| RALBP1 | ENSG00000017797 | ENST00000011619 | (TG)3 |
| RALY | ENSG00000125970 | ENST00000356415 | (AG)3 |
| RAN | ENSG00000132341 | ENST00000369709 | (GA)3 |
| RANBP9 | ENSG00000010017 | ENST00000495204 |  |
| RAP1A | ENSG00000116473 | ENST00000540393 | (AG)3 |
|  |  |  | (TGGAGGAGG)3 |
| RAP1GAP | ENSG00000076864 | ENST00000541245 | (GCG)3 |
| RAP1GAP2 | ENSG00000132359 | ENST00000517963 | (CA)3 |
|  |  |  | (GA)3 |
| RAPGEFL1 | ENSG00000108352 | ENST00000567686 | (GT)3 |
| RB1CC1 | ENSG00000023287 | ENST00000468092  ENST00000360790 | (GGCG)3 |
|  |  |  | (GGC)3 |
| RBBP6 | ENSG00000122257 | ENST00000581687 | (CA)3 |
| RBBP7 | ENSG00000102054 |  | (GAA)3 |
| RBBP8 | ENSG00000101773 | ENST00000353660 | (AG)3 |
|  |  | ENST00000400247  ENST00000547338 |  |
|  |  |  | (GA)3 |
| RBCK1 | ENSG00000125826 | ENST00000580508 | (CA)3 |
|  |  |  |  |
| RBFOX1 | ENSG00000078328 | ENST00000446108 | (GT)3 |
| RBFOX3 | ENSG00000167281 | ENST00000425446  ENST00000434927 | (CG)3 |
|  |  |  | (GCC)3 |
| RBM17 | ENSG00000134453 | ENST00000316997 | (CT)3 |
| RBM24 | ENSG00000112183 | ENST00000611254 | (GT)3 |
| RBM39 | ENSG00000131051 | ENST00000421682 |  |
| RBM44 | ENSG00000177483 | ENST00000545678 | (GCG)3 |
|  |  | ENST00000507574 | (AG)3 |
| RBM6 | ENSG00000004534 | ENST00000381135 | (GA)3 |
| RBM7 | ENSG00000076053 | ENST00000371374 | (CT)3 |
| RBPJ | ENSG00000168214 |  | (TA)3 |
| RCAN1 | ENSG00000159200 | ENST00000486790 | (AG)3 |
| RCAN2 | ENSG00000172348 | ENST00000556618  ENST00000381249 | (CG)3 |
|  |  |  | (TCCGCC)3 |
| RCC1 | ENSG00000180198 | ENST00000527874 | (GC)3 |
| RCCD1 | ENSG00000166965 | ENST00000393580 | (GAG)3 |
| RDH14 | ENSG00000240857 | ENST00000638118 | (GGC)3 |
| RELA | ENSG00000173039 |  | (GT)3 |
| RELT | ENSG00000054967 | ENST00000170168 | (CG)3 |
| REN | ENSG00000143839 | ENST00000448497  ENST00000449641 | (CA)3 |
|  |  |  |  |
| REXO1 | ENSG00000079313 | ENST00000537315 | (GCG)3 |
| RFC4 | ENSG00000163918 | ENST00000290524 | (ACC)3 |
| RFC5 | ENSG00000111445 | ENST00000368870 | (TA)3 |
|  |  | ENST00000452671 |  |
| RFX5 | ENSG00000143390 | ENST00000422595 | (AT)3 |
|  |  | ENST00000450506 |  |
|  |  | ENST00000430227 |  |
|  |  | ENST00000412774 |  |
|  |  | ENST00000436271 |  |
|  |  | ENST00000290691 |  |
|  |  |  |  |
|  |  | ENST00000423392 |  |
| RGL4 | ENSG00000159496 | ENST00000520117 | (GA)3 |
|  |  |  | (TCC)3 |
|  |  | ENST00000374136  ENST00000620489 | (GA)3 |
|  |  |  | (TCC)3 |
| RGS22 | ENSG00000132554 | ENST00000262316 | (CCA)3 |
| RGS3 | ENSG00000138835 | ENST00000536287 | (TG)3 |
|  |  | ENST00000294413 |  |
| RHBDF1 | ENSG00000007384 | ENST00000349320 | (GCGG)3 |
| RHBDL3 | ENSG00000141314 | ENST00000328664 | (GT)3 |
| RHCE | ENSG00000188672 | ENST00000622561 | (GA)3 |
|  |  | ENST00000445425 | (TG)3 |
| RHD | ENSG00000187010 | ENST00000414971 | (GA)3 |
|  |  | ENST00000620804 |  |
| RHOA | ENSG00000067560 | ENST00000433039 |  |
| RHOC | ENSG00000155366 | ENST00000537189 | (AG)3 |
| RIMBP3B | ENSG00000274600 | ENST00000609492 | (CGG)3 |
| RIMBP3C | ENSG00000183246 | ENST00000381627 |  |
| RIMKLB | ENSG00000166532 | ENST00000557105 | (GC)3 |
| RIT1 | ENSG00000143622 | ENST00000221486 | (CCT)3 |
| RLN2 | ENSG00000107014 | ENST00000263895 | (GT)3 |
| RNASE11 | ENSG00000173464 | ENST00000559160 | (GAA)3 |
| RNASEH2A | ENSG00000104889 | ENST00000470151 | (TGG)3 |
| RND3 | ENSG00000115963 | ENST00000521389 | (CTC)3 |
| RNF111 | ENSG00000157450 | ENST00000261947 | (ATTT)3 |
| RNF13 | ENSG00000082996 | ENST00000521606 | (GA)3 |
| RNF130 | ENSG00000113269 | ENST00000306799 | (GCC)3 |
|  |  | ENST00000507500 |  |
| RNF145 | ENSG00000145860 | ENST00000524954 | (TG)3 |
| RNF150 | ENSG00000170153 | ENST00000441031 | (AGC)3 |
|  |  | ENST00000416588 |  |
| RNF170 | ENSG00000120925 | ENST00000478815 | (CG)3 |
| RNF183 | ENSG00000165188 | ENST00000297894 | (GT)3 |
|  |  | ENST00000375121 |  |
|  |  | ENST00000453650 |  |
|  |  | ENST00000432459 |  |
| RNF186 | ENSG00000178828 | ENST00000554606 | (CT)3 |
| RNF2 | ENSG00000121481 | ENST00000525801 | (CA)3 |
| RNF32 | ENSG00000105982 | ENST00000547686 | (GC)3 |
| RNF34 | ENSG00000170633 | ENST00000551593 | (GA)3 |
| ROM1 | ENSG00000149489 | ENST00000573448 |  |
| RPH3A | ENSG00000089169 | ENST00000298283 | (CA)3 |
|  |  |  | (CG)3 |
| RPH3AL | ENSG00000181031 |  | (AG)3 |
| RPL10L | ENSG00000165496 | ENST00000467736  ENST00000422218  ENST00000326092 | (TA)3 |
|  |  |  | (GC)3 |
|  |  |  | (AG)3 |
| RPL13 | ENSG00000167526 | ENST00000589037 | (TTC)3 |
| RPL15 | ENSG00000174748 | ENST00000530913 | (AG)3 |
| RPL21 | ENSG00000122026 | ENST00000466397 | (CT)3 |
| RPL27 | ENSG00000131469 | ENST00000495383 | (CA)3 |
| RPL27A | ENSG00000166441 |  | (GT)3 |
| RPL29 | ENSG00000162244 | ENST00000492277 |  |
|  |  | ENST00000401609  ENST00000402527 | (CG)3 |
|  |  |  | (GT)3 |
|  |  | ENST00000553110 |  |
| RPL3 | ENSG00000100316 | ENST00000296277 | (GCA)3 |
|  |  | ENST00000396466 |  |
| RPL36A | ENSG00000241343 | ENST00000396465 | (TA)3 |
| RPL39L | ENSG00000163923 |  | (GA)3 |
| RPL7 | ENSG00000147604 | ENST00000435330 |  |
|  |  | ENST00000426651  ENST00000392514 | (AG)3 |
|  |  |  | (AAG)3 |
|  |  | ENST00000456102 | (GA)3 |
| RPL7A | ENSG00000148303 | ENST00000378202 | (AG)3 |
| RPLP0 | ENSG00000089157 | ENST00000322329 | (CG)3 |
| RPN2 | ENSG00000118705 | ENST00000586656 | (GA)3 |
| RPP38 | ENSG00000152464 | ENST00000591804 | (CG)3 |
| RPRML | ENSG00000179673 | ENST00000617694 | (GC)3 |
| RPS15 | ENSG00000115268 | ENST00000521262 | (TC)3 |
|  |  | ENST00000458478 |  |
|  |  | ENST00000424438 |  |
| RPS20 | ENSG00000008988 |  | (CGG)3 |
| RPSA | ENSG00000168028 | ENST00000531807 | (CT)3 |
| RPUSD3 | ENSG00000156990 | ENST00000442639  ENST00000573791 | (TC)3 |
|  |  |  | (CT)3 |
| RRAS2 | ENSG00000133818 | ENST00000539903 | (GA)3 |
| RSAD2 | ENSG00000134321 | ENST00000221538 | (CTC)3 |
| RSL1D1 | ENSG00000171490 | ENST00000517939 | (CT)3 |
| RSPH10B | ENSG00000155026 | ENST00000521502 | (TA)3 |
| RSPH6A | ENSG00000104941 | ENST00000521757 | (TC)3 |
| RSPO2 | ENSG00000147655 | ENST00000520026 | (TTC)3 |
|  |  | ENST00000562959 |  |
|  |  |  |  |
|  |  | ENST00000494002 | (GT)3 |
| RSPRY1 | ENSG00000159579 | ENST00000526560  ENST00000602389 | (TA)3 |
|  |  |  | (TC)3 |
| RSRC1 | ENSG00000174891 | ENST00000458671 | (GT)3 |
| RSRC2 | ENSG00000111011 | ENST00000315289 | (AG)3 |
| RSU1 | ENSG00000148484 | ENST00000578780 | (CA)3 |
| RTBDN | ENSG00000132026 | ENST00000497930 | (AG)3 |
| RTKN2 | ENSG00000182010 | ENST00000506467 | (AC)3 |
| RTTN | ENSG00000176225 | ENST00000368698 | (GA)3 |
| RUSC1 | ENSG00000160753 | ENST00000368710 | (CTG)3 |
| RWDD4 | ENSG00000182552 | ENST00000368732 | (TA)3 |
| S100A1 | ENSG00000160678 | ENST00000532858 | (AC)3 |
| S100A2 | ENSG00000196754 | ENST00000405158 | (GA)3 |
| S100A8 | ENSG00000143546 | ENST00000536229 |  |
| SAA1 | ENSG00000173432 | ENST00000533683 | (AG)3 |
|  |  | ENST00000620985 |  |
| SALL3 | ENSG00000256463 | ENST00000538735 | (GC)3 |
| SAMD1 | ENSG00000141858 | ENST00000614631 |  |
| SAMD9 | ENSG00000205413 | ENST00000373239 | (AT)3 |
| SAP25 | ENSG00000205307 | ENST00000550619 | (GCT)3 |
|  |  | ENST00000491519 |  |
| SAR1A | ENSG00000079332 | ENST00000452260 | (TC)3 |
| SART3 | ENSG00000075856 |  | (AG)3 |
| SATB1 | ENSG00000182568 | ENST00000415069 | (TG)3 |
|  |  | ENST00000414509  ENST00000360565 | (GA)3 |
|  |  |  | (AT)3 |
|  |  | ENST00000411971 | (GAG)3 |
|  |  | ENST00000305978 | (TA)3 |
| SCAF1 | ENSG00000126461 | ENST00000615116 | (CT)3 |
| SCAMP4 | ENSG00000227500 | ENST00000373991 | (CA)3 |
| SCAND1 | ENSG00000171222 | ENST00000636985 |  |
|  |  | ENST00000527836 |  |
|  |  | ENST00000638820 |  |
| SCN2A | ENSG00000136531 | ENST00000540037 | (TC)3 |
| SCN3B | ENSG00000166257 | ENST00000379116 | (GC)3 |
| SCN8A | ENSG00000196876 | ENST00000470022 | (GCGG)3 |
| SCNN1A | ENSG00000111319 |  | (GGA)3 |
| SCNN1D | ENSG00000162572 |  | (AG)3 |
|  |  | ENST00000325425 |  |
|  |  |  | (CA)3 |
|  |  |  |  |
|  |  | ENST00000630739  ENST00000336798  ENST00000339394 | (AG)3 |
|  |  |  | (CA)3 |
|  |  |  |  |
| SCTR | ENSG00000080293 |  | (TG)3 |
| SDC3 | ENSG00000162512 |  | (AG)3 |
|  |  | ENST00000381812  ENST00000553930  ENST00000554698 | (CGC)3 |
|  |  |  | (CGCC)3 |
|  |  |  | (CGC)3 |
| SDCBP2 | ENSG00000125775 | ENST00000555365 | (TCC)3 |
| SDR39U1 | ENSG00000100445 | ENST00000553343 | (GGT)3 |
|  |  | ENST00000555225 | (GA)3 |
|  |  | ENST00000556523 | (GGT)3 |
|  |  | ENST00000429917 | (GA)3 |
|  |  | ENST00000415072 | (GGT)3 |
|  |  | ENST00000251170 |  |
| SEC14L2 | ENSG00000100003 | ENST00000313084 | (GT)3 |
|  |  | ENST00000537403 | (CT)3 |
| SEC14L5 | ENSG00000103184 |  | (AC)3 |
| SEC16A | ENSG00000148396 | ENST00000504968 | (GT)3 |
| SEC23A | ENSG00000100934 | ENST00000368286  ENST00000518124 | (AG)3 |
|  |  |  | (TA)3 |
| SEC24B | ENSG00000138802 | ENST00000586582 | (CT)3 |
| SEMA4A | ENSG00000196189 | ENST00000394085 | (CTT)3 |
| SEMA4G | ENSG00000095539 |  | (CG)3 |
| SEMA6B | ENSG00000167680 |  | (CCT)3 |
| SENP7 | ENSG00000138468 | ENST00000567794  ENST00000564863  ENST00000564082 | (AT)3 |
|  |  |  |  |
|  |  |  |  |
| SENP8 | ENSG00000166192 | ENST00000507348 | (GAG)3 |
|  |  | ENST00000512649 |  |
|  |  | ENST00000515588 |  |
| SERF1A | ENSG00000172058 | ENST00000409614 | (AC)3 |
|  |  | ENST00000532265 |  |
| SERF1B | ENSG00000205572 | ENST00000529728 |  |
| SERF2 | ENSG00000140264 | ENST00000530613 | (GT)3 |
| SERGEF | ENSG00000129158 | ENST00000532389 | (CA)3 |
|  |  | ENST00000557598 |  |
|  |  | ENST00000448305 |  |
|  |  | ENST00000546329 |  |
| SERPINA5 | ENSG00000188488 | ENST00000283752 | (GA)3 |
| SERPINA9 | ENSG00000170054 | ENST00000397985 | (TCT)3 |
|  |  | ENST00000353706 | (AT)3 |
| SERPINB3 | ENSG00000057149 | ENST00000636430 | (AC)3 |
| SERPINB8 | ENSG00000166401 | ENST00000397988 | (GCAGCA)3 |
|  |  | ENST00000432738 |  |
|  |  | ENST00000453066 |  |
|  |  | ENST00000324015 |  |
| SERPINE2 | ENSG00000135919 | ENST00000450523 | (CA)3 |
| SERPINF2 | ENSG00000167711 | ENST00000529643 | (AG)3 |
|  |  | ENST00000357949 |  |
|  |  | ENST00000409104 |  |
| SERPINH1 | ENSG00000149257 | ENST00000322030 | (TGCG)3 |
| SERTAD1 | ENSG00000197019 | ENST00000638947 | (GCC)3 |
| SET | ENSG00000119335 | ENST00000399212 | (GAG)3 |
|  |  | ENST00000399205 | (TC)3 |
| SETD2 | ENSG00000181555 | ENST00000399201 | (AG)3 |
| SETD4 | ENSG00000185917 | ENST00000399207 | (GA)3 |
|  |  | ENST00000446166 |  |
|  |  | ENST00000442559 |  |
|  |  | ENST00000608795 | (GC)3 |
|  |  |  | (GA)3 |
|  |  |  |  |
| SETD7 | ENSG00000145391 | ENST00000358065  ENST00000339276 | (AC)3 |
|  |  |  | (GGA)3 |
|  |  |  | (AC)3 |
| SETMAR | ENSG00000170364 | ENST00000379845 | (CG)3 |
| SFN | ENSG00000175793 | ENST00000634371  ENST00000457767 | (GA)3 |
|  |  |  | (TG)3 |
| SFRP1 | ENSG00000104332 | ENST00000381007 | (AG)3 |
| SFTA2 | ENSG00000196260 | ENST00000518040 | (TC)3 |
| SGSM3 | ENSG00000100359 | ENST00000517430 | (GT)3 |
| SGTB | ENSG00000197860 |  | (CAG)3 |
| SH2D4A | ENSG00000104611 | ENST00000452765 | (CCCT)3 |
| SH2D5 | ENSG00000189410 | ENST00000503219  ENST00000502260 | (CGG)3 |
|  |  |  | (AC)3 |
| SH3BP2 | ENSG00000087266 | ENST00000324537 | (GCC)3 |
|  |  | ENST00000403657 |  |
|  |  |  | (GCA)3 |
| SH3GL3 | ENSG00000140600 | ENST00000396535 | (CT)3 |
| SH3YL1 | ENSG00000035115 | ENST00000558220  ENST00000442747 | (CA)3 |
|  |  |  |  |
| SHC4 | ENSG00000185634 | ENST00000482706 | (GA)3 |
|  |  | ENST00000531353 | (AT)3 |
| SHISA5 | ENSG00000164054 | ENST00000270162 | (CCG)3 |
| SIAH2 | ENSG00000181788 |  | (GT)3 |
| SIDT2 | ENSG00000149577 | ENST00000644750 |  |
| SIK1 | ENSG00000142178 | ENST00000508639  ENST00000534313 | (GCA)3 |
|  |  |  | (GC)3 |
|  |  | ENST00000563840 |  |
| SIL1 | ENSG00000120725 | ENST00000579336 | (TG)3 |
| SIPA1 | ENSG00000213445 |  | (GCG)3 |
| SIRPB1 | ENSG00000101307 | ENST00000476188 | (TC)3 |
| SKAP1 | ENSG00000141293 | ENST00000426052  ENST00000368092 | (AG)3 |
|  |  |  | (TC)3 |
| SKIL | ENSG00000136603 | ENST00000546743 | (AC)3 |
|  |  | ENST00000547732 |  |
| SLAMF9 | ENSG00000162723 | ENST00000587601 | (ACTG)3 |
| SLC11A2 | ENSG00000110911 | ENST00000502059 | (GAG)3 |
|  |  | ENST00000586951 |  |
| SLC14A1 | ENSG00000141469 | ENST00000402943 | (CTGG)3 |
|  |  | ENST00000590246 | (GC)3 |
|  |  | ENST00000447225 |  |
|  |  | ENST00000412034 | (CTGG)3 |
|  |  | ENST00000425822 |  |
| SLC16A11 | ENSG00000174326 | ENST00000587091 | (TC)3 |
| SLC16A14 | ENSG00000163053 | ENST00000580098 | (AC)3 |
|  |  | ENST00000392339 |  |
| SLC16A2 | ENSG00000147100 | ENST00000567670 | (GGCAGC)3 |
| SLC16A3 | ENSG00000141526 | ENST00000443742 | (GGC)3 |
|  |  | ENST00000258403 |  |
| SLC19A1 | ENSG00000173638 | ENST00000646591 | (GA)3 |
|  |  | ENST00000412532 |  |
| SLC19A3 | ENSG00000135917 | ENST00000598022 | (AGAA)3 |
|  |  | ENST00000540443 | (AC)3 |
| SLC1A5 | ENSG00000105281 | ENST00000473690 | (AG)3 |
|  |  | ENST00000434949 |  |
| SLC22A1 | ENSG00000175003 | ENST00000437378 | (CT)3 |
| SLC22A12 | ENSG00000197891 | ENST00000424139 | (GT)3 |
| SLC22A16 | ENSG00000004809 | ENST00000625099 | (AG)3 |
|  |  | ENST00000526203 |  |
|  |  | ENST00000328041 |  |
| SLC22A18AS | ENSG00000254827 |  | (GA)3 |
|  |  |  | (GT)3 |
| SLC24A3 | ENSG00000185052 | ENST00000393265  ENST00000531433  ENST00000545862 | (CG)3 |
|  |  |  | (GC)3 |
|  |  |  | (GCC)3 |
|  |  |  |  |
| SLC24A4 | ENSG00000140090 | ENST00000331531 | (TC)3 |
|  |  | ENST00000580273 | (AG)3 |
| SLC25A10 | ENSG00000183048 | ENST00000331299 | (CGCGGGG)3 |
|  |  | ENST00000370041 |  |
| SLC25A19 | ENSG00000125454 | ENST00000603486 | (AGG)3 |
| SLC25A21 | ENSG00000183032 |  | (CT)3 |
| SLC25A24 | ENSG00000085491 | ENST00000359232 | (GT)3 |
| SLC25A27 | ENSG00000153291 | ENST00000392908  ENST00000554060 | (TG)3 |
|  |  |  | (AG)3 |
| SLC25A29 | ENSG00000197119 | ENST00000320442 | (CG)3 |
|  |  | ENST00000440056 |  |
|  |  | ENST00000358747 |  |
| SLC26A10 | ENSG00000135502 | ENST00000394602 | (GA)3 |
| SLC26A4 | ENSG00000091137 | ENST00000544960 | (GC)3 |
| SLC26A6 | ENSG00000225697 | ENST00000393844 | (TC)3 |
| SLC26A8 | ENSG00000112053 | ENST00000434816 | (GAAT)3 |
| SLC27A2 | ENSG00000140284 | ENST00000396872 | (AG)3 |
| SLC29A1 | ENSG00000112759 | ENST00000444741 | (CAG)3 |
| SLC29A4 | ENSG00000164638 | ENST00000297195 | (AG)3 |
|  |  | ENST00000406453 | (GGCCG)3 |
|  |  | ENST00000610552 |  |
|  |  | ENST00000451404 |  |
|  |  | ENST00000373352 |  |
| SLC2A8 | ENSG00000136856 |  | (TG)3 |
|  |  | ENST00000439597 |  |
|  |  | ENST00000423934  ENST00000621204 |  |
|  |  |  | (GTC)3 |
|  |  | ENST00000504577 | (TC)3 |
|  |  | ENST00000494706 |  |
| SLC30A5 | ENSG00000145740 |  | (AG)3 |
| SLC34A1 | ENSG00000131183 | ENST00000317734 |  |
| SLC35A5 | ENSG00000138459 | ENST00000360388  ENST00000532000 | (TG)3 |
|  |  |  |  |
| SLC35C2 | ENSG00000080189 | ENST00000526105 | (CGG)3 |
| SLC35F1 | ENSG00000196376 | ENST00000412905 | (CGC)3 |
| SLC37A2 | ENSG00000134955 | ENST00000255559 | (CTG)3 |
| SLC38A6 | ENSG00000139974 | ENST00000542342 | (TG)3 |
| SLC39A10 | ENSG00000196950 | ENST00000579732 | (GA)3 |
| SLC39A11 | ENSG00000133195 | ENST00000524285 |  |
|  |  | ENST00000409380 |  |
|  |  | ENST00000367137 |  |
| SLC39A14 | ENSG00000104635 | ENST00000529113 | (AG)3 |
| SLC3A1 | ENSG00000138079 | ENST00000446120 | (GT)3 |
| SLC41A1 | ENSG00000133065 | ENST00000271227 | (AGA)3 |
| SLC43A3 | ENSG00000134802 | ENST00000483786 | (TG)3 |
| SLC44A3 | ENSG00000143036 | ENST00000461735 | (AGCCCC)3 |
|  |  | ENST00000639096 | (GCCCCG)3 |
| SLC4A2 | ENSG00000164889 | ENST00000535225 | (GA)3 |
|  |  | ENST00000358657 | (GCCG)3 |
| SLC4A4 | ENSG00000080493 | ENST00000552192 | (AGA)3 |
| SLC4A8 | ENSG00000050438 | ENST00000551612 | (GAG)3 |
|  |  | ENST00000452775 |  |
| SLC6A15 | ENSG00000072041 | ENST00000528803 | (AC)3 |
|  |  | ENST00000565855 |  |
| SLC6A6 | ENSG00000131389 | ENST00000566198 | (TG)3 |
| SLC6A9 | ENSG00000196517 | ENST00000566345 | (AC)3 |
| SLC9A3R2 | ENSG00000065054 | ENST00000413682 | (AG)3 |
|  |  | ENST00000421287 |  |
| SLC9A5 | ENSG00000135740 | ENST00000530556 | (GC)3 |
| SLCO1A2 | ENSG00000084453 | ENST00000527180 | (TG)3 |
|  |  | ENST00000394566 | (ATC)3 |
| SLCO2B1 | ENSG00000137491 | ENST00000591682 | (CA)3 |
|  |  | ENST00000441608 | (AGATGG)3 |
| SLFN11 | ENSG00000172716 | ENST00000589811 | (TC)3 |
|  |  | ENST00000430814 |  |
|  |  | ENST00000427966 |  |
|  |  | ENST00000588579 |  |
|  |  | ENST00000592122 |  |
|  |  | ENST00000622093 |  |
|  |  | ENST00000590061 |  |
|  |  | ENST00000425815 |  |
| SLIT2 | ENSG00000145147 | ENST00000323347 | (GA)3 |
| SMAD4 | ENSG00000141646 | ENST00000286398 | (GTC)3 |
| SMARCAL1 | ENSG00000138375 | ENST00000485867 | (GGA)3 |
| SMARCD2 | ENSG00000108604 | ENST00000369592 | (GT)3 |
| SMC2 | ENSG00000136824 | ENST00000539041 | (GGT)3 |
| SMC4 | ENSG00000113810 | ENST00000443900 | (TCC)3 |
| SMNDC1 | ENSG00000119953 | ENST00000568859 | (CG)3 |
| SMPDL3A | ENSG00000172594 | ENST00000617596 | (TCC)3 |
| SMYD5 | ENSG00000135632 | ENST00000439399 | (AG)3 |
| SNAP23 | ENSG00000092531 | ENST00000521743 | (CT)3 |
| SNAP47 | ENSG00000143740 |  | (CA)3 |
| SNAP91 | ENSG00000065609 |  | (CCG)3 |
|  |  | ENST00000261367  ENST00000374018  ENST00000374017 | (AG)3 |
|  |  |  | (GCG)3 |
|  |  |  | (GGC)3 |
| SNCAIP | ENSG00000064692 | ENST00000391932 | (TA)3 |
| SNRPC | ENSG00000124562 | ENST00000643809 | (AC)3 |
|  |  | ENST00000517473 | (GTTT)3 |
| SNRPD2 | ENSG00000125743 | ENST00000560829 | (AG)3 |
| SNTG1 | ENSG00000147481 | ENST00000559061 |  |
|  |  | ENST00000396376 |  |
| SNX1 | ENSG00000028528 | ENST00000581298 | (GAA)3 |
|  |  | ENST00000584335 |  |
| SNX10 | ENSG00000086300 | ENST00000374274 | (GA)3 |
| SNX11 | ENSG00000002919 | ENST00000377759 | (GCA)3 |
|  |  | ENST00000419004 | (CT)3 |
| SNX12 | ENSG00000147164 | ENST00000606557 | (CCG)3 |
| SNX5 | ENSG00000089006 | ENST00000606602 | (CTT)3 |
|  |  | ENST00000486039 |  |
|  |  | ENST00000435336 |  |
|  |  | ENST00000492542 |  |
|  |  | ENST00000612154 |  |
| SNX8 | ENSG00000106266 |  | (AG)3 |
| SORBS1 | ENSG00000095637 | ENST00000622431 |  |
| SORCS1 | ENSG00000108018 | ENST00000344440 |  |
|  |  |  | (TG)3 |
|  |  | ENST00000451331 | (AG)3 |
|  |  |  | (TG)3 |
|  |  | ENST00000370536  ENST00000529469 | (AG)3 |
|  |  |  | (TG)3 |
| SOS1 | ENSG00000115904 | ENST00000637943 | (ATG)3 |
| SOX3 | ENSG00000134595 | ENST00000635868 | (TA)3 |
| SOX6 | ENSG00000110693 |  | (GT)3 |
| SP2 | ENSG00000167182 | ENST00000342234 | (GAGGA)3 |
|  |  | ENST00000580599  ENST00000400125 | (AGG)3 |
|  |  |  | (CA)3 |
| SP6 | ENSG00000189120 | ENST00000434307 | (GCC)3 |
| SPACA3 | ENSG00000141316 | ENST00000642566 | (GTG)3 |
| SPAG11A | ENSG00000178287 | ENST00000543631 | (CA)3 |
|  |  | ENST00000439395 |  |
|  |  | ENST00000377185 |  |
| SPARCL1 | ENSG00000152583 |  | (GAAAA)3 |
| SPATS2L | ENSG00000196141 | ENST00000332397 | (AGCA)3 |
| SPDYC | ENSG00000204710 | ENST00000583482 | (TA)3 |
|  |  |  | (GCG)3 |
| SPDYE3 | ENSG00000214300 | ENST00000583463  ENST00000582604 | (AC)3 |
|  |  |  | (AGA)3 |
| SPECC1 | ENSG00000128487 | ENST00000581973 | (CAC)3 |
|  |  | ENST00000433215 | (GT)3 |
|  |  | ENST00000601704 |  |
|  |  | ENST00000551346 | (TG)3 |
| SPG21 | ENSG00000090487 | ENST00000374908 |  |
| SPHK2 | ENSG00000063176 |  | (GAG)3 |
| SPIC | ENSG00000166211 | ENST00000374906 | (TTATT)3 |
| SPIN2A | ENSG00000147059 | ENST00000379725  ENST00000511717 | (TC)3 |
|  |  |  | (CT)3 |
|  |  | ENST00000587090 | (AC)3 |
| SPINK4 | ENSG00000122711 | ENST00000592007 | (GA)3 |
| SPINK9 | ENSG00000204909 | ENST00000409402 | (TC)3 |
| SPINT2 | ENSG00000167642 | ENST00000410092 | (CG)3 |
|  |  | ENST00000257100 | (GA)3 |
| SPIRE1 | ENSG00000134278 | ENST00000528791 | (CGA)3 |
|  |  | ENST00000394945 |  |
| SPOCD1 | ENSG00000134668 | ENST00000505690 | (TG)3 |
|  |  | ENST00000421836 |  |
| SPOCK1 | ENSG00000152377 | ENST00000541354 | (CGG)3 |
|  |  | ENST00000425558 | (GC)3 |
| SPOCK3 | ENSG00000196104 | ENST00000587013 | (TC)3 |
|  |  | ENST00000587947 |  |
| SPP2 | ENSG00000072080 | ENST00000307098 | (GAT)3 |
| SPRED3 | ENSG00000188766 | ENST00000331860 | (ACC)3 |
|  |  | ENST00000443178 | (AGA)3 |
| SPRR1B | ENSG00000169469 | ENST00000505319 | (CT)3 |
| SPRR3 | ENSG00000163209 | ENST00000610581 | (GT)3 |
|  |  | ENST00000394339 |  |
| SPRY1 | ENSG00000164056 | ENST00000569769 | (CT)3 |
|  |  | ENST00000523430 |  |
|  |  | ENST00000521232 | (GT)3 |
| SPSB3 | ENSG00000162032 | ENST00000264228 | (CTGC)3 |
| SQLE | ENSG00000104549 | ENST00000543397 | (GCC)3 |
|  |  | ENST00000572793 |  |
| SRD5A3 | ENSG00000128039 | ENST00000558720 | (CG)3 |
| SRGAP1 | ENSG00000196935 | ENST00000560773 | (TC)3 |
| SRGAP2 | ENSG00000266028 | ENST00000307877 | (GAAA)3 |
| SRP14 | ENSG00000140319 | ENST00000640889 | (AAG)3 |
|  |  | ENST00000344595 |  |
| SRP68 | ENSG00000167881 | ENST00000576620 | (CGG)3 |
| SRPX2 | ENSG00000102359 | ENST00000574340 | (GAG)3 |
| SRR | ENSG00000167720 | ENST00000395136 | (GC)3 |
|  |  | ENST00000370949 |  |
| SRRM2 | ENSG00000167978 | ENST00000450482 | (AT)3 |
| SRSF11 | ENSG00000116754 | ENST00000597724 | (CT)3 |
|  |  |  | (GT)3 |
| SS18L1 | ENSG00000184402 | ENST00000594321 | (ACC)3 |
| SSBP4 | ENSG00000130511 | ENST00000409136 | (TG)3 |
|  |  |  |  |
| SSC5D | ENSG00000179954 | ENST00000326470  ENST00000546697 | (TCCC)3 |
|  |  |  | (TC)3 |
| SSFA2 | ENSG00000138434 | ENST00000592397 | (TG)3 |
| SSH1 | ENSG00000084112 | ENST00000582084 | (GAG)3 |
|  |  | ENST00000531405 | (GGA)3 |
| SSH2 | ENSG00000141298 | ENST00000527920 | (TC)3 |
|  |  | ENST00000376919 | (CCGC)3 |
| SSSCA1 | ENSG00000173465 | ENST00000603677 | (CT)3 |
|  |  | ENST00000298181 |  |
| SSX1 | ENSG00000126752 | ENST00000562759 | (CTC)3 |
| SSX2IP | ENSG00000117155 | ENST00000478497 | (GAA)3 |
| SSX7 | ENSG00000187754 | ENST00000523854 | (CT)3 |
| ST20 | ENSG00000180953 | ENST00000517668 | (AG)3 |
|  |  | ENST00000265261 | (CA)3 |
| ST3GAL1 | ENSG00000008513 | ENST00000477574 |  |
|  |  | ENST00000485145 |  |
| ST3GAL6 | ENSG00000064225 | ENST00000528196 |  |
|  |  | ENST00000533681 | (AGG)3 |
|  |  | ENST00000530938 | (CA)3 |
| ST5 | ENSG00000166444 | ENST00000369669 | (CTG)3 |
|  |  | ENST00000521236 |  |
|  |  |  | (TG)3 |
| ST7L | ENSG00000007341 |  | (AG)3 |
| STAR | ENSG00000147465 | ENST00000580331  ENST00000581310  ENST00000252336 | (CT)3 |
|  |  |  | (TC)3 |
|  |  |  | (CT)3 |
| STARD3 | ENSG00000131748 | ENST00000585517 | (AG)3 |
| STARD6 | ENSG00000174448 | ENST00000588969 | (CCA)3 |
| STARD8 | ENSG00000130052 | ENST00000404395 | (GAA)3 |
| STAT3 | ENSG00000168610 | ENST00000588065 | (TC)3 |
|  |  | ENST00000543873 |  |
|  |  | ENST00000556155 |  |
|  |  | ENST00000554663 | (TG)3 |
| STAT6 | ENSG00000166888 | ENST00000347458 | (CT)3 |
|  |  | ENST00000371802 | (CAG)3 |
|  |  | ENST00000371792 | (CT)3 |
| STAU1 | ENSG00000124214 | ENST00000525055 | (AGC)3 |
|  |  | ENST00000532990 |  |
|  |  | ENST00000478049 |  |
| STIM1 | ENSG00000167323 | ENST00000523601 | (AG)3 |
|  |  |  | (TG)3 |
| STIM2 | ENSG00000109689 | ENST00000368620 | (AG)3 |
| STK3 | ENSG00000104375 | ENST00000368619  ENST00000399728 | (GA)3 |
|  |  |  | (CT)3 |
| STK32C | ENSG00000165752 | ENST00000535552 | (AG)3 |
|  |  | ENST00000563965 |  |
| STMN1 | ENSG00000117632 | ENST00000574278 | (CG)3 |
| STRA6 | ENSG00000137868 | ENST00000571341 | (CAG)3 |
|  |  | ENST00000527606 | (AC)3 |
|  |  | ENST00000529196 | (CT)3 |
|  |  | ENST00000507908 | (TCTT)3 |
| STT3A | ENSG00000134910 | ENST00000637521 | (GC)3 |
|  |  | ENST00000627871 | (GA)3 |
| STX18 | ENSG00000168818 | ENST00000414284 | (TCC)3 |
| STXBP1 | ENSG00000136854 | ENST00000602355 | (AG)3 |
|  |  | ENST00000396700 | (AAG)3 |
| STXBP2 | ENSG00000076944 | ENST00000601879 | (GGC)3 |
|  |  | ENST00000437955 | (GA)3 |
| STXBP6 | ENSG00000168952 | ENST00000350842 | (GT)3 |
| SUGP2 | ENSG00000064607 | ENST00000395607 | (CT)3 |
| SULF2 | ENSG00000196562 | ENST00000395630 | (GC)3 |
| SULT1A1 | ENSG00000196502 | ENST00000338971 | (CG)3 |
|  |  |  | (CA)3 |
| SULT1A2 | ENSG00000197165 | ENST00000360423 |  |
| SULT1A3 | ENSG00000261052 | ENST00000409498 | (AG)3 |
|  |  |  | (GA)3 |
| SULT1A4 | ENSG00000213648 | ENST00000409205  ENST00000405018 | (AG)3 |
|  |  |  | (GA)3 |
| SUMO1 | ENSG00000116030 | ENST00000394115 | (AT)3 |
|  |  | ENST00000552258 |  |
| SUN2 | ENSG00000100242 | ENST00000577713 | (GGC)3 |
| SUOX | ENSG00000139531 | ENST00000276646 | (GT)3 |
|  |  | ENST00000533171 |  |
| SUPT6H | ENSG00000109111 | ENST00000534184 | (TC)3 |
| SYBU | ENSG00000147642 | ENST00000602061 | (GCC)3 |
|  |  | ENST00000618464 | (CTT)3 |
|  |  | ENST00000638626 |  |
| SYMPK | ENSG00000125755 | ENST00000560674 | (GT)3 |
| SYNGR3 | ENSG00000127561 | ENST00000446242 | (ATCAGC)3 |
| SYNJ2 | ENSG00000078269 | ENST00000393946 | (ACG)3 |
| SYNM | ENSG00000182253 |  | (CA)3 |
| SYT1 | ENSG00000067715 | ENST00000430451 | (TA)3 |
| SYT12 | ENSG00000173227 | ENST00000367267  ENST00000589172 | (CA)3 |
|  |  |  | (AC)3 |
| SYT16 | ENSG00000139973 | ENST00000609117 | (AG)3 |
| SYT2 | ENSG00000143858 | ENST00000430303 | (GCCCCC)3 |
| SYT5 | ENSG00000129990 | ENST00000341958 | (GA)3 |
| SYT6 | ENSG00000134207 | ENST00000359152 | (GC)3 |
| SYT8 | ENSG00000149043 | ENST00000389960 | (CA)3 |
|  |  | ENST00000316356 | (GCT)3 |
| SYTL2 | ENSG00000137501 | ENST00000528231 | (GT)3 |
|  |  | ENST00000527523 |  |
|  |  |  |  |
|  |  | ENST00000532221 |  |
|  |  | ENST00000524911  ENST00000437822 | (AG)3 |
|  |  |  | (GC)3 |
|  |  | ENST00000529792 | (AG)3 |
|  |  | ENST00000530649 |  |
| TAF6 | ENSG00000106290 | ENST00000320307 | (TCC)3 |
| TAGLN | ENSG00000149591 | ENST00000616041 | (AG)3 |
|  |  | ENST00000514078 | (GA)3 |
| TAGLN2 | ENSG00000158710 | ENST00000506620 | (TC)3 |
| TARM1 | ENSG00000248385 | ENST00000247883 | (GAAA)3 |
| TAS2R1 | ENSG00000169777 | ENST00000240691 | (GTG)3 |
|  |  | ENST00000523152 |  |
| TAS2R5 | ENSG00000127366 | ENST00000572862 | (GT)3 |
| TAS2R9 | ENSG00000121381 | ENST00000513455 | (TCA)3 |
| TATDN1 | ENSG00000147687 |  | (CG)3 |
| TBC1D16 | ENSG00000167291 | ENST00000512840 | (AG)3 |
| TBC1D19 | ENSG00000109680 | ENST00000513596 |  |
|  |  |  |  |
|  |  | ENST00000375063  ENST00000620215 |  |
|  |  |  |  |
|  |  | ENST00000611257 | (AT)3 |
| TBC1D2 | ENSG00000095383 | ENST00000622206 | (CT)3 |
| TBC1D3 | ENSG00000274611 | ENST00000620210 | (AC)3 |
| TBC1D3B | ENSG00000274808 | ENST00000569055 |  |
| TBC1D3C | ENSG00000278299 | ENST00000610350 |  |
| TBC1D3F | ENSG00000275954 | ENST00000429924 |  |
| TBC1D3G | ENSG00000260287 |  |  |
| TBC1D3H | ENSG00000274226 | ENST00000415814 |  |
| TBC1D5 | ENSG00000131374 | ENST00000428355  ENST00000444471 | (TA)3 |
|  |  |  | (TC)3 |
|  |  | ENST00000356436 | (GC)3 |
|  |  |  |  |
|  |  | ENST00000343141 |  |
| TBC1D7 | ENSG00000145979 | ENST00000379307  ENST00000606370 | (GCG)3 |
|  |  |  | (GCAGCG)3 |
|  |  | ENST00000372876 | (CAGCGG)3 |
|  |  | ENST00000383032 |  |
|  |  | ENST00000355162 |  |
| TBCC | ENSG00000124659 | ENST00000346432 | (GA)3 |
| TBL1Y | ENSG00000092377 | ENST00000615855 | (CA)3 |
|  |  | ENST00000540980 |  |
|  |  | ENST00000416849 |  |
| TBL3 | ENSG00000183751 | ENST00000438104 | (AC)3 |
| TBP | ENSG00000112592 | ENST00000336425 | (CG)3 |
| TBXAS1 | ENSG00000059377 | ENST00000361317 | (AAC)3 |
|  |  | ENST00000557947 |  |
|  |  | ENST00000263347 |  |
| TCEA2 | ENSG00000171703 | ENST00000356073 | (GGA)3 |
| TCF12 | ENSG00000140262 |  | (CG)3 |
| TCF25 | ENSG00000141002 | ENST00000635822 | (GT)3 |
| TCF4 | ENSG00000196628 | ENST00000354452 | (GGC)3 |
|  |  |  |  |
|  |  | ENST00000565908 |  |
|  |  |  |  |
|  |  | ENST00000627784 |  |
|  |  |  |  |
|  |  | ENST00000520958 |  |
|  |  |  |  |
|  |  | ENST00000442813  ENST00000637574 |  |
|  |  |  |  |
| TCF7 | ENSG00000081059 | ENST00000217162 | (GT)3 |
| TCF7L1 | ENSG00000152284 | ENST00000617120 |  |
| TCF7L2 | ENSG00000148737 | ENST00000472557 | (GGA)3 |
| TCFL5 | ENSG00000101190 | ENST00000366832 | (CCG)3 |
| TCP10 | ENSG00000203690 | ENST00000418521 | (CT)3 |
| TCP10L | ENSG00000242220 | ENST00000553617 | (CAG)3 |
| TCP10L2 | ENSG00000166984 | ENST00000458431 | (CT)3 |
| TCP11 | ENSG00000124678 | ENST00000535814 |  |
| TDP1 | ENSG00000042088 |  | (TGA)3 |
| TDRKH | ENSG00000182134 | ENST00000374320 | (GTG)3 |
| TEX10 | ENSG00000136891 | ENST00000403298  ENST00000419396 | (GA)3 |
|  |  |  | (GT)3 |
| TEX11 | ENSG00000120498 | ENST00000419574 | (AG)3 |
| TFEB | ENSG00000112561 | ENST00000445214 | (GC)3 |
|  |  | ENST00000425401 |  |
|  |  | ENST00000433032 | (GTG)3 |
|  |  | ENST00000484212 |  |
|  |  | ENST00000463568 | (CG)3 |
|  |  | ENST00000549253 | (TC)3 |
| TFEC | ENSG00000105967 | ENST00000544573 | (GA)3 |
| TFG | ENSG00000114354 | ENST00000202625 | (CG)3 |
| TGIF1 | ENSG00000177426 | ENST00000381423 | (GCGCC)3 |
| TGM1 | ENSG00000092295 | ENST00000541990 | (TG)3 |
| TGM6 | ENSG00000166948 | ENST00000537166 |  |
|  |  | ENST00000419759 |  |
| THBS3 | ENSG00000169231 | ENST00000455450 | (GT)3 |
| THEMIS | ENSG00000172673 | ENST00000295899 | (CT)3 |
| THNSL2 | ENSG00000144115 | ENST00000280696 | (GCGCCCC)3 |
| THOC5 | ENSG00000100296 |  | (GCC)3 |
| THOC7 | ENSG00000163634 | ENST00000409968 | (CCT)3 |
| THRB | ENSG00000151090 | ENST00000524659  ENST00000636887 | (TGTA)3 |
|  |  |  | (TG)3 |
| THSD7B | ENSG00000144229 | ENST00000521943 | (CGG)3 |
| THY1 | ENSG00000154096 | ENST00000361424 | (GT)3 |
| TIAM1 | ENSG00000156299 | ENST00000642580 | (CT)3 |
| TLK1 | ENSG00000198586 | ENST00000504367 | (GA)3 |
| TLR10 | ENSG00000174123 | ENST00000436693 |  |
| TLR2 | ENSG00000137462 | ENST00000381950 | (TC)3 |
| TLR3 | ENSG00000164342 | ENST00000508254 | (CCA)3 |
| TLR6 | ENSG00000174130 | ENST00000561373 | (GAA)3 |
|  |  | ENST00000472441 |  |
|  |  | ENST00000446879 |  |
| TM2D3 | ENSG00000184277 | ENST00000454715 | (TC)3 |
| TM4SF1 | ENSG00000169908 | ENST00000371142 | (TG)3 |
| TM4SF19 | ENSG00000145107 | ENST00000547798 | (TC)3 |
|  |  | ENST00000545499 |  |
| TM9SF3 | ENSG00000077147 | ENST00000396667 | (GGA)3 |
| TMBIM6 | ENSG00000139644 | ENST00000442107 | (GCA)3 |
| TMCC2 | ENSG00000133069 | ENST00000511555 | (TG)3 |
| TMEM106B | ENSG00000106460 | ENST00000304511 | (TCC)3 |
|  |  | ENST00000393375 |  |
| TMEM108 | ENSG00000144868 | ENST00000438715 | (AT)3 |
| TMEM126A | ENSG00000171202 | ENST00000321639 | (AGG)3 |
| TMEM126B | ENSG00000171204 | ENST00000631683 | (GA)3 |
| TMEM131 | ENSG00000075568 | ENST00000381787 | (TC)3 |
| TMEM132E | ENSG00000181291 | ENST00000379542 | (CT)3 |
|  |  | ENST00000612333 |  |
| TMEM138 | ENSG00000149483 | ENST00000461342 | (CA)3 |
| TMEM14B | ENSG00000137210 | ENST00000475942 | (GA)3 |
|  |  | ENST00000379530 |  |
|  |  | ENST00000473276 |  |
|  |  | ENST00000304385 |  |
|  |  | ENST00000504064 |  |
|  |  | ENST00000468689 |  |
| TMEM154 | ENSG00000170006 | ENST00000401466 | (AG)3 |
|  |  |  |  |
| TMEM176A | ENSG00000002933 | ENST00000272521 | (GCA)3 |
| TMEM177 | ENSG00000144120 | ENST00000616017 | (CG)3 |
|  |  |  | (GCG)3 |
|  |  |  | (CG)3 |
|  |  |  | (GCG)3 |
| TMEM179 | ENSG00000258986 | ENST00000639249  ENST00000409528  ENST00000640575 | (GT)3 |
|  |  |  |  |
|  |  |  |  |
|  |  |  |  |
| TMEM182 | ENSG00000170417 | ENST00000414730 | (CA)3 |
|  |  | ENST00000422233 |  |
|  |  | ENST00000433641 |  |
| TMEM184A | ENSG00000164855 | ENST00000296978 | (CCTC)3 |
| TMEM196 | ENSG00000173452 |  | (TG)3 |
|  |  | ENST00000330115 |  |
| TMEM200A | ENSG00000164484 | ENST00000378602  ENST00000371344 | (CA)3 |
|  |  |  | (AG)3 |
| TMEM44 | ENSG00000145014 |  | (TG)3 |
| TMEM52 | ENSG00000178821 | ENST00000519519 | (GA)3 |
| TMEM59 | ENSG00000116209 | ENST00000521852 | (AT)3 |
|  |  |  |  |
| TMEM64 | ENSG00000180694 | ENST00000409623 | (AGG)3 |
|  |  |  | (CA)3 |
|  |  | ENST00000544540  ENST00000537354 | (AGG)3 |
|  |  |  | (CA)3 |
| TMEM67 | ENSG00000164953 | ENST00000395149 | (GT)3 |
| TMEM72 | ENSG00000187783 | ENST00000583437 | (CTG)3 |
| TMEM91 | ENSG00000142046 | ENST00000309134 | (AGA)3 |
| TMEM98 | ENSG00000006042 | ENST00000528117 | (CT)3 |
|  |  | ENST00000332149 |  |
| TMEM9B | ENSG00000175348 | ENST00000256062 | (TGC)3 |
|  |  | ENST00000524158 |  |
| TMPRSS2 | ENSG00000184012 | ENST00000481401 | (GGAGGC)3 |
| TMTC1 | ENSG00000133687 | ENST00000396542 | (TC)3 |
| TNFRSF10A | ENSG00000104689 | ENST00000438470 | (AG)3 |
| TNFRSF25 | ENSG00000215788 | ENST00000315050 | (CCTG)3 |
| TNFSF13 | ENSG00000161955 | ENST00000523338 | (TG)3 |
|  |  | ENST00000522100 |  |
| TNIP1 | ENSG00000145901 | ENST00000520695 | (CA)3 |
|  |  | ENST00000057513 |  |
|  |  | ENST00000641119 |  |
|  |  | ENST00000641787 |  |
| TNIP3 | ENSG00000050730 | ENST00000434361 | (TG)3 |
| TNNT3 | ENSG00000130595 | ENST00000446688 | (CT)3 |
|  |  | ENST00000450444 |  |
| TNRC18 | ENSG00000182095 | ENST00000451343 | (CGG)3 |
| TNS1 | ENSG00000079308 | ENST00000608674 | (AGG)3 |
| TNS3 | ENSG00000136205 | ENST00000443206 | (AG)3 |
| TNXB | ENSG00000168477 | ENST00000424225 | (AC)3 |
| TOM1 | ENSG00000100284 | ENST00000437929 | (AG)3 |
|  |  | ENST00000437103 | (GT)3 |
| TOP2B | ENSG00000077097 | ENST00000435319 |  |
| TOP3B | ENSG00000100038 | ENST00000407228 | (CTT)3 |
|  |  | ENST00000525138 |  |
| TOR1AIP1 | ENSG00000143337 | ENST00000398682 | (GA)3 |
| TOX3 | ENSG00000103460 | ENST00000569420 | (AG)3 |
| TP53I11 | ENSG00000175274 | ENST00000341305 | (CAG)3 |
| TP53TG3 | ENSG00000183632 | ENST00000569741 | (GA)3 |
|  |  | ENST00000586499 |  |
| TP53TG3B | ENSG00000261509 | ENST00000647464 |  |
|  |  | ENST00000588483 |  |
| TPM4 | ENSG00000167460 | ENST00000592138 | (CT)3 |
|  |  | ENST00000299427 | (GAG)3 |
|  |  | ENST00000400103 |  |
|  |  | ENST00000303434 |  |
| TPP1 | ENSG00000166340 | ENST00000348124 | (GTG)3 |
| TPTE2 | ENSG00000132958 | ENST00000567645 | (CTA)3 |
| TRABD | ENSG00000170638 | ENST00000548092 | (GC)3 |
| TRAF6 | ENSG00000175104 | ENST00000310754 | (GA)3 |
| TRAF7 | ENSG00000131653 | ENST00000561840 | (TGG)3 |
| TRAFD1 | ENSG00000135148 | ENST00000373162 | (AG)3 |
| TRAM1L1 | ENSG00000174599 | ENST00000596148 | (GGC)3 |
| TRAPPC2L | ENSG00000167515 | ENST00000317378 | (CA)3 |
| TRAPPC3 | ENSG00000054116 | ENST00000426877 | (CAA)3 |
| TRAPPC5 | ENSG00000181029 | ENST00000433541 | (GGC)3 |
|  |  | ENST00000492235 |  |
|  |  | ENST00000635452 |  |
| TREX1 | ENSG00000213689 | ENST00000330912 | (CA)3 |
|  |  | ENST00000338525 |  |
|  |  | ENST00000370231 |  |
| TREX2 | ENSG00000183479 | ENST00000619857 | (TG)3 |
|  |  | ENST00000494872 |  |
|  |  | ENST00000437089 |  |
| TRIM15 | ENSG00000204610 | ENST00000526881 | (AC)3 |
| TRIM2 | ENSG00000109654 | ENST00000272395 | (GCA)3 |
| TRIM26 | ENSG00000234127 | ENST00000507119 | (GC)3 |
| TRIM29 | ENSG00000137699 | ENST00000269383 | (CT)3 |
| TRIM43 | ENSG00000144015 |  | (AT)3 |
| TRIM60 | ENSG00000176979 | ENST00000526337 | (TG)3 |
| TRIM65 | ENSG00000141569 | ENST00000354852  ENST00000323819 | (GC)3 |
|  |  |  | (CGC)3 |
| TRIM68 | ENSG00000167333 | ENST00000430211 | (AGC)3 |
| TRIM6-TRIM34 | ENSG00000258588 | ENST00000417857 | (GC)3 |
| TRIM73 | ENSG00000178809 |  | (AG)3 |
|  |  | ENST00000435716 |  |
| TRIOBP | ENSG00000100106 | ENST00000403707  ENST00000420393 | (TC)3 |
|  |  |  | (GA)3 |
| TRIP12 | ENSG00000153827 | ENST00000402675 | (ACC)3 |
| TRMT2A | ENSG00000099899 | ENST00000452830 | (GGCG)3 |
| TRNT1 | ENSG00000072756 | ENST00000453081 | (TA)3 |
|  |  | ENST00000409625 |  |
| TRO | ENSG00000067445 | ENST00000611198 | (GT)3 |
|  |  | ENST00000402228 |  |
| TRPM8 | ENSG00000144481 | ENST00000434359 | (TG)3 |
| TSC22D1 | ENSG00000102804 | ENST00000457189 | (AG)3 |
| TSEN2 | ENSG00000154743 | ENST00000520462 | (GA)3 |
| TSFM | ENSG00000123297 | ENST00000429989 | (CA)3 |
|  |  |  |  |
| TSNARE1 | ENSG00000171045 | ENST00000481124 | (CG)3 |
| TSPAN14 | ENSG00000108219 | ENST00000372164 | (GCC)3 |
|  |  |  | (CG)3 |
|  |  | ENST00000341863 | (GCC)3 |
|  |  |  | (CG)3 |
|  |  | ENST00000552816 | (GCC)3 |
|  |  |  | (CG)3 |
|  |  | ENST00000548167  ENST00000451520 | (GCC)3 |
|  |  |  | (CG)3 |
| TSPAN31 | ENSG00000135452 |  | (ACC)3 |
|  |  | ENST00000397411 |  |
| TSPAN32 | ENSG00000064201 | ENST00000397396 | (TC)3 |
|  |  |  | (GA)3 |
| TSPAN4 | ENSG00000214063 | ENST00000525334 | (GGC)3 |
|  |  | ENST00000525201 | (CA)3 |
|  |  |  | (TCA)3 |
|  |  | ENST00000451548 | (CA)3 |
|  |  |  | (TCA)3 |
|  |  | ENST00000428845  ENST00000429039 | (CA)3 |
|  |  |  | (TCA)3 |
| TSPY1 | ENSG00000258992 | ENST00000320701 | (CG)3 |
| TSPY10 | ENSG00000236424 | ENST00000457222 |  |
| TSPY2 | ENSG00000168757 | ENST00000426950 |  |
|  |  | ENST00000287721 |  |
| TSPY3 | ENSG00000228927 | ENST00000322128 |  |
| TSPY4 | ENSG00000233803 | ENST00000368236 |  |
| TSPY8 | ENSG00000229549 | ENST00000448773 |  |
| TSPYL5 | ENSG00000180543 | ENST00000397906 | (GA)3 |
| TTC24 | ENSG00000187862 | ENST00000579214 | (AG)3 |
| TTC27 | ENSG00000018699 | ENST00000530625 | (GA)3 |
| TTC28 | ENSG00000100154 | ENST00000373448 | (GCG)3 |
| TTC39C | ENSG00000168234 | ENST00000321582 | (AG)3 |
| TTC9C | ENSG00000162222 | ENST00000438596 | (GC)3 |
| TTI1 | ENSG00000101407 | ENST00000417065 | (CAC)3 |
| TTLL11 | ENSG00000175764 | ENST00000554510 | (CG)3 |
| TTLL3 | ENSG00000214021 | ENST00000375938 | (CT)3 |
|  |  | ENST00000398989 |  |
| TTLL5 | ENSG00000119685 | ENST00000427737 | (AT)3 |
| TTLL9 | ENSG00000131044 |  | (CT)3 |
| TUBA4A | ENSG00000127824 | ENST00000543663 | (GGA)3 |
|  |  | ENST00000368563  ENST00000368562 | (GCG)3 |
|  |  |  | (GGA)3 |
| TUBGCP2 | ENSG00000130640 | ENST00000313511 | (CCG)3 |
|  |  | ENST00000518572 |  |
|  |  | ENST00000520977 | (GC)3 |
| TUFM | ENSG00000178952 | ENST00000358022 | (TTC)3 |
| TULP2 | ENSG00000104804 |  | (AG)3 |
|  |  |  | (CT)3 |
| TUSC1 | ENSG00000198680 | ENST00000552521  ENST00000546506  ENST00000305533 | (GCC)3 |
|  |  |  | (CTC)3 |
|  |  |  | (CG)3 |
| TWF1 | ENSG00000151239 | ENST00000499914 | (TA)3 |
|  |  | ENST00000403313 |  |
| TWF2 | ENSG00000247596 | ENST00000409705 | (CT)3 |
|  |  | ENST00000524462 |  |
| TXN2 | ENSG00000100348 | ENST00000473763 | (TG)3 |
| TXNDC9 | ENSG00000115514 |  | (AC)3 |
| TYK2 | ENSG00000105397 | ENST00000468022 | (GAG)3 |
| TYRP1 | ENSG00000107165 | ENST00000494238  ENST00000464068 | (AT)3 |
|  |  |  | (GA)3 |
| UBA5 | ENSG00000081307 | ENST00000598780 | (GT)3 |
|  |  | ENST00000425792 |  |
|  |  | ENST00000452894 |  |
| UBA52 | ENSG00000221983 | ENST00000396703 | (GA)3 |
| UBE2E2 | ENSG00000182247 | ENST00000502382 | (GCG)3 |
|  |  | ENST00000486663 |  |
|  |  | ENST00000456378 | (CT)3 |
| UBE2J2 | ENSG00000160087 | ENST00000375225 | (TG)3 |
| UBN2 | ENSG00000157741 | ENST00000375218 | (AATT)3 |
| UBP1 | ENSG00000153560 | ENST00000505698 | (CT)3 |
| UBR4 | ENSG00000127481 | ENST00000406651 | (AT)3 |
|  |  | ENST00000274278 | (CAC)3 |
| UGDH | ENSG00000109814 | ENST00000503189 | (CT)3 |
| UGT1A6 | ENSG00000167165 | ENST00000507113 | (AG)3 |
| UGT3A1 | ENSG00000145626 | ENST00000625798 | (GC)3 |
|  |  | ENST00000510698 |  |
|  |  | ENST00000367351 |  |
|  |  | ENST00000321867 | (TG)3 |
| UIMC1 | ENSG00000087206 | ENST00000570689 | (GGA)3 |
| ULBP2 | ENSG00000131015 | ENST00000453304 | (TC)3 |
| ULK1 | ENSG00000177169 | ENST00000513796 | (GCCC)3 |
| UMOD | ENSG00000169344 | ENST00000506749 | (GT)3 |
| UNC5C | ENSG00000182168 | ENST00000504962 | (TC)3 |
|  |  | ENST00000404895 |  |
|  |  | ENST00000605860 |  |
|  |  | ENST00000005756 |  |
| UNC5D | ENSG00000156687 | ENST00000591899 | (CT)3 |
| UPP2 | ENSG00000007001 | ENST00000514213 | (CG)3 |
|  |  | ENST00000264904 | (TC)3 |
| UQCR11 | ENSG00000127540 | ENST00000546694 | (GC)3 |
| USO1 | ENSG00000138768 | ENST00000392784 | (GT)3 |
|  |  | ENST00000398571 |  |
| USP15 | ENSG00000135655 | ENST00000528910 | (AG)3 |
| USP30 | ENSG00000135093 | ENST00000409945 | (GT)3 |
| USP34 | ENSG00000115464 | ENST00000433394 | (TC)3 |
| USP35 | ENSG00000118369 | ENST00000566004 | (GAG)3 |
| USP40 | ENSG00000085982 | ENST00000569230 | (AG)3 |
| USP54 | ENSG00000166348 | ENST00000566273 | (CTT)3 |
| USP7 | ENSG00000187555 | ENST00000564117 | (GGA)3 |
|  |  | ENST00000254803 |  |
|  |  | ENST00000432686 | (GT)3 |
|  |  | ENST00000417142 | (GGA)3 |
| UTP3 | ENSG00000132467 | ENST00000536184 | (GC)3 |
| UTRN | ENSG00000152818 | ENST00000366966 | (AT)3 |
|  |  | ENST00000366968 |  |
| VAC14 | ENSG00000103043 | ENST00000490792 | (AGG)3 |
| VASH2 | ENSG00000143494 | ENST00000381059 | (GAG)3 |
|  |  | ENST00000620630 |  |
|  |  | ENST00000317103 |  |
| VCX | ENSG00000182583 | ENST00000381089 | (TA)3 |
|  |  | ENST00000612369 |  |
| VCX2 | ENSG00000177504 | ENST00000381032 |  |
| VCX3A | ENSG00000169059 | ENST00000453306 |  |
|  |  | ENST00000444481 |  |
| VCX3B | ENSG00000205642 | ENST00000298468 |  |
|  |  | ENST00000543351 |  |
|  |  | ENST00000313132 |  |
| VDAC2 | ENSG00000165637 | ENST00000518563 | (CA)3 |
|  |  | ENST00000498155 |  |
|  |  |  | (GC)3 |
| VDAC3 | ENSG00000078668 | ENST00000472468 | (AC)3 |
| VKORC1 | ENSG00000167397 | ENST00000321039  ENST00000530536 | (GA)3 |
|  |  |  |  |
|  |  | ENST00000371248 | (AC)3 |
| VN1R1 | ENSG00000178201 | ENST00000413141 |  |
| VNN2 | ENSG00000112303 | ENST00000418457 | (CTC)3 |
| VPS37B | ENSG00000139722 | ENST00000643970 | (GA)3 |
| VPS41 | ENSG00000006715 | ENST00000369128 | (CA)3 |
|  |  | ENST00000440705 | (AG)3 |
| VPS45 | ENSG00000136631 | ENST00000428021 | (CT)3 |
|  |  | ENST00000603594 |  |
| VRK2 | ENSG00000028116 | ENST00000628285 | (GA)3 |
|  |  | ENST00000557297 | (GT)3 |
| VWA2 | ENSG00000165816 | ENST00000429900 | (AG)3 |
| WAC | ENSG00000095787 | ENST00000419837 | (CT)3 |
| WARS | ENSG00000140105 | ENST00000397143 | (CA)3 |
| WDR6 | ENSG00000178252 | ENST00000371026 | (TG)3 |
|  |  | ENST00000488333 |  |
| WDR60 | ENSG00000126870 | ENST00000477459 | (AG)3 |
| WDR78 | ENSG00000152763 | ENST00000628331 | (CG)3 |
|  |  | ENST00000621812 | (CA)3 |
| WDR86 | ENSG00000187260 | ENST00000592765 | (AC)3 |
|  |  | ENST00000361680 |  |
|  |  | ENST00000406245 |  |
| WDR88 | ENSG00000166359 | ENST00000342873 | (GCGGGC)3 |
|  |  | ENST00000583268 |  |
| WDR92 | ENSG00000243667 | ENST00000599686 | (AT)3 |
| WFDC2 | ENSG00000101443 | ENST00000596159 | (AGG)3 |
| WIPF2 | ENSG00000171475 | ENST00000410089 | (TCC)3 |
| WIZ | ENSG00000011451 | ENST00000398753 | (CCA)3 |
|  |  | ENST00000442773 | (CG)3 |
| WNT7B | ENSG00000188064 | ENST00000573634 | (TC)3 |
| WRB | ENSG00000182093 | ENST00000526685 | (TCC)3 |
|  |  | ENST00000356003 | (GA)3 |
| WSCD1 | ENSG00000179314 | ENST00000460517 | (CAC)3 |
| WT1 | ENSG00000184937 | ENST00000375602 | (AC)3 |
| WWP2 | ENSG00000198373 | ENST00000375600 | (GA)3 |
| WWTR1 | ENSG00000018408 | ENST00000375616 | (CTC)3 |
| XAGE1A | ENSG00000204379 | ENST00000375613 | (CA)3 |
|  |  | ENST00000518075 |  |
| XAGE1B | ENSG00000204382 | ENST00000375491 |  |
|  |  | ENST00000405219 |  |
|  |  | ENST00000355640 |  |
| XAGE3 | ENSG00000171402 | ENST00000401558 |  |
| XBP1 | ENSG00000100219 | ENST00000457483 | (CT)3 |
| XIAP | ENSG00000101966 | ENST00000565698 | (GTGG)3 |
| XPO1 | ENSG00000082898 |  | (AGGA)3 |
|  |  | ENST00000570033 | (GTG)3 |
| XPO6 | ENSG00000169180 | ENST00000566073 | (GT)3 |
|  |  |  | (CT)3 |
|  |  | ENST00000517551 |  |
|  |  | ENST00000525407  ENST00000549685 | (GT)3 |
|  |  |  | (CT)3 |
| XPO7 | ENSG00000130227 | ENST00000496746 | (AT)3 |
| XRRA1 | ENSG00000166435 | ENST00000392124 | (GC)3 |
| YEATS4 | ENSG00000127337 | ENST00000238831 | (AGA)3 |
| YIF1A | ENSG00000174851 | ENST00000415895 | (TCC)3 |
| YIF1B | ENSG00000167645 | ENST00000508947 | (CT)3 |
| YIPF4 | ENSG00000119820 |  | (CGC)3 |
| YIPF7 | ENSG00000177752 | ENST00000419477 | (CA)3 |
|  |  | ENST00000368339 | (CT)3 |
|  |  |  | (TC)3 |
| YWHAZ | ENSG00000164924 | ENST00000368340 | (GC)3 |
| YY1AP1 | ENSG00000163374 | ENST00000368330 | (GGT)3 |
|  |  |  | (GC)3 |
|  |  | ENST00000405763  ENST00000554015 | (GGT)3 |
|  |  |  | (GC)3 |
|  |  |  | (GGA)3 |
|  |  | ENST00000449886 | (GC)3 |
| ZBTB1 | ENSG00000126804 | ENST00000450858  ENST00000354590 | (AG)3 |
|  |  |  | (TG)3 |
| ZBTB43 | ENSG00000169155 | ENST00000594051 | (AG)3 |
|  |  | ENST00000600130 |  |
| ZBTB45 | ENSG00000119574 | ENST00000601588 | (GCGGG)3 |
|  |  | ENST00000465588 | (CGC)3 |
|  |  | ENST00000242848 | (GC)3 |
| ZBTB7A | ENSG00000178951 | ENST00000490721 | (GCC)3 |
| ZBTB8OS | ENSG00000176261 |  | (TC)3 |
| ZC3H13 | ENSG00000123200 | ENST00000511193 | (CGT)3 |
| ZCWPW1 | ENSG00000078487 | ENST00000550876  ENST00000436556 | (TC)3 |
|  |  |  |  |
| ZDHHC11 | ENSG00000188818 | ENST00000613266 | (TTC)3 |
| ZDHHC17 | ENSG00000186908 | ENST00000561060 | (TG)3 |
| ZFAND2B | ENSG00000158552 | ENST00000559157 | (AG)3 |
| ZFAND6 | ENSG00000086666 | ENST00000564367 | (GA)3 |
|  |  | ENST00000558494 |  |
|  |  | ENST00000517585 |  |
|  |  | ENST00000557086 |  |
|  |  | ENST00000557022 |  |
| ZFHX4 | ENSG00000091656 | ENST00000520584 | (GT)3 |
| ZFP36L1 | ENSG00000185650 | ENST00000330701 | (ACC)3 |
|  |  | ENST00000379177 | (GGC)3 |
| ZFP41 | ENSG00000181638 | ENST00000394207 | (AG)3 |
|  |  | ENST00000555072 |  |
| ZFX | ENSG00000005889 | ENST00000572863 | (TTC)3 |
| ZFYVE1 | ENSG00000165861 | ENST00000463250 | (AGG)3 |
|  |  | ENST00000620447 |  |
| ZG16B | ENSG00000162078 | ENST00000442887 | (CT)3 |
| ZIC4 | ENSG00000174963 | ENST00000248211 | (CG)3 |
| ZMYM2 | ENSG00000121741 | ENST00000358296 | (CT)3 |
| ZMYND10 | ENSG00000004838 | ENST00000598026 | (CTG)3 |
| ZNF10 | ENSG00000256223 | ENST00000613690 | (TGC)3 |
| ZNF100 | ENSG00000197020 | ENST00000620827 | (TG)3 |
|  |  | ENST00000282869 |  |
| ZNF107 | ENSG00000196247 | ENST00000620222 | (CTC)3 |
|  |  | ENST00000610793 |  |
| ZNF117 | ENSG00000152926 | ENST00000439995 | (GA)3 |
|  |  | ENST00000429434 |  |
|  |  | ENST00000601421 |  |
| ZNF136 | ENSG00000196646 | ENST00000601982 | (AA)3 |
| ZNF140 | ENSG00000196387 | ENST00000318529 | (TA)3 |
| ZNF160 | ENSG00000170949 | ENST00000429541 | (TC)3 |
|  |  | ENST00000534569 |  |
| ZNF185 | ENSG00000147394 | ENST00000396868 | (CT)3 |
| ZNF195 | ENSG00000005801 | ENST00000396871 | (GA)3 |
|  |  | ENST00000575617 |  |
| ZNF200 | ENSG00000010539 | ENST00000579634 | (GGC)3 |
|  |  | ENST00000580759 |  |
|  |  | ENST00000342555 |  |
| ZNF207 | ENSG00000010244 | ENST00000394679 | (AT)3 |
|  |  | ENST00000582165 |  |
|  |  | ENST00000578918 | (AG)3 |
|  |  | ENST00000599916 | (AT)3 |
|  |  | ENST00000601773 |  |
|  |  | ENST00000609966 |  |
| ZNF208 | ENSG00000160321 | ENST00000397126 | (TG)3 |
|  |  | ENST00000597040 |  |
|  |  | ENST00000360947 |  |
|  |  |  |  |
|  |  | ENST00000554478 | (TC)3 |
| ZNF219 | ENSG00000165804 | ENST00000554923 | (CGC)3 |
|  |  |  | (CCG)3 |
|  |  | ENST00000251269 | (GA)3 |
|  |  | ENST00000594839  ENST00000339844 | (TG)3 |
|  |  |  | (AG)3 |
| ZNF221 | ENSG00000159905 | ENST00000579694 | (TA)3 |
| ZNF274 | ENSG00000171606 | ENST00000303809 | (GA)3 |
| ZNF28 | ENSG00000198538 | ENST00000505163 | (TC)3 |
| ZNF286A | ENSG00000187607 | ENST00000598312 | (GA)3 |
| ZNF296 | ENSG00000170684 | ENST00000358067 | (TGCC)3 |
| ZNF302 | ENSG00000089335 | ENST00000625284 | (GGC)3 |
| ZNF329 | ENSG00000181894 | ENST00000359467 | (CA)3 |
|  |  | ENST00000493285 |  |
| ZNF334 | ENSG00000198185 | ENST00000586646 |  |
| ZNF33B | ENSG00000196693 | ENST00000595967 | (CT)3 |
|  |  | ENST00000435416 |  |
| ZNF345 | ENSG00000251247 | ENST00000546970 | (AC)3 |
| ZNF347 | ENSG00000197937 | ENST00000517459 | (TC)3 |
| ZNF382 | ENSG00000161298 | ENST00000556179 | (CA)3 |
| ZNF385A | ENSG00000161642 | ENST00000601493 | (AGA)3 |
| ZNF395 | ENSG00000186918 | ENST00000595830 | (AGAGG)3 |
| ZNF410 | ENSG00000119725 | ENST00000396147 | (GA)3 |
| ZNF415 | ENSG00000170954 |  | (AT)3 |
| ZNF418 | ENSG00000196724 | ENST00000600989 | (AC)3 |
|  |  | ENST00000599852  ENST00000616958 | (GGC)3 |
|  |  |  | (AC)3 |
|  |  | ENST00000601593 |  |
|  |  | ENST00000593296 | (GT)3 |
|  |  | ENST00000598288 | (AC)3 |
|  |  | ENST00000478765 |  |
|  |  | ENST00000455282 |  |
| ZNF43 | ENSG00000198521 |  | (AGG)3 |
| ZNF433 | ENSG00000197647 | ENST00000457526 | (AG)3 |
| ZNF439 | ENSG00000171291 | ENST00000242804  ENST00000438182 | (AGA)3 |
|  |  |  | (GT)3 |
| ZNF440 | ENSG00000171295 | ENST00000588140 |  |
| ZNF442 | ENSG00000198342 |  | (AC)3 |
|  |  | ENST00000601847 | (GA)3 |
| ZNF45 | ENSG00000124459 | ENST00000335090 | (GT)3 |
|  |  |  | (GAG)3 |
| ZNF468 | ENSG00000204604 | ENST00000361807 | (TC)3 |
| ZNF480 | ENSG00000198464 | ENST00000430885  ENST00000585316 | (TG)3 |
|  |  |  | (TC)3 |
| ZNF485 | ENSG00000198298 |  | (CT)3 |
|  |  | ENST00000456783 |  |
| ZNF488 | ENSG00000265763 | ENST00000596302  ENST00000392288 | (AG)3 |
|  |  |  | (GT)3 |
| ZNF492 | ENSG00000229676 | ENST00000594390 | (TC)3 |
| ZNF493 | ENSG00000196268 | ENST00000355504 | (CT)3 |
|  |  | ENST00000595763 |  |
|  |  | ENST00000295208 |  |
|  |  | ENST00000474037 | (GA)3 |
| ZNF497 | ENSG00000174586 | ENST00000491101 | (TG)3 |
| ZNF514 | ENSG00000144026 | ENST00000467003 | (GAG)3 |
| ZNF525 | ENSG00000203326 | ENST00000588456 | (CG)3 |
|  |  | ENST00000591049 |  |
|  |  | ENST00000596282 | (TC)3 |
| ZNF532 | ENSG00000074657 | ENST00000391701 | (CA)3 |
|  |  | ENST00000603380 |  |
| ZNF548 | ENSG00000188785 | ENST00000592504 | (GGT)3 |
| ZNF552 | ENSG00000178935 | ENST00000592298 | (GTG)3 |
| ZNF559 | ENSG00000188321 | ENST00000393883 | (GA)3 |
|  |  | ENST00000355114 |  |
|  |  | ENST00000586353 |  |
|  |  | ENST00000433993 |  |
| ZNF565 | ENSG00000196357 | ENST00000592567 | (CCT)3 |
| ZNF568 | ENSG00000198453 | ENST00000455817 | (GA)3 |
|  |  | ENST00000270451 |  |
|  |  | ENST00000531805 |  |
|  |  | ENST00000591492 |  |
| ZNF581 | ENSG00000171425 | ENST00000354698 | (CGC)3 |
| ZNF585B | ENSG00000245680 |  | (AG)3 |
|  |  |  | (TA)3 |
| ZNF589 | ENSG00000164048 | ENST00000427617 | (CA)3 |
|  |  |  | (CGTG)3 |
|  |  |  | (GC)3 |
|  |  | ENST00000412564 | (CA)3 |
|  |  |  | (CGTG)3 |
|  |  |  | (GC)3 |
|  |  | ENST00000440261 | (CA)3 |
|  |  |  | (CGTG)3 |
|  |  |  | (GC)3 |
|  |  | ENST00000608255  ENST00000521145  ENST00000320552 | (CA)3 |
|  |  |  | (CGTG)3 |
|  |  |  | (GC)3 |
| ZNF595 | ENSG00000272602 | ENST00000640035 | (GA)3 |
| ZNF596 | ENSG00000172748 | ENST00000521270 | (CG)3 |
|  |  | ENST00000563630 |  |
|  |  | ENST00000562103 |  |
|  |  | ENST00000403906 | (CA)3 |
| ZNF598 | ENSG00000167962 | ENST00000601151 | (GGC)3 |
|  |  | ENST00000593698 |  |
| ZNF610 | ENSG00000167554 | ENST00000327920 | (CA)3 |
|  |  | ENST00000319783 |  |
|  |  |  |  |
|  |  | ENST00000595798 |  |
| ZNF611 | ENSG00000213020 | ENST00000593650  ENST00000466975 | (GT)3 |
|  |  |  | (GA)3 |
|  |  | ENST00000550342 |  |
| ZNF615 | ENSG00000197619 | ENST00000339948 |  |
| ZNF638 | ENSG00000075292 | ENST00000397121 | (AG)3 |
| ZNF641 | ENSG00000167528 |  | (TG)3 |
| ZNF648 | ENSG00000179930 | ENST00000598513 | (CTC)3 |
| ZNF676 | ENSG00000196109 | ENST00000594517  ENST00000599012 | (AT)3 |
|  |  |  | (TC)3 |
| ZNF677 | ENSG00000197928 | ENST00000594681 | (CTT)3 |
|  |  | ENST00000598806 |  |
|  |  | ENST00000601828 |  |
|  |  | ENST00000601413 |  |
|  |  | ENST00000397162 |  |
|  |  | ENST00000358523 |  |
|  |  | ENST00000601100 |  |
| ZNF682 | ENSG00000197124 | ENST00000372507 | (TC)3 |
|  |  | ENST00000518432 |  |
|  |  | ENST00000528372 |  |
| ZNF691 | ENSG00000164011 | ENST00000611267 | (CA)3 |
| ZNF696 | ENSG00000185730 | ENST00000396570 | (CCG)3 |
| ZNF7 | ENSG00000147789 | ENST00000610508 | (GCG)3 |
| ZNF701 | ENSG00000167562 | ENST00000359286 | (GA)3 |
| ZNF705A | ENSG00000196946 | ENST00000400120 | (AG)3 |
|  |  | ENST00000400085 |  |
|  |  | ENST00000400156 |  |
| ZNF705B | ENSG00000215356 | ENST00000526970 |  |
| ZNF705D | ENSG00000215343 | ENST00000616183 |  |
| ZNF705G | ENSG00000215372 |  |  |
| ZNF707 | ENSG00000181135 | ENST00000596053 | (CA)3 |
| ZNF714 | ENSG00000160352 | ENST00000618422 | (TA)3 |
|  |  |  | (GA)3 |
|  |  | ENST00000456283 | (TC)3 |
|  |  | ENST00000618008  ENST00000425625 | (GA)3 |
|  |  |  | (TC)3 |
|  |  | ENST00000620627 |  |
|  |  | ENST00000468296 |  |
|  |  | ENST00000601693 |  |
|  |  | ENST00000593635 |  |
| ZNF717 | ENSG00000227124 | ENST00000415248 | (GT)3 |
| ZNF729 | ENSG00000196350 | ENST00000592625 | (TG)3 |
| ZNF730 | ENSG00000183850 | ENST00000343949 | (TC)3 |
| ZNF749 | ENSG00000186230 | ENST00000358987 | (GT)3 |
| ZNF763 | ENSG00000197054 | ENST00000439461 | (AG)3 |
|  |  | ENST00000595149 |  |
|  |  | ENST00000595000 |  |
| ZNF766 | ENSG00000196214 | ENST00000427512 | (GC)3 |
|  |  | ENST00000610548 | (TC)3 |
|  |  | ENST00000306502 |  |
| ZNF772 | ENSG00000197128 | ENST00000450241 | (GGC)3 |
|  |  | ENST00000593605 |  |
| ZNF778 | ENSG00000170100 | ENST00000358582 | (AG)3 |
| ZNF780A | ENSG00000197782 | ENST00000590008 | (TC)3 |
| ZNF780B | ENSG00000128000 | ENST00000302277 | (TAT)3 |
| ZNF781 | ENSG00000196381 |  | (TG)3 |
|  |  | ENST00000426391 |  |
| ZNF804A | ENSG00000170396 | ENST00000550826 | (GC)3 |
|  |  |  | (GGC)3 |
| ZNF841 | ENSG00000197608 | ENST00000592859  ENST00000436261 | (TC)3 |
|  |  |  | (TG)3 |
| ZNF844 | ENSG00000223547 | ENST00000424032 | (CA)3 |
| ZNF846 | ENSG00000196605 | ENST00000599743 | (GA)3 |
| ZNF852 | ENSG00000178917 | ENST00000300619 | (TC)3 |
| ZNF880 | ENSG00000221923 |  | (GC)3 |
| ZNF91 | ENSG00000167232 | ENST00000397082 | (TG)3 |
|  |  | ENST00000595533  ENST00000357774 | (TC)3 |
|  |  |  | (TG)3 |
|  |  | ENST00000593657 |  |
|  |  | ENST00000601553 |  |
| ZNF98 | ENSG00000197360 | ENST00000332435 | (TC)3 |
|  |  | ENST00000336517 |  |
|  |  | ENST00000416245 |  |
| ZNRD1 | ENSG00000066379 | ENST00000421612 | (TA)3 |
| ZP3 | ENSG00000188372 | ENST00000358697 | (AC)3 |
|  |  | ENST00000374888 | (CT)3 |
| ZSCAN18 | ENSG00000121413 | ENST00000392910 | (AG)3 |
| ZXDA | ENSG00000198205 |  | (CCT)3 |
| ZXDB | ENSG00000198455 |  |  |
| ZYX | ENSG00000159840 |  | (GGA)3 |
